# Traceless enzymatic protein synthesis without ligation sites constraint

**DOI:** 10.1093/nsr/nwab158

**Published:** 2021-08-24

**Authors:** Ruifeng Li, Marcel Schmidt, Tong Zhu, Xinyu Yang, Jing Feng, Yu’e Tian, Yinglu Cui, Timo Nuijens, Bian Wu

**Affiliations:** CAS Key Laboratory of Microbial Physiological and Metabolic Engineering, State Key Laboratory of Microbial Resources, Institute of Microbiology, Chinese Academy of Sciences, Beijing 100101, China; University of Chinese Academy of Sciences, Beijing 100049, China; Fresenius Kabi iPSUM, I&D Center EnzyPep B.V., Geleen 6167 RD, the Netherlands; CAS Key Laboratory of Microbial Physiological and Metabolic Engineering, State Key Laboratory of Microbial Resources, Institute of Microbiology, Chinese Academy of Sciences, Beijing 100101, China; University of Chinese Academy of Sciences, Beijing 100049, China; CAS Key Laboratory of Microbial Physiological and Metabolic Engineering, State Key Laboratory of Microbial Resources, Institute of Microbiology, Chinese Academy of Sciences, Beijing 100101, China; University of Chinese Academy of Sciences, Beijing 100049, China; CAS Key Laboratory of Microbial Physiological and Metabolic Engineering, State Key Laboratory of Microbial Resources, Institute of Microbiology, Chinese Academy of Sciences, Beijing 100101, China; University of Chinese Academy of Sciences, Beijing 100049, China; CAS Key Laboratory of Microbial Physiological and Metabolic Engineering, State Key Laboratory of Microbial Resources, Institute of Microbiology, Chinese Academy of Sciences, Beijing 100101, China; CAS Key Laboratory of Microbial Physiological and Metabolic Engineering, State Key Laboratory of Microbial Resources, Institute of Microbiology, Chinese Academy of Sciences, Beijing 100101, China; Fresenius Kabi iPSUM, I&D Center EnzyPep B.V., Geleen 6167 RD, the Netherlands; CAS Key Laboratory of Microbial Physiological and Metabolic Engineering, State Key Laboratory of Microbial Resources, Institute of Microbiology, Chinese Academy of Sciences, Beijing 100101, China

**Keywords:** protein synthesis, traceless peptide ligation, biocatalysis, synthetic biology, chemical biology

## Abstract

Protein synthesis and semisynthesis offer immense promise for life sciences and have impacted pharmaceutical innovation. The absence of a generally applicable method for traceless peptide conjugation with a flexible choice of junction sites remains a bottleneck for accessing many important synthetic targets, however. Here we introduce the PALME (protein activation and ligation with multiple enzymes) platform designed for sequence-unconstrained synthesis and modification of biomacromolecules. The upstream activating modules accept and process easily accessible synthetic peptides and recombinant proteins, avoiding the challenges associated with preparation and manipulation of activated peptide substrates. Cooperatively, the downstream coupling module provides comprehensive solutions for sequential peptide condensation, cyclization and protein N/C-terminal or internal functionalization. The practical utility of this methodology is demonstrated by synthesizing a series of bioactive targets ranging from pharmaceutical ingredients to synthetically challenging proteins. The modular PALME platform exhibits unprecedentedly broad accessibility for traceless protein synthesis and functionalization, and holds enormous potential to extend the scope of protein chemistry and synthetic biology.

## Teaser

A multi-enzyme platform is constructed for sequence-unconstrained traceless protein synthesis and modification with either synthetic peptides or recombinant proteins.

## INTRODUCTION

**Figure 1.**
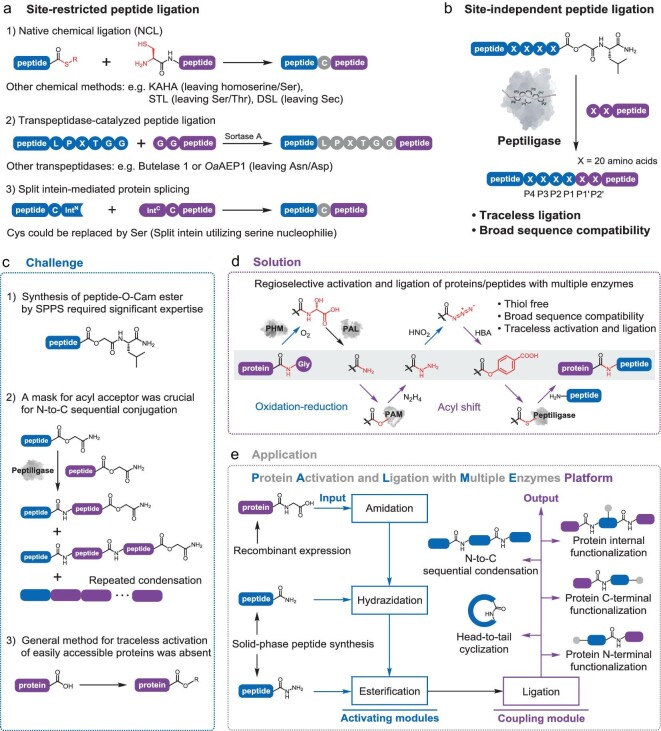
Overview of the PALME platform. (a) Reported chemical and enzymatic methods for site-restricted peptide ligation. Residues remaining at the ligation junction (scar) are colored grey. (b) Site-independent peptide ligation via an engineered subtilisin-derived ligase termed peptiligase. (c) The challenges faced in peptiligase-catalyzed peptide ligation to date. (d) Our solution as reported in the present study. (e) Flexible multi-input and multi-output options of the PALME platform.

Breaking away from the central dogma, protein total synthesis and semisynthesis are powerful strategies for generating and functionalizing naturally inaccessible proteins, which have enabled groundbreaking applications driving life science advances and impacted the industrial production of biomolecular therapeutics [1–3]. Solid-phase peptide synthesis (SPPS) [4] has been developed as an effective means of incorporating the full spectrum of chemical functional groups at desired locations while assembling amino acids into a peptide chain, but synthesizing longer peptides and proteins has been considered an arduous task because of the exponential decrease in the overall yield as the number of residues increases. To acquire larger proteins, it is generally more efficient to divide the whole polypeptide chain into several fragments, then synthesize and couple them sequentially or to ligate synthesized peptides to recombinant protein segments. Therefore, for the past two decades, the development of protein synthesis methods has been focused on how to perform the selective ligation of unprotected peptides in aqueous phase in the presence of the full diversity of reactive functionalities within peptide side chains [5]. In this context, following the principle of tandem chemoselective capture and intramolecular rearrangement, native chemical ligation (NCL) has been a transformative advance that has revolutionized protein chemistry through its application in the synthesis of thousands of proteins to date [6,7]. However, the need for a relatively rare cysteine residue has limited its utility for many synthetic targets. To extend the scope of protein synthesis, considerable efforts have been directed toward incorporating desulfurization chemistry [8] or other ligation strategies such as ketoacid-hydroxylamine ligation (KAHA) [9], serine/threonine ligation (STL) [10] and diselenide selenoester ligation (DSL) [11], as well as automated fast-flow technology [12] for long peptide synthesis. In addition to chemical methods, biocatalytic strategies have received increasing attention as they exhibit inherent properties such as excellent regio- and chemoselectivity. Alongside split intein tools [13,14], genomic mining and protein engineering have led to the discovery and refinement of sortase-A [15], butelase-1 [16] and other transpeptidases with similar mechanisms. These peptide ligation strategies have given rise to a flourishing research field of protein synthesis and functionalization. Nevertheless, possible retrosynthetic disconnections are limited (Fig. 1a), and demanding challenges remain in attempts to access many biomacromolecules of interest.

To release ligation site restrictions, subtilisin-derived ligase-catalyzed peptide coupling is deemed the most promising solution to date [17] because this class of enzymes enables traceless amide bond formation with broad sequence compatibility (Fig. 1b). In the 1990s, Wells and coworkers created an enzyme called subtiligase by introducing S221C and P225A mutations into subtilisin BPN’. Subtiligase was mechanistically altered to favor aminolysis over hydrolysis, laying the foundation for development of this ligation technology [18]. Recently, we developed another exceptionally robust cation-independent subtilisin-derived ligase, termed peptiligase [19], to make this approach industrially viable. Peptiligase has remarkable catalytic efficiency and offers significantly high average ligation yields (up to 98% in <1 hour). Further engineering efforts have resulted in a series of peptiligase variants with either broad specificity that would maximize utility, or tight specificity that would allow selective ligation [20]. As demonstrated by the hundred-gram-scale synthesis of an active pharmaceutical ingredient (API) in industrial settings, peptiligase has proven to be applicable for practical manufacture of therapeutic peptides in a cost-efficient and environmentally sustainable manner [21].

However, the great potential of peptiligase for further widespread applications in protein chemistry was severely restricted by problems in the preparation and manipulation of reactive handles (Fig. 1c). First, the required acyl donors bearing an active ester at the C-terminus are sometimes challenging to prepare through SPPS. In addition, sequential enzymatic ligations for large synthetic targets are generally impractical because C-terminal protection of the acyl acceptor is necessary to prevent repeated condensation. More importantly, although several C-terminal functionalization strategies that rely on inteins [22] or specific sequences [23–25] have been developed, convenient activation of recombinant proteins for traceless ligation with broad sequence compatibility remains a longstanding challenge. Consequently, a peptiligase-compatible method that enables regioselective C-terminal activation in the aqueous phase using easily accessible peptides and proteins would be highly desirable to overcome the constraints on realizing extensively practicable and traceless protein synthesis and functionalization.

Nature brings forth sophisticated biomolecule systems for assembling free amino acids into proteins. In the course of evolution, the elegant collaboration of aminoacyl-tRNA synthetases (amino acid activation), tRNAs (intermediate ester formation) and ribosomes (amide-forming ligation) has allowed precise assembly of diverse L-amino acids with unmodified α-amino groups and side chains. Revisiting the fascinating principles of nature and possessing the peptiligase family for peptide ligation, we envisioned that sequence-independent assembly of native peptides might also be feasible through iterative activation and ligation processes using multiple enzymes that present both strict regioselectivity and broad substrate specificity. To address this possibility, we sought to design a multienzyme cooperative activation and ligation strategy (Fig. 1d) for traceless protein synthesis and functionalization, and we now present this platform, termed PALME (protein activation and ligation with multiple enzymes). By unifying acyl-shift chemistry from enzymatic and chemical protein synthesis, the PALME platform accepts SPPS products (i.e. peptide carboxylic acids, amides or hydrazides) and recombinant proteins as the input and presents proteins synthesized via sequential condensation, cyclization, protein N/C-terminal or internal functionalization as the output (Fig. 1e). Our results highlight that enzymes with diverse functions can be rationally harnessed to offer traceless protein synthesis and functionalization with remarkable flexibility in the choice of ligation sites and peptide substrates, providing unprecedentedly broad application potential.

## RESULTS AND DISCUSSION

### Establishing compatibility between peptide-activating and peptide-coupling enzymes

At the outset of our studies, we searched for a broadly applicable enzyme for C-terminal peptide esterification, to provide accessible reactive handles for peptiligase. Accordingly, we explored the peptide amidase (PAM) from *Stenotrophomonas maltophilia*, which allows sequence-independent C-terminal peptide modification with absolute regioselectivity [26]. Using computational redesign, we significantly improved the robustness and synthetic utility of PAM [27,28]. However, after exhausting different protein engineering strategies, our surveys for a mutant that catalyzes direct esterification reactions in aqueous solution were unfruitful, leading to requirement for a bridge to join the two biocatalysts. Thus, we began to consider hydrazide chemistry, which was implemented by the Liu group and has been one of the most widely used extensions to NCL [29]. In this method, the thioester functionality of the acyl donor is initially masked in the form of a C-terminal hydrazide and is sequentially retrieved via a combination of nitrite oxidation and thiolysis. We envisioned that this strategy might be adapted for peptiligase-catalyzed ligation, although it was unclear if there is an appropriate alcohol reagent for peptide acyl shifting.

We initially explored the feasibility of using 2-hydroxyacetamide, which would afford the peptide carboxamidomethyl (Cam) ester (standard peptiligase substrate) for intermediate ester formation. The model peptide hydrazide Ac-DFSKL-N_2_H_3_ was oxidized using sodium nitrite in an acidic buffer solution at −15°C, producing a peptide azide. Subsequently, 2-hydroxyacetamide was added to form the corresponding peptide Cam ester. Finally, the acyl acceptor ALKKA-NH_2_ (1.5 equiv.) and omniligase-1 (0.003 equiv., a commercially available enzyme from the peptiligase family [30]) were added to the reaction mixture, and the ligation was allowed to proceed for 30 minutes at pH 8.5 and room temperature. To our delight, the desired ligation product Ac-DFSKLALKKA-NH_2_ was formed (20% yield). This preliminary result demonstrated the possibility of using peptide hydrazide in peptiligase-catalyzed peptide ligation in a one-pot approach, albeit with low ligation efficiency. A detailed analysis of the intermediates in the cascade reactions revealed that multiple side products were formed during the esterification process, most likely because of Curtius rearrangement of the peptide azide. Therefore, to obtain a high ligation yield and a clean reaction, it is crucial to rapidly convert the peptide azide into the corresponding ester with a strong nucleophile. The formed peptide ester is also expected to be stable in a weakly alkaline solution and to fit the binding sites of peptiligase. Besides, the alcohol should be a good leaving group for enzymatic S-O exchange.

**Figure 2.**
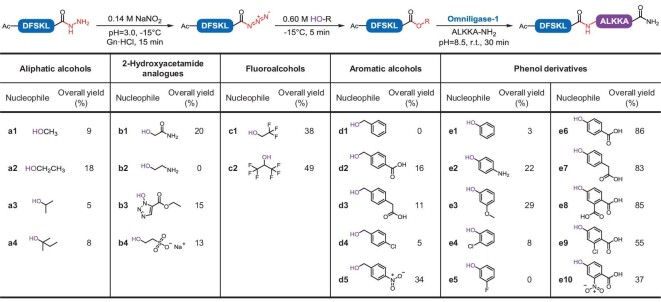
Screening of the alcohols for peptiligase-compatible esterification. The overall yield was calculated by the integration of the peak areas [19,20,31] of the ligation, hydrolysis and esterification products monitored by HPLC (220 nm). Gn·HCl: guanidine hydrochloride. More details can be found in the Supplementary data (Methods and results 4.1).

With these stipulations in mind, we investigated a panel of alcohols including aliphatic alcohols, aromatic alcohols, fluoroalcohols and 2-hydroxyacetamide analogues, for their efficiency in the model 5 + 5 reaction (Fig. 2). Most of the tested alcohols were able to mediate peptide ligation with moderate overall yields (10–50%). In particular, in phenol-mediated reactions, the peptide phenolic ester performed well in the ligation step. Accordingly, we further examined a series of phenol derivatives containing polar and electron-withdrawing substituents on the aromatic ring to improve their nucleophilicity and aqueous solubility. Gratifyingly, the use of 4-hydroxybenzoic acid (HBA, **e6**), 4-hydroxyphenylacetic acid (**e7**) and 4-hydroxyphthalic acid (**e8**) resulted in almost quantitative conversion of the peptide ester within 5 minutes. The resulting peptide esters demonstrated good chemical stability under ligation conditions, and their ligation efficiencies (>90%) were comparable to those of the standard peptide Cam esters. Further experiments showed that the racemization extents of the peptide HBA esters were <4%, and the formed D-peptide ester was not depleted after omniligase-catalyzed ligation process. Considering its overall performance and commercial availability at scale, HBA was selected for further studies.

**Figure 3.**
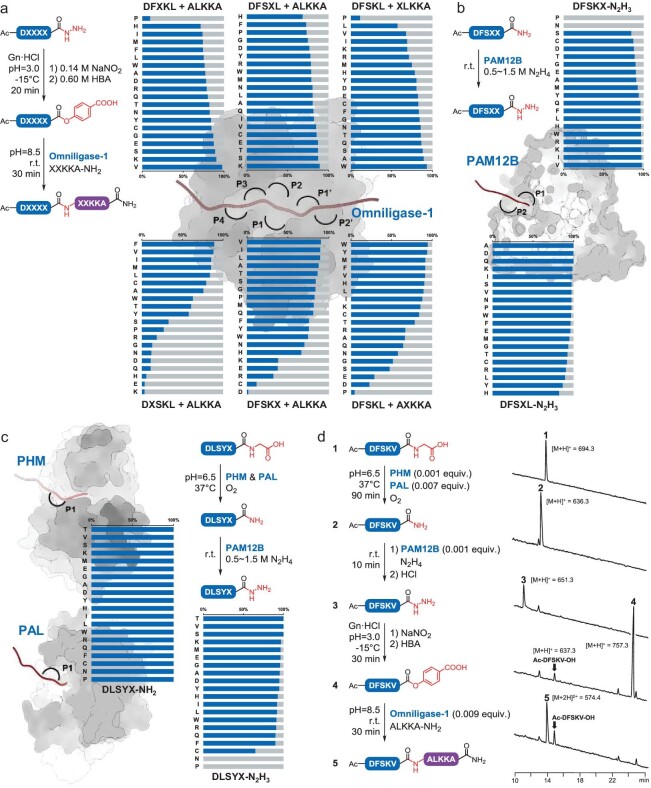
The scope and compatibility of activating and coupling modules (a) Six site-saturation peptide hydrazide and peptide amide libraries (Ac-DXSKL-N_2_H_3_, Ac-DFXKL-N_2_H_3_, Ac-DFSXL-N_2_H_3_, Ac-DFSKX-N_2_H_3_, XLKKA-NH_2_ and AXKKA-NH_2_) were utilized to investigate omniligase-1 substrate profiles. Ligation efficiency (blue band) was calculated by the integration of the peak areas [19,20,31] of the ligation and hydrolysis products monitored by HPLC (220 nm). (b) Two site-saturation peptide amide libraries (Ac-DFSXL-NH_2_ and Ac-DFSKX-NH_2_) were utilized to investigate PAM12B substrate profiles. The hydrazidation yield (blue band) was calculated by the integration of the peak areas [27] of the hydrazidation and hydrolysis products monitored by HPLC (220 nm) except for P1 = Pro or Asn. (c) Amidation and hydrazidation reactions were performed on one site-saturation peptide glycine library (DLSYXG-OH). The amidation yield (blue band) was calculated by integration of the peak areas of the amidation products and residual substrates monitored by HPLC (220 nm). The hydrazidation yield was determined as described above. (d) One-pot activation and ligation of Ac-DFSKV-G and ALKKA-NH_2_. Analytical HPLC traces (220 nm) of the reaction mixture (top to bottom): substrate, 90 minutes after the addition of PHM and PAL, 10 minutes after the addition of PAM12B, before the addition of omniligase-1, 30 minutes after the addition of omniligase-1. More details can be found in the Supplementary data (Methods and results 4.2, omniligase; 4.3 to 4.5, PAM12B, PHM and PAL; and 4.6, one-pot conjugation of Ac-DFSKV-G and ALKKA-NH_2_).

The scope of enzymatic peptide hydrazide ligation was then investigated. To map the substrate profile of six binding pockets of omniligase-1 in HBA-mediated peptide hydrazide ligations, we performed an extensive series of reactions with four acyl donor and two acyl acceptor site-saturation peptide libraries under identical reaction conditions (Fig. 3a). We were pleased to observe that the desired ligation products were obtained in all tested reactions in this thorough substrate scan, and a majority of the ligations proceeded smoothly with high coupling yields (>70%) within 1 hour. Remarkably, using this activation-ligation approach, we were even able to ligate peptides that usually serve as poor substrates for omniligase-1, for example, P1 = Pro peptide, with >80% coupling efficiency. These results indicated that the broad sequence compatibility of omniligase-1 was well maintained and even partially improved in this newly devised HBA-mediated ligation process. To accomplish the small portion of less efficient ligations, the reaction conditions can be specifically optimized by adopting approaches such as raising the acyl acceptor peptide concentration or using peptiligase variants with different substrate profiles. In addition, a group of preliminary experiments on the short peptides (e.g. a couple of pentapeptides) could provide valuable reference for selecting efficient ligation sites when formulating the synthetic scheme of a bulky target.

Having demonstrated the utilization of peptide hydrazides in peptiligase-catalyzed ligation, we continued to investigate whether the peptide-modifying enzyme PAM is feasible for converting the most basic SPPS products (peptide amides/carboxylic acids) into the corresponding peptide hydrazides, using computationally redesigned PAM12B [27]. Preliminary experiments showed that hydrazidation of the model peptide Ac-DFSKL-NH_2_ proceeded smoothly in aqueous solution at room temperature through kinetic control. In the presence of 0.5 M hydrazine and 0.00001 equiv. PAM12B, the conversion of the peptide amide was complete after 45 minutes, giving a hydrazidation product with 96% efficiency. Based on the initial success, we next evaluated the substrate sequence preference of the two terminal residue binding pockets of PAM12B by performing hydrazidation reactions with two site-saturation peptide amide libraries. In most reactions, hydrazidation products (except for P1 = Pro or Asn peptides) could be obtained in >90% yield in 1 hour (Fig. 3b), demonstrating the desired versatility of the peptide-activating enzyme.

However, the PAM12B-catalyzed modification of peptide carboxylic acids is practical only in organic environments (H_2_O < 10%) because of a thermodynamic barrier that restricts direct activation of recombinant proteins. To overcome this severe limitation, we sought to recruit an additional biocatalytic module for the selective functionalization of the peptide or protein carboxyl terminus. In the animal kingdom, many secreted peptides are processed by a peptidyl-glycine oxidation system [32]. During transformation, peptidyl-glycine hydroxylating monooxygenase (PHM) catalyzes the stereospecific hydroxylation of the α-carbon of the terminal glycine with oxygen and ascorbate, and one molecule of glyoxylate is sequentially removed by peptidyl-α-hydroxyglycine amidating lyase (PAL) to form the des-glycine peptide amide, which is the ideal substrate for PAM. With the expectation that the activating modules might work cooperatively, we prepared PHM from *Rattus norvegicus* and PAL from *Exiguobacterium* sp. by recombinant expression. Initially, we tested the activity of PHM and PAL with the model peptide DLSYLG-OH under denaturing conditions as the ligation process (2 M guanidine); however, neither hydroxylated product nor des-glycine peptide amide was detected. Then the amidation reaction was performed in the absence of guanidine, and the peptide DLSYLG-OH (1 mM) was converted to the corresponding peptide amide in 15 minutes, indicating that PHM and PAL exhibit high activity under nondenaturing conditions. We next evaluated the substrate spectrum of PHM and PAL, and all 20 model peptides DLSYXG-OH were quantitatively converted to the corresponding peptide amides, which could be swiftly (<30 minutes) and efficiently (>90% yield) hydrazinolyzed by PAM12B in a one-pot reaction as expected (Fig. 3c).

Having acquired all activating modules for supplying appropriate substrates for the coupling module, we finally tested the complete reaction route utilizing all catalytic modules with the model peptide Ac-DFSKVG-OH (**1**). Briefly, this native peptide carboxylic acid was successively converted to Ac-DFSKV-NH_2_ (**2**) by PHM and PAL and to Ac-DFSKV-N_2_H_3_ (**3**) by PAM12B. Then, excessive HNO_2_ was utilized to remove residual hydrazine and oxidize **3** at −15°C. Upon addition of HBA, the corresponding peptide ester (**4**) was obtained and subsequently conjugated with equivalent ALKKA-NH_2_ by omniligase-1 to produce Ac-DFSKVALKKA-NH_2_ (**5**, Fig. 3d). The whole process was conducted in one pot in 3 hours with only trace amounts of enzymes, exhibiting excellent catalytic efficiency, chemoselectivity and regioselectivity in the presence of a multitude of side-chain reactive functionalities. These results demonstrated that all catalytic modules exhibited a broad substrate spectrum and functioned well in series and the PALME platform was ready for further investigation.

### Broad application scope of the PALME platform for protein synthesis and functionalization

**Figure 4.**
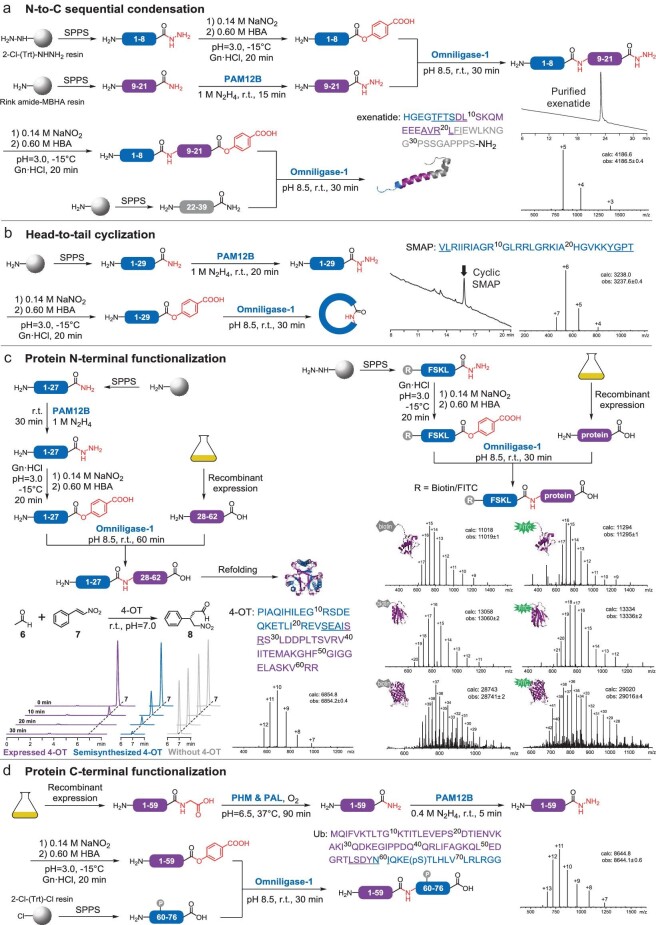
Broad applications of the PALME platform. (a) Total synthesis of exenatide (PDB: 1JRJ) by N-to-C sequential peptide condensation. (b) Cyclization of SMAP. (c) (left) Semisynthesis of 4-OT monomer. The cartoon demonstrates the structure of catalytically active 4-OT hexamer (PDB: 4X19, the A33D mutant was introduced to raise Michael-type addition activity). The activity of semisynthesized and recombinant 4-OT was determined according to the consumption of (*E*)-2-nitroethenylbenzene (7), which was monitored by HPLC (320 nm). (right) N-terminal modification of the ubiquitin-like modifier FAT10 (PDB: 6GF2), the rationally designed immunogen C4S3 (PDB: 6CBU) and EGFP (PDB: 2Y0G) with biotin/FITC-modified FSKL-N_2_H_3_. (d) Semisynthesis of Ser65-phosphorylated ubiquitin. The protein structures were constructed with PyMOL (Version 1.7 Schrödinger, LLC.). More details can be found in the Supplementary data (Methods and results 4.7, exenatide; 4.8, SMAP; 4.9 to 4.10, 4-OT; 4.11, FAT10, C4S3 and EGFP; and 4.12, Ub).

We next examined the PALME’s utility for practical applications. Considering synthetic availability and cost, we were poised to synthesize short peptide hydrazides directly via SPPS and to prepare peptide hydrazides longer than 10 residues using peptide-activating enzymes on ten-milligram scale. Initially, we tested the feasibility of enzymatic peptide N-to-C sequential condensation and employed this strategy to synthesize exenatide [33], the API of the antidiabetes drugs Byetta^®^ and Bydureon^®^. We divided exenatide into three segments, which were prepared in the form of peptide hydrazide (N-part) or peptide amide (middle part and C-part). The N-terminal segment was transformed into its HBA ester and ligated with the middle segment, generating the conjugation product with a 48% isolated yield. The obtained peptide hydrazide was then activated and conjugated with the C-terminal segment to produce 3.0 mg of purified exenatide with a 63% isolated yield (Fig. 4a). Compared to multifragment condensation in the C-to-N direction [34], our strategy avoids the multiple intractable metal-mediated deprotection processes at the N-terminus, providing a more efficient protocol for chemoenzymatic total synthesis of biomacromolecules.

In addition to intermolecular conjugation, we investigated intramolecular ligation that could generate more rigid cyclic peptides than linear substrates [35]. We selected sheep myeloid antimicrobial peptide (SMAP) [36], which does not contain Cys/Ser/Asp/Asn residues for sequence-limited chemical ligation or transpeptidation in aqueous solution, as the tested object. After esterification and ligation processes, SMAP was cyclized with an 86% efficiency according to HPLC analysis (Fig. 4b), illustrating that our activation and ligation strategy is a good supplement to current peptide cyclization methodologies.

Encouraged by the success of peptide sequential condensation and cyclization, we asked whether this strategy could be applied to objects prepared by recombinant expression, hence Cys-free 4-oxalocrotonate tautomerase (4-OT) was selected as the target for semisynthesis. 4-OT is a fascinating enzyme that promiscuously catalyzes various important synthetic reactions, including Michael addition [37], aldol condensation [38] and epoxidation [39]. As 4-OT is considered to lie at the interface between organocatalysis and biocatalysis, this protein scaffold serves as an excellent template for chemical engineering to further broaden the synthetic versatility of biomacromolecules. We prepared the C-terminal part of 4-OT by recombinant expression followed by removal of the His-SUMO-tag and then attempted to conjugate it with the SPPS-synthesized N-terminal fragment. The full-length protein was obtained after HPLC purification, and the Michaelase activity of the refolded semisynthesized enzyme resembled that of recombinant 4-OT (Fig. 4c). Subsequently, we performed N-terminal functionalization of much bulkier recombinant proteins. In 10 molar equivalents of biotin/FITC-modified peptide hydrazides, the 10 kDa ubiquitin-like modifier FAT10 [40] and the 12 kDa rationally designed HIV-1 immunogen C4S3 [41] could be labelled with an efficiency of up to 92% (Fig. 4c). The modification process was also successfully applied to a 248-mer enhanced green fluorescent protein (EGFP), which implied that our strategy is highly promising for functionalization of the majority of human proteins (those with a mass of up to ∼30 kDa).

Next, we tested whether the proteins bearing post-translational modifications in the C-terminal region were accessible via the PALME platform. As one of the most widely investigated regulatory proteins [42], ubiquitin (Ub) has hitherto been a classic target of chemical protein synthesis and semisynthesis, despite the tedious desulfurization process after NCL. We divided Ser65-phosphorylated ubiquitin into two segments, and the majority of the targeted protein could be obtained via recombinant expression. Accordingly, recombinant Ub(1–59)-Gly was amidated by PHM and PAL, followed by PAM12B-mediated hydrazidation. Afterward, the purified protein hydrazide was esterified and ligated with the synthetic 17-mer phosphorylated peptide, successfully producing the full-length phosphorylated Ub (Fig. 4d). Overall, by harnessing multiple activating and coupling enzymes that present both strict regioselectivity and broad substrate specificity, we demonstrated that the designed PALME platform should be able to cover the expected full spectrum of applications.

### Semisynthesis of intractable proteins via the PALME platform

**Figure 5.**
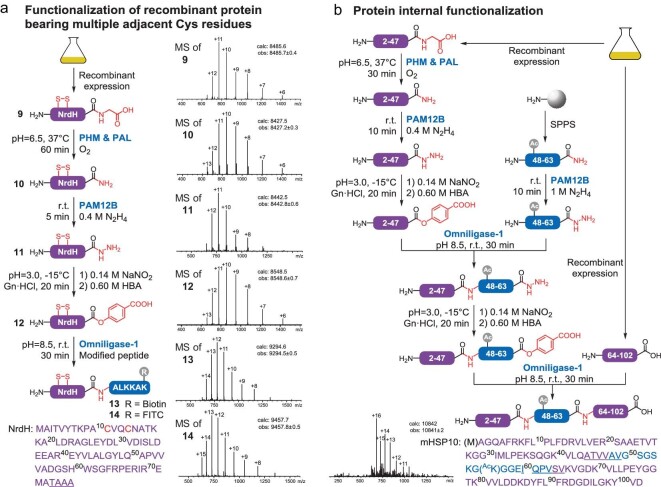
Semisynthesis of intractable proteins. (a) C-terminal modification of NrdH-redoxin. The ESI-MS results showed that the observed molecular weights of the substrate, intermediate and final products were in accord with the theoretical weight of the corresponding biomacromolecules in the oxidation state. (b) Semisynthesis of Lys56-acetylated mHSP10. Additional details can be found in the Supplementary data (Methods and results 4.13, NrdH-redoxin and 4.14, mHSP10).

Having verified the PALME platform's broad application scope, we next attempted to apply it to currently intractable targets. Recombinant proteins bearing multiple adjacent Cys residues are tricky to handle because chemical methods involving cysteine/thiols require pretreatment of native proteins to reduce the disulfide bonds [43]. When applying thiol-dependent chemical methods to synthesize these targets, native Cys residues are often mutated or protected to avoid side reactions. With the thiol-free, native Cys-independent activation and ligation strategy in hand, we were poised to activate and functionalize NrdH-redoxin, an electron donor bearing a CXXC catalytic motif at the active site that forms a disulfide bond in the oxidation state. This protein is a promising drug target as it functions cooperatively with prokaryote-specific class Ib ribonucleotide reductase and is essential for cell metabolism [44]. By utilizing PHM, PAL, PAM12B and omniligase-1 together, we converted the recombinant NrdH-Gly to the corresponding protein hydrazide and labelled it with biotin/FITC at the C-terminus (Fig. 5a). The vital disulfide bond was not disturbed throughout the process, suggesting that the PALME platform could be a suitable supplement for handling intractable multiple-Cys proteins without protection/deprotection processes.

Finally, we examined the PALME’s utility in protein semisynthesis applied to internal regions, which is one of the most in-demand methodologies in protein synthesis [3]. We chose Lys56-acetylated human mitochondrial heat shock protein 10 (mHSP10), which participates in cellular protein folding by composing a chaperonin symmetrical football complex with mitochondrial heat shock protein 60 (mHSP60) [45], as the target for demonstration. The location of the desired modification site within the protein sequence most often determines whether semisynthesis is viable. As the modified Lys56 is located in the internal region of mHSP10, a multistep ligation strategy involving the assembly of three segments was expected to be adopted. Unfortunately, no Cys or even Ala residue is available for conventional NCL protocols between Lys56 and Asp102 at C-terminus. The PALME platform's broad substrate spectrum in terms of both sequence and C-terminal functionality meant that we could design a synthetic scheme that requires the chemical synthesis of only one 16-mer peptide amide. First, we converted the synthetic peptide amide to an almost equivalent amount of the corresponding peptide hydrazide. Next, the protein hydrazide of the N-terminal region was produced from the recombinant protein glycine smoothly. Afterward, two rounds of esterification, ligation and HPLC purification were performed following a general protocol for sequential fragment condensation, producing full-length acetylated mHSP10 (Fig. 5b). Overall, the platform's modular nature could provide researchers with a flexible selection of input substrates and output functions and their combinations, which would generate plentiful retrosynthetic disconnections for disassembling hard-to-access proteins.

## CONCLUSION

In summary, we have designed and built a robust, modular and efficient multienzyme platform (PALME) for traceless total/semi protein synthesis and functionalization. The versatility of each module was evaluated via hundreds of model reactions, and we also demonstrated the utility of PALME by synthesizing a series of real-case targets on the scale of hundred micrograms to milligrams. This platform offers a comprehensive range of solutions for chemical protein synthesis because of its unique features. First, the peptide-activating modules endow the platform with an impressively broad substrate spectrum in terms of both sequence and C-terminal functionality. While experienced protein chemists can directly synthesize the desired peptide hydrazides, biological researchers have the flexible choice of using more easily accessible peptide amides (provided by peptide synthesis companies) or native peptides/proteins (obtained from in-house recombinant expression) as assembly materials. Second, by introducing new activating reagents in the coupling module, previously challenging applications of peptiligase, such as multifragment sequential ligation and recombinant protein C-terminal ligation/functionalization, are readily feasible. Thus the full potential of peptiligase technology is assuredly realized. Finally, the platform's modular nature is likely to work well with flow chemistry, which offers more efficient multistep synthesis than traditional batch methods. Taken together, the PALME platform is highly complementary to other modern techniques for chemical protein synthesis and provides viable solutions to challenges that previous strategies could not address.

While the catalysts used in this system function well in series, their performances can be further improved by directed evolution or computational engineering. PHM and PAL are expected to tolerate higher guanidine concentrations through enzyme engineering [27,46] to accept recombinant peptides with low solubility under nondenaturing conditions. In addition, the use of PAM mutants that greatly suppress hydrolysis in the acyl-shift process can considerably decrease the hydrazine concentration required in the one-pot process. Moreover, proteolytically removable sequences may be introduced at the N-termini of acyl donor peptides to prevent undesired cyclization or self-condensation. We anticipate that this study will serve as a blueprint for future development of a widely applicable protocol to access synthetic proteins, and facilitate artificial biomacromolecule design and applications.

## MATERIALS AND METHODS

Detailed materials and methods are available in the Supplementary data.

## Supplementary Material

nwab158_Supplemental_Files
