# Supplementary material for: Traceless enzymatic protein synthesis without ligation sites constraint
Source: Natl Sci Rev. 2021 Aug 24;9(5):nwab158. doi: 10.1093/nsr/nwab158 (PMC9155641; doi:10.1093/nsr/nwab158)
Supplement: nwab158_Supplemental_Files [file nwab158_supplemental_files.zip › Revised_supplementary_HPLC_traces_and_MS_figures-210811.docx]

Supplementary HPLC traces and MS figures for

**Traceless enzymatic protein synthesis**

**without ligation sites constraint**

Ruifeng Li^1,2#^, Marcel Schmidt^3#^, Tong Zhu^1,2#^, Xinyu Yang^1,2^, Jing Feng^1,2^, Yu’e Tian^1^, Yinglu Cui^1^, Timo Nuijens^3*^, Bian Wu^1*^

^1^ CAS Key Laboratory of Microbial Physiological and Metabolic Engineering, State Key Laboratory of Microbial Resources, Institute of Microbiology, Chinese Academy of Sciences, China

^2^  University of Chinese Academy of Sciences, China

^3^ Fresenius Kabi iPSUM, I&D Center EnzyPep B.V., the Netherlands

# Equal contribution.

* Email: [timo.nuijens@fresenius-kabi.com](mailto:timo.nuijens@fresenius-kabi.com) or [wub@im.ac.cn](mailto:wub@im.ac.cn)

Table of contents

[1 Alcohol screening for enzymatic ligation 1](#_Toc69988968)

[1.1 Screening of the alcohols 1](#_Toc69988969)

[1.2 Identifying the racemization of the peptide HBA esters 4](#_Toc69988970)

[2 Substrate screening of Omniligase-1 6](#_Toc69988971)

[2.1 screening of Omniligase-1 P4 substrates 6](#_Toc69988972)

[2.2 screening of Omniligase-1 P3 substrates 11](#_Toc69988973)

[2.3 screening of Omniligase-1 P2 substrates 16](#_Toc69988974)

[2.4 screening of Omniligase-1 P1 substrates 21](#_Toc69988975)

[2.5 screening of Omniligase-1 P1’ substrates 26](#_Toc69988976)

[2.6 screening of Omniligase-1 P2’ substrates 31](#_Toc69988977)

[3 Substrate screening of PAM12B 36](#_Toc69988978)

[3.1 screening of PAM12B P2 substrates 36](#_Toc69988979)

[3.2 screening of PAM12B P1 substrates 38](#_Toc69988980)

[4 Screening of PHM, PAL & PAM12B P1 substrates 40](#_Toc69988981)

# Alcohol screening for enzymatic ligation

## 1.1 Screening of the alcohols

1. **aliphatic alcohols, 2-hydroxyacetamide analogues, fluoroalcohols**


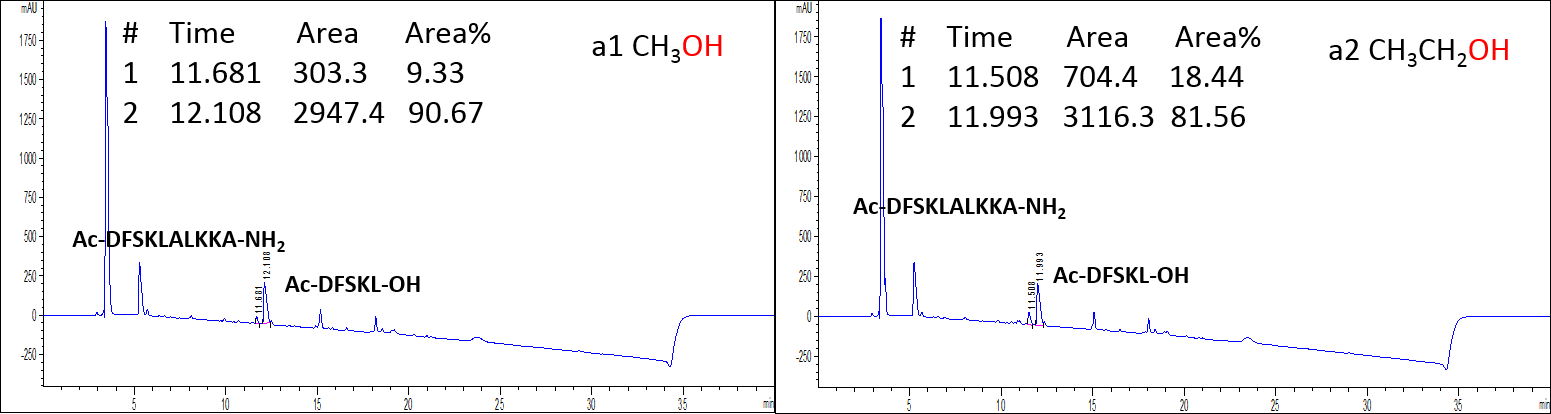

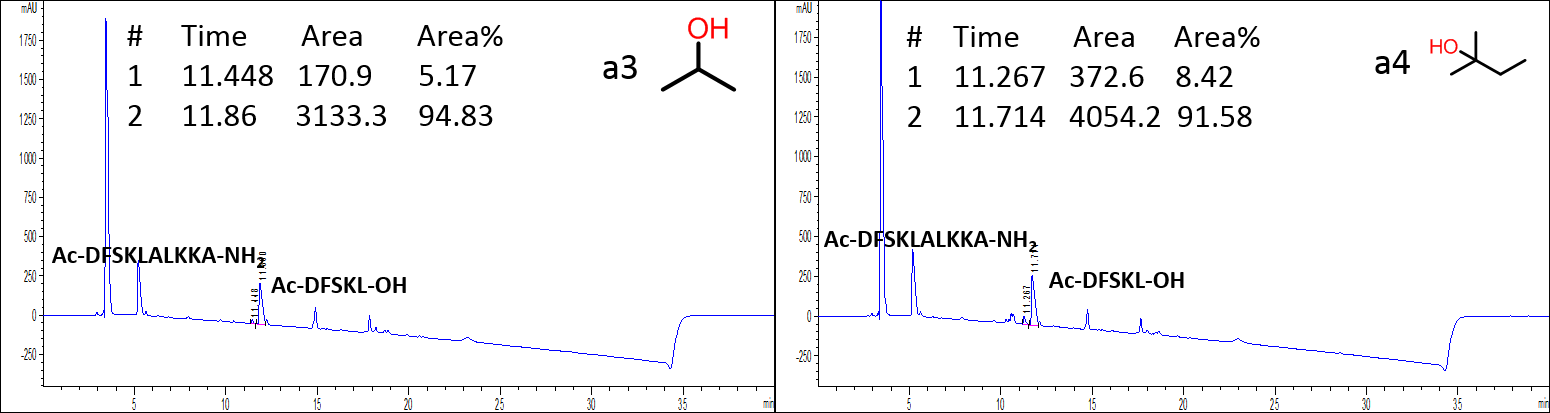

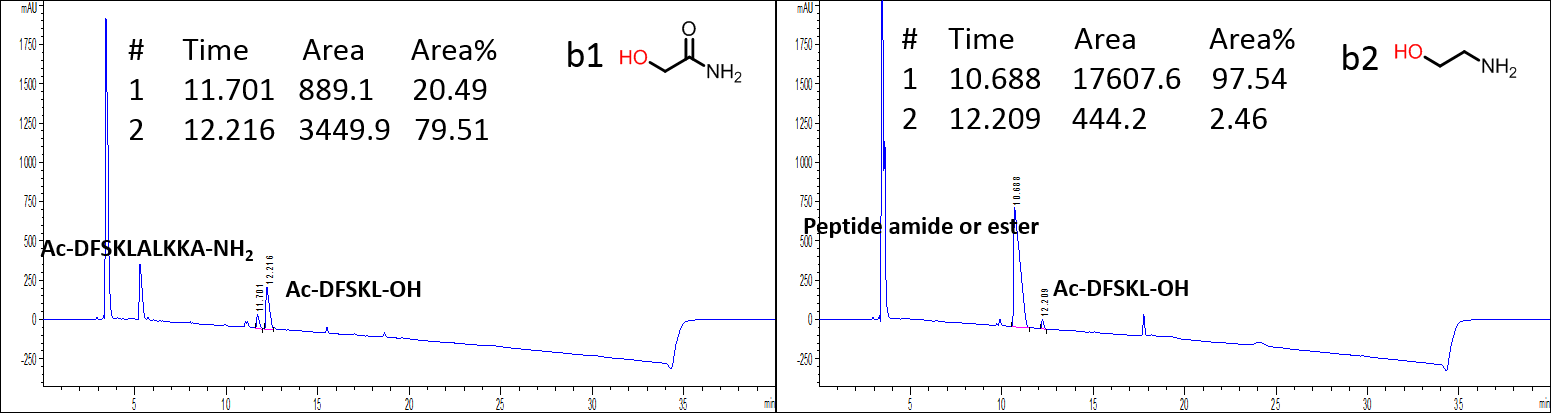

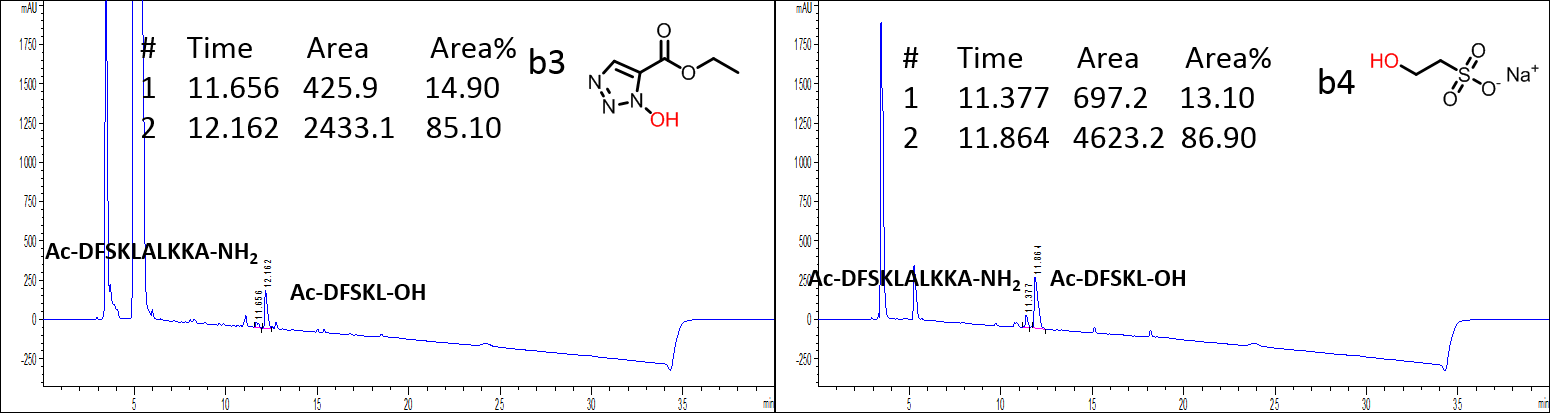

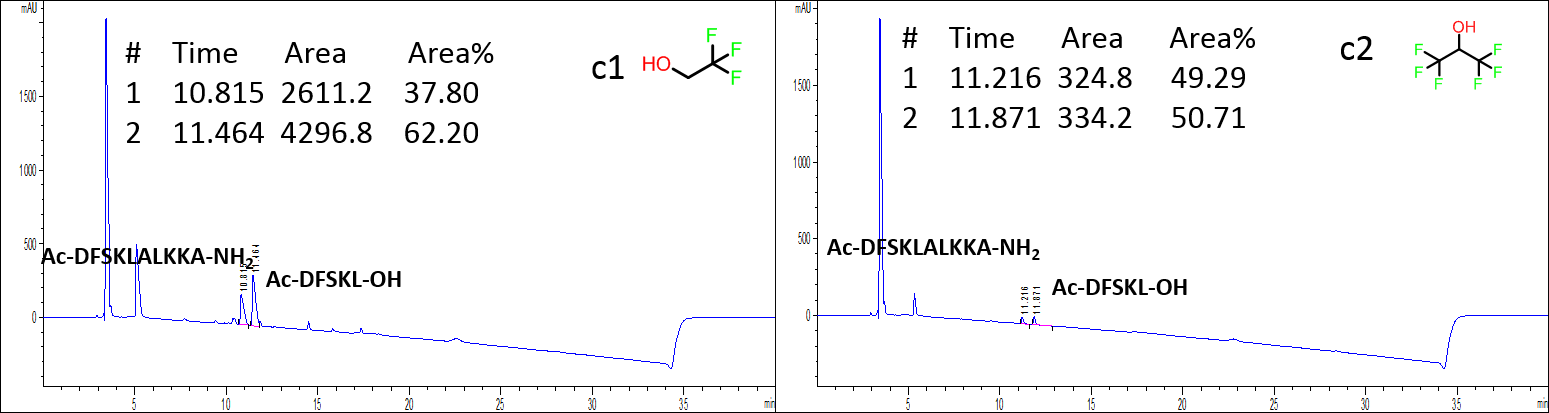


1. **aromatic alcohols**


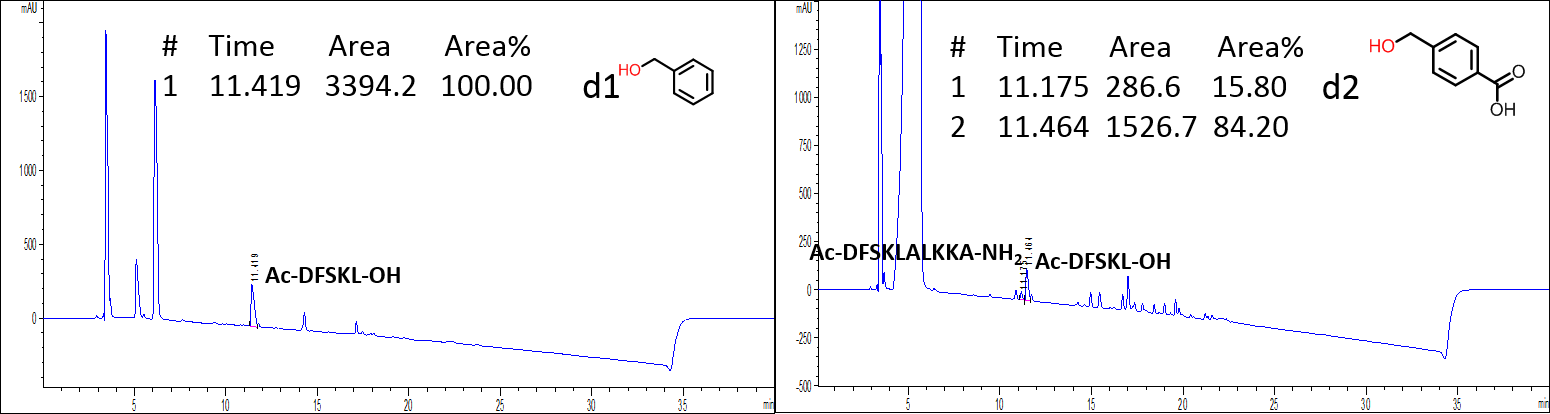

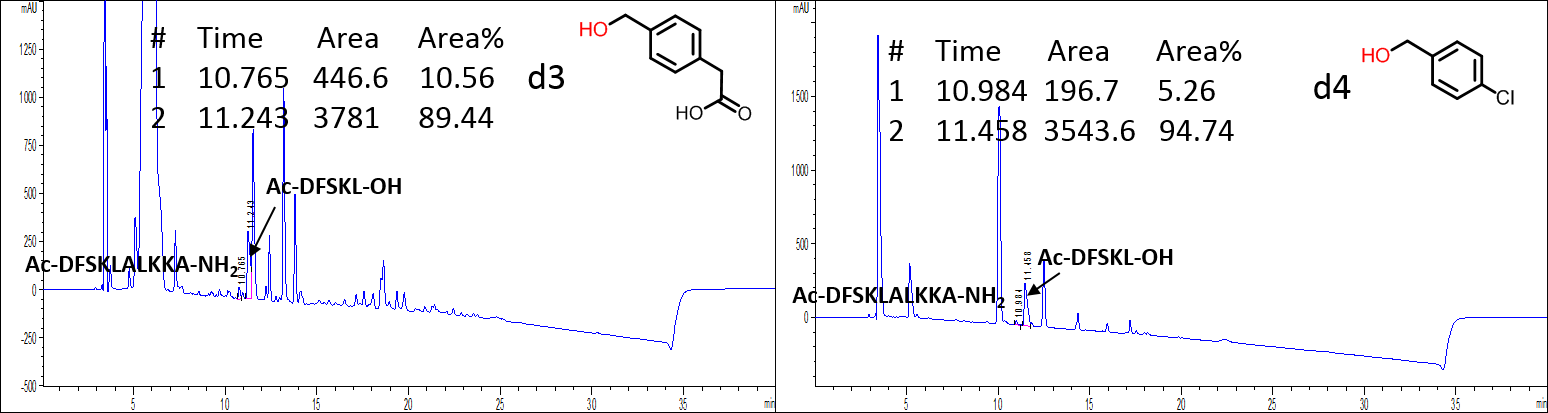

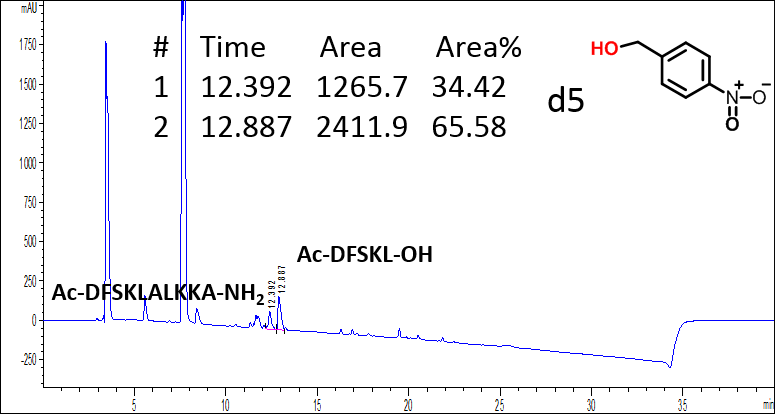


1. **phenol derivatives**


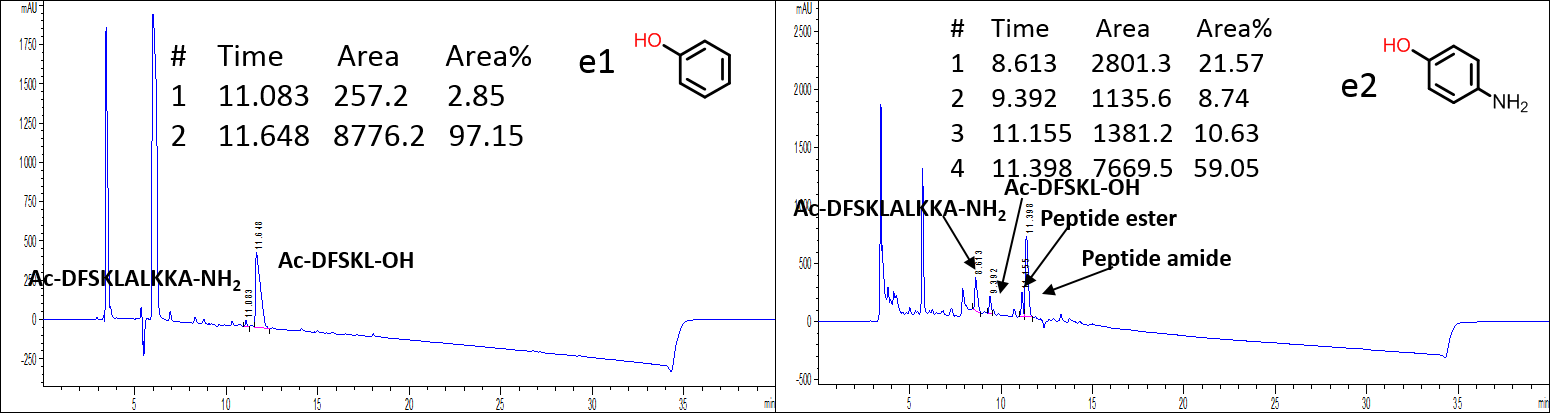

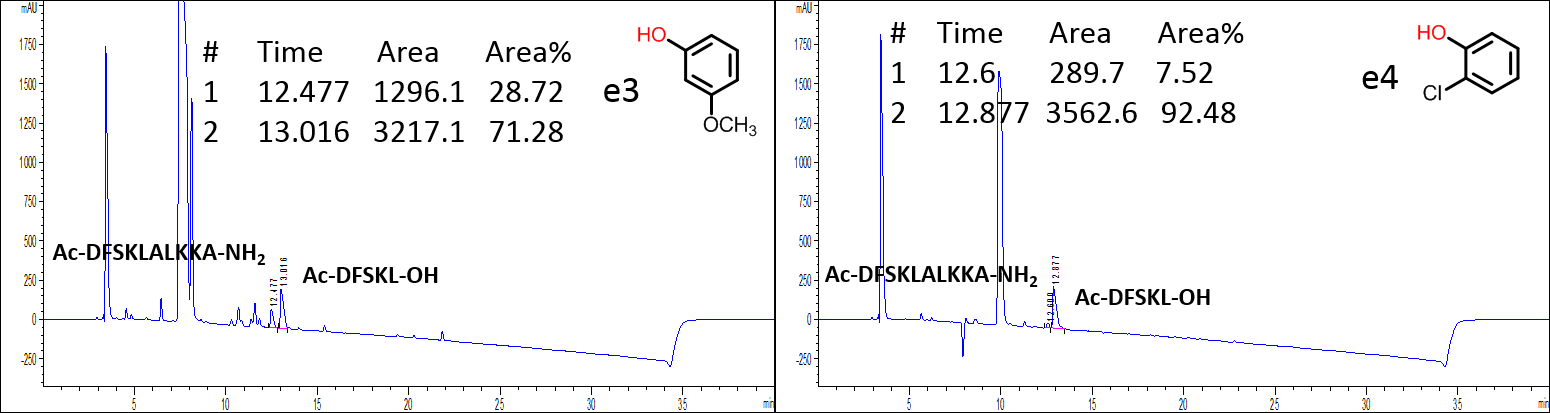

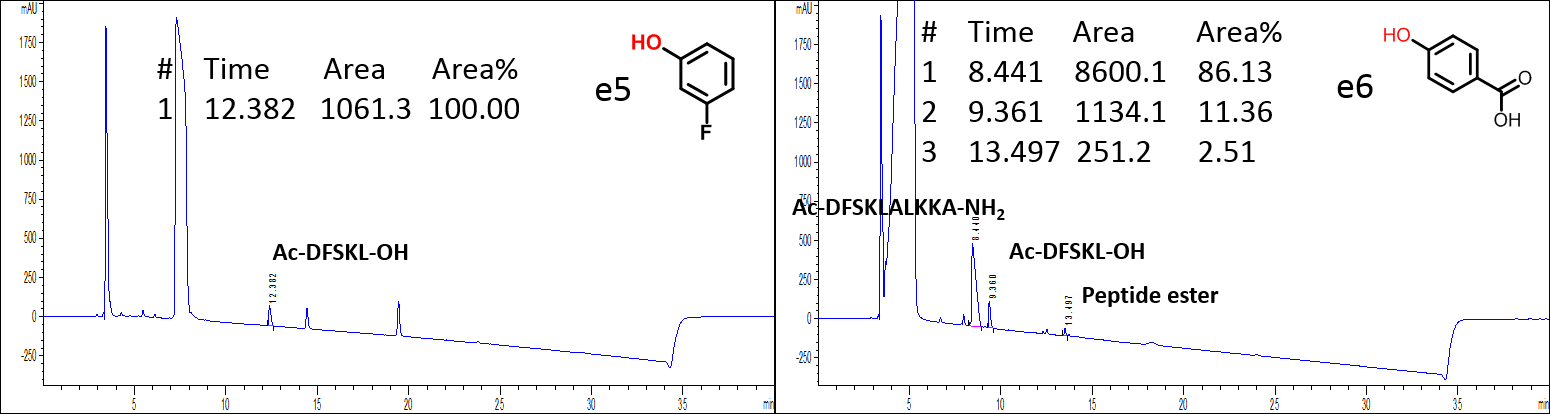

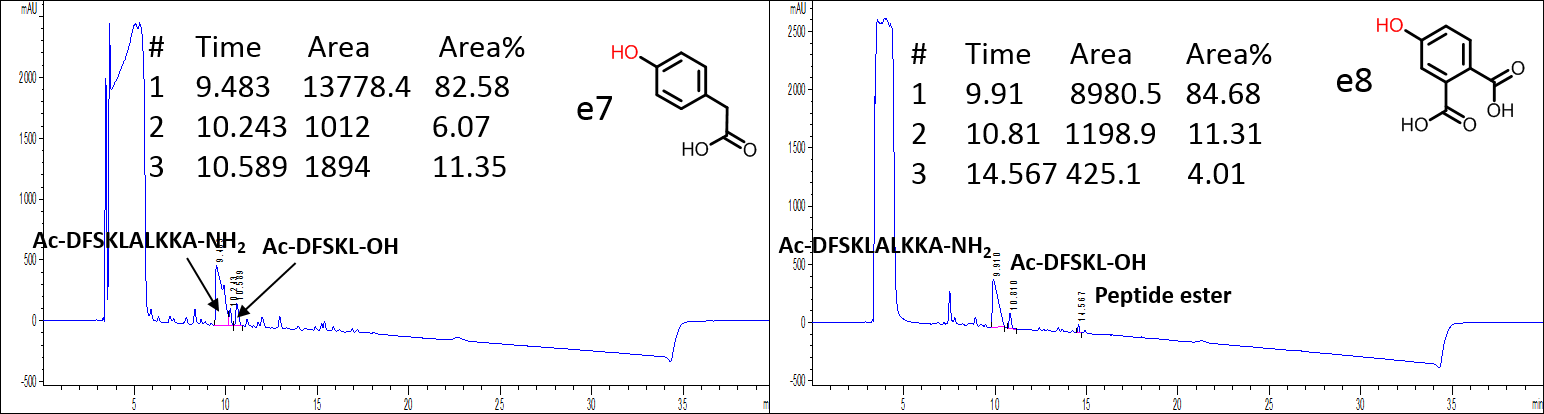

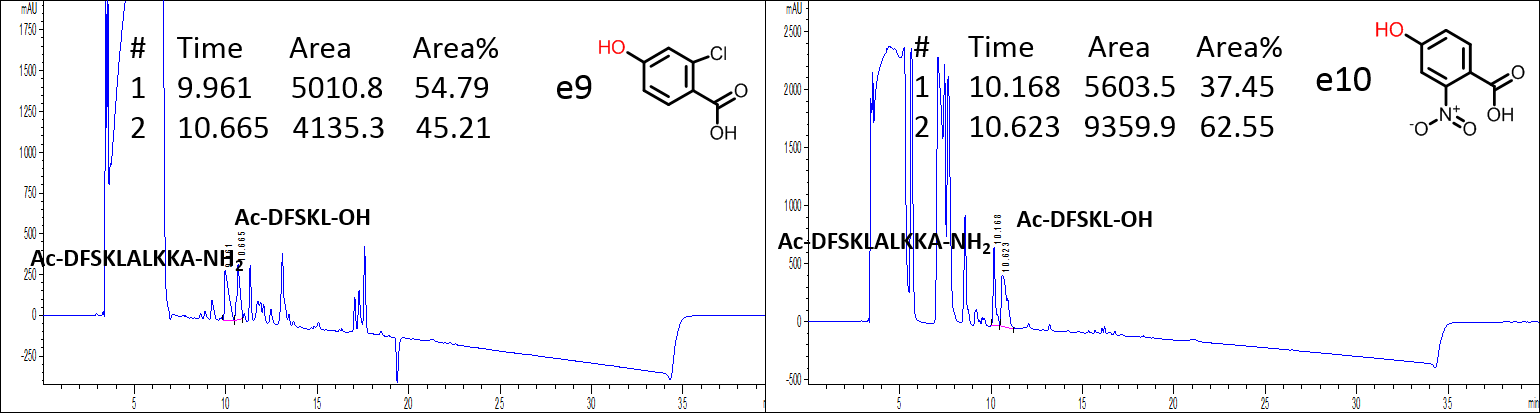


## 1.2 Identifying the racemization of the peptide HBA esters

1. **Esterification of Ac-Asp-Phe-Ser-Lys-(L)-Phe-N_2_H_3_ (top) and Ac-Asp-Phe-Ser-Lys-(D)-Phe-N_2_H_3_ (bottom)**


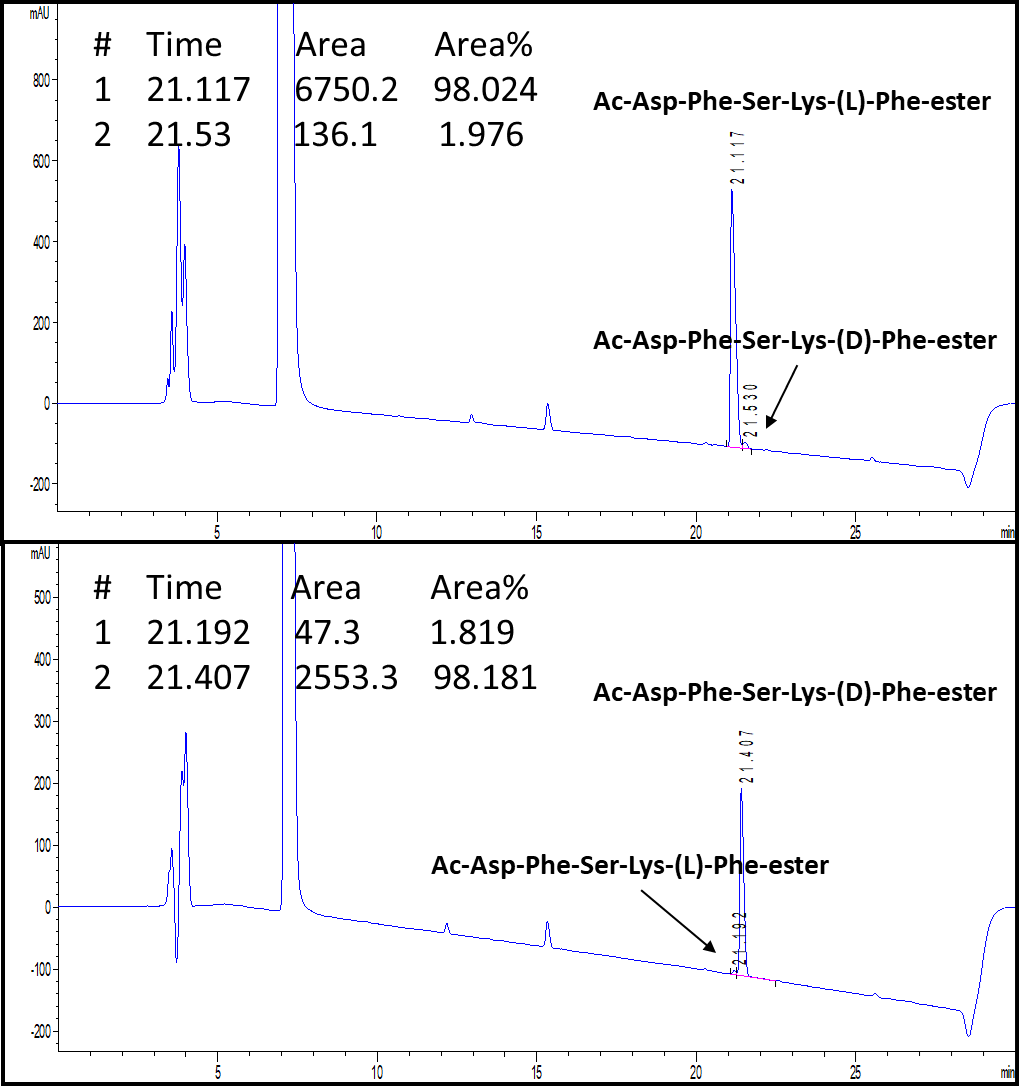


1. **Esterification of Ac-Asp-Phe-Ser-Lys-(L)-Leu-N_2_H_3_ (top) and Ac-Asp-Phe-Ser-Lys-(D)-Leu-N_2_H_3_ (bottom)**


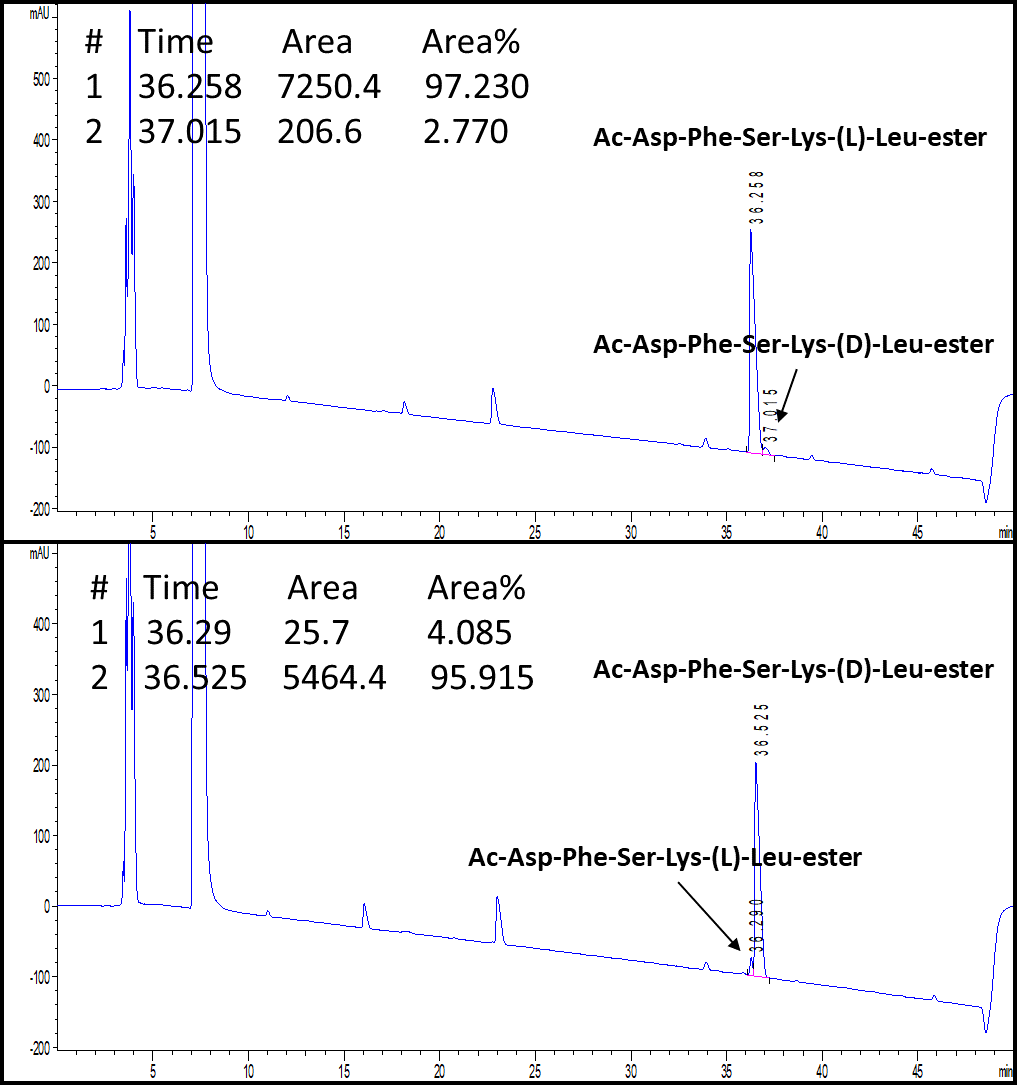


1. **Ac-Asp-Phe-Ser-Lys-(L)-Val-Ester was utilized as the substrate of Omniligase, while Ac-Asp-Phe-Ser-Lys-(D)-Val-Ester was not depleted (results of the experiments in supplementary methods and results chapter 4.6).**


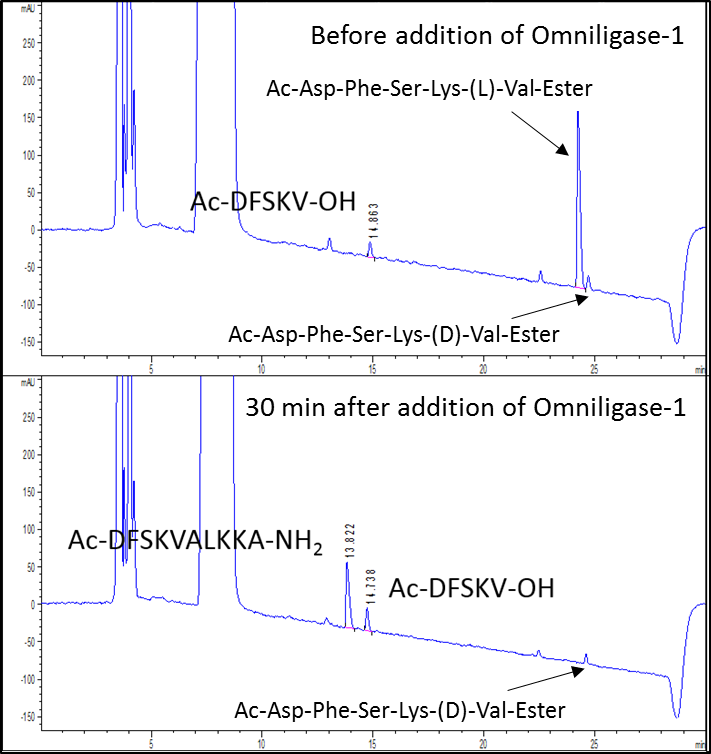


# 2 Substrate screening of Omniligase-1

## 2.1 screening of Omniligase-1 P4 substrates

1. **P4 = V, I, M, L**


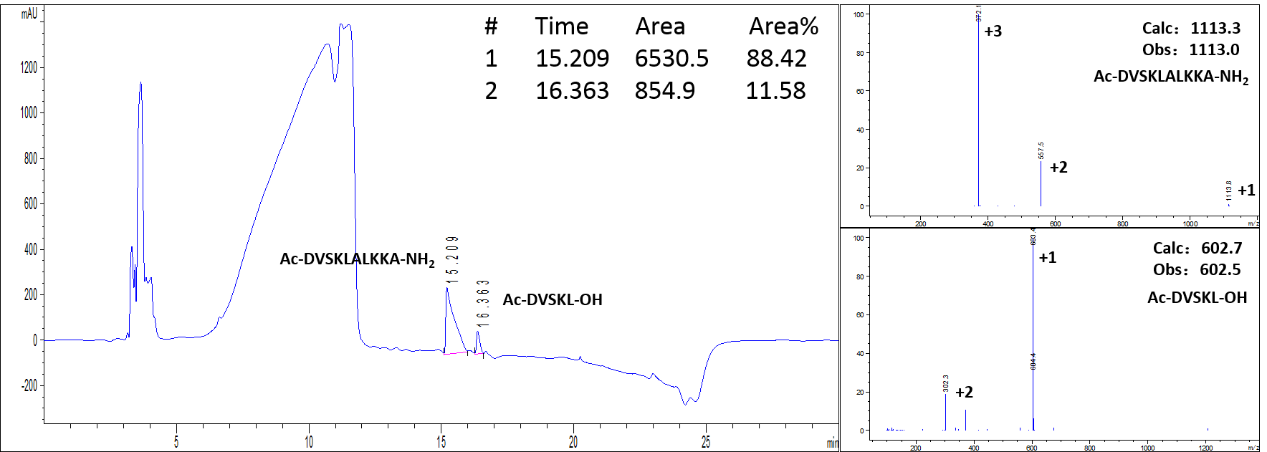

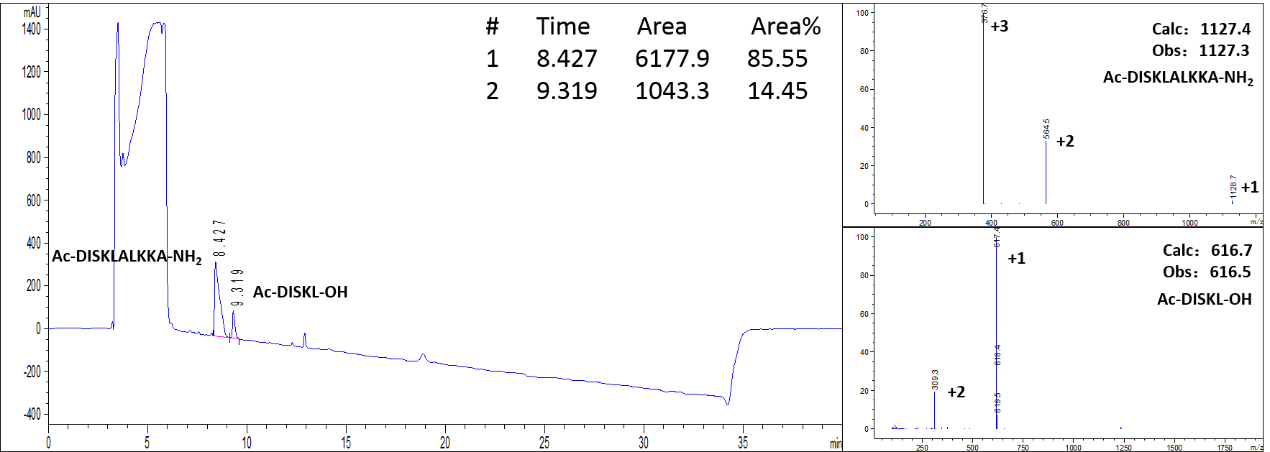

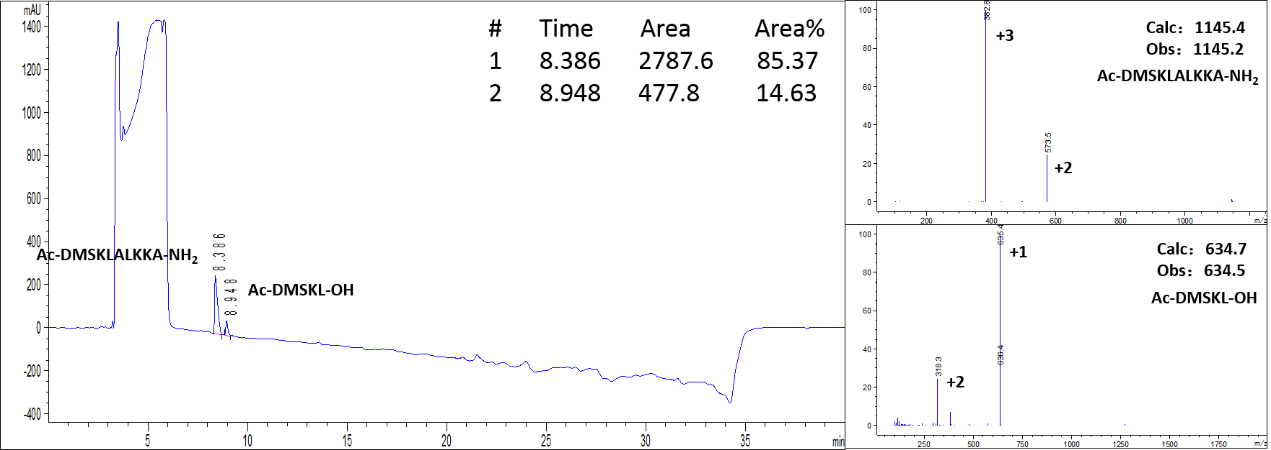

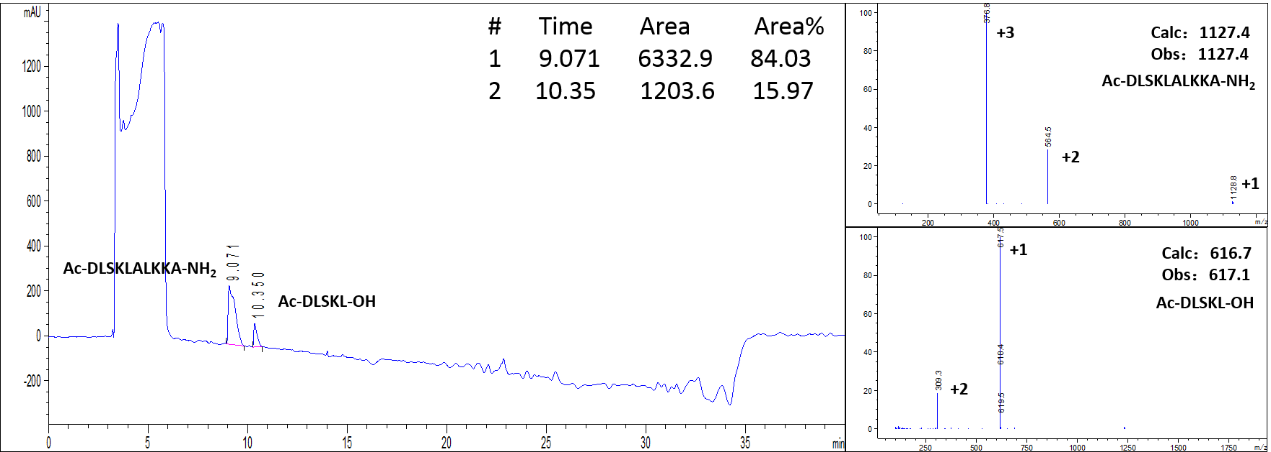


1. **P4 = C, A, W, Y**


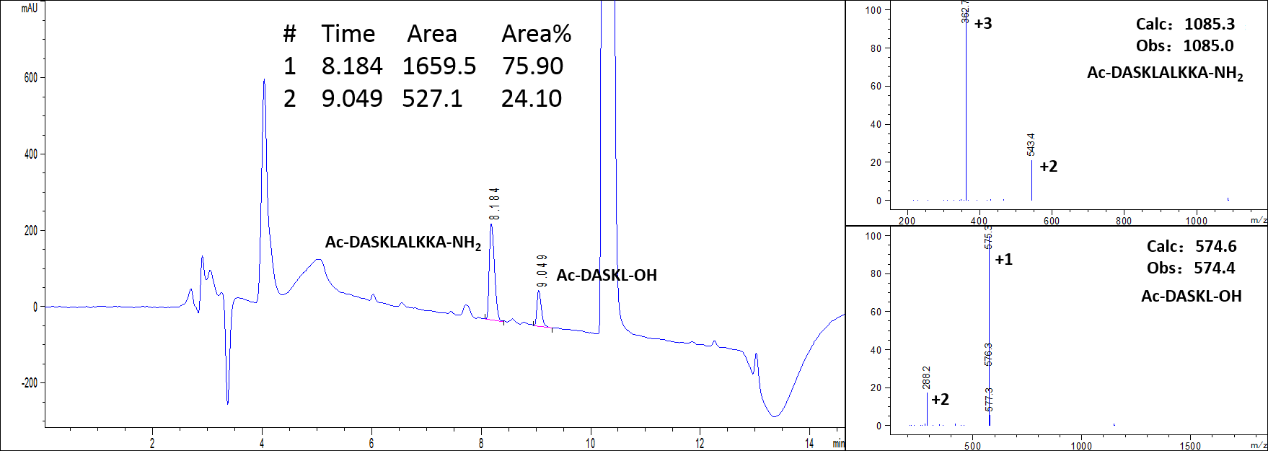

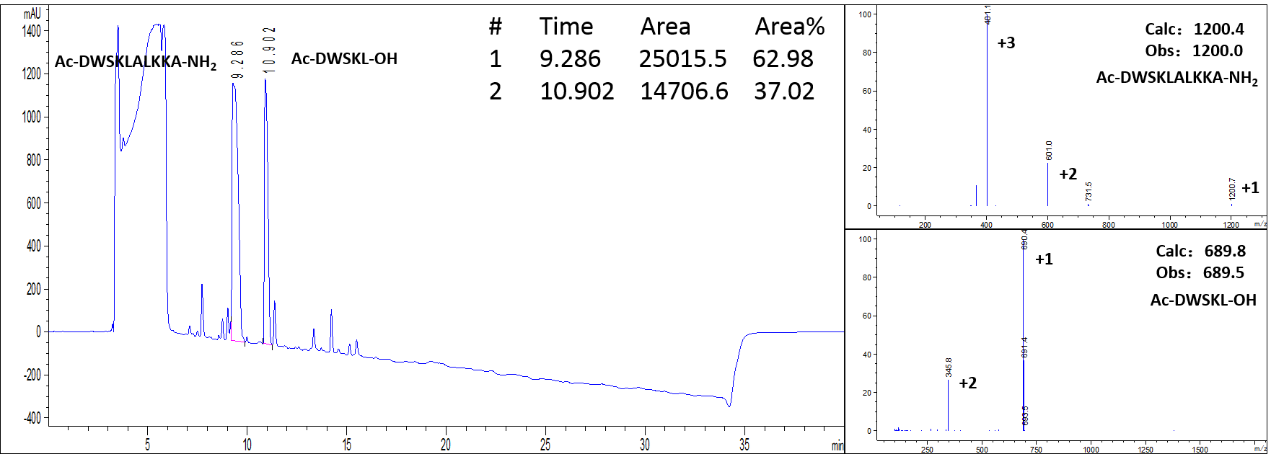

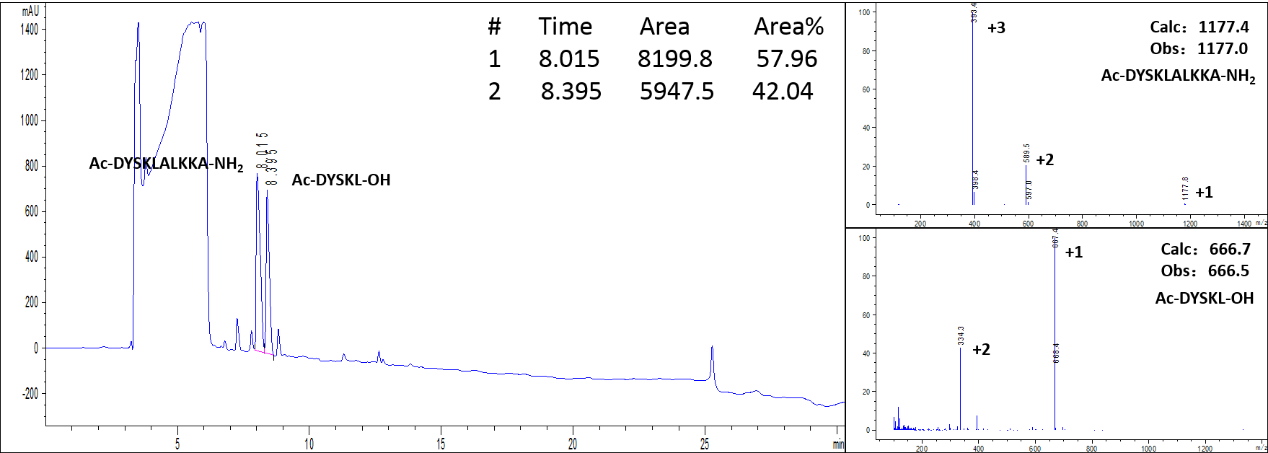

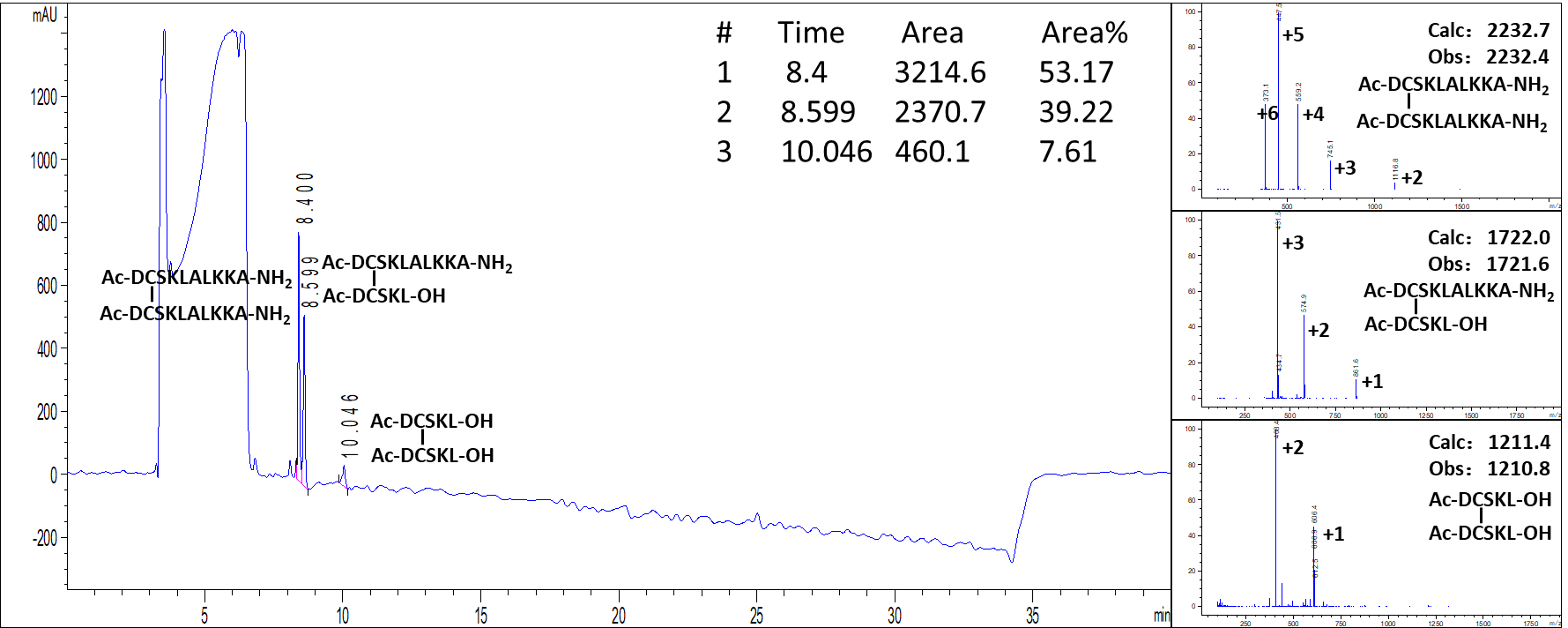


1. **P4 = S, R, Q, D**


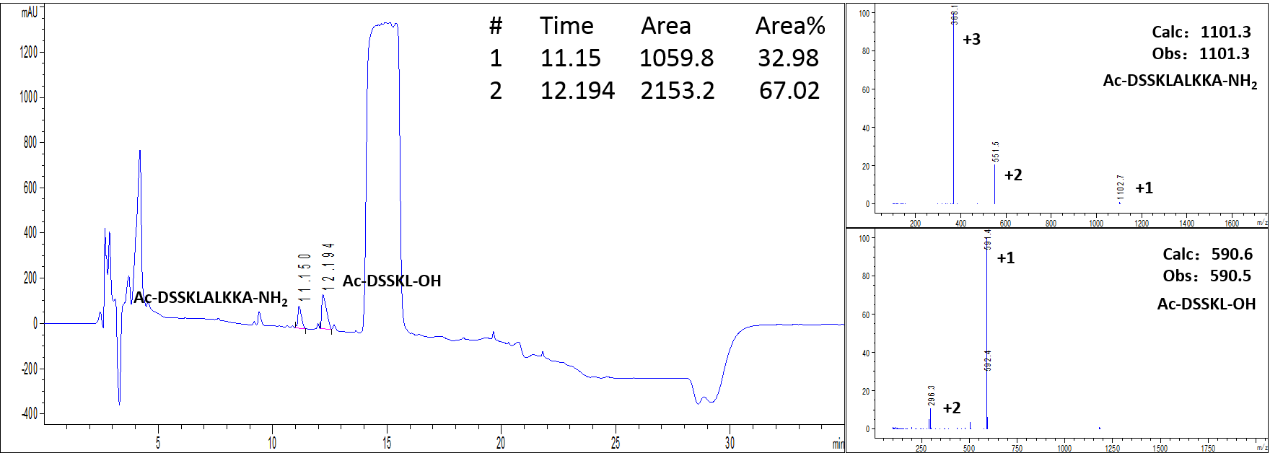

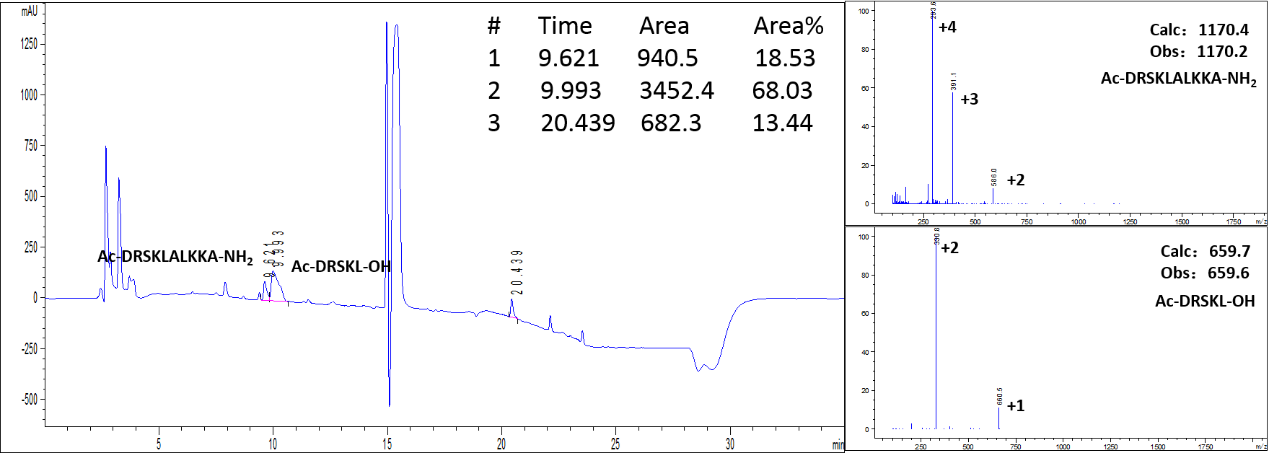

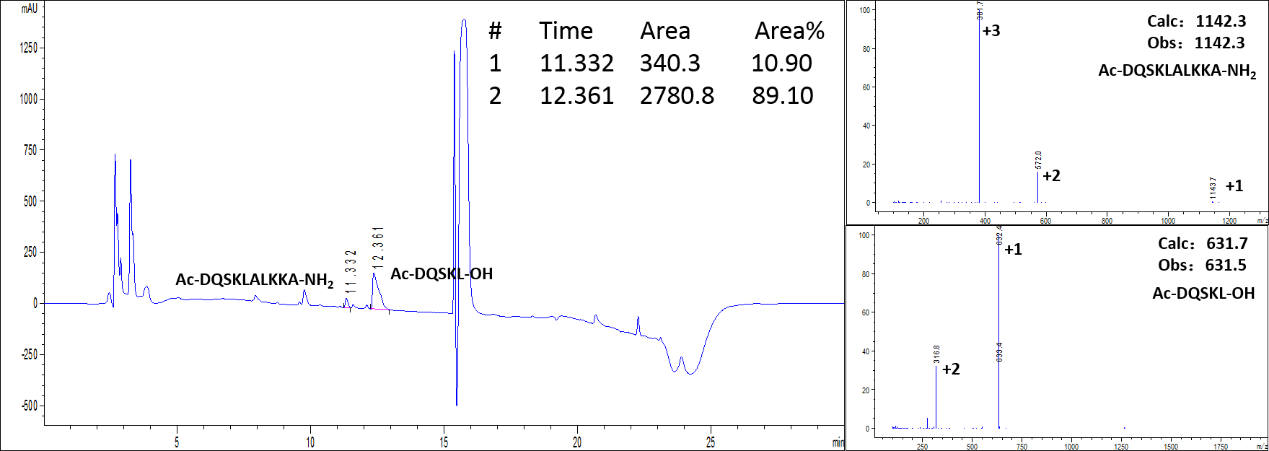

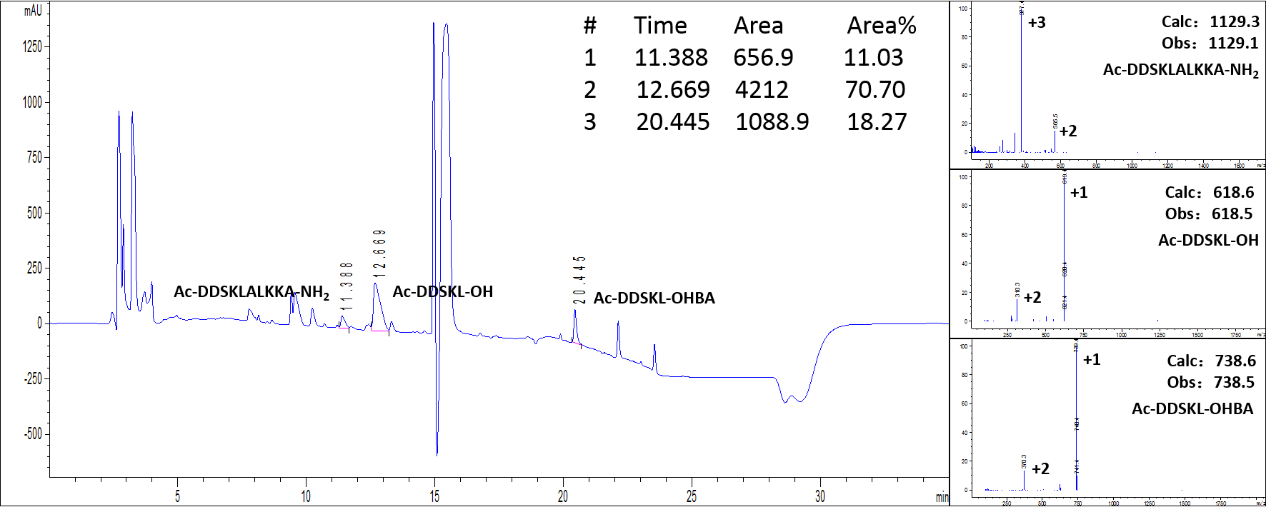


1. **P4 = N, G, H, E**


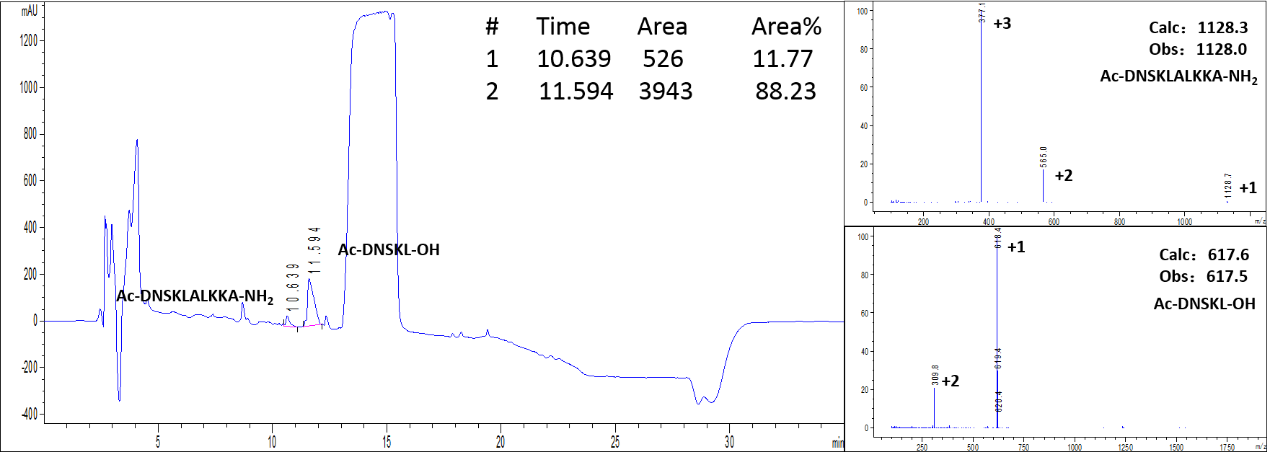

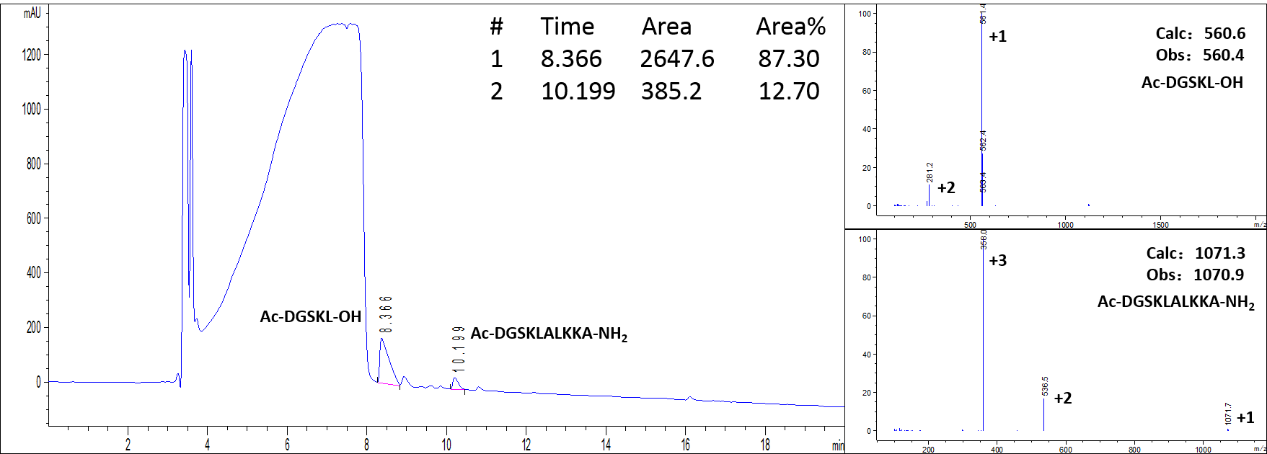

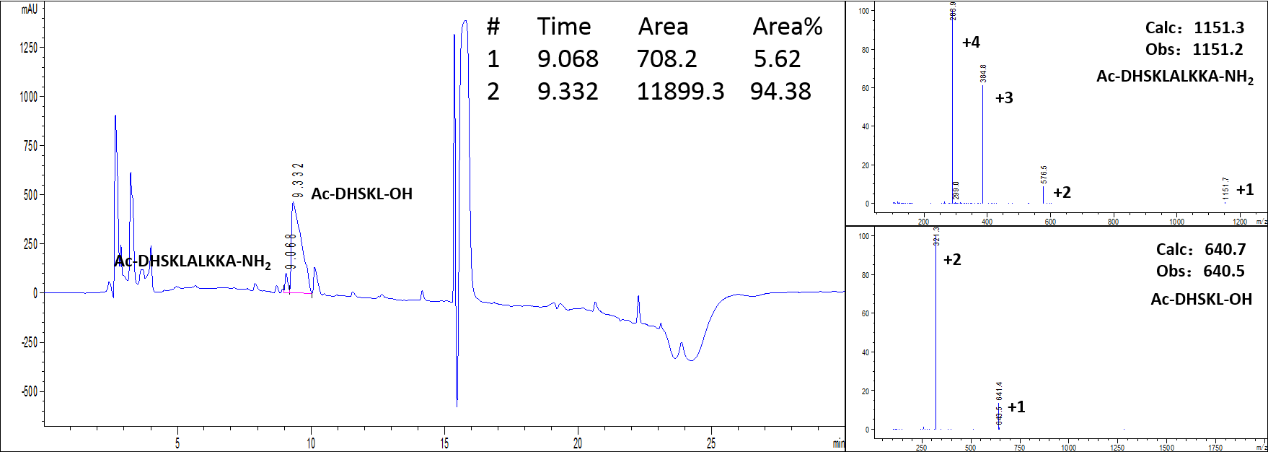

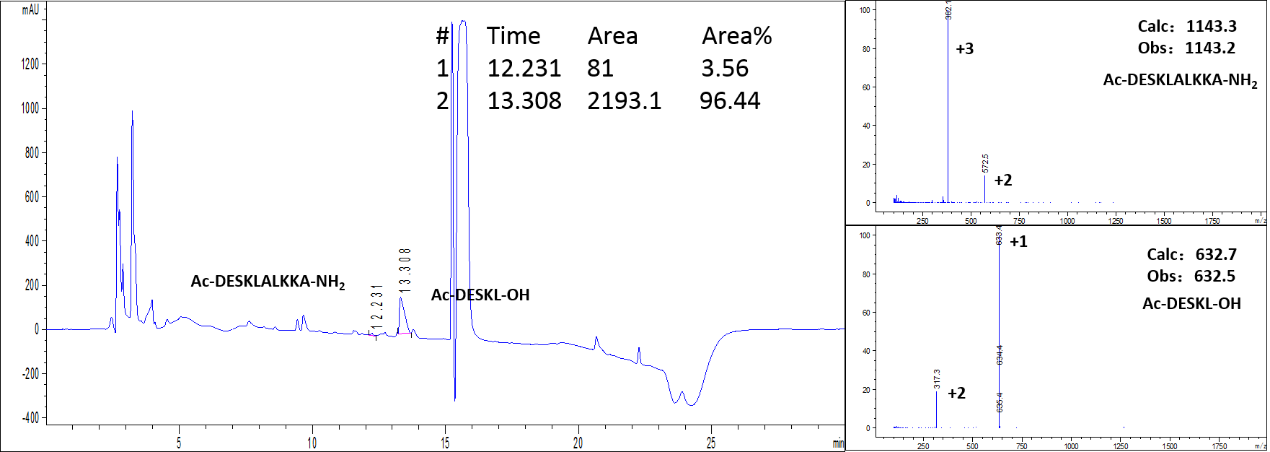


1. **P4 = K, P, T**


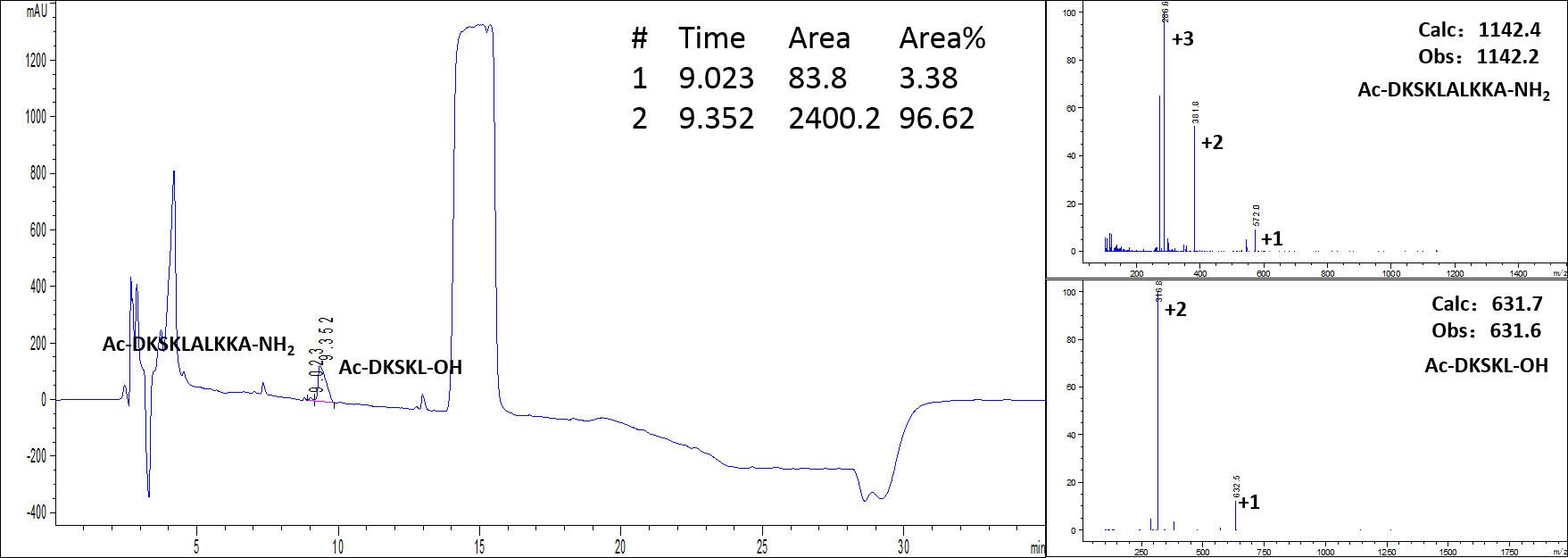

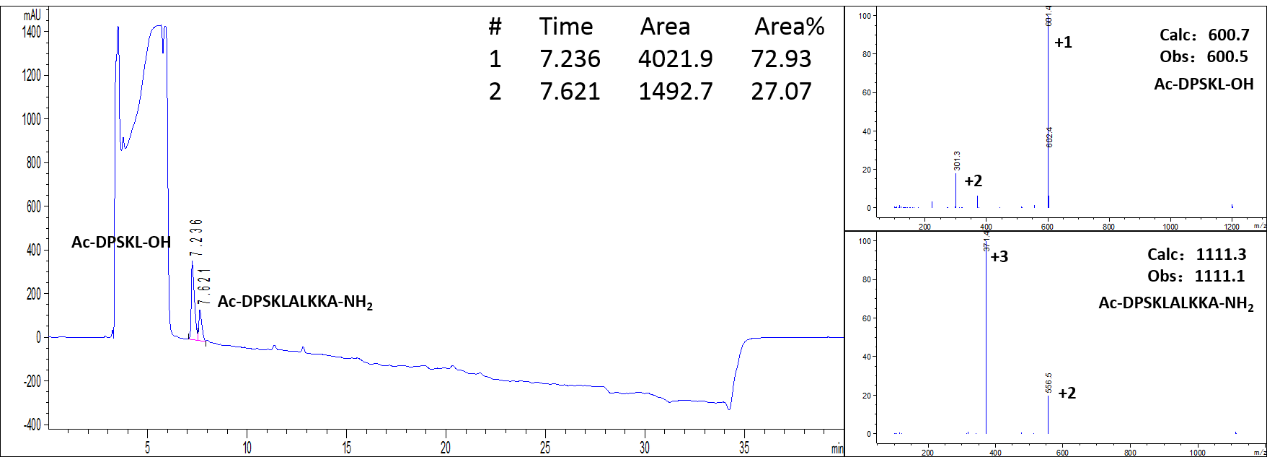

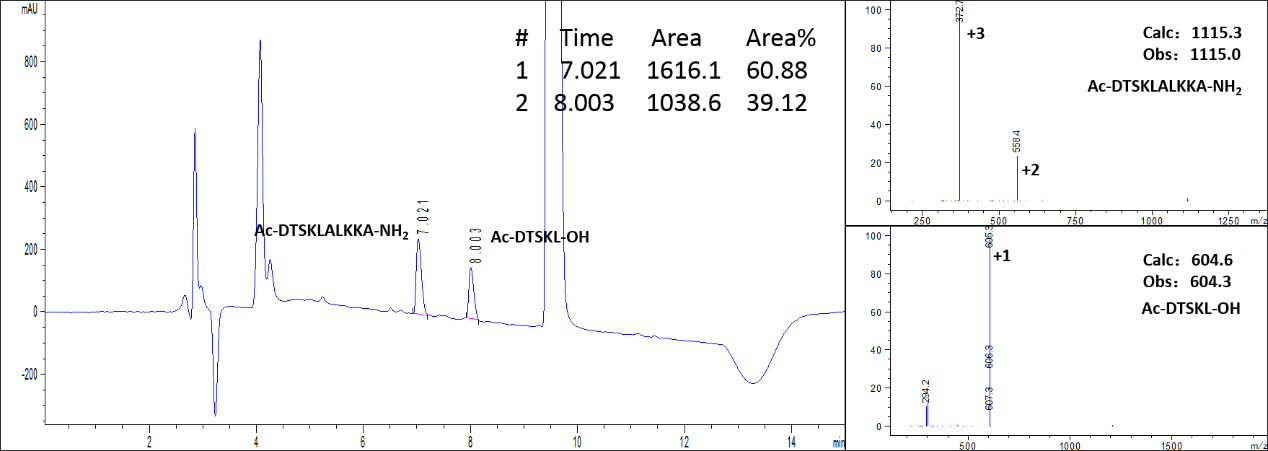


## 2.2 screening of Omniligase-1 P3 substrates

1. **P3 = F, V, I, M**


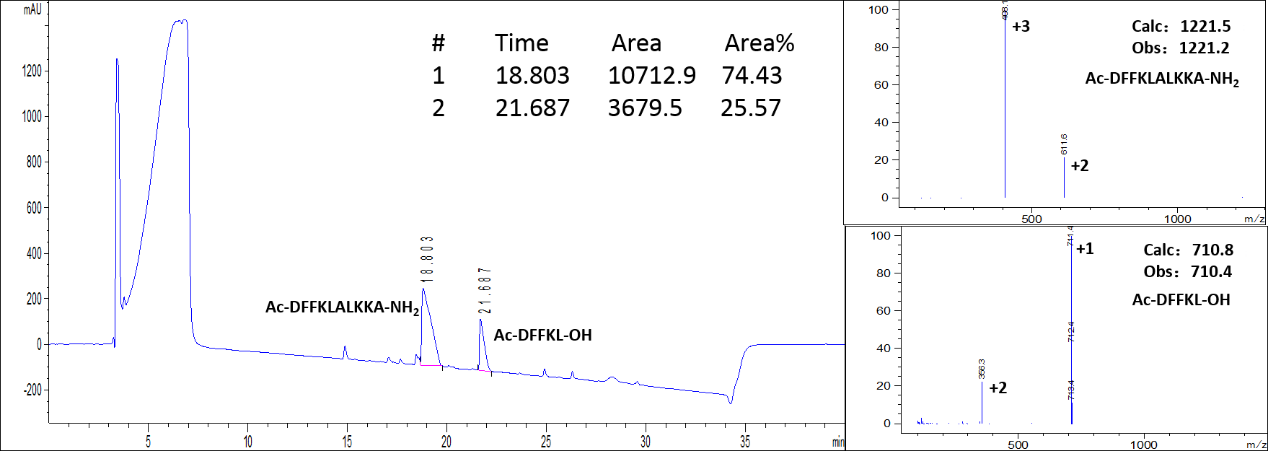

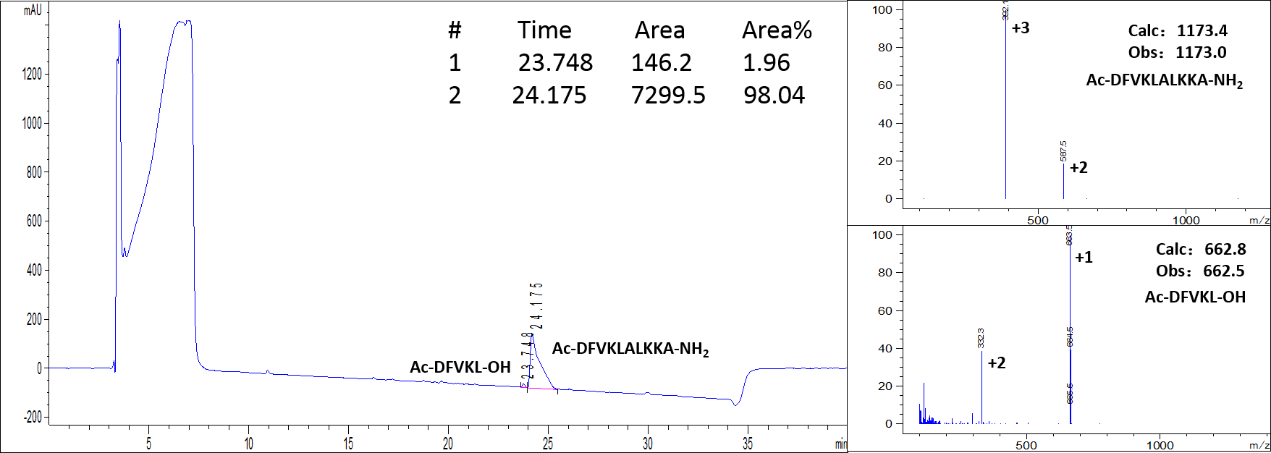

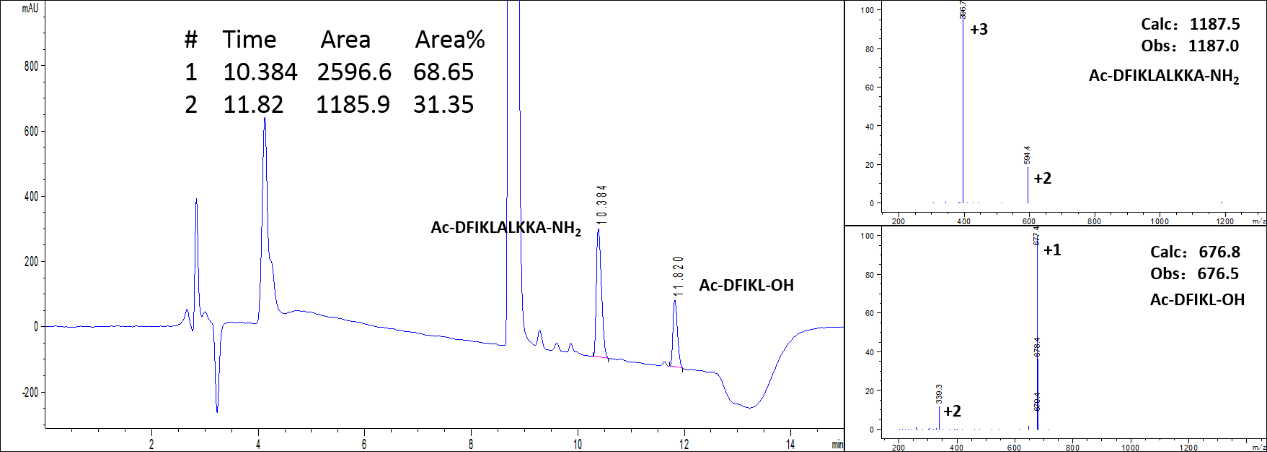

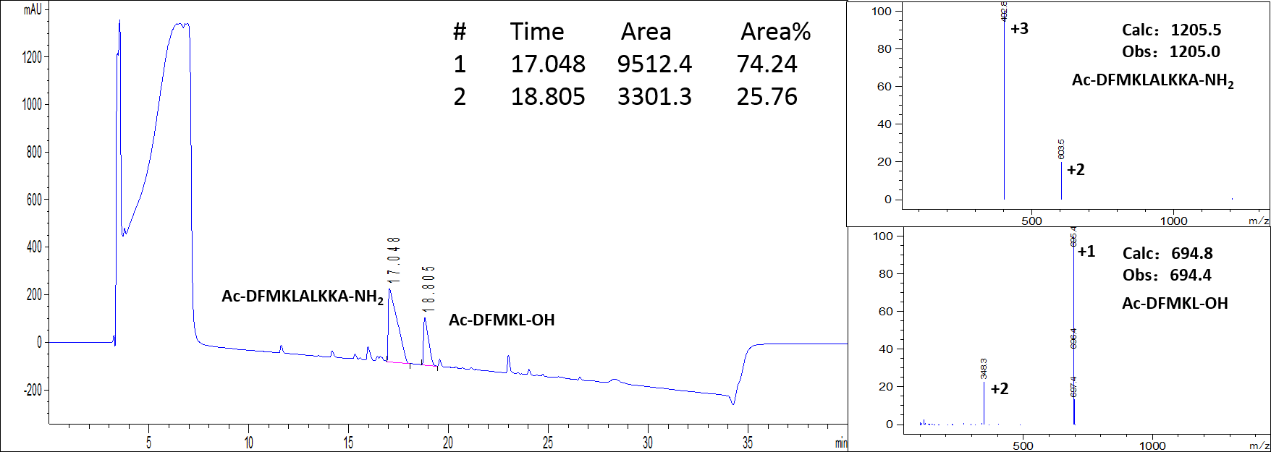


1. **P3 = K, C, A, W**


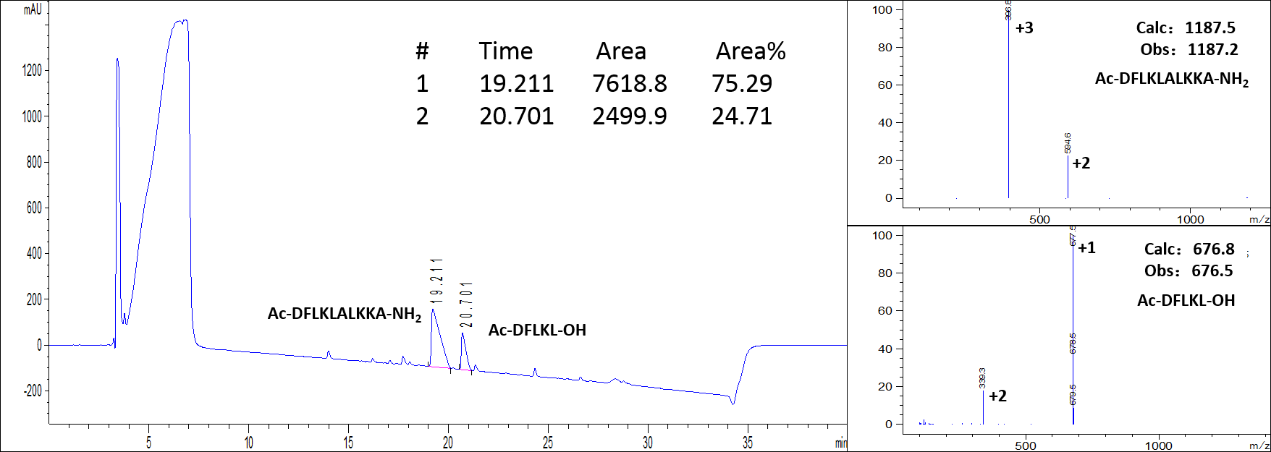

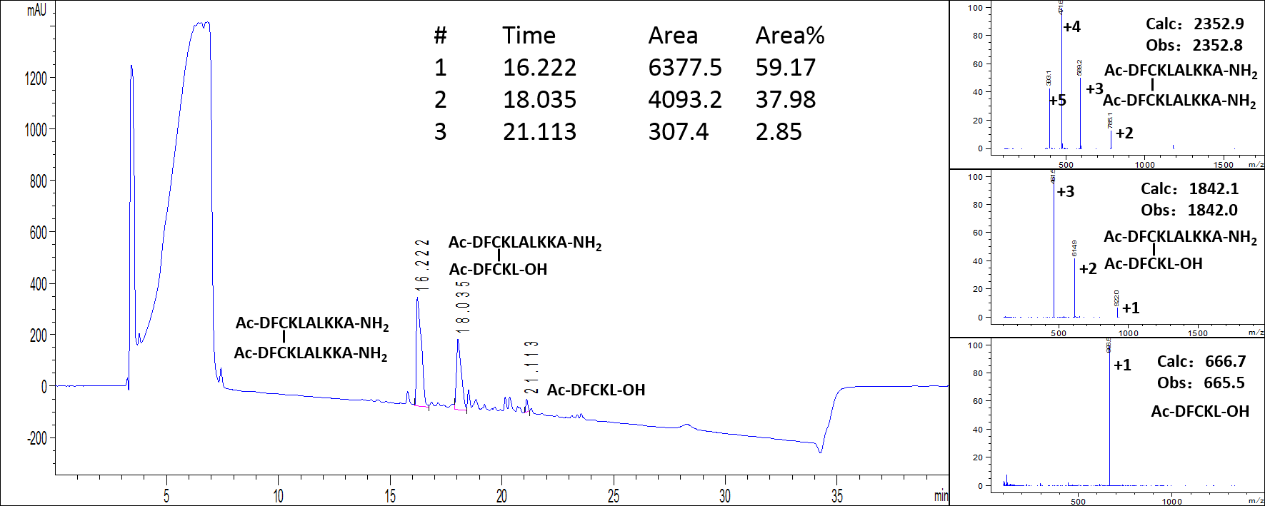

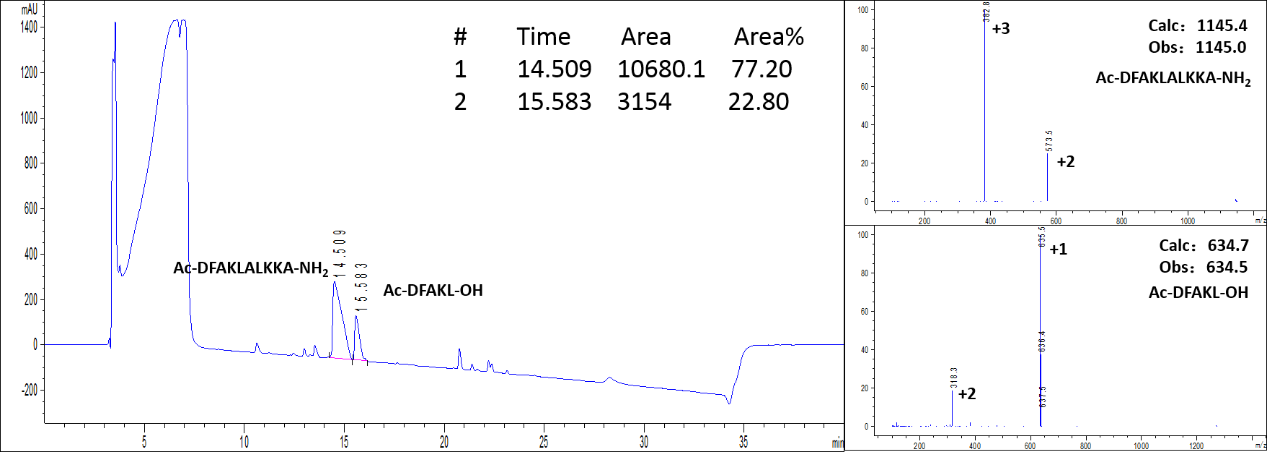

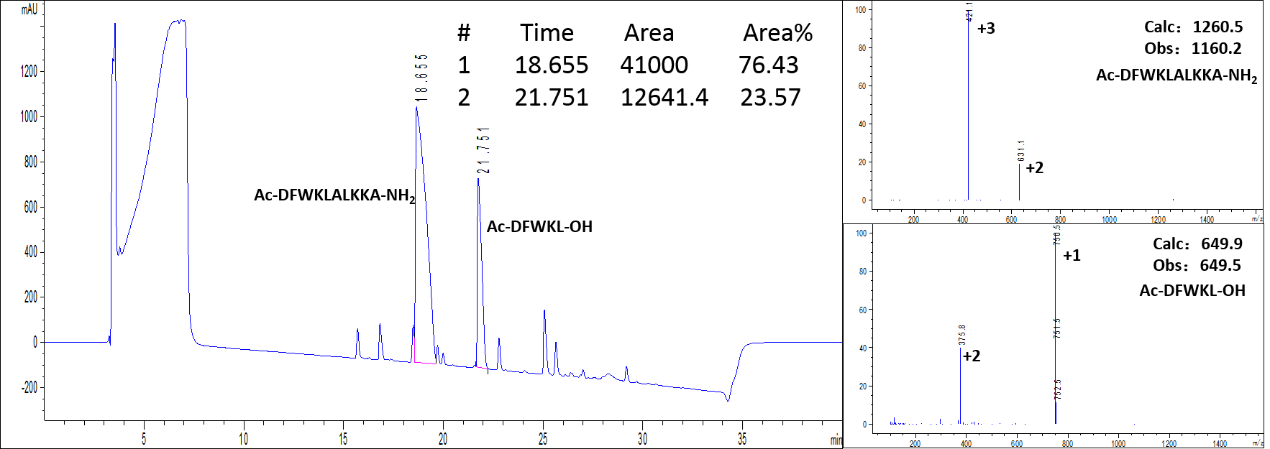


1. **P3 = Y, R, Q, D**


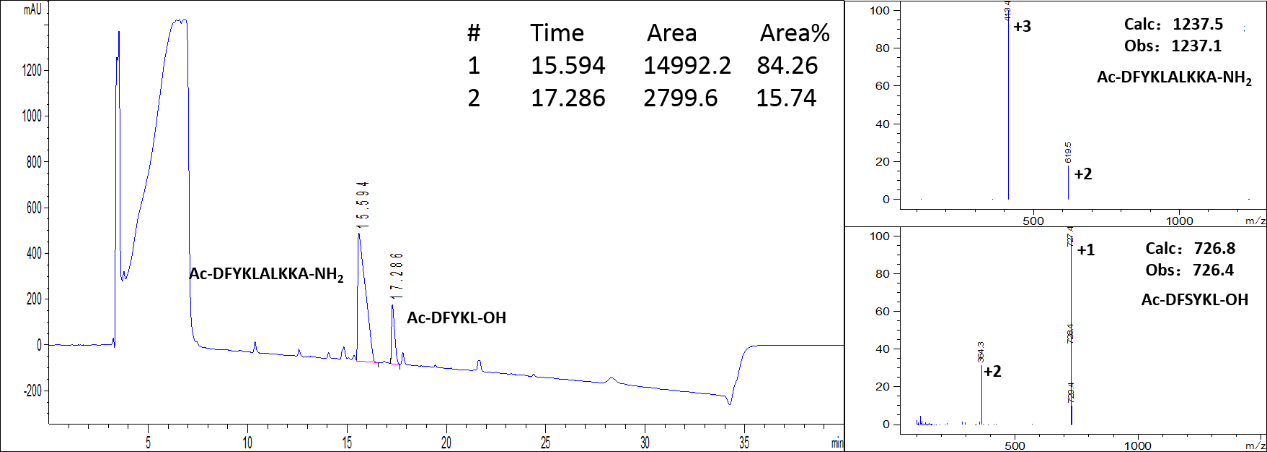

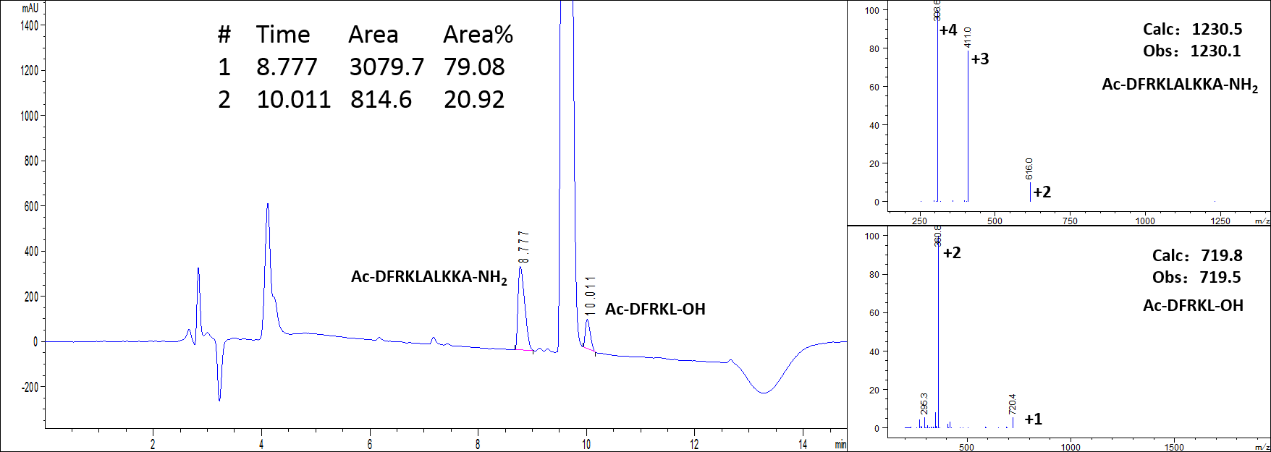

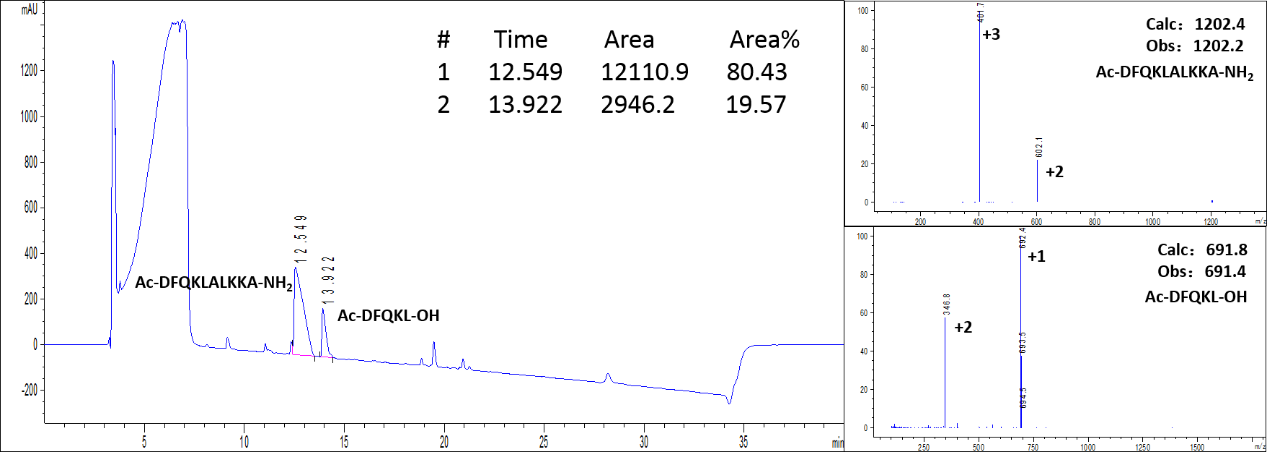

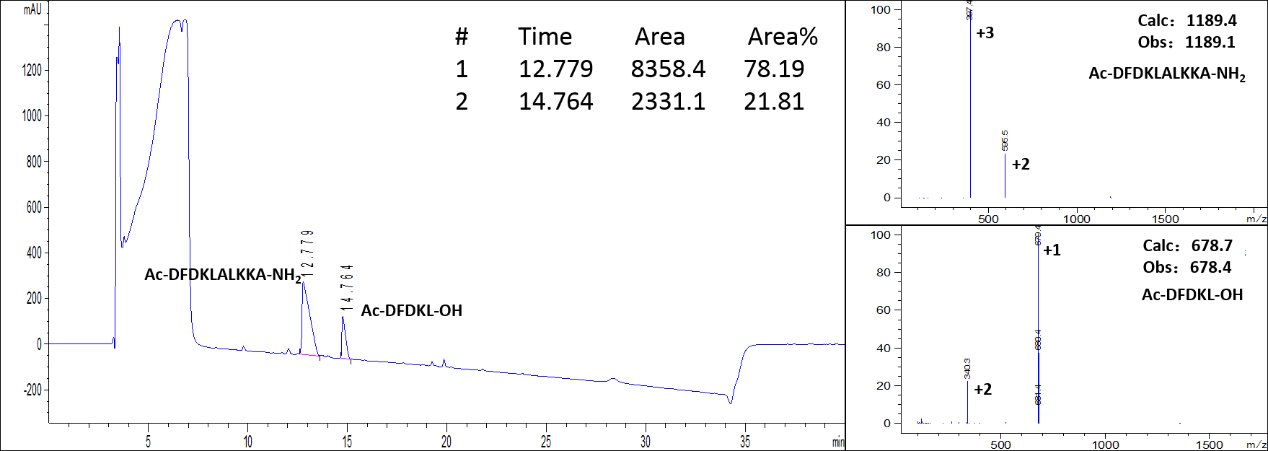


1. **P3 = N, G, H, E**


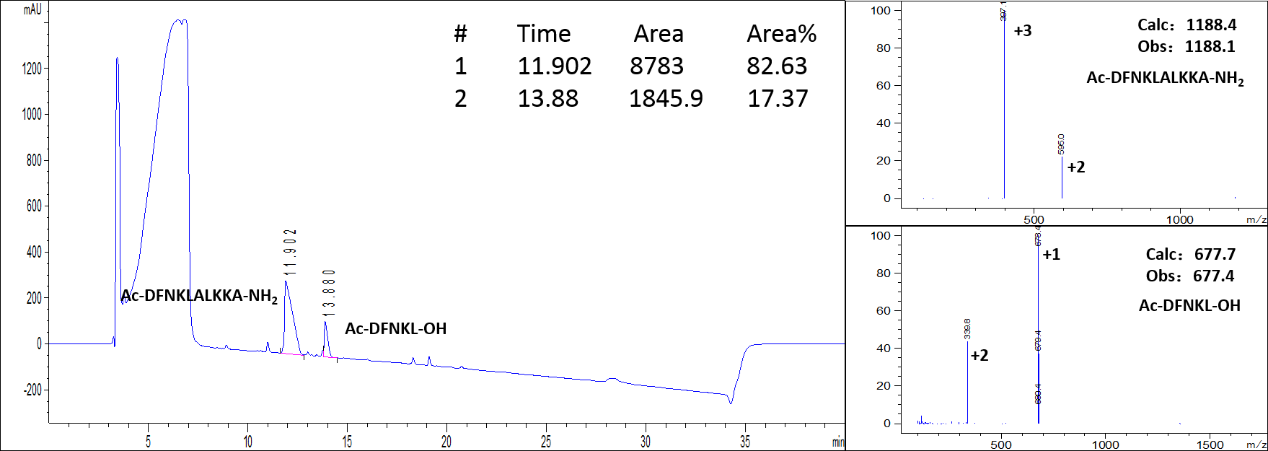

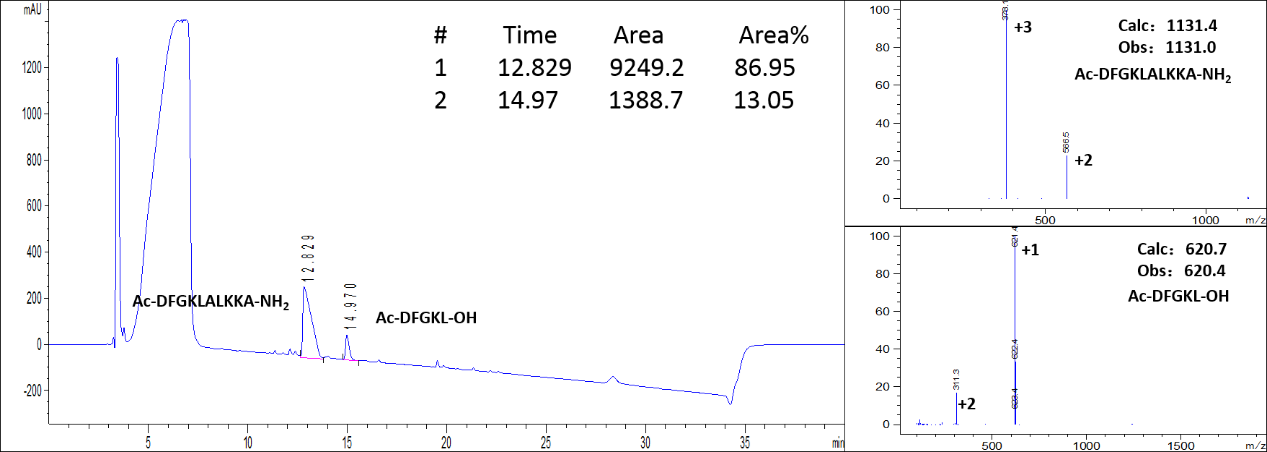

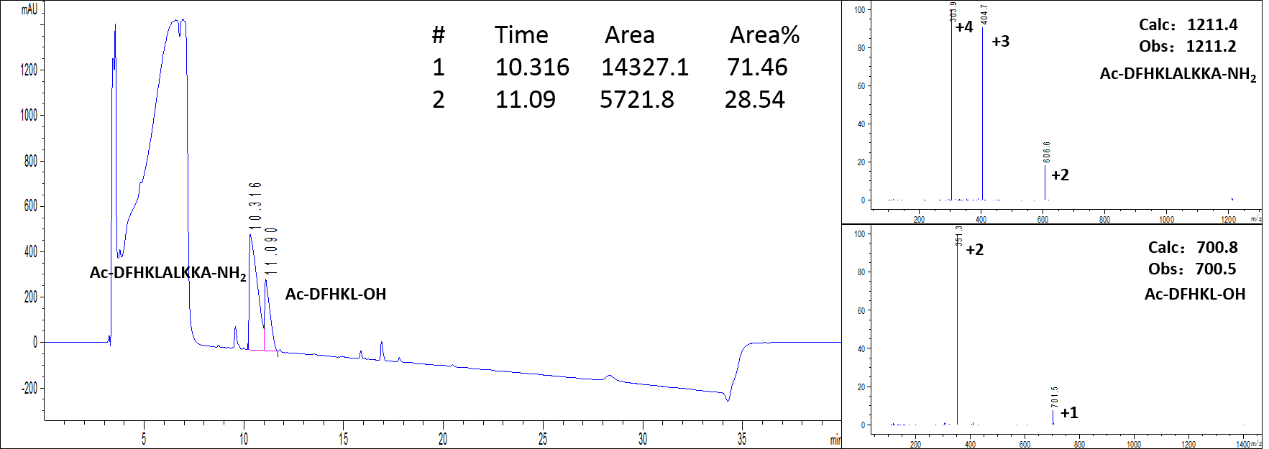

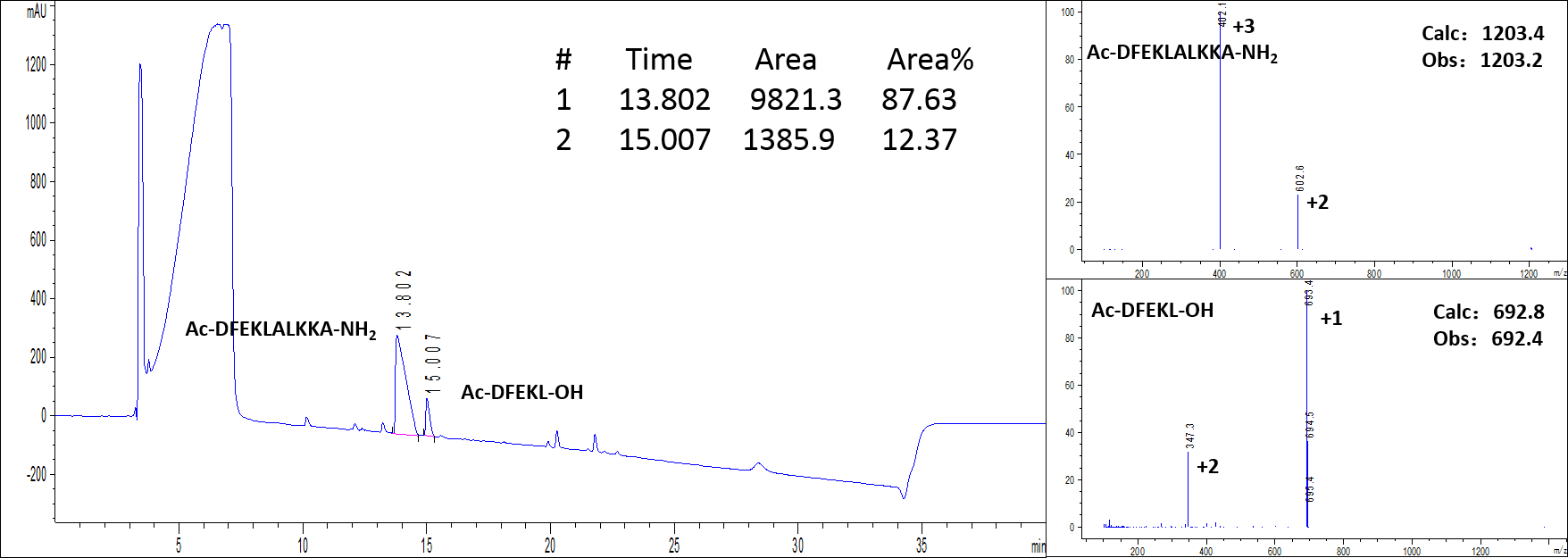


1. **P3 = K, P, T**


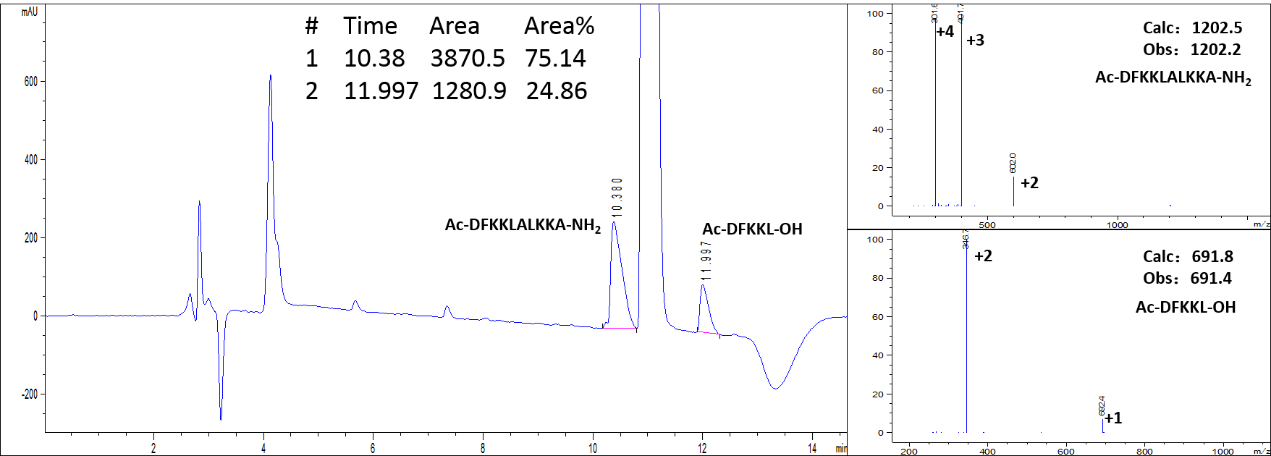

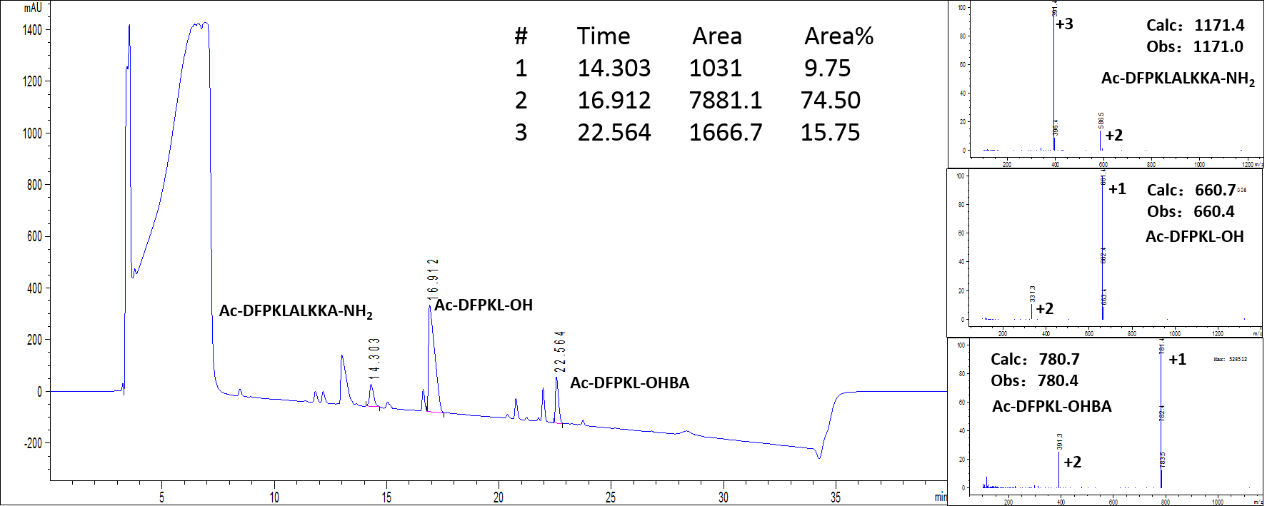

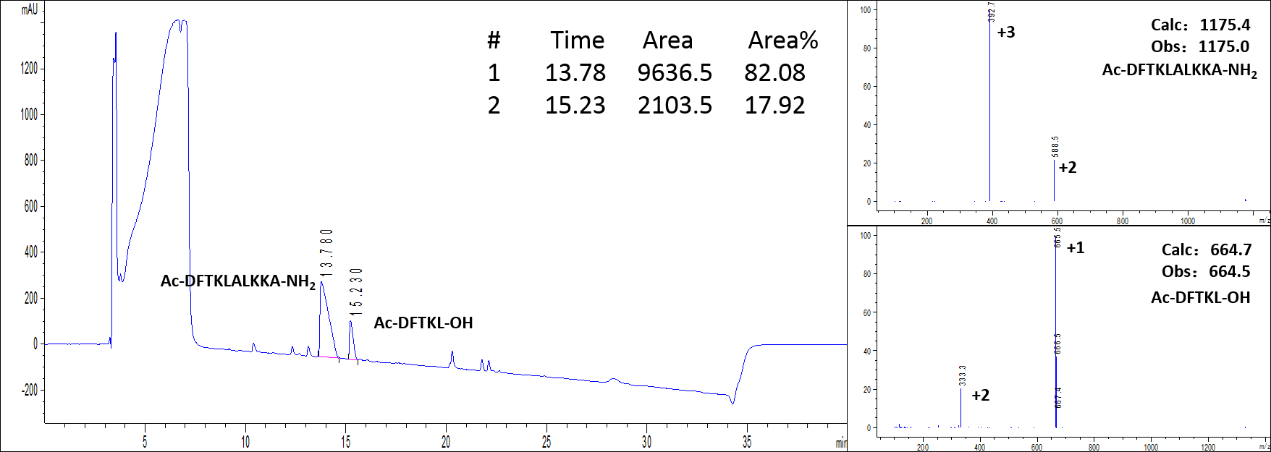


## 2.3 screening of Omniligase-1 P2 substrates

1. **P2 = F, V, I, M**


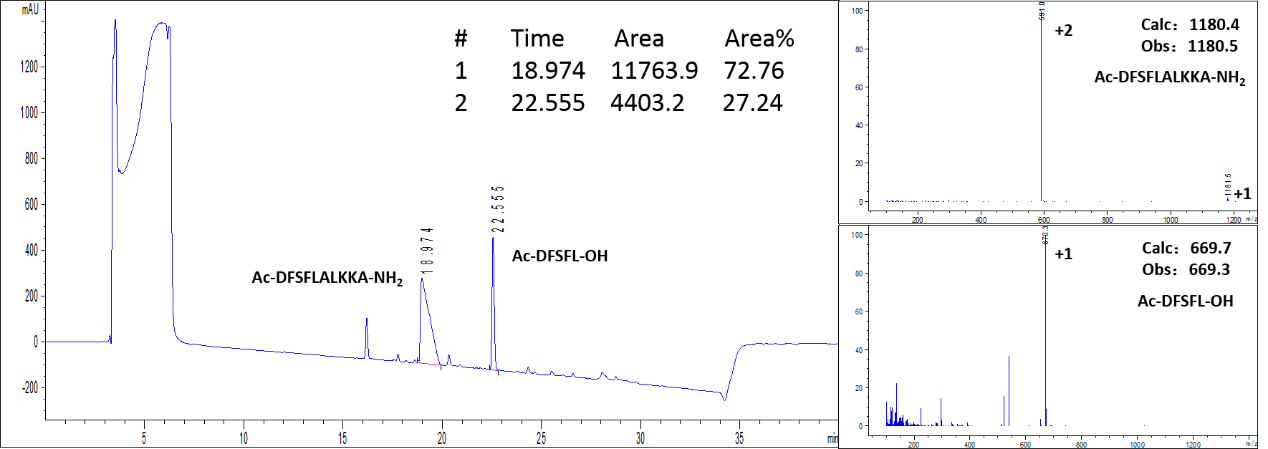

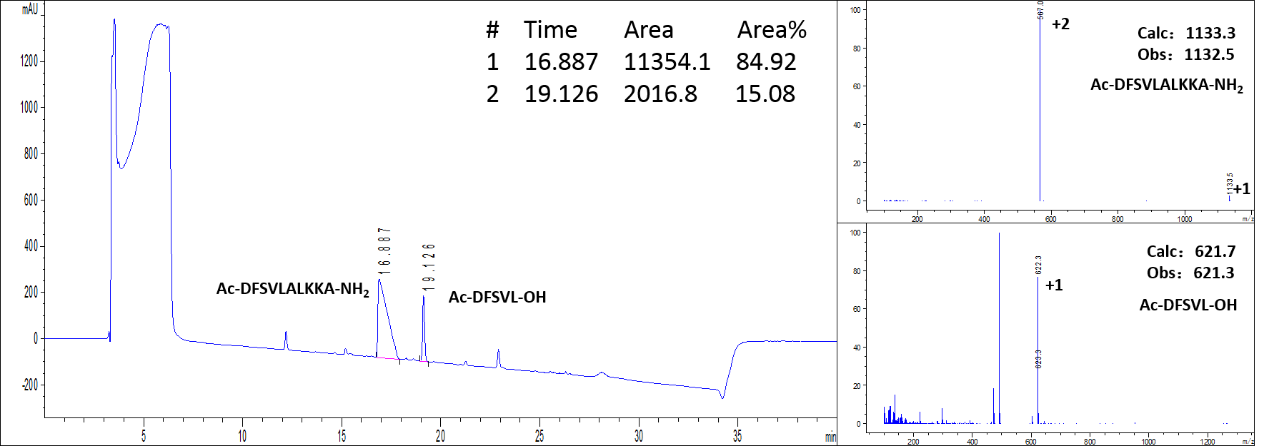

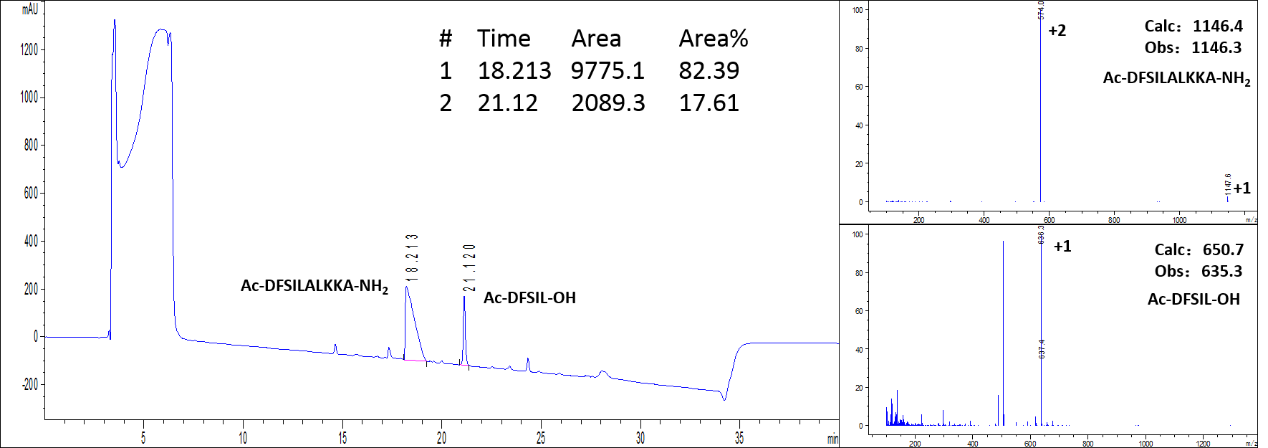

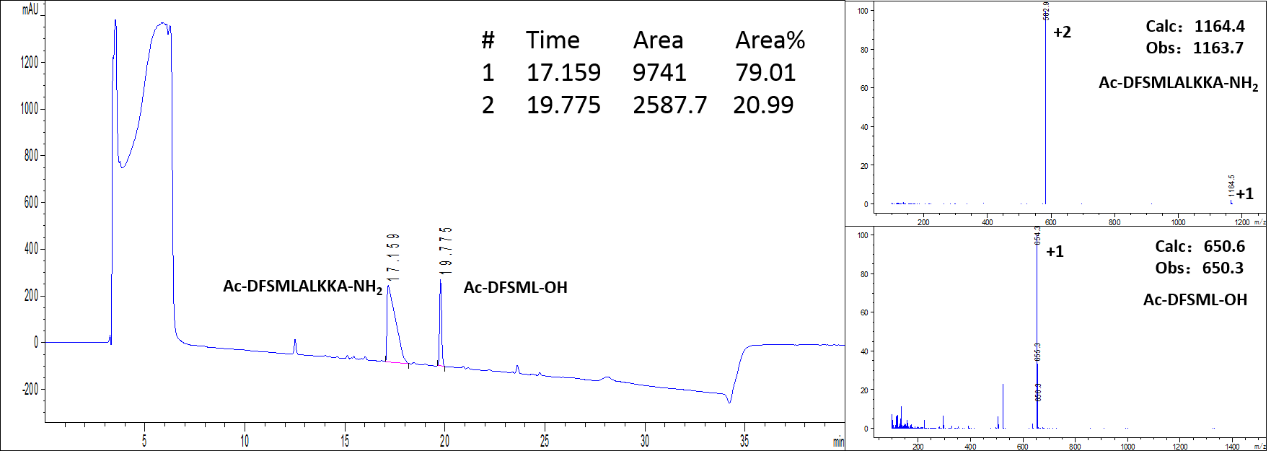


1. **P2 = L, C, A, W**


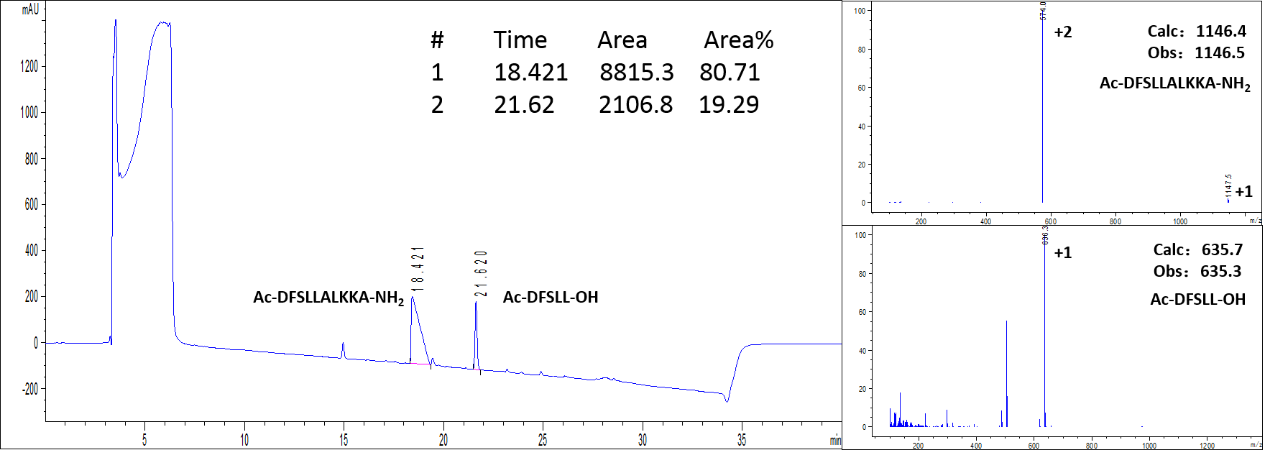

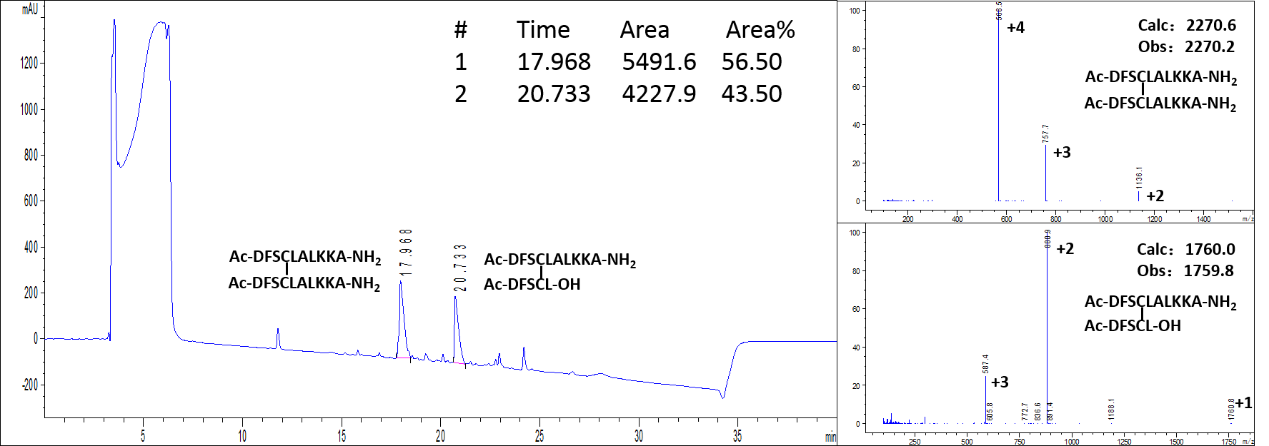

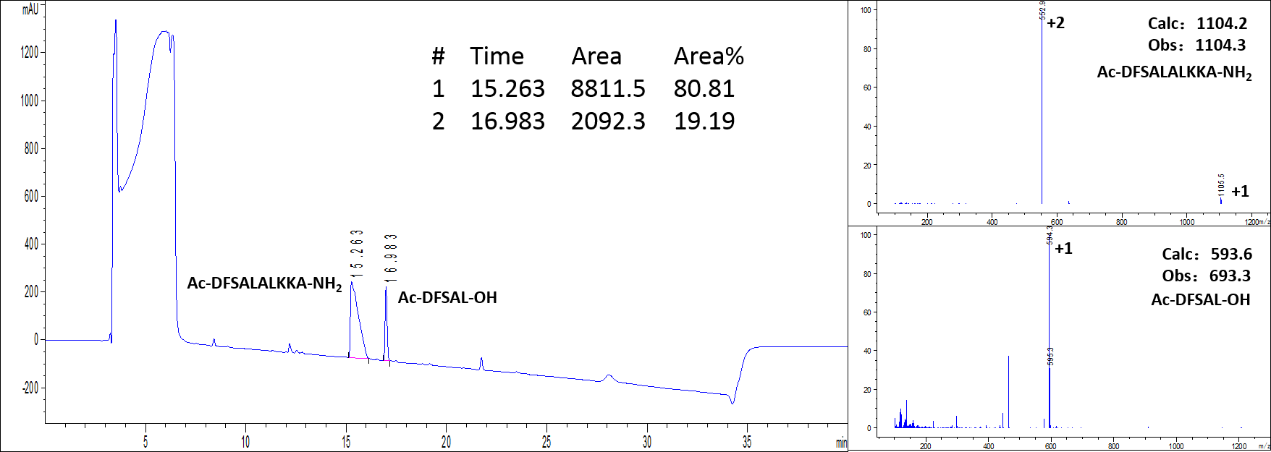

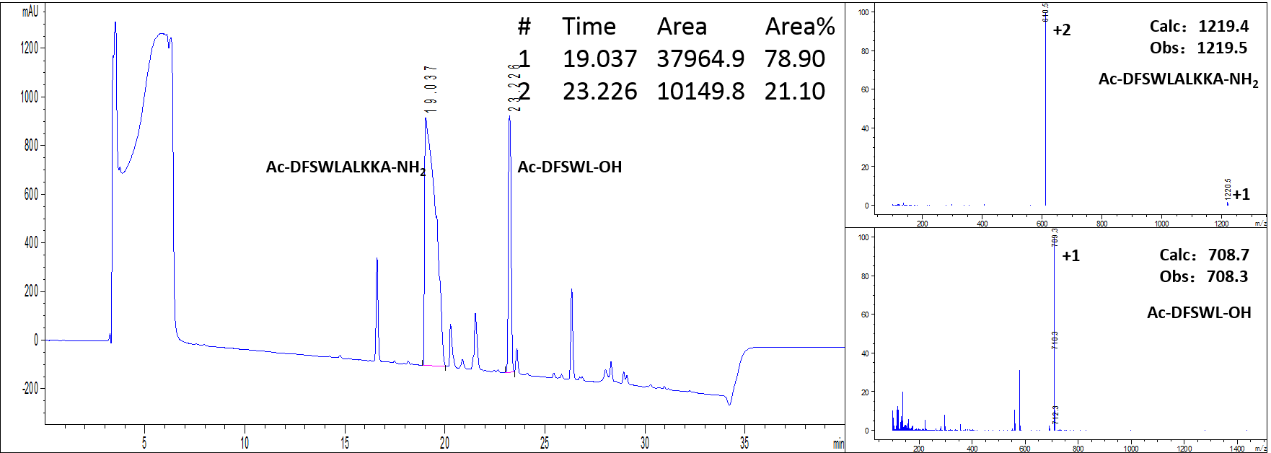


1. **P2 = Y, S, R, Q**


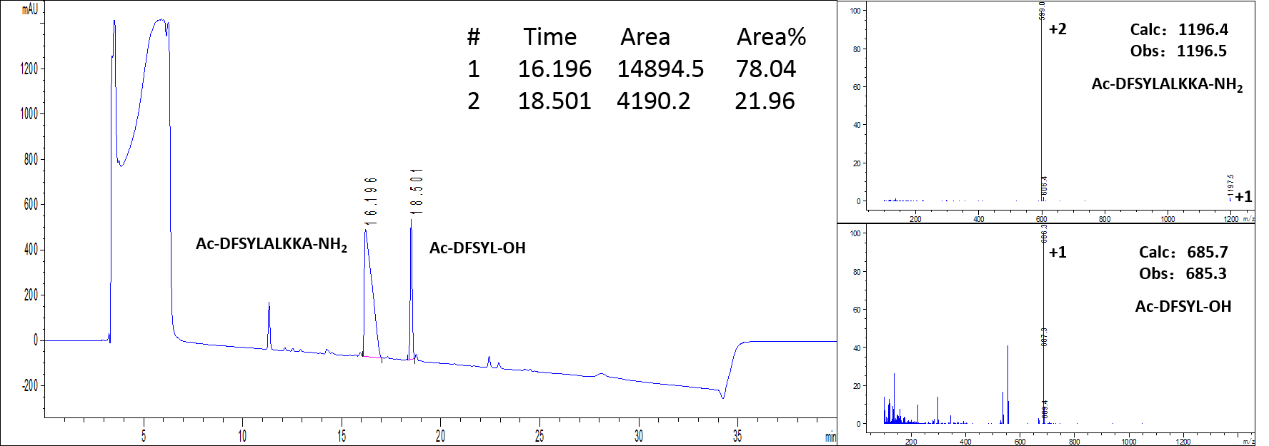

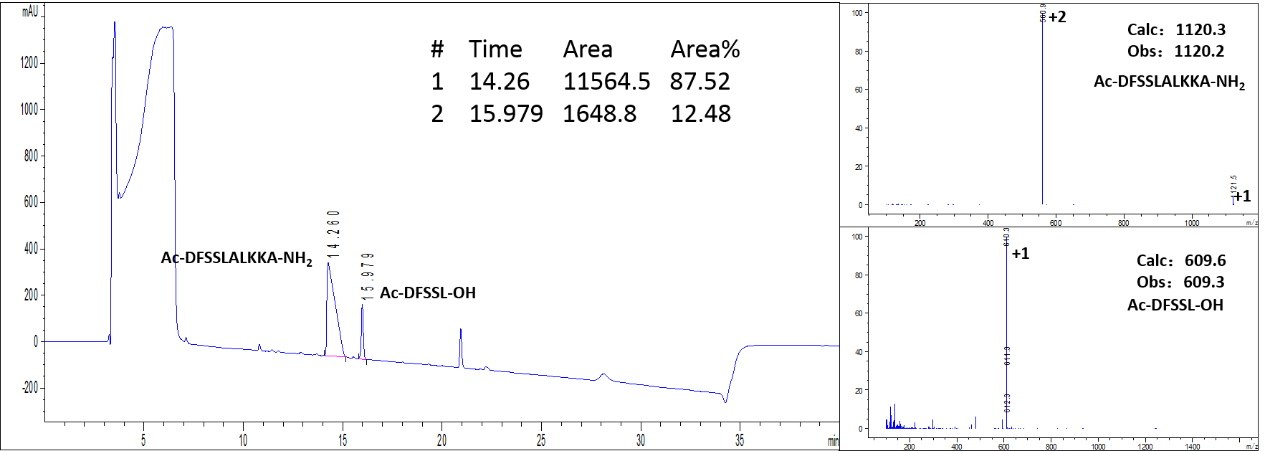

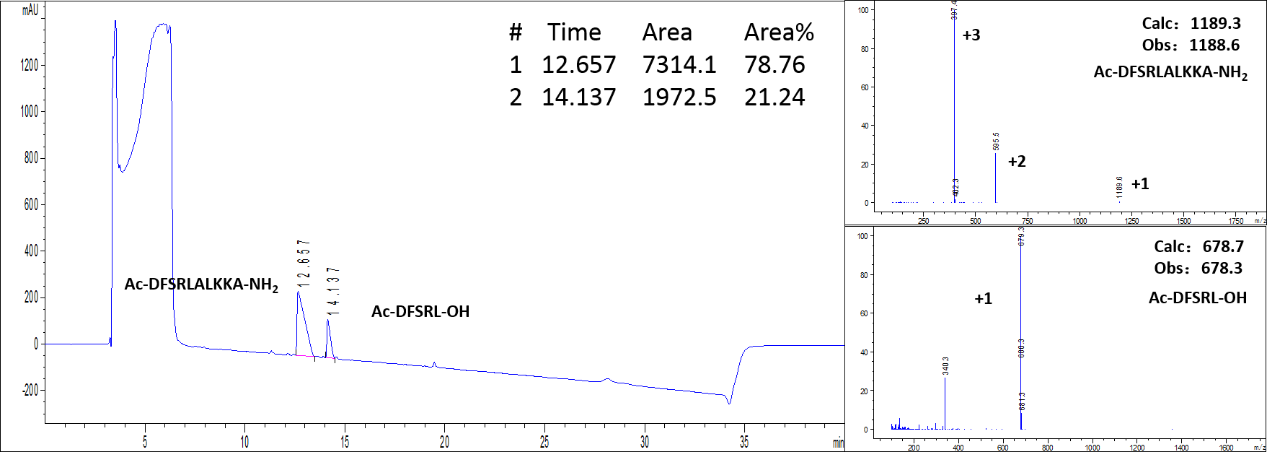

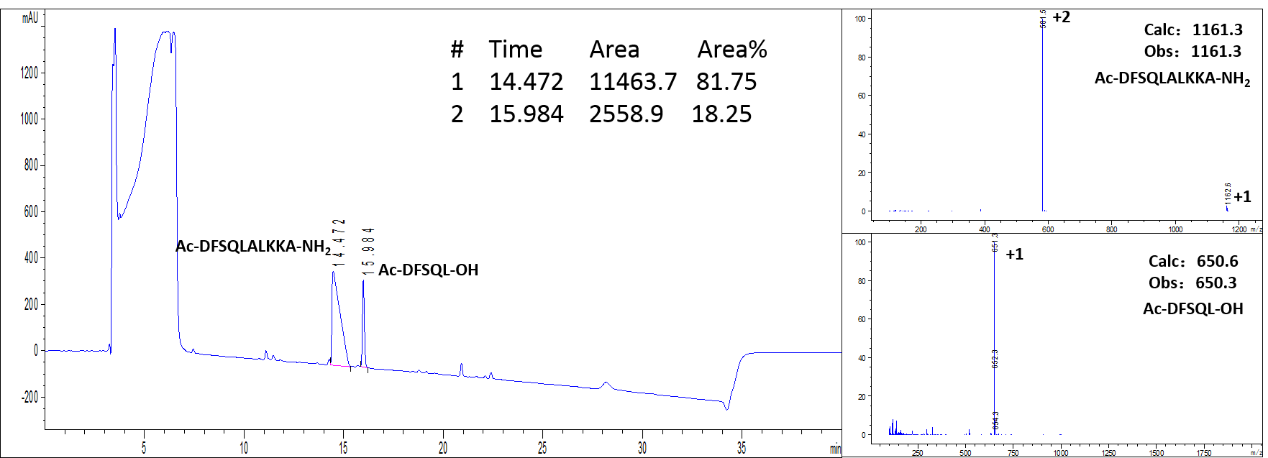


1. **P2 = D, N, G, H**


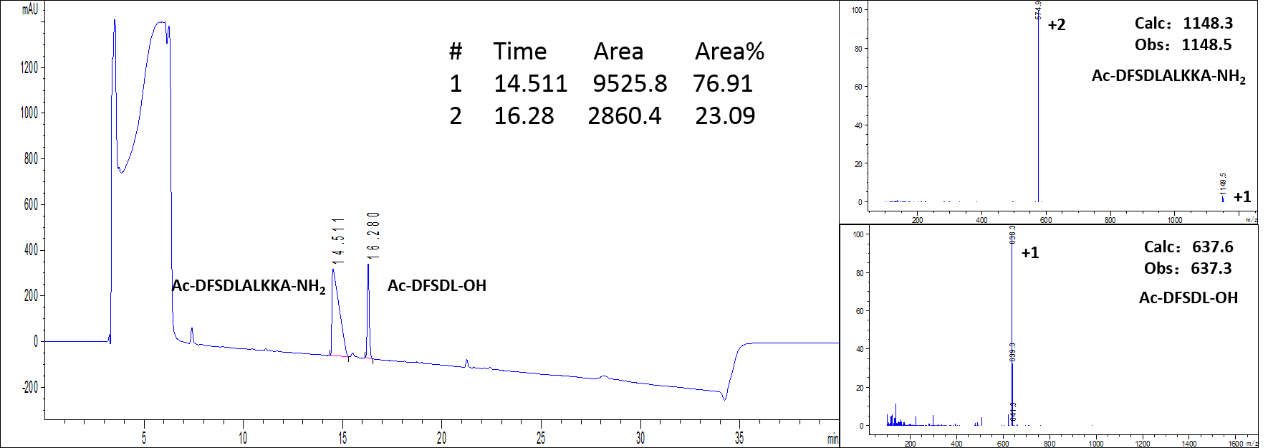

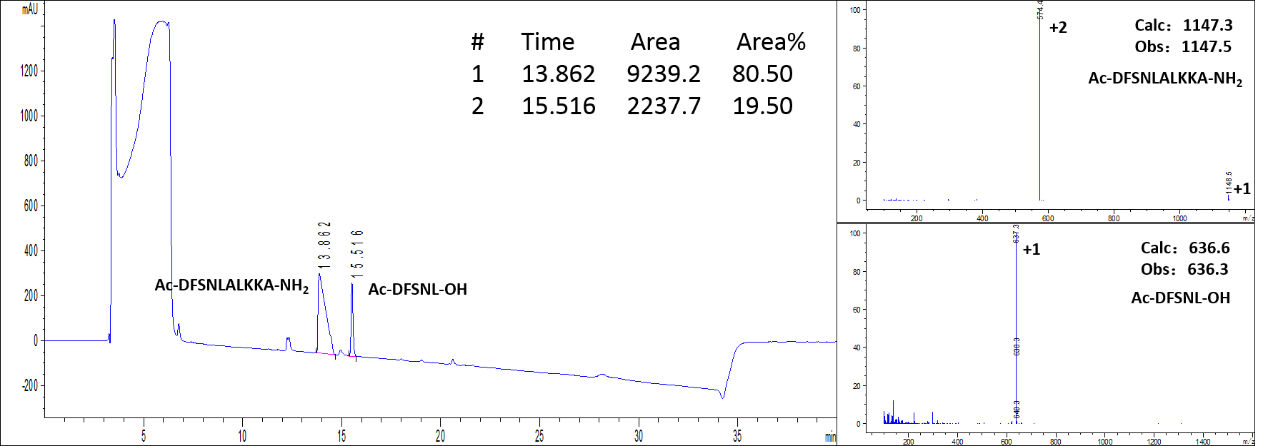

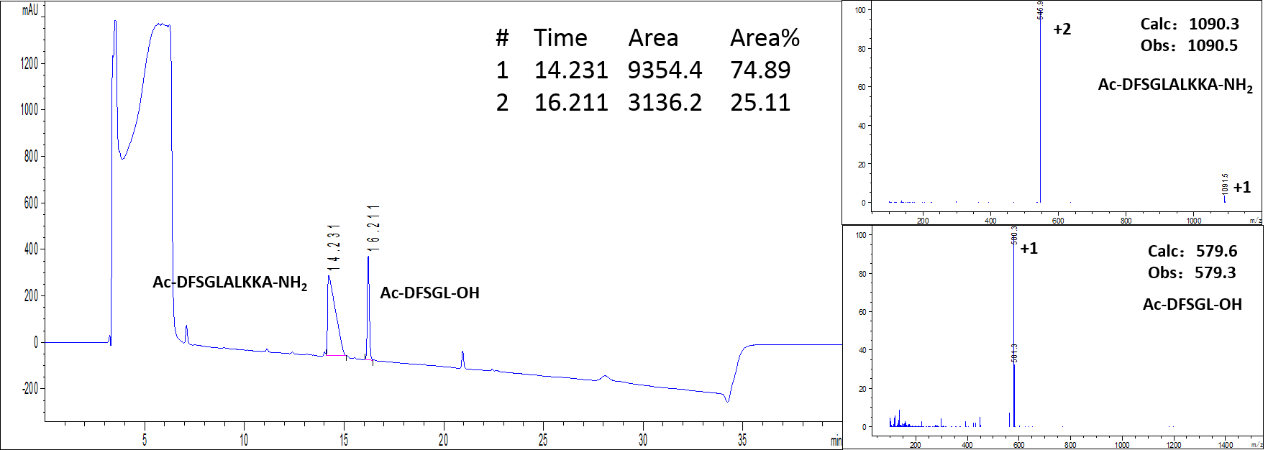

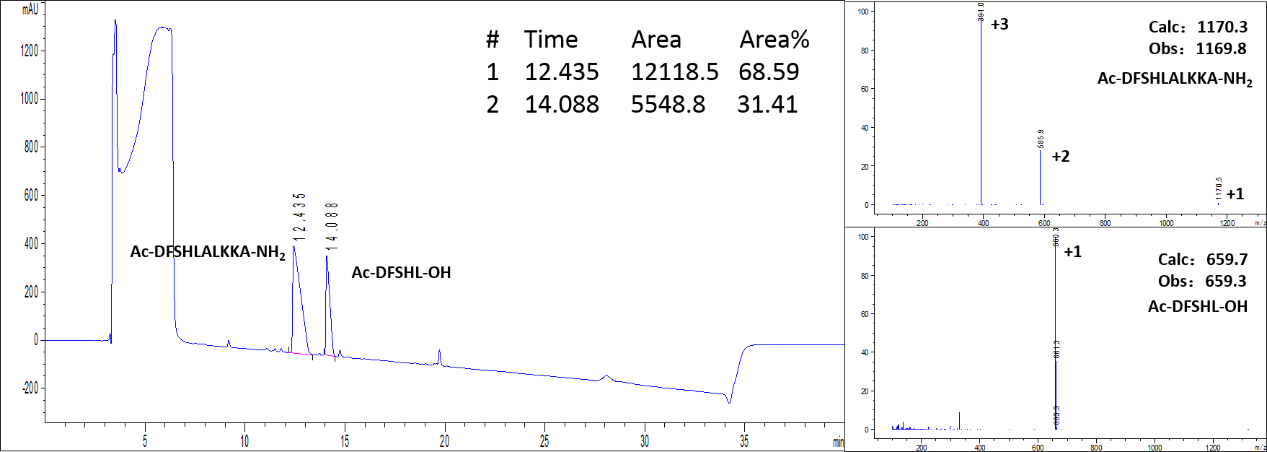


1. **P2 = E, P, T**


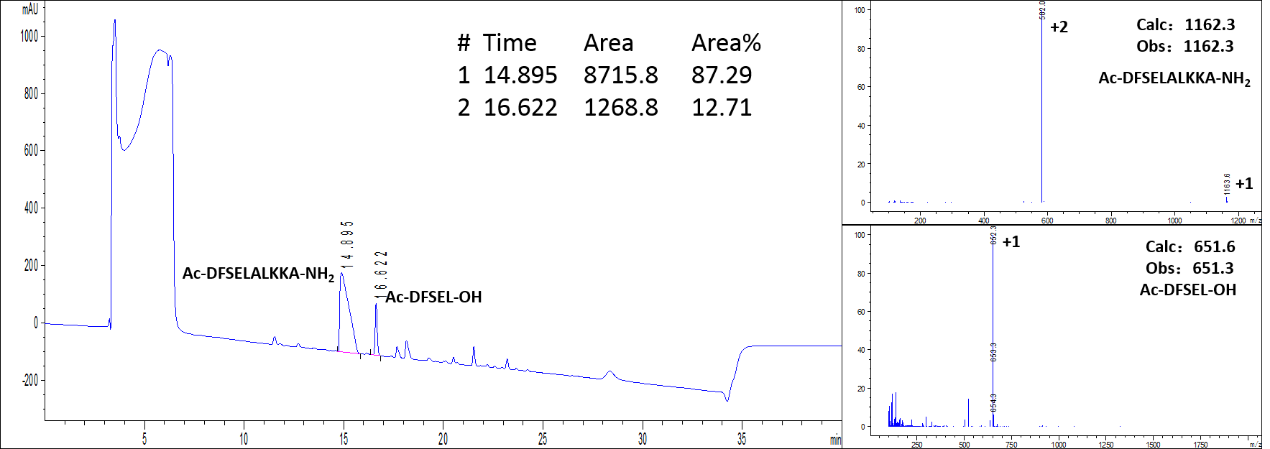

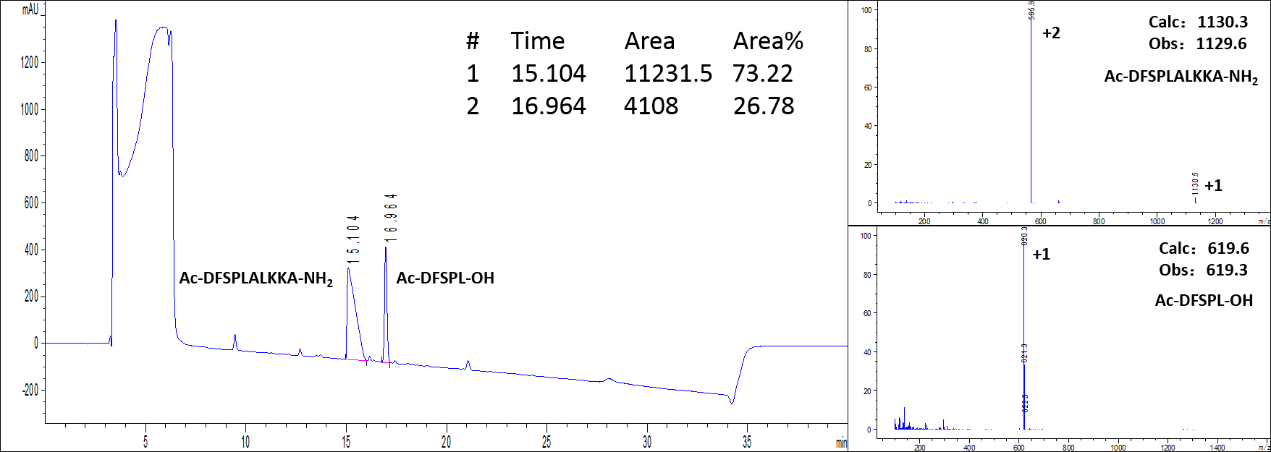

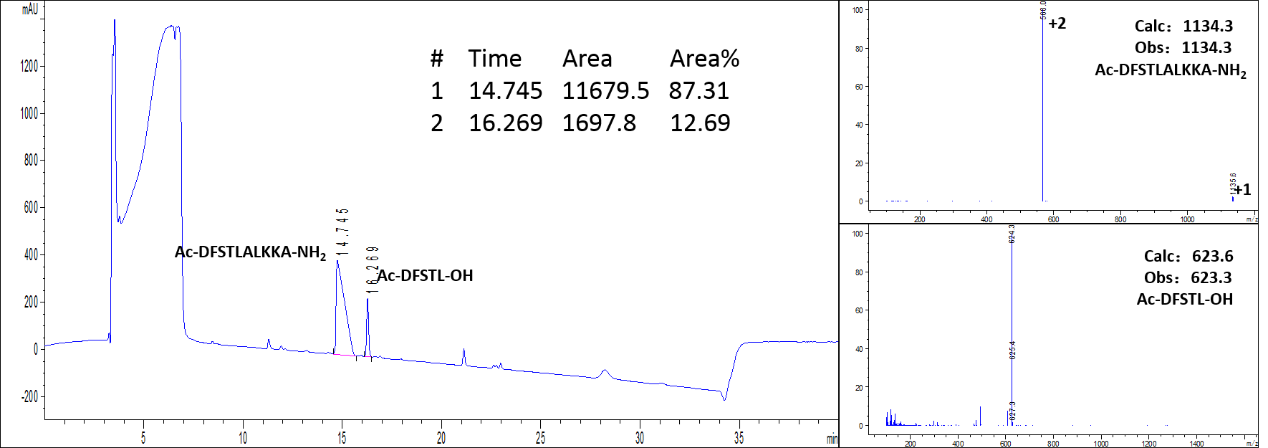


## 2.4 screening of Omniligase-1 P1 substrates

1. **P1 = C, R, D, P**


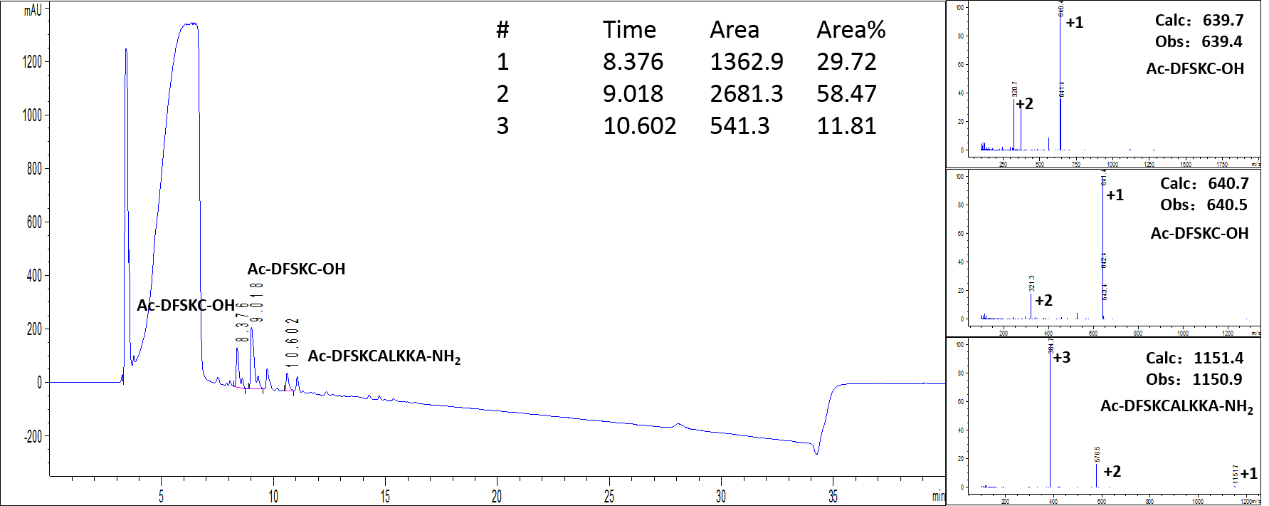

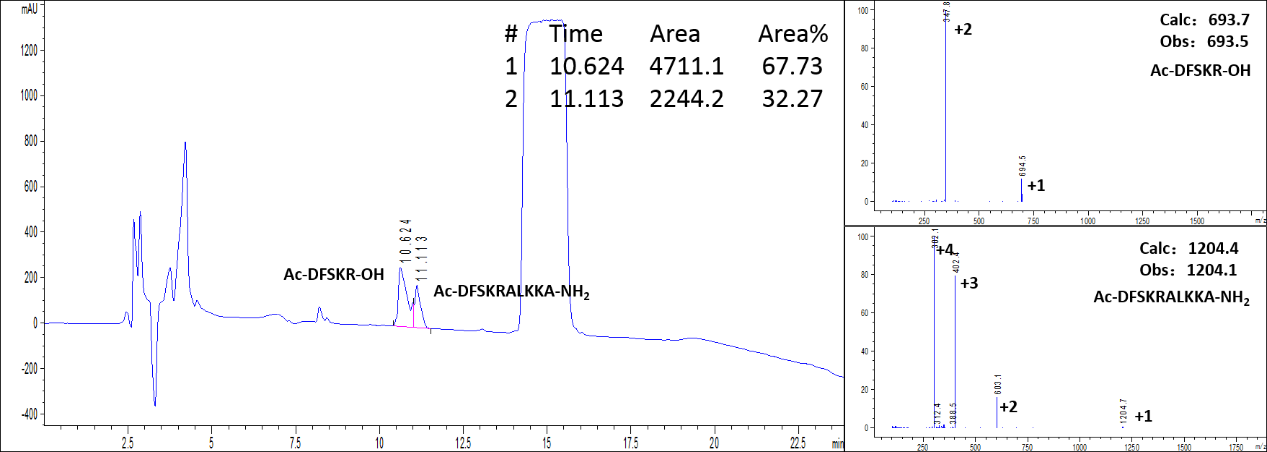

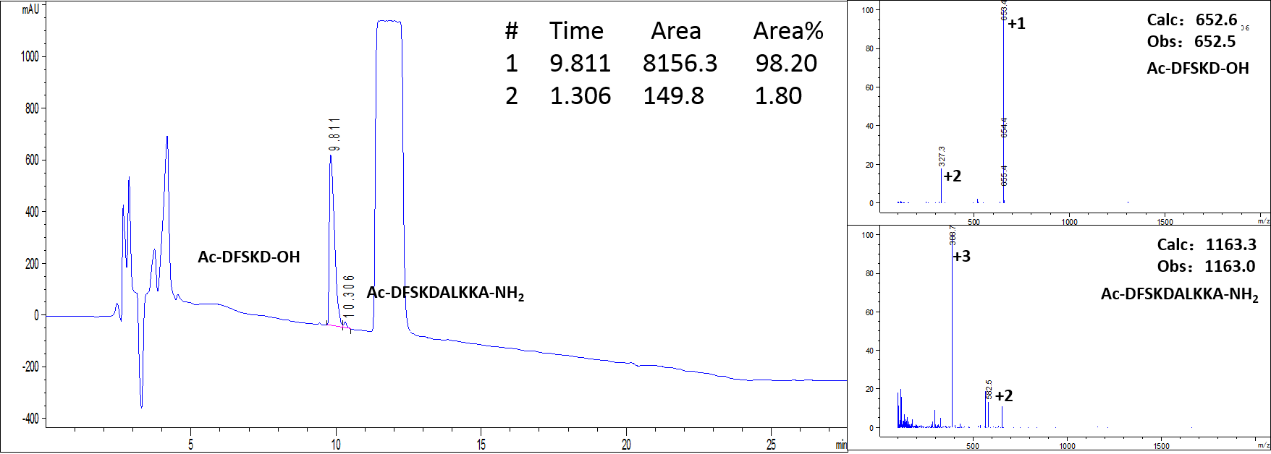

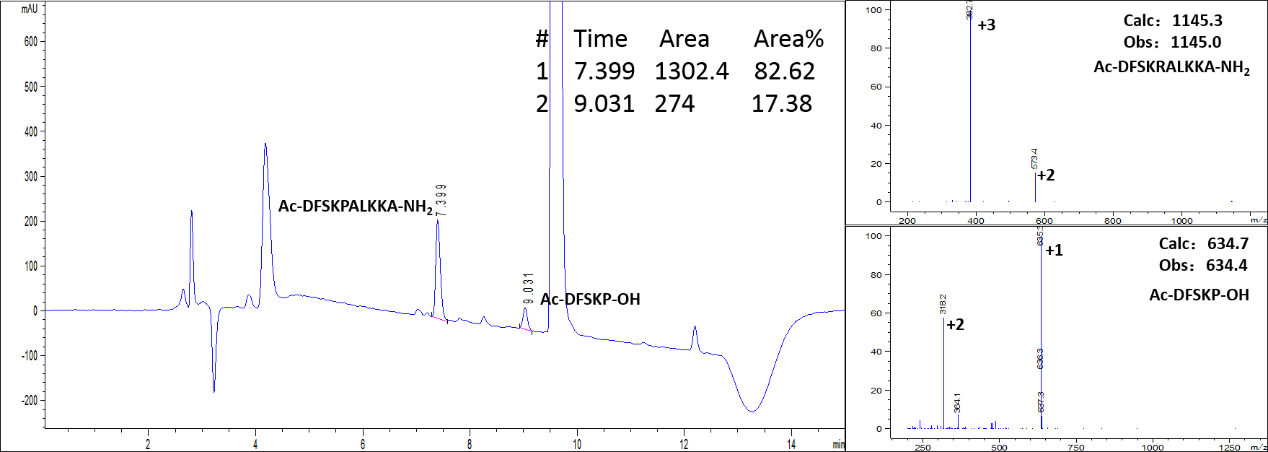


1. **P1 = F, V, I, M**


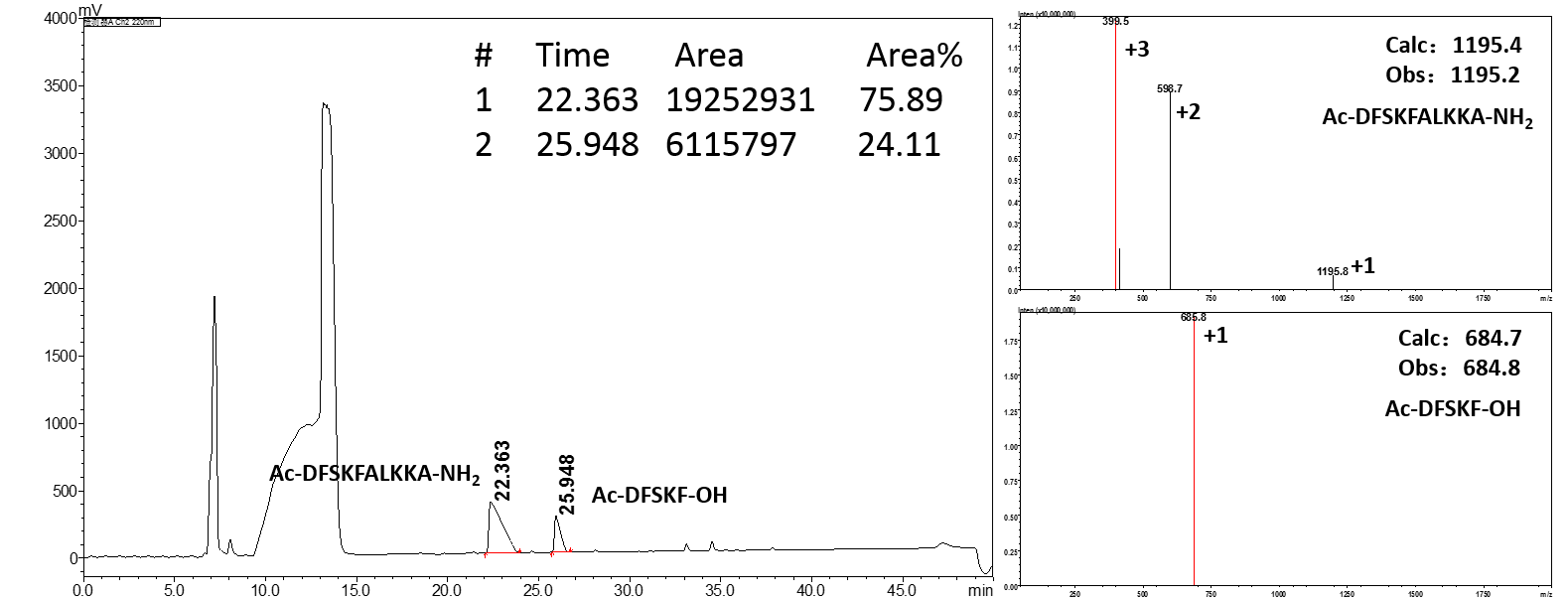

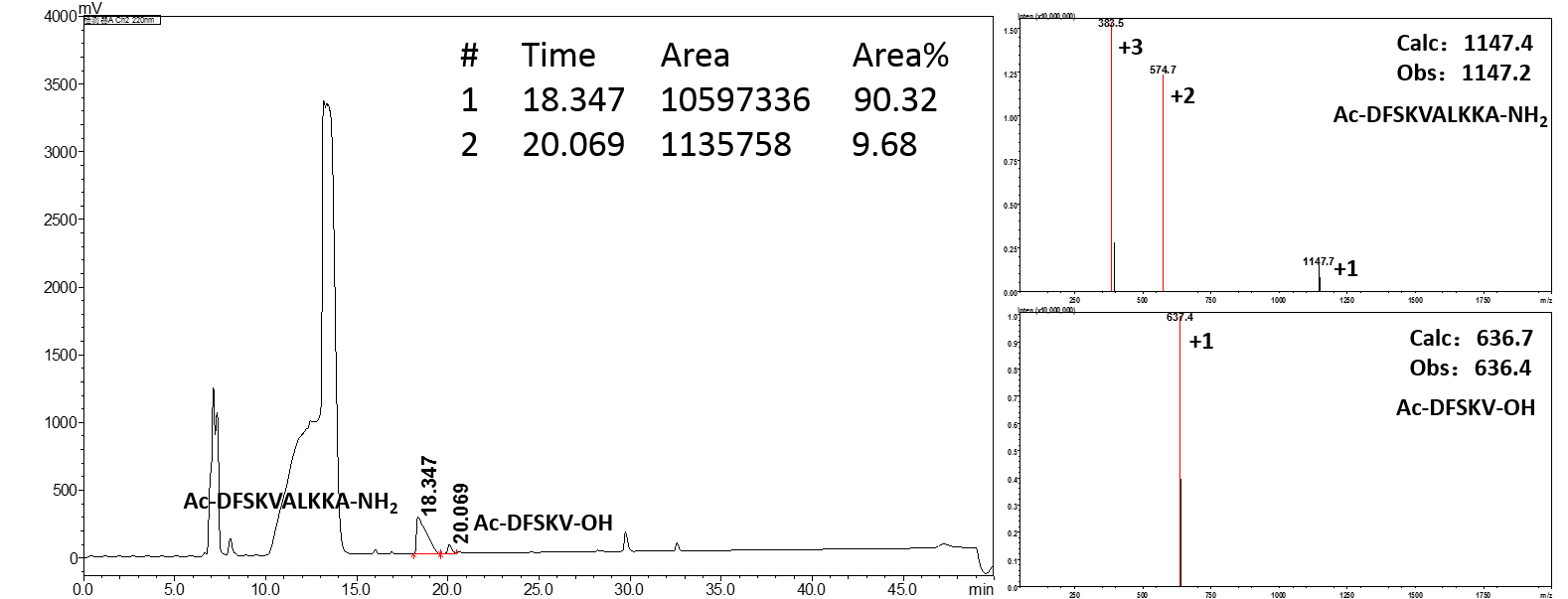

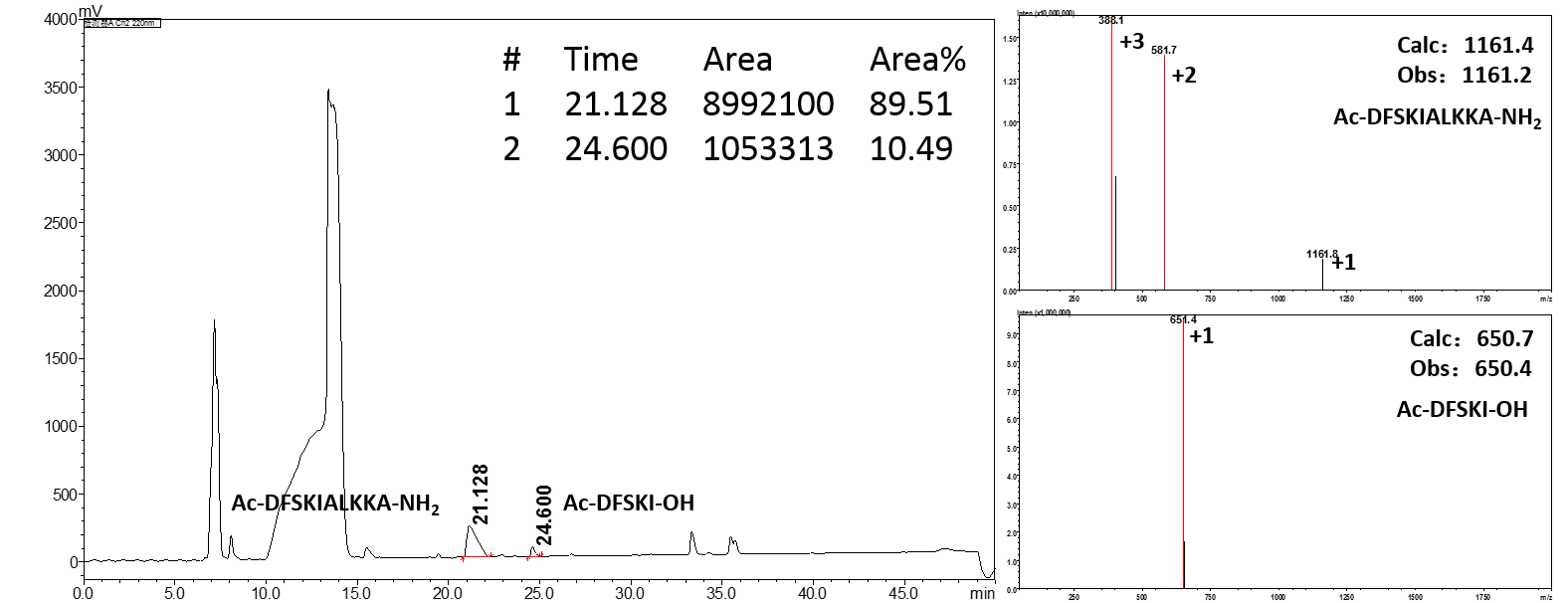

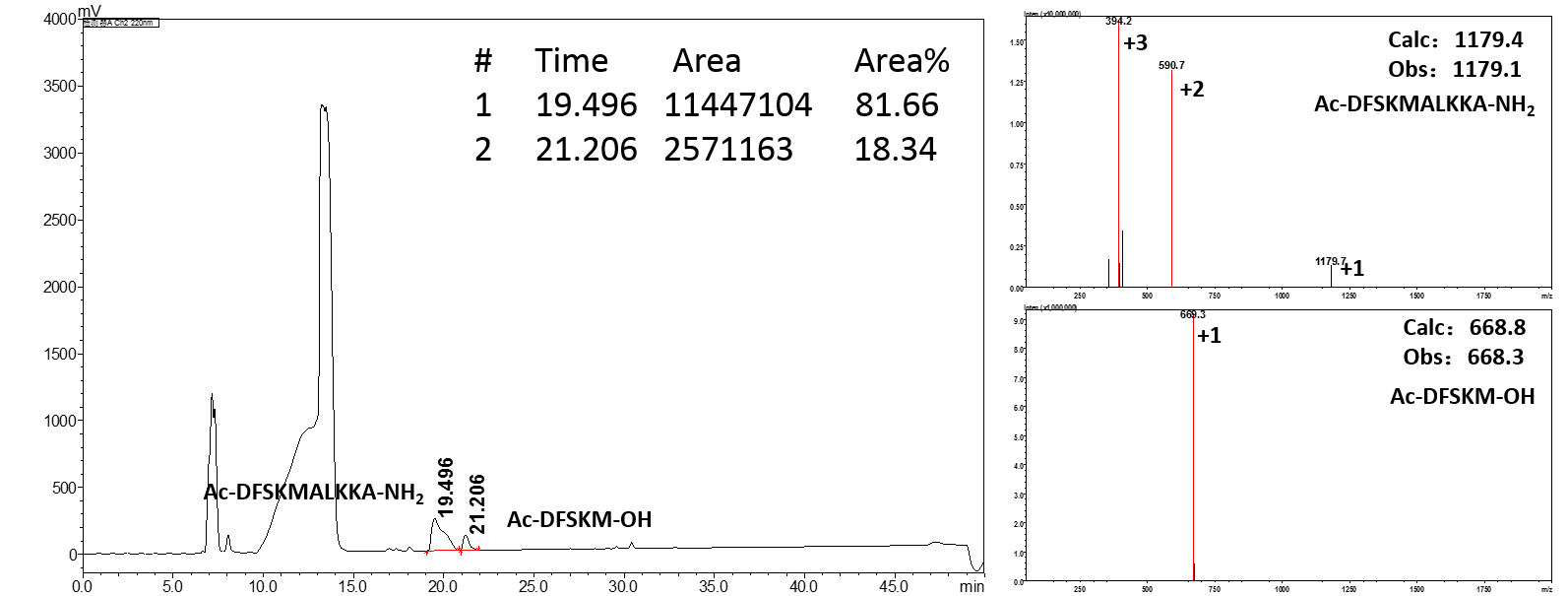


1. **P1 = A, W, Y, S**


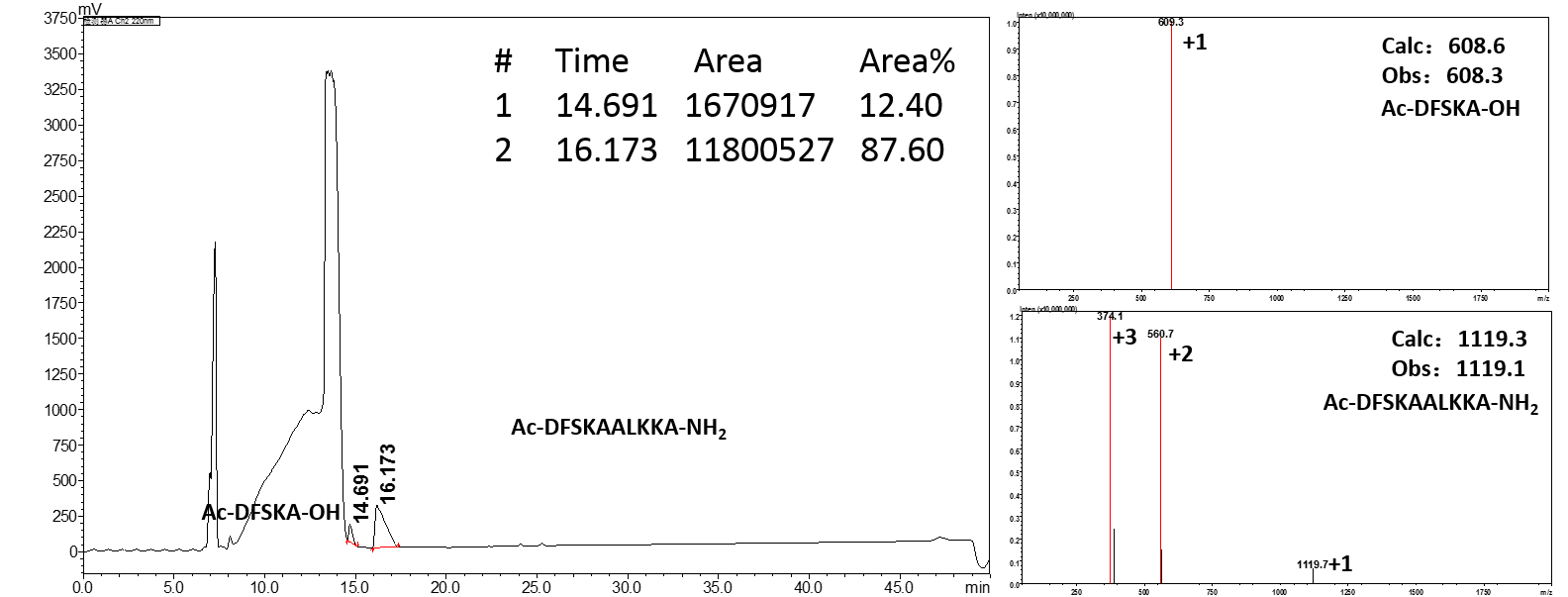

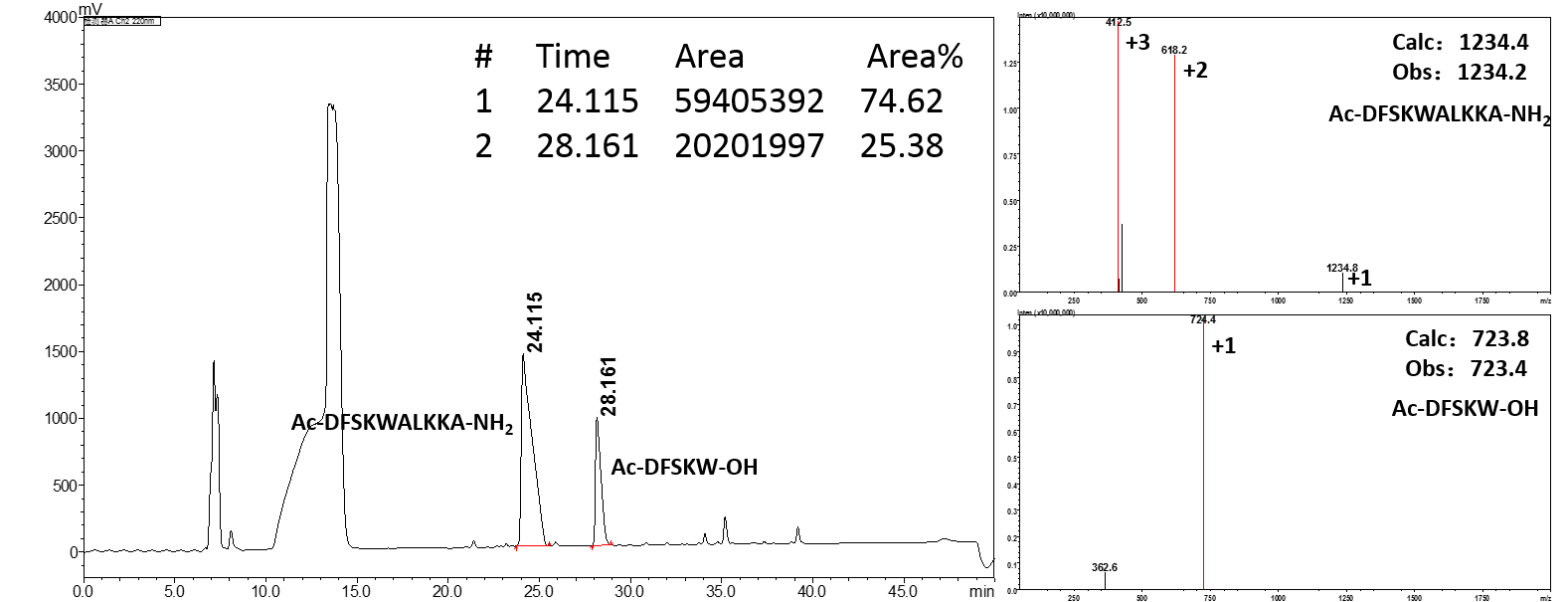

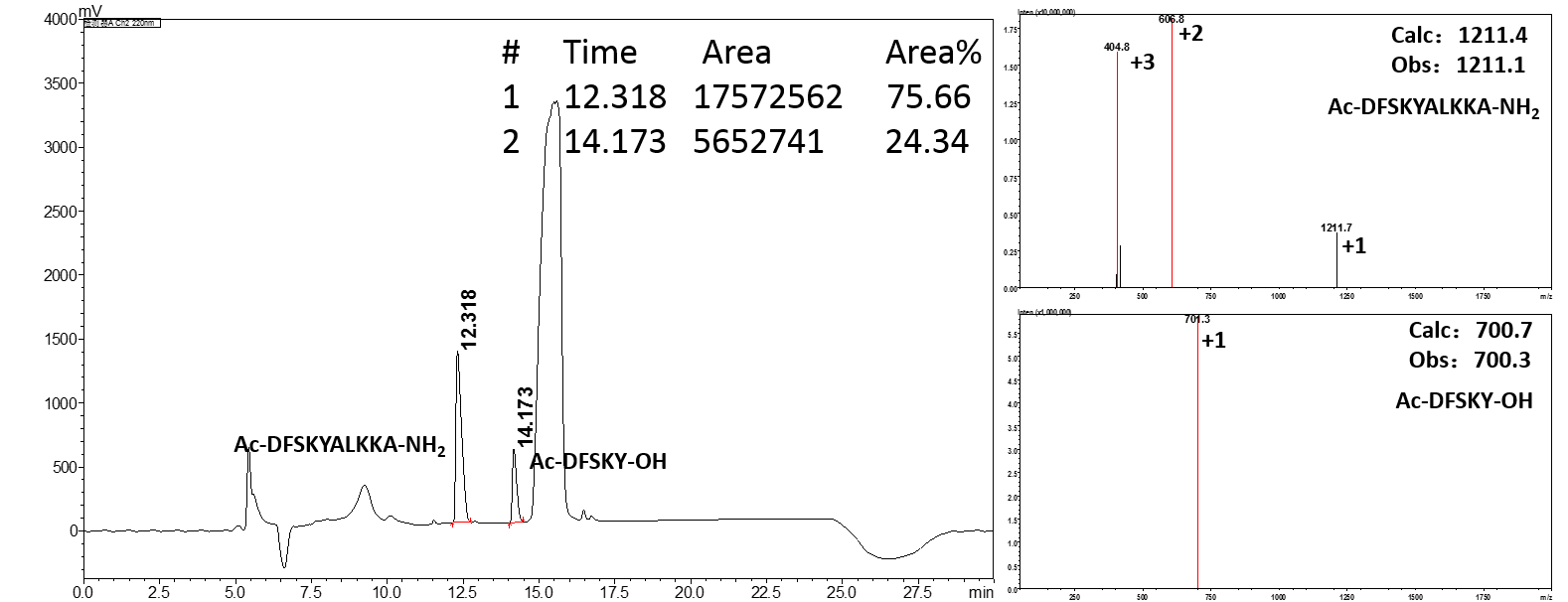

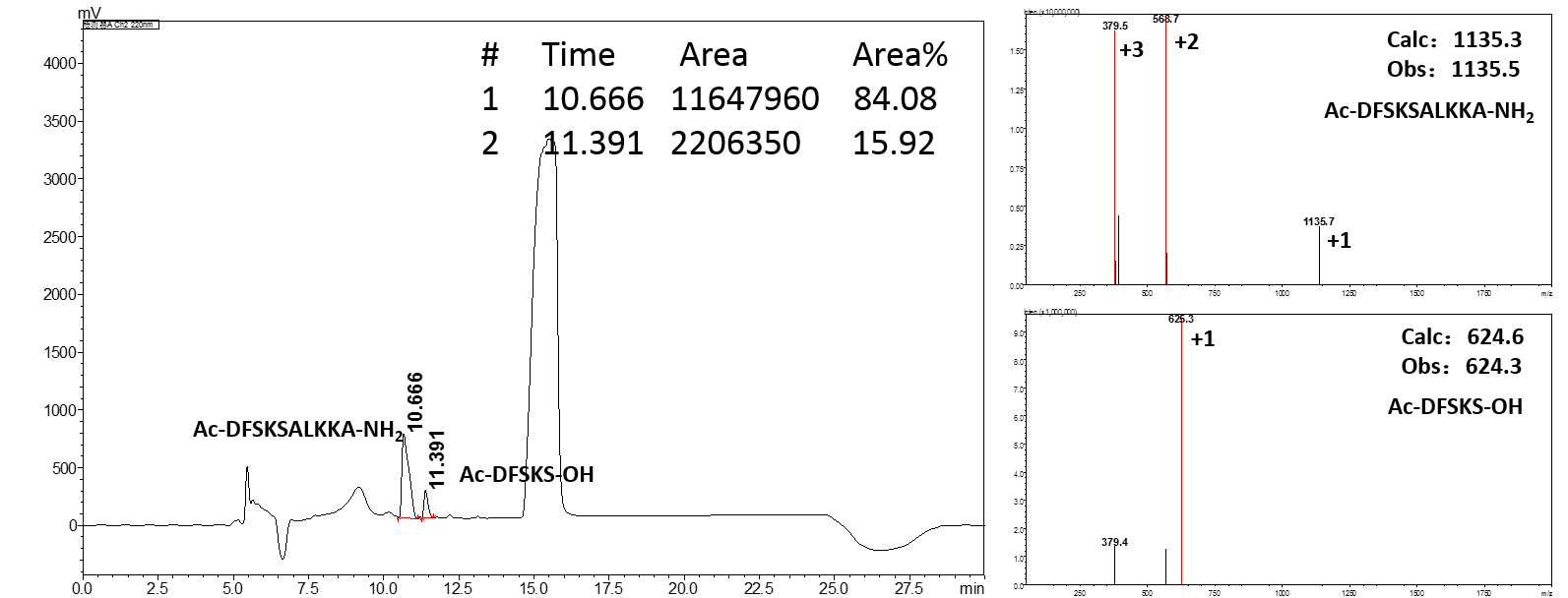


1. **P1 = Q, N, G, H**


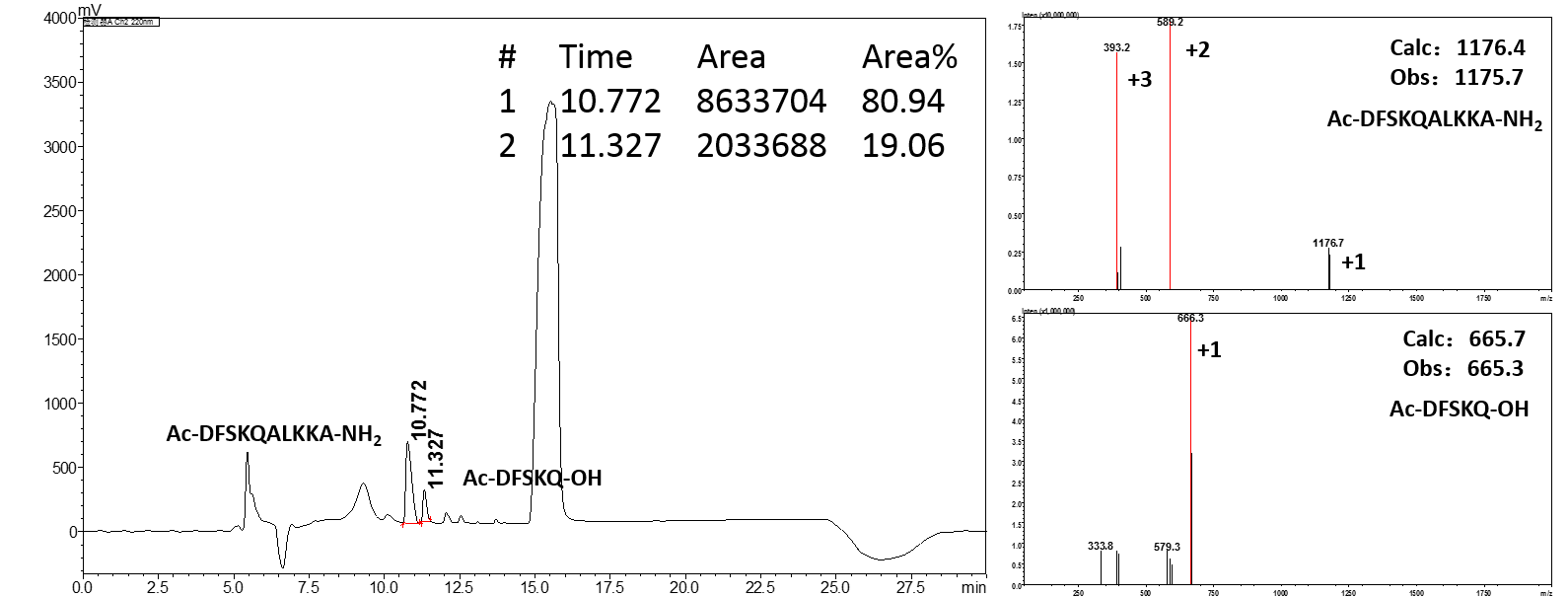

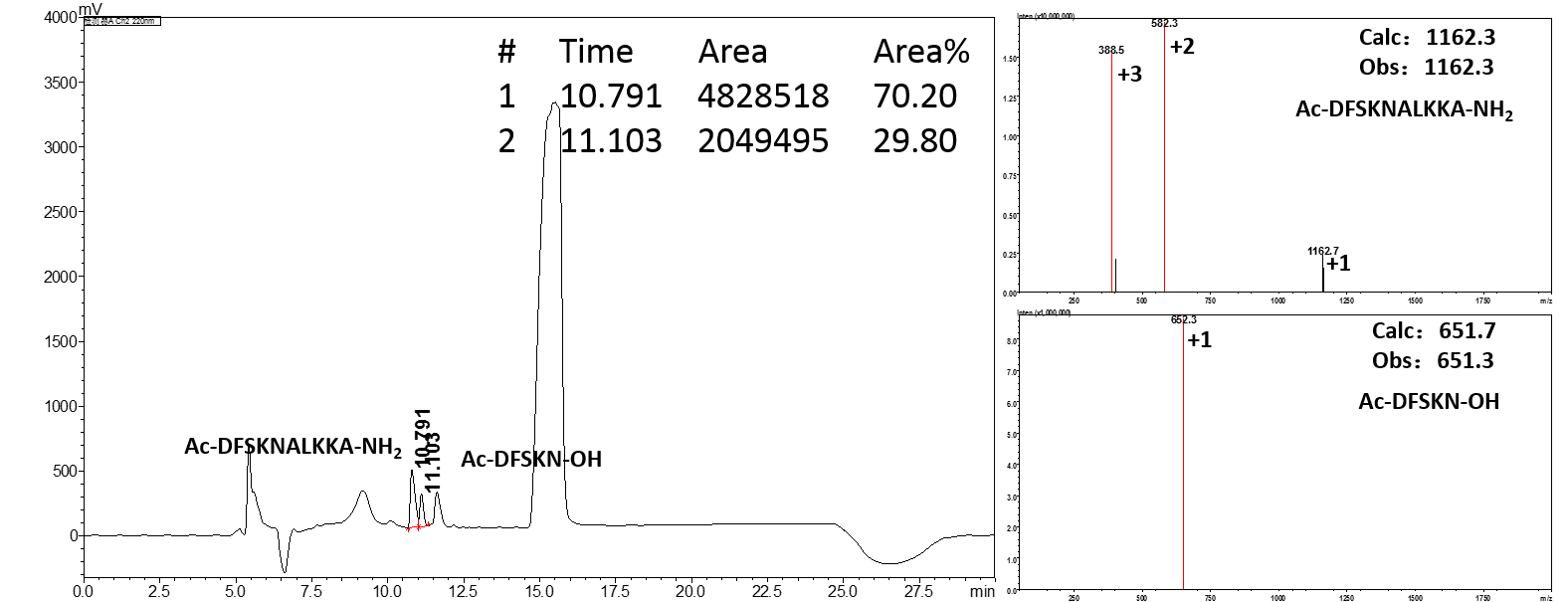

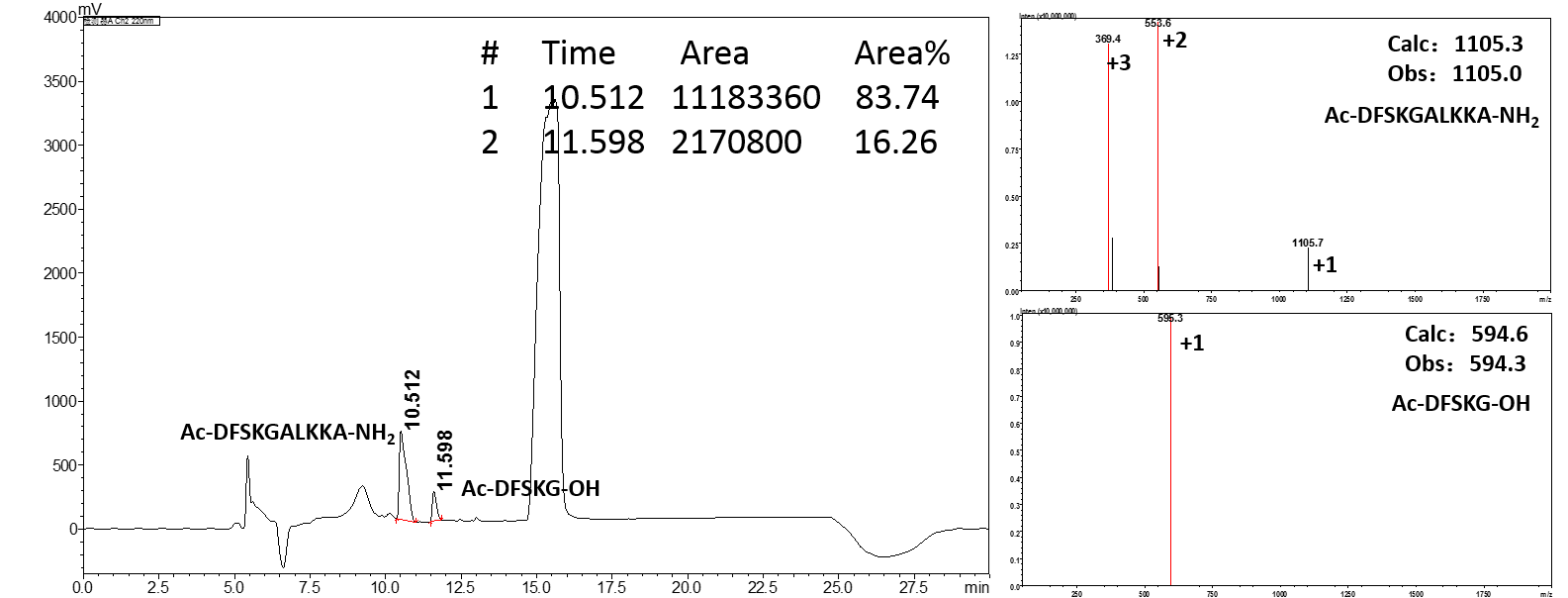

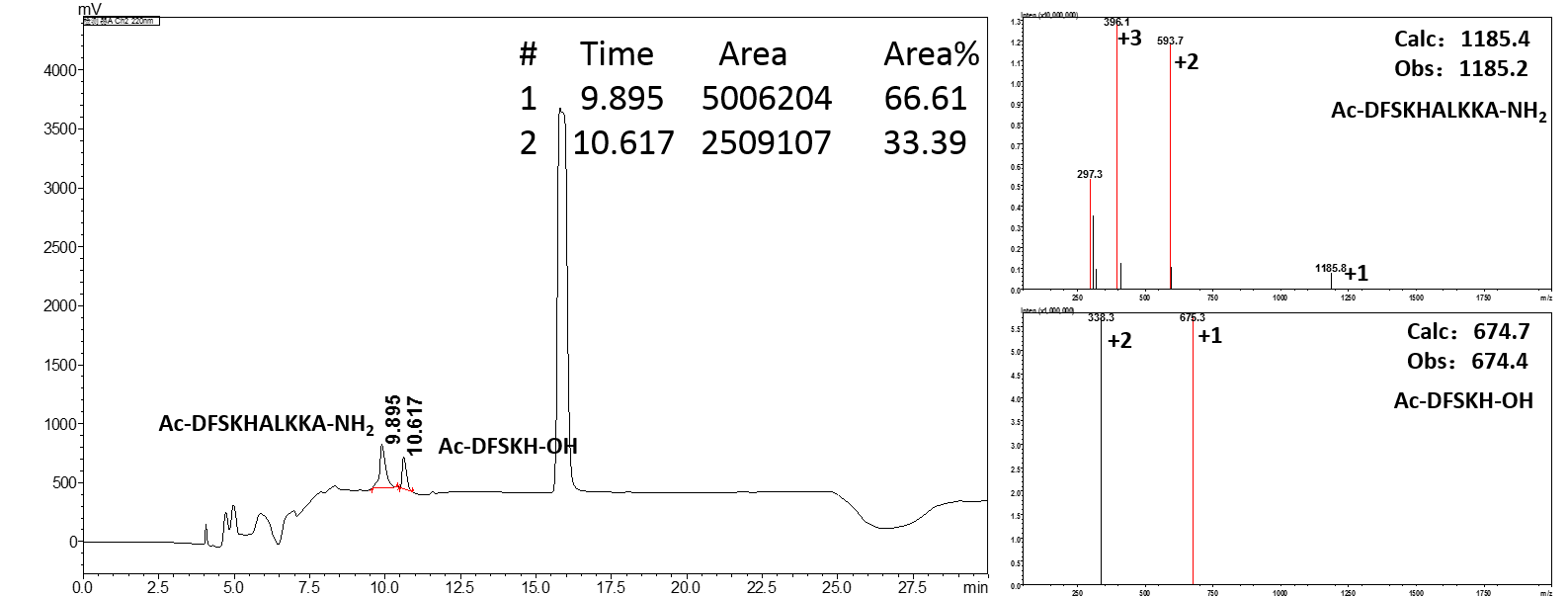


1. **P1 = E, K, T**


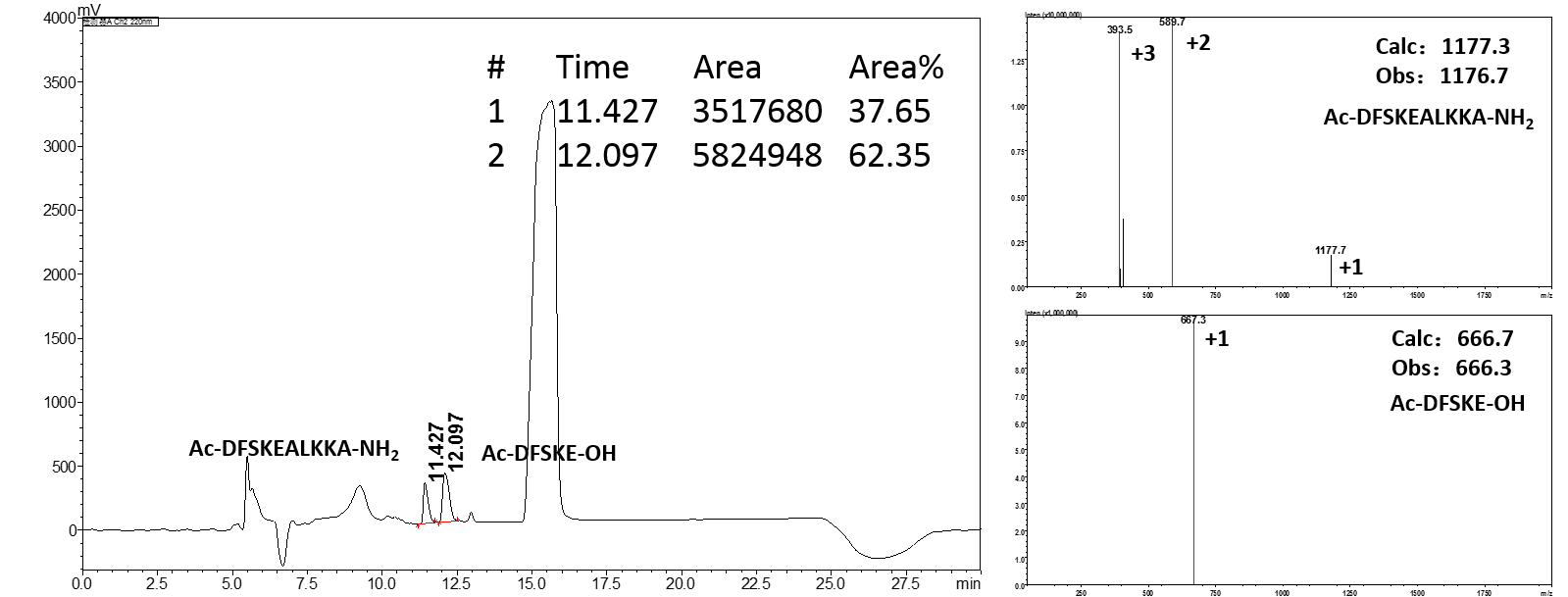

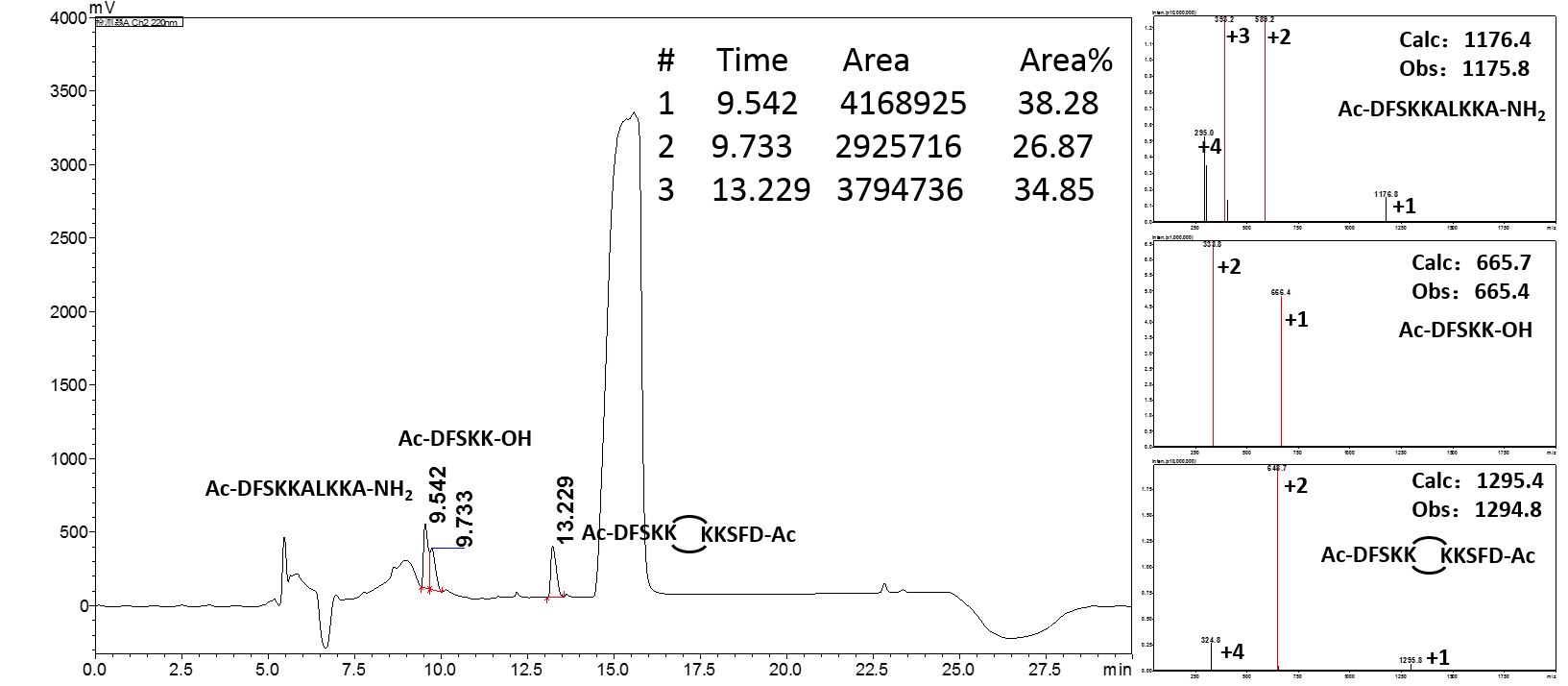

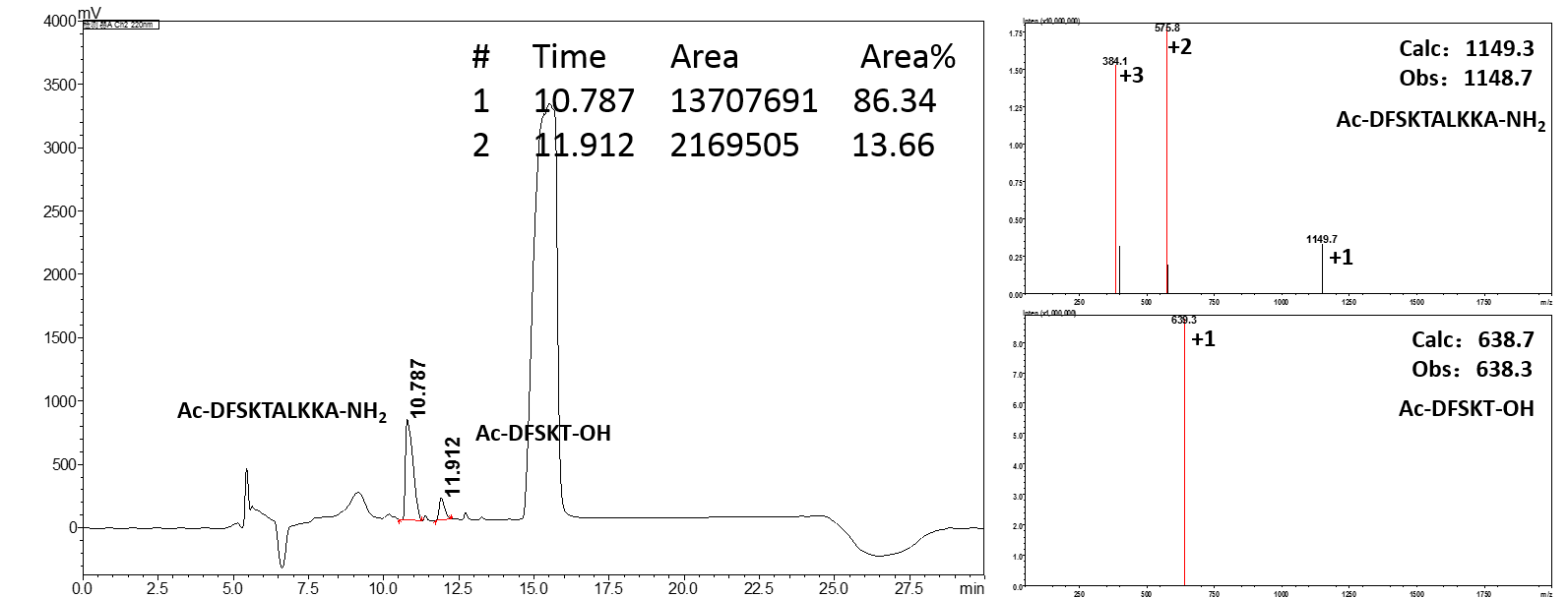


## 2.5 screening of Omniligase-1 P1’ substrates

1. **P1’ = F, I, L, C**


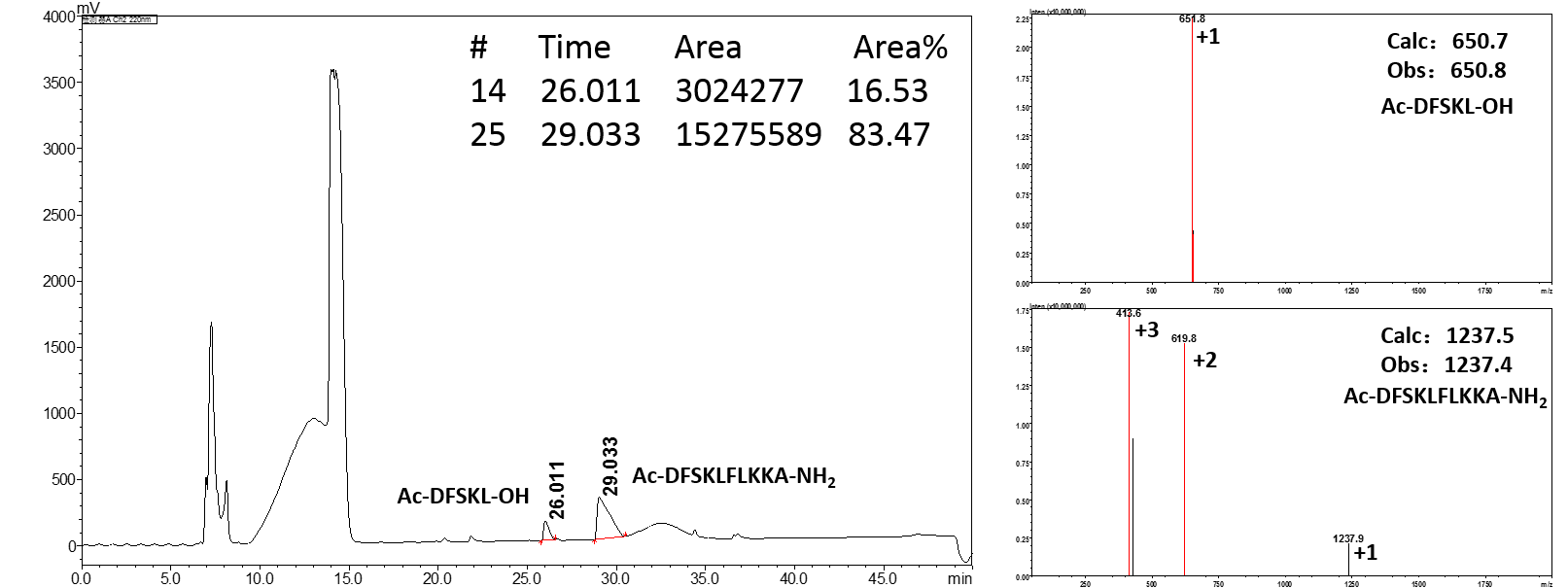

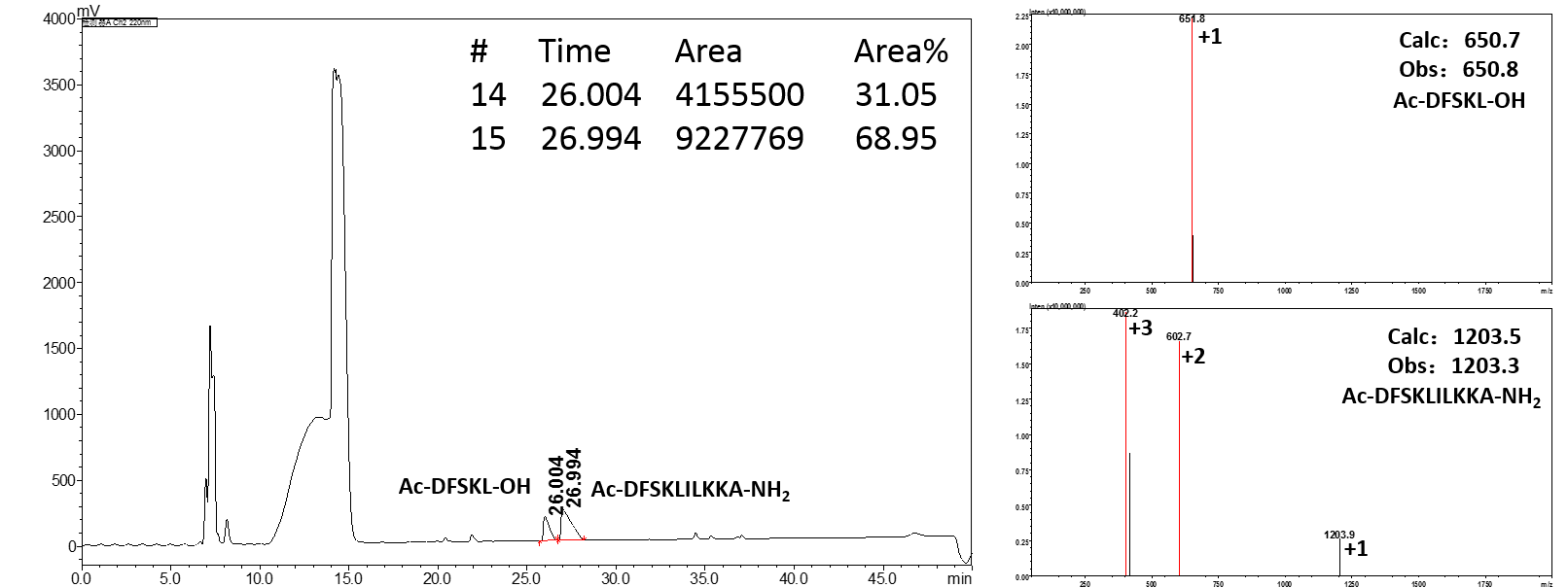

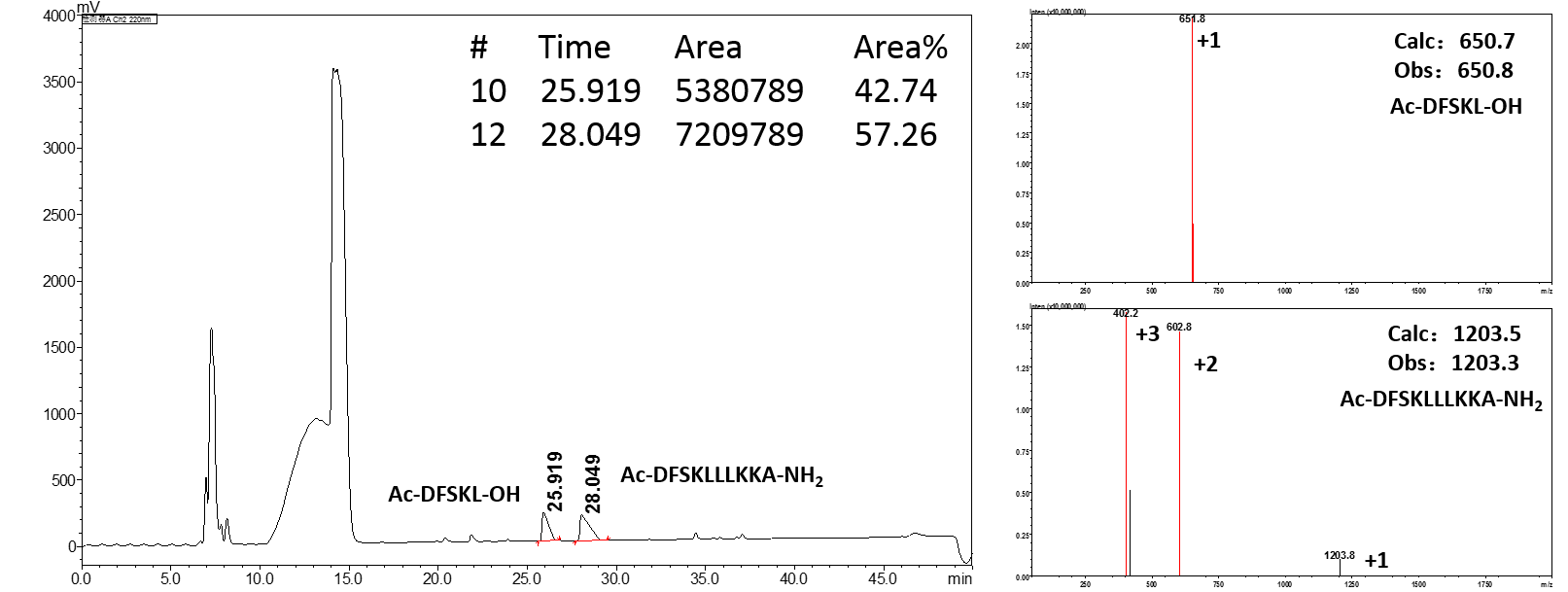

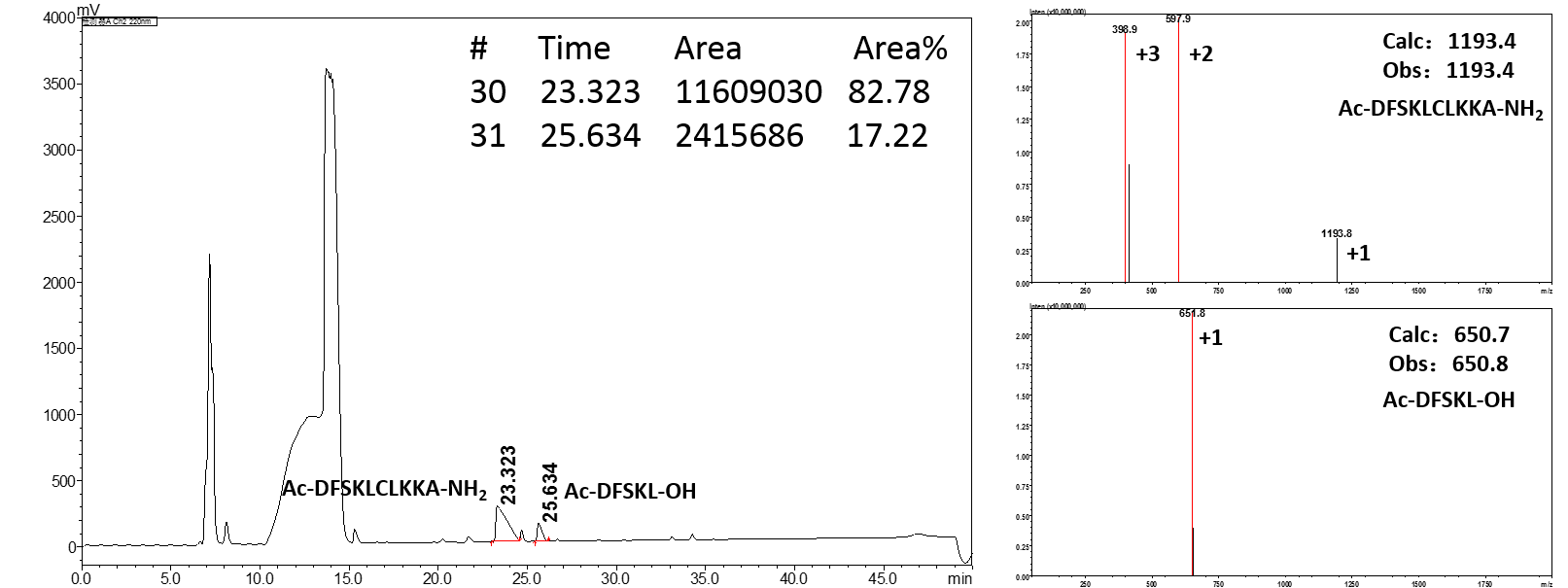


1. **P1’ = W, Y, S, R**


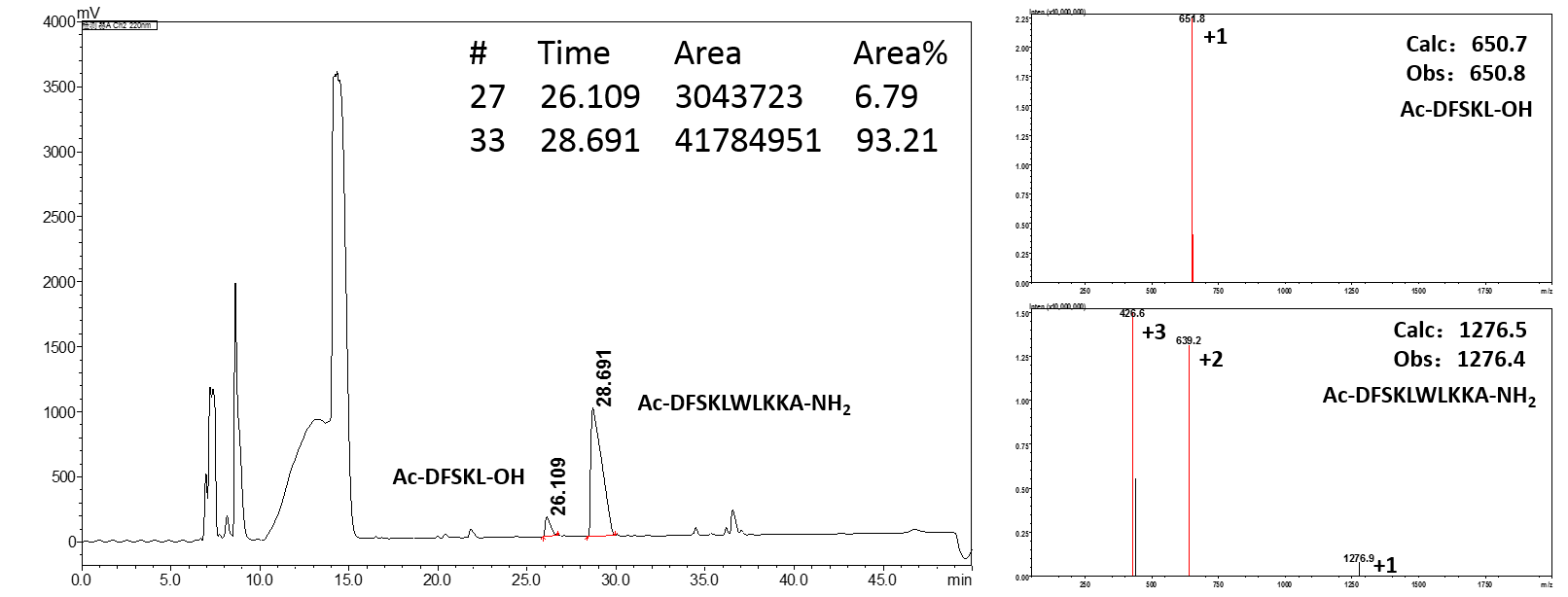

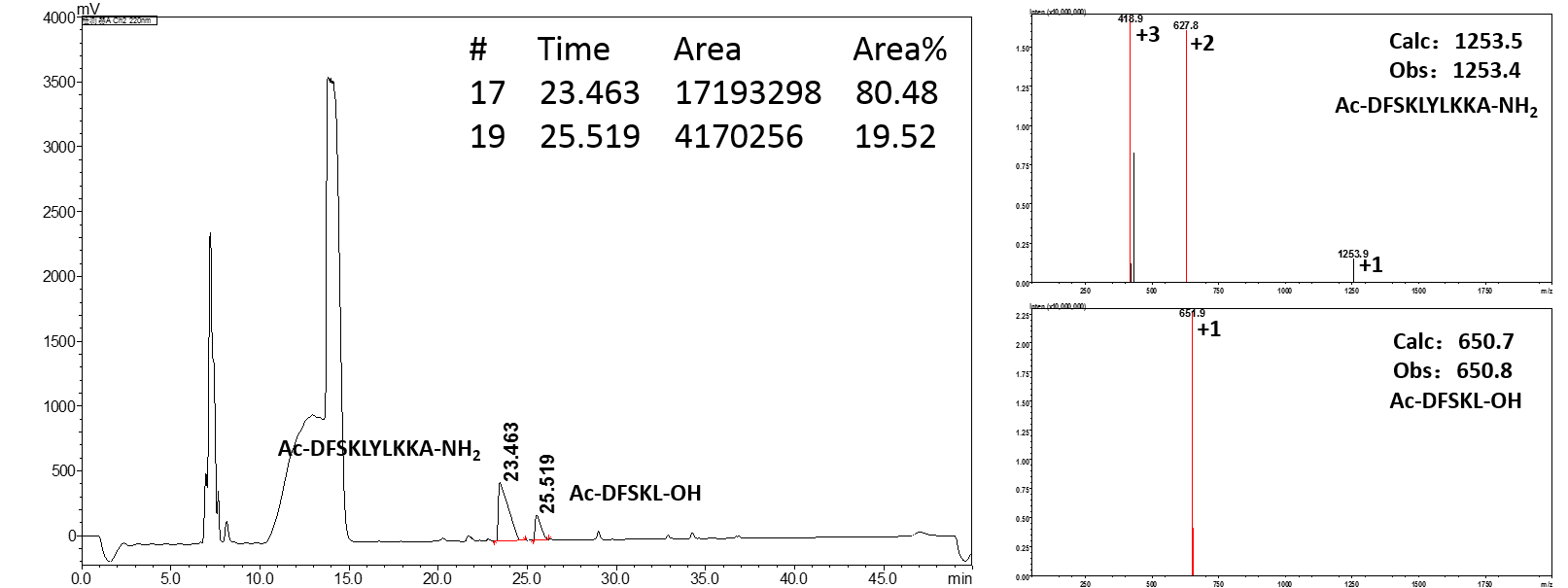

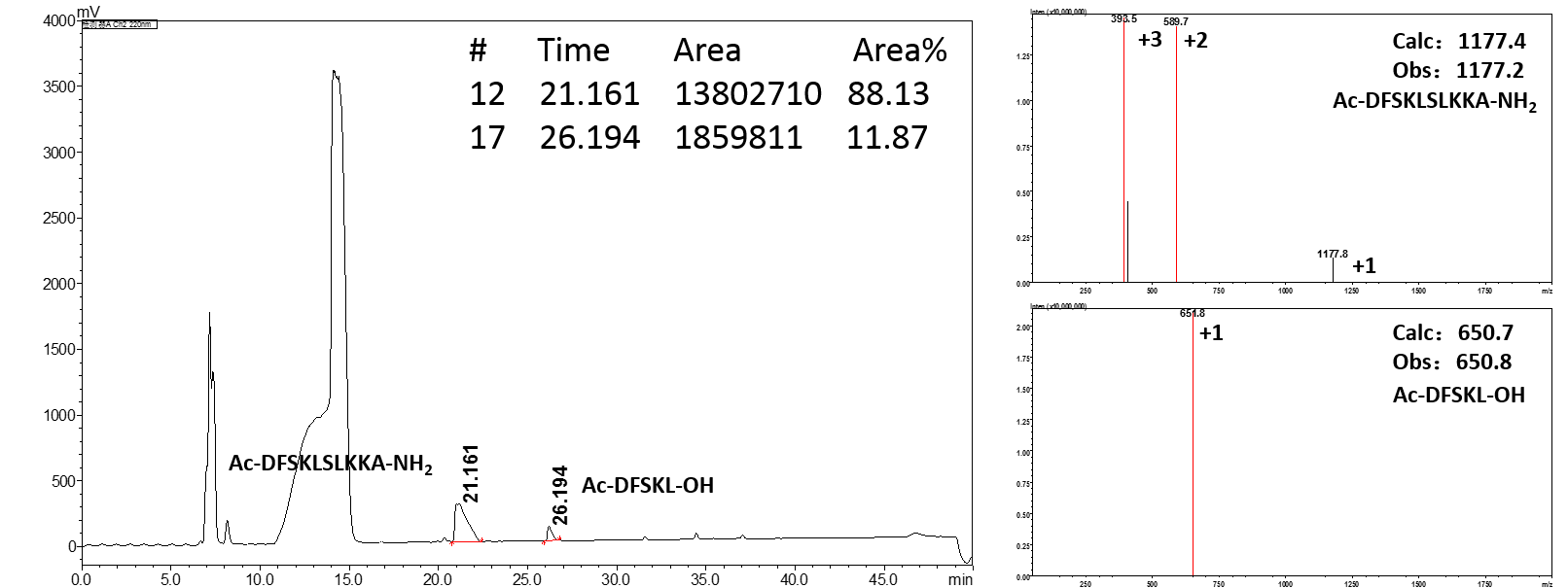

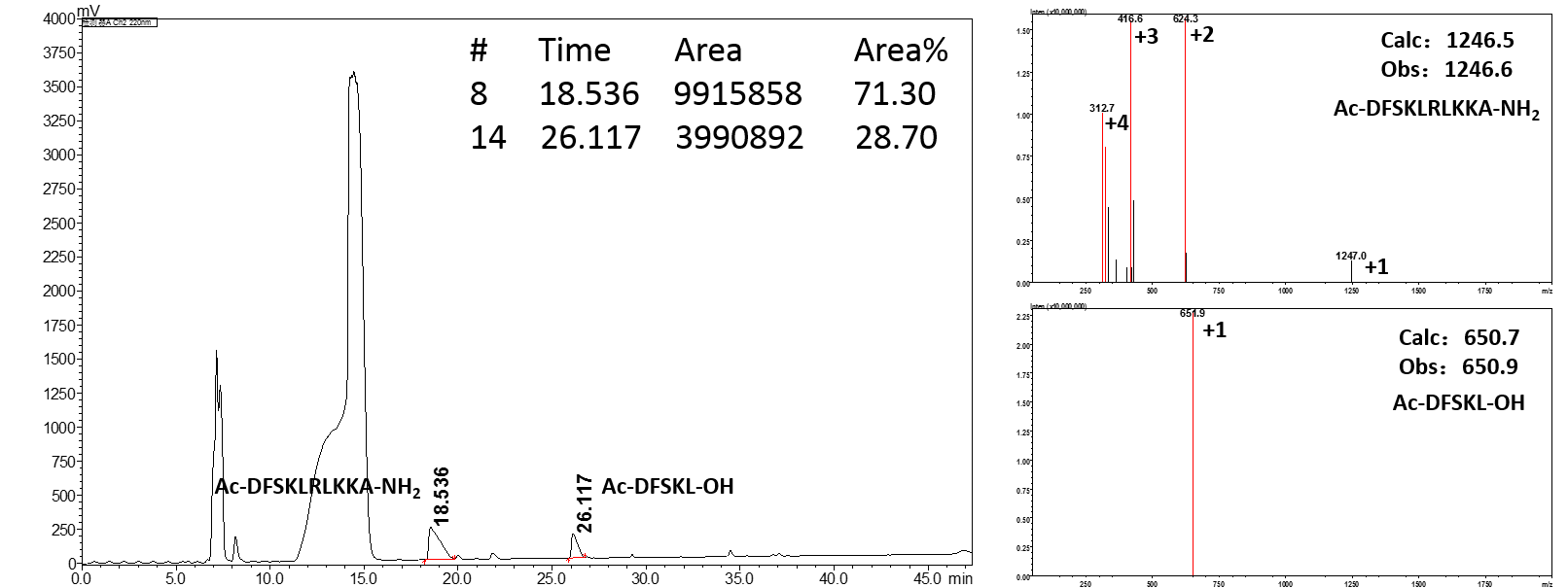


1. **P1’ = Q, D, N, G**

1. **P1’ = H, E, K, P**

1. **P1’ = T, V, M**

## 2.6 screening of Omniligase-1 P2’ substrates

1. **P2’ = F, V, I, M**

1. **P2’ = C, A, W, Y**

1. **P2’ = S, Q, D, N**

1. **P2’ = G, E, P, T**

1. **P2’ = R, H, K**

**DFSKL + ALKKA**

# 3 Substrate screening of PAM12B

## 3.1 screening of PAM12B P2 substrates

1. **P2 = L, Y, V, I, M, F, W, A, R, G**

1. **P2 = E, S, T, H, K, D, C, P, N, Q**

## 3.2 screening of PAM12B P1 substrates

1. **P1 = L, Y, V, I, M, F, W, A, R, G**

1. **P1 = E, S, T, H, K, D, C, Q**

# 4 Screening of PHM, PAL & PAM12B P1 substrates

1. **P1 = A, R**

1. **P1 = D, C**

1. **P1 = E, Q**

1. **P1 = G, H**

1. **P1 = I, L**

1. **P1 = K, M**

1. **P1 = F, S**

1. **P1 = T, W**

1. **P1 = Y, V**

1. **P1 = P, N**
