# Supplementary material for: Traceless enzymatic protein synthesis without ligation sites constraint
Source: Natl Sci Rev. 2021 Aug 24;9(5):nwab158. doi: 10.1093/nsr/nwab158 (PMC9155641; doi:10.1093/nsr/nwab158)
Supplement: nwab158_Supplemental_Files [file nwab158_supplemental_files.zip › Revised_supplementary_methods_and_results-210811.docx]

Supplementary methods and results for

**Traceless enzymatic protein synthesis**

**without ligation sites constraint**

Ruifeng Li^1,2#^, Marcel Schmidt^3#^, Tong Zhu^1,2#^, Xinyu Yang^1,2^, Jing Feng^1,2^, Yu’e Tian^1^, Yinglu Cui^1^, Timo Nuijens^3*^, Bian Wu^1*^

^1^ CAS Key Laboratory of Microbial Physiological and Metabolic Engineering, State Key Laboratory of Microbial Resources, Institute of Microbiology, Chinese Academy of Sciences, China

^2^  University of Chinese Academy of Sciences, China

^3^ Fresenius Kabi iPSUM, I&D Center EnzyPep B.V., the Netherlands

# Equal contribution.

* Email: [timo.nuijens@fresenius-kabi.com](mailto:timo.nuijens@fresenius-kabi.com) or [wub@im.ac.cn](mailto:wub@im.ac.cn)

**Table of contents**

[1 General materials 1](#_Toc69742634)

[1.1 Chemical reagents 1](#_Toc69742635)

[1.2 Synthetic peptides 2](#_Toc69742636)

[1.3 Genes, vectors, and strains 6](#_Toc69742637)

[2 General methods 8](#_Toc69742638)

[2.1 Reagent setup 8](#_Toc69742639)

[2.2 HPLC and LC-MS methods 9](#_Toc69742640)

[2.3 Determination of protein concentration 9](#_Toc69742641)

[2.4 Reaction equipments 9](#_Toc69742642)

[3 Material preparation 10](#_Toc69742643)

[3.1 Enzyme preparation 10](#_Toc69742644)

[3.2 Protein substrate preparation 12](#_Toc69742645)

[3.3 Peptide hydrazide substrate preparation 14](#_Toc69742646)

[4 Supplementary methods and results 16](#_Toc69742647)

[4.1 Screening of alcohols for enzymatic ligation 16](#_Toc69742648)

[4.2 Screening of substrate spectrum of Omniligase-1 18](#_Toc69742649)

[4.3 Screening of substrate spectrum of PAM12B 23](#_Toc69742650)

[4.4 Screening of substrate spectrum of PHM and PAL 25](#_Toc69742651)

[4.5 Cascade reaction of PHM, PAL and PAM12B 27](#_Toc69742652)

[4.6 Conjugation between Ac-DFSKV-G and ALKKA-NH_2_ 29](#_Toc69742653)

[4.7 Total synthesis of exenatide 31](#_Toc69742654)

[4.8 Cyclization of sheep myeloid antimicrobial peptide 34](#_Toc69742655)

[4.9 Semisynthesis of 4-oxalocrotonate tautomerase 35](#_Toc69742656)

[4.10 Total synthesis of 4-oxalocrotonate tautomerase 37](#_Toc69742657)

[4.11 N-terminal modification of FAT10, C4S3 and EGFP 40](#_Toc69742658)

[4.12 Semisynthesis of pSer65-ubiquitin by expressed protein ligation 43](#_Toc69742659)

[4.13 C-terminal modification of NrdH-redoxin by expressed protein ligation 46](#_Toc69742660)

[4.14 Semisynthesis of Lys56-acetylated mitochondrial heat shock protein 10 49](#_Toc69742661)

[References 53](#_Toc69742662)

# 1 General materials

## 1.1 Chemical reagents

Common reagents were all commercially available and used without further purification. Details of the alcohols screened for enzymatic ligation were shown in **Supplementary Table 1**.

**
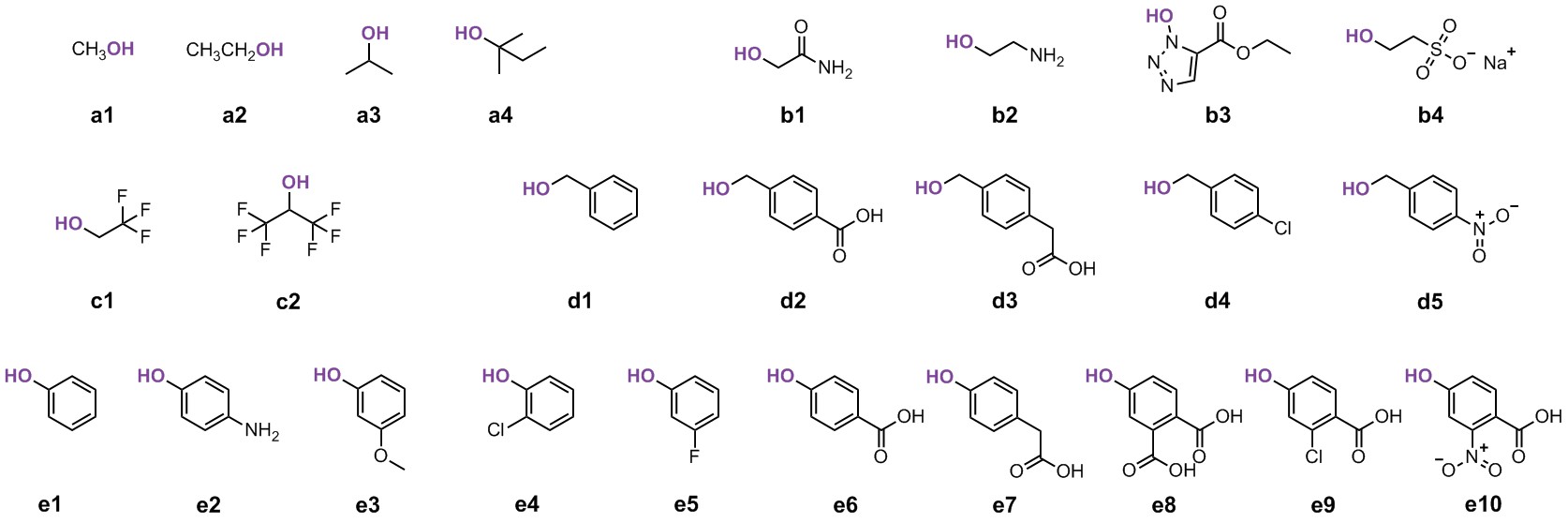
**

**Supplementary Figure 1.** Alcohols screened for enzymatic ligation.

**Supplementary Table 1.** Alcohols screened for enzymatic ligation.

| No. | Name | CAS No. | MW | Purity | Source of manufacturer |
| --- | --- | --- | --- | --- | --- |
| a1 | methanol | 67-56-1 | 32.0 | 99.9% | Fisher Chemical, USA |
| a2 | ethanol | 64-17-5 | 46.1 | AR | Beijing chemical works, China |
| a3 | isopropanol | 67-63-0 | 60.0 | AR | Beijing chemical works, China |
| a4 | 2-methyl-2-butanol | 75-85-4 | 88.2 | 99% | Macklin, Shanghai, China |
| b1 | glycolamide | 598-42-5 | 75.1 | 98% | Sigma aldrich |
| b2 | ethanolamine | 141-43-5 | 61.1 | 99.5% | Aladdin, Shanghai, China |
| b3 | ethyl 1-hydroxy-1H-1,2,3-triazole-4-carboxylate | 137156-41-3 | 157.1 | 98% | Aladdin, Shanghai, China |
| b4 | 4-hydroxyethanesulfonic acid sodium salt | 1562-00-1 | 148.1 | 98% | Macklin, Shanghai, China |
| c1 | 2,2,2-trifluoroethanol | 75-89-8 | 100.0 | 99.5% | Aladdin, Shanghai, China |
| c2 | hexafluoroisopropanol | 920-66-1 | 290.2 | 99.5% | Aladdin, Shanghai, China |
| d1 | benzyl alcohol | 100-51-6 | 108.1 | 99% | Aladdin, Shanghai, China |
| d2 | 4-(hydroxymethyl) phenyl) benzoic acid | 3006-96-0 | 152.2 | 99% | Aladdin, Shanghai, China |
| d3 | 2-(4-(hydroxymethyl)phenyl) acetic acid | 73401-74-8 | 166.2 | 97% | Bidepharm, Shanghai, China |
| d4 | (4-chlorophenyl) methaol | 873-76-7 | 142.6 | 98% | Bidepharm, Shanghai, China |
| d5 | 4-nitrobenzyl alcohol | 619-73-8 | 153.1 | 97% | Bidepharm, Shanghai, China |
| e1 | phenol | 108-95-2 | 94.1 | 98% | Meryer, Shanghai, China |
| e2 | p-aminophenol | 123-30-8 | 109.1 | 98% | Aladdin, Shanghai, China |
| e3 | 3-methoxyphenol | 150-19-6 | 125.1 | 98% | Energy, Shanghai, China |
| e4 | o-chlorophenol | 95-57-8 | 128.6 | 99% | Rhawn, Shanghai, China |
| e5 | 3-fluorophenol | 372-20-3 | 148.1 | 98% | Aladdin, Shanghai, China |
| e6 | 4-hydroxybenzoic acid | 99-96-7 | 138.1 | 99% | D&B, Shanghai, China |
| e7 | 4-hydroxyphenylacetic | 156-38-7 | 152.2 | 98% | Ouhe, Beijing, China |
| e8 | 4-hydroxyphthalic acid | 610-35-5 | 182.1 | 97% | Haohong, Shanghai, China |
| e9 | 2-chloro-4-hyroxybenzoic acid hydrate | 440123-65-9 | 172.6 | 97% | Accela, San Diego, USA |
| e10 | 4-hydroxy-2-nitrobenzoic acid | 74230-08-3 | 184.1 | 95% | Bidepharm, Shanghai, China |

## 1.2 Synthetic peptides

Synthetic peptides were all purchased from GL Biochem (Shanghai, China) except for No.179, 187, 190, 191 (purchased from Anhui Guoping Pharmaceutical, Hefei, China) and No.178 (kindly provided by Prof. L. Liu). Peptide hydrazides were synthesized through a protocol [1] from Liu group.

**Supplementary Table 2.** Synthetic peptides.

| No. | Sequence | MW | Purity |
| --- | --- | --- | --- |
| 1 | Ac-DFSKE-N_2_H_3_ | 680.7 | >95% |
| 2 | Ac-DFSKT-N_2_H_3_ | 652.7 | >95% |
| 3 | Ac-DFSKK-N_2_H_3_ | 679.8 | >95% |
| 4 | Ac-DFSKQ-N_2_H_3_ | 679.7 | >95% |
| 5 | Ac-DFSKS-N_2_H_3_ | 638.7 | >95% |
| 6 | Ac-DFSKA-N_2_H_3_ | 622.7 | >95% |
| 7 | Ac-DFSKI-N_2_H_3_ | 664.8 | >95% |
| 8 | Ac-DFSKW-N_2_H_3_ | 737.8 | >95% |
| 9 | Ac-DFSKH-N_2_H_3_ | 688.8 | >95% |
| 10 | Ac-DFSKP-N_2_H_3_ | 648.7 | >95% |
| 11 | Ac-DFSKM-N_2_H_3_ | 682.8 | >95% |
| 12 | Ac-DFSKG-N_2_H_3_ | 608.7 | >95% |
| 13 | Ac-DFSKV-N_2_H_3_ | 650.7 | >95% |
| 14 | Ac-DFSKR-N_2_H_3_ | 707.8 | >95% |
| 15 | Ac-DFSKY-N_2_H_3_ | 714.8 | >95% |
| 16 | Ac-DFSKD-N_2_H_3_ | 666.7 | >95% |
| 17 | Ac-DFSKN-N_2_H_3_ | 665.7 | >95% |
| 18 | Ac-DFSKC-N_2_H_3_ | 654.7 | >95% |
| 19 | Ac-DFSKF-N_2_H_3_ | 698.8 | >95% |
| 20 | Ac-DFSKL-N_2_H_3_ | 664.8 | >95% |
| 21 | Ac-DFSEL-N_2_H_3_ | 665.7 | >95% |
| 22 | Ac-DFSTL-N_2_H_3_ | 637.7 | >95% |
| 23 | Ac-DFSQL-N_2_H_3_ | 664.7 | >95% |
| 24 | Ac-DFSSL-N_2_H_3_ | 623.7 | >95% |
| 25 | Ac-DFSAL-N_2_H_3_ | 607.7 | >95% |
| 26 | Ac-DFSIL-N_2_H_3_ | 649.8 | >95% |
| 27 | Ac-DFSWL-N_2_H_3_ | 722.8 | >95% |
| 28 | Ac-DFSHL-N_2_H_3_ | 673.7 | >95% |
| 29 | Ac-DFSPL-N_2_H_3_ | 633.7 | >95% |
| 30 | Ac-DFSML-N_2_H_3_ | 667.8 | >95% |
| 31 | Ac-DFSGL-N_2_H_3_ | 593.6 | >95% |
| 32 | Ac-DFSVL-N_2_H_3_ | 635.7 | >95% |
| 33 | Ac-DFSRL-N_2_H_3_ | 692.8 | >95% |
| 34 | Ac-DFSYL-N_2_H_3_ | 699.8 | >95% |
| 35 | Ac-DFSDL-N_2_H_3_ | 651.7 | >95% |
| 36 | Ac-DFSNL-N_2_H_3_ | 650.7 | >95% |
| 37 | Ac-DFSCL-N_2_H_3_ | 639.7 | >95% |
| 38 | Ac-DFSFL-N_2_H_3_ | 683.8 | >95% |
| 39 | Ac-DFSLL-N_2_H_3_ | 649.8 | >95% |
| 40 | Ac-DFEKL-N_2_H_3_ | 706.8 | >95% |
| 41 | Ac-DFTKL-N_2_H_3_ | 678.8 | >95% |
| 42 | Ac-DFKKL-N_2_H_3_ | 705.9 | >95% |
| 43 | Ac-DFQKL-N_2_H_3_ | 705.8 | >95% |
| 44 | Ac-DFAKL-N_2_H_3_ | 648.8 | >95% |
| 45 | Ac-DFIKL-N_2_H_3_ | 690.9 | >95% |
| 46 | Ac-DFWKL-N_2_H_3_ | 763.9 | >95% |
| 47 | Ac-DFHKL-N_2_H_3_ | 714.8 | >95% |
| 48 | Ac-DFPKL-N_2_H_3_ | 674.8 | >95% |
| 49 | Ac-DFMKL-N_2_H_3_ | 708.9 | >95% |
| 50 | Ac-DFGKL-N_2_H_3_ | 634.7 | >95% |
| 51 | Ac-DFVKL-N_2_H_3_ | 676.8 | >95% |
| 52 | Ac-DFRKL-N_2_H_3_ | 733.9 | >95% |
| 53 | Ac-DFYKL-N_2_H_3_ | 740.9 | >95% |
| 54 | Ac-DFDKL-N_2_H_3_ | 692.8 | >95% |
| 55 | Ac-DFNKL-N_2_H_3_ | 691.8 | >95% |
| 56 | Ac-DFCKL-N_2_H_3_ | 680.8 | >95% |
| 57 | Ac-DFFKL-N_2_H_3_ | 724.9 | >95% |
| 58 | Ac-DFLKL-N_2_H_3_ | 690.9 | >95% |
| 59 | Ac-DESKL-N_2_H_3_ | 646.7 | >95% |
| 60 | Ac-DTSKL-N_2_H_3_ | 618.7 | >95% |
| 61 | Ac-DKSKL-N_2_H_3_ | 645.8 | >95% |
| 62 | Ac-DQSKL-N_2_H_3_ | 645.7 | >95% |
| 63 | Ac-DSSKL-N_2_H_3_ | 604.7 | >95% |
| 64 | Ac-DASKL-N_2_H_3_ | 588.7 | >95% |
| 65 | Ac-DISKL-N_2_H_3_ | 630.8 | >95% |
| 66 | Ac-DWSKL-N_2_H_3_ | 703.8 | >95% |
| 67 | Ac-DHSKL-N_2_H_3_ | 654.7 | >95% |
| 68 | Ac-DPSKL-N_2_H_3_ | 614.7 | >95% |
| 69 | Ac-DMSKL-N_2_H_3_ | 648.8 | >95% |
| 70 | Ac-DGSKL-N_2_H_3_ | 574.6 | >95% |
| 71 | Ac-DVSKL-N_2_H_3_ | 616.7 | >95% |
| 72 | Ac-DRSKL-N_2_H_3_ | 673.8 | >95% |
| 73 | Ac-DYSKL-N_2_H_3_ | 680.8 | >95% |
| 74 | Ac-DDSKL-N_2_H_3_ | 632.7 | >95% |
| 75 | Ac-DNSKL-N_2_H_3_ | 631.7 | >95% |
| 76 | Ac-DCSKL-N_2_H_3_ | 620.7 | >95% |
| 77 | Ac-DLSKL-N_2_H_3_ | 630.8 | >95% |
| 78 | ELKKA-NH_2_ | 586.7 | >95% |
| 79 | TLKKA-NH_2_ | 558.7 | >95% |
| 80 | KLKKA-NH_2_ | 585.8 | >95% |
| 81 | QLKKA-NH_2_ | 585.8 | >95% |
| 82 | SLKKA-NH_2_ | 544.7 | >95% |
| 83 | ALKKA-NH_2_ | 528.7 | >95% |
| 84 | ILKKA-NH_2_ | 570.8 | >95% |
| 85 | WLKKA-NH_2_ | 643.8 | >95% |
| 86 | HLKKA-NH_2_ | 594.8 | >95% |
| 87 | PLKKA-NH_2_ | 554.7 | >95% |
| 88 | MLKKA-NH_2_ | 588.8 | >95% |
| 89 | GLKKA-NH_2_ | 514.7 | >95% |
| 90 | VLKKA-NH_2_ | 556.8 | >95% |
| 91 | RLKKA-NH_2_ | 613.8 | >95% |
| 92 | YLKKA-NH_2_ | 620.8 | >95% |
| 93 | DLKKA-NH_2_ | 572.7 | >95% |
| 94 | NLKKA-NH_2_ | 571.7 | >95% |
| 95 | CLKKA-NH_2_ | 560.8 | >95% |
| 96 | FLKKA-NH_2_ | 604.8 | >95% |
| 97 | LLKKA-NH_2_ | 570.8 | >95% |
| 98 | AEKKA-NH_2_ | 544.7 | >95% |
| 99 | ATKKA-NH_2_ | 516.7 | >95% |
| 100 | AKKKA-NH_2_ | 543.7 | >95% |
| 101 | AQKKA-NH_2_ | 543.7 | >95% |
| 102 | ASKKA-NH_2_ | 502.6 | >95% |
| 103 | AAKKA-NH_2_ | 486.6 | >95% |
| 104 | AIKKA-NH_2_ | 528.7 | >95% |
| 105 | AWKKA-NH_2_ | 601.8 | >95% |
| 106 | AHKKA-NH_2_ | 552.7 | >95% |
| 107 | APKKA-NH_2_ | 512.7 | >95% |
| 108 | AMKKA-NH_2_ | 546.7 | >95% |
| 109 | AGKKA-NH_2_ | 472.6 | >95% |
| 110 | AVKKA-NH_2_ | 514.7 | >95% |
| 111 | ARKKA-NH_2_ | 571.7 | >95% |
| 112 | AYKKA-NH_2_ | 578.7 | >95% |
| 113 | ADKKA-NH_2_ | 530.6 | >95% |
| 114 | ANKKA-NH_2_ | 529.6 | >95% |
| 115 | ACKKA-NH_2_ | 518.7 | >95% |
| 116 | AFKKA-NH_2_ | 562.7 | >95% |
| 117 | Ac-DFSKA-NH_2_ | 607.7 | >95% |
| 118 | Ac-DFSKR-NH_2_ | 692.8 | >95% |
| 119 | Ac-DFSKN-NH_2_ | 650.7 | >95% |
| 120 | Ac-DFSKC-NH_2_ | 639.7 | >95% |
| 121 | Ac-DFSKE-NH_2_ | 665.7 | >95% |
| 122 | Ac-DFSKQ-NH_2_ | 664.7 | >95% |
| 123 | Ac-DFSKG-NH_2_ | 593.6 | >95% |
| 124 | Ac-DFSKH-NH_2_ | 673.7 | >95% |
| 125 | Ac-DFSKI-NH_2_ | 649.8 | >95% |
| 126 | Ac-DFSKK-NH_2_ | 664.8 | >95% |
| 127 | Ac-DFSKM-NH_2_ | 667.8 | >95% |
| 128 | Ac-DFSKF-NH_2_ | 683.8 | >95% |
| 129 | Ac-DFSKP-NH_2_ | 633.7 | >95% |
| 130 | Ac-DFSKS-NH_2_ | 623.7 | >95% |
| 131 | Ac-DFSKT-NH_2_ | 637.7 | >95% |
| 132 | Ac-DFSKW-NH_2_ | 722.8 | >95% |
| 133 | Ac-DFSKY-NH_2_ | 699.8 | >95% |
| 134 | Ac-DFSKV-NH_2_ | 635.7 | >95% |
| 135 | Ac-DFSKD-NH_2_ | 651.7 | >95% |
| 136 | Ac-DFSKL-NH_2_ | 649.8 | >95% |
| 137 | Ac-DFSAL-NH_2_ | 592.7 | >95% |
| 138 | Ac-DFSRL-NH_2_ | 677.8 | >95% |
| 139 | Ac-DFSNL-NH_2_ | 635.7 | >95% |
| 140 | Ac-DFSDL-NH_2_ | 636.7 | >95% |
| 141 | Ac-DFSCL-NH_2_ | 624.7 | >95% |
| 142 | Ac-DFSEL-NH_2_ | 650.7 | >95% |
| 143 | Ac-DFSQL-NH_2_ | 649.7 | >95% |
| 144 | Ac-DFSGL-NH_2_ | 578.6 | >95% |
| 145 | Ac-DFSHL-NH_2_ | 658.7 | >95% |
| 146 | Ac-DFSIL-NH_2_ | 634.7 | >95% |
| 147 | Ac-DFSLL-NH_2_ | 634.7 | >95% |
| 148 | Ac-DFSML-NH_2_ | 652.8 | >95% |
| 149 | Ac-DFSFL-NH_2_ | 668.8 | >95% |
| 150 | Ac-DFSPL-NH_2_ | 618.7 | >95% |
| 151 | Ac-DFSSL-NH_2_ | 608.7 | >95% |
| 152 | Ac-DFSTL-NH_2_ | 622.7 | >95% |
| 153 | Ac-DFSWL-NH_2_ | 707.8 | >95% |
| 154 | Ac-DFSYL-NH_2_ | 684.8 | >95% |
| 155 | Ac-DFSVL-NH_2_ | 620.7 | >95% |
| 156 | DLSYAG | 624.7 | >95% |
| 157 | DLSYRG | 709.8 | >95% |
| 158 | DLSYNG | 667.7 | >95% |
| 159 | DLSYDG | 668.7 | >95% |
| 160 | DLSYCG | 656.8 | >95% |
| 161 | DLSYEG | 682.7 | >95% |
| 162 | DLSYQG | 681.7 | >95% |
| 163 | DLSYGG | 610.7 | >95% |
| 164 | DLSYHG | 690.8 | >95% |
| 165 | DLSYIG | 666.8 | >95% |
| 166 | DLSYLG | 666.8 | >95% |
| 167 | DLSYKG | 681.8 | >95% |
| 168 | DLSYMG | 684.8 | >95% |
| 169 | DLSYFG | 700.8 | >95% |
| 170 | DLSYPG | 650.7 | >95% |
| 171 | DLSYSG | 640.7 | >95% |
| 172 | DLSYTG | 654.7 | >95% |
| 173 | DLSYWG | 739.8 | >95% |
| 174 | DLSYYG | 716.8 | >95% |
| 175 | DLSYVG | 652.7 | >95% |
| 176 | HGEGTFTS-N_2_H_3_ | 858.8 | >95% |
| 177 | DLSKQMEEEAVRL-NH_2_ | 1546.7 | >95% |
| 178 | FIEWLKNGGPSSGAPPPS-NH_2_ | 1840.0 | >95% |
| 179 | PIAQIHILEGRSDEQKETLIREVSEAI-NH_2_ | 3074.5 | >95% |
| 180 | VLRIIRIAGRGLRRLGRKIAHGVKKYGPT-NH_2_ | 3255.0 | >95% |
| 181 | Biotin-FSKL-N_2_H_3_ | 733.4 | >95% |
| 182 | FITC-FSKL-N_2_H_3_ | 1009.6 | >95% |
| 183 | ALKKAK-Biotin | 884.1 | >95% |
| 184 | ALKKAK-FITC | 1047.2 | >95% |
| 185 | Ac-DFSKVG | 693.8 | >95% |
| 186 | NIQKES-(pS)-TLHLVLRLRGG | 2014.3 | >95% |
| 187 | AVGSGSKG-(^Ac^K)-GGEIQPV-NH_2_ | 1511.4 | >95% |
| 188 | SRSLDDPLTSV-NH_2_ | 1188.3 | >95% |
| 189 | RVIITEMAKGHFGIGGELASKVRR | 2626.1 | >95% |
| 190 | Ac-DFSK-(D)-F-N_2_H_3_ | 698.8 | >95% |
| 191 | Ac-DFSK-(D)-L-N_2_H_3_ | 664.8 | >95% |

## 1.3 Genes, vectors, and strains

Genes of the enzymes and recombinant proteins were synthesized by General Biosystems (Anhui, China) with sequence optimization for expression in *Escherichia coli* (except for PHM). Expression vectors used in this study were shown in **Supplementary Table 3**. Expression strain *E. coli* BL21 (DE3) was purchased from TIANGEN Biotech (Beijing, China).

**Supplementary Table 3.** Vectors used in this study.

| Vectors | Description | Source |
| --- | --- | --- |
| pET-21a(+) | T7 promoter, pBR322 ori, Amp^R^ | Commercially available |
| pET-28a(+) | T7 promoter, pBR322 ori, Kan^R^ | Commercially available |
| pET-28b(+) | T7 promoter, pBR322 ori, Kan^R^ | Commercially available |
| pCAGGS | AG promoter, SV40 ori & pMB1 ori, Amp^R^ | A gift from Prof. J. H. Yan |
| pET21a-PAM12B | pET-21a(+) carrying PAM12B | [1] |
| pET28a-PAL | pET-28a(+) carrying PAL | This study |
| pCAGGS-PHM | pCAGGS carrying PHM | This study |
| pET21a-FAT10 | pET-21a(+) carrying FAT10 | This study |
| pET21a-C4S3 | pET-21a(+) carrying C4S3 | This study |
| pET21a-AL-EGFP | pET-21a(+) carrying Ala-Leu-EGFP | This study |
| pET21a-4-OT | pET-21a(+) carrying 4-OT | This study |
| pETSUMO | pET-28b(+) carrying SUMO | This study |
| pETSUMO-4-OT(28-62) | pETSUMO carrying 4-OT(28-62) | This study |
| pETSUMO-NrdH-G | pETSUMO carrying NrdH-Gly | This study |
| pETSUMO-Ub(1-59)-G | pETSUMO carrying Ub(1-59)-Gly | This study |
| pETSUMO-mHSP10(2-47)-G | pETSUMO carrying mHSP10(2-47)-Gly | This study |
| pETSUMO-mHSP10(64-102) | pETSUMO carrying mHSP10(64-102) | This study |

# 2 General methods

## 2.1 Reagent setup

1. **12 M HCl solution:**

Commercial reagent (hydrochloric acid).

1. **6 M HCl solution:**

50 mL of 12 M HCl solution was mixed with 50 mL of deionized H_2_O.

1. **1 M HCl solution:**

10 mL of 12 M HCl solution was mixed with 110 mL of deionized H_2_O.

1. **6 M NaOH solution:**

24.00 g of NaOH was dissolved in 80 mL of deionized H_2_O and the volume was adjusted to 100 mL with deionized H_2_O.

1. **1 M NaOH solution:**

10 mL of 6 M NaOH solution was mixed with 50 mL of deionized H_2_O.

1. **0.2 M Phosphate buffer solution containing 6 M Gn·HCl (pH 3.0):**

1.56 g of NaH_2_PO_4_·2H_2_O and 28.65 g of guanidine hydrochloride (Gn·HCl) were dissolved in 40 mL of deionized H_2_O, then the pH was adjusted to 3.0~3.1 with 6 M HCl solution. The volume was adjusted to 50 mL with deionized H_2_O.

1. **0.2 M Phosphate buffer solution containing 6 M Gn·HCl (pH 7.0):**

1.56 g of NaH_2_PO_4_·2H_2_O and 28.65 g of Gn·HCl were dissolved in 40 mL of deionized H_2_O, then the pH was adjusted to 7.0~7.1 with 6 M HCl solution. The volume was adjusted to 50 mL with deionized H_2_O.

1. **0.05 M Phosphate buffer solution (pH 7.5):**

0.34 g of KH_2_PO_4_ was dissolved in 40 mL of deionized H_2_O, then the pH was adjusted to 7.5~7.6 with 1 M NaOH solution. The volume was adjusted to 50 mL with deionized H_2_O.

1. **5 M NaNO_2_ solution:**

173 mg of NaNO_2_ was dissolved in deionized H_2_O and the volume was adjusted to 500 μL (Freshly prepare before use).

1. **0.5 M NaNO_2_ solution:**

35 mg of NaNO_2_ was dissolved in 1000 μL of deionized H_2_O (Freshly prepare before use).

1. **3 M HBA solution:**

41 mg of 4-hydroxybenzoic acid (HBA) was dissolved in 50 μL of 6 M NaOH solution and the volume was adjusted to 100 μL with deionized H_2_O (Freshly prepare before use).

1. **2 M HBA solution:**

41 mg of HBA was dissolved in 50 μL of 6 M NaOH solution and the volume was adjusted to 150 μL with deionized H_2_O (Freshly prepare before use).

1. **20 M N_2_H_4_ solution:**

Commercial reagent (hydrazine hydrate).

1. **5 M N_2_H_4_ solution:**

10 mL of 20 M N_2_H_4_ solution was mixed with 30 mL of deionized H_2_O.

1. **0.2 M sodium ascorbate solution:**

40 mg of sodium ascorbate was dissolved in 1000 μL of deionized H_2_O (Freshly prepare before use).

## 2.2 HPLC and LC-MS methods

1. **Instruments**

LC-MS analysis was performed with two set of instruments. Agilent (Santa Clara, USA) set: 1200 Series + G1946D. Shimadzu (Kyoto, Japan) set: LC-30AD + LCMS-2020 + SPD-20A.

Preparative HPLC was performed on Shimadzu set: LC-20AP + SPD-M20A.

1. **Analytical LC-MS methods**

**General method A:** column: C4 reversed-phase column (4.6×250 mm, pore size: 300 Å, particle size: 5 μm, Nanochrom, Suzhou, China); temperature: 25°C; eluent A: 0.1% formic acid in water, eluent B: acetonitrile, gradient from 5% to 65% CH_3_CN in 30 min (final concentration of CH_3_CN depended on the peptides or reactions); flow rate: 1 mL·min^−1^; detection: UV at 220 nm; ESI-MS: positive scan mode (m/z 200~2000).

**General method B:** column: C18 reversed-phase column (4.6×250 mm, pore size: 100 Å, particle size: 5 μm, Nanochrom, Suzhou, China); temperature: 25°C; eluent A: 0.1% formic acid in water, eluent B: acetonitrile, gradient from 5% to 65% CH_3_CN in 30 min (final concentration of CH_3_CN depended on the peptides or reactions); flow rate: 1 mL·min^−1^; detection: UV at 220 nm; ESI-MS: positive scan mode (m/z 200~2000).

**General method C:** column: C4 reversed-phase column (4.6×250mm, pore size: 300 Å, particle size: 5 μm, Nanochrom, Suzhou, China); temperature: 25°C; eluent A: 0.1% formic acid in water, eluent B: acetonitrile, isocratic 10~25% CH_3_CN for 10~20 min (depending on the peptides or reactions); flow rate: 1 mL·min^−1^; detection: UV at 220 nm; ESI-MS: positive scan mode (m/z 200~2000).

1. **Preparative HPLC method**

**General method D:** column: C4 reversed-phase column (21.2×250mm, pore size: 300 Å, particle size: 5 μm, Nanochrom, Suzhou, China); temperature: 25°C; eluent A: 0.1% formic acid in water, eluent B: acetonitrile, gradient from 5% to 65% CH_3_CN in 60 min; flow rate: 10 mL·min^−1^; detection: UV at 220 nm.

## 2.3 Determination of protein concentration

Concentrations of the enzymes and the recombinant proteins were determined using BCA assay with bovine serum albumin as the standard (Solarbio, Beijing, China).

## 2.4 Reaction equipments

Amidation reactions (37°C) were performed in static water bath (reaction volume < 100 μL) or air bath shaker (reaction volume ≥ 100 μL). Esterification reactions (-15°C) were performed in a low-temperature reactor with anhydrous alcohol as the heat transfer medium. For ligation reactions, pH value was determined with pH indicator paper (pH 5.5~9.0, Aladdin, Shanghai, China).

# 3 Material preparation

## 3.1 Enzyme preparation

1. **SUMO Protease**

Recombinant SUMO Protease (100 U/100 μL) was purchased from novoprotein (Shanghai, China). The enzyme was used without further treatment and stored at -20°C.

1. **Omniligase-1**

Omniligase-1 was commercially available from Fresenius Kabi. The enzyme (5 mg/mL) was stored at -20°C (short-term) or -80°C (for over a year).

1. **PAM12B**

The expression strain and preparation method was derived from our previous work [2]. *E. coli* BL21(DE3) cells carrying the expression vector pET21a-PAM12B were cultured in 200 mL auto-induction medium (comprising 10.0 g/L tryptone, 5.0 g/L yeast extract, 3.3 g/L (NH_4_)_2_SO_4_, 6.8 g/L KH_2_PO_4_, 17.9 g/L Na_2_HPO_4_∙12H_2_O, 0.5 g/L Glucose∙H_2_O, 2.1 g/L Lactose∙H_2_O, 0.5 g/L MgSO_4_·7H_2_O and 2.0 mL/L glycerol) containing 50 μg/mL ampicillin at 30°C, 180 rpm for 24 h. The cells were collected by centrifugation (8000 g, 10 min, 4°C) and lysed by sonication on ice in buffer A (comprising 50 mM KH_2_PO_4_, 200 mM NaCl and 20 mM imidazole, pH 7.5). After removal of precipitates via centrifugation (14000 g, 60 min, 4°C) and filtration (0.22 μm filter, Millex),the cell extract was obtained, then loaded onto a 5 mL HisTrap HP column (GE Healthcare). The enzyme was collected by elution with buffer B (comprising 50 mM KH_2_PO_4_, 200 mM NaCl and 300 mM imidazole, pH 7.5), then imidazole and NaCl were removed using a HiPrep 26/10 desalting column (GE Healthcare). Purification processes above were carried out using NGC Quest 10 Medium-Pressure Chromatography Systems (Bio-Rad, Hercules, USA). The purified enzyme (in a buffer comprising 50 mM KH_2_PO_4_, pH 7.5) was concentrated to 7 mg/mL via Amicon filtration (30 kDa, Millipore). The yield of purified protein was ~100 mg/L culture. The enzyme was stored at 4°C (for months) and diluted with 0.05 M phosphate buffer solution (pH 7.5) before use. For long-term storage (at 4°C for years), the enzyme could be lyophilized with sucrose (16.7 mg of sucrose per mL of enzyme stock solution) and dissolved with deionized H_2_O before use.

Protein sequence (containing His-tag and linker):

MAEPASRNVPFPYAETDVADLQARMTAGELDSTTLTQAYLQRIAALDRTGPRLHAVIELNPDALKEAAERDRERRDGRLRGPLHGIPLLLKDNINAAPMATSAGSLALQDFRPDDAYLVRRLRDAGAVVLGKTNLSEWGNFRSNNSISGWSARGGQTRNPYRPSHSPCGSSSGSAVAVAANLASVAIGTETDGSIVCPAAINGVVGLKPTVGLVSRDGIIPISFSQDTPGPMARSVADAAAVLTAIAGRDPADPATATMPGRAVYDYTARLDPQGLRGKRIGLLQVPLLKYRGMPPLIEQAATELRRAGAVVVPVELPNYGAWAEAERTLLLYEFKAGLERYFNTHRAPLRSLADLIAFNQAHSKQELALFGQELLVEADATAGLADPAYIRARSDARRLAGPEGIDAALAAHQLDALVAPTTGVAWPIRPEGDDFPGESYSAAAVAGYPSLTVPMGQIDGLPVGLLFMGTAWSEPKLIEMAYAYEQRTRARRPPHFDTDALIDAGEPIEGRLEHHHHHH

1. **PAL**

The protein sequence of PAL (from *Exiguobacterium*) was derived from a US patent (US 9,096,843 B2). *E. coli* BL21(DE3) cells carrying the expression vector pET28a-PAL were cultured in 200 mL LB broth containing 50 μg/mL kanamycin at 37°C, 180 rpm until optical density at 600 nm (OD_600_) reached approximately 0.8. At that point, PAL expression was induced by the addition of 1 mM isopropyl thio-β-D-galactoside (IPTG) followed by incubation at 30°C, 180 rpm overnight. Protein purification process of PAL was identical to that of PAM12B. The purified enzyme (in a buffer comprising 50 mM KH_2_PO_4_ and 50 μM ZnCl_2_, pH 7.5) was concentrated to 12 mg/mL via Amicon filtration (30 kDa, Millipore). The yield of purified protein was ~30 mg/L culture. The enzyme was stored at -20°C (short-term) or -80°C (for over a year).

Protein sequence (containing His-tag and linker):

MGSSHHHHHHSSGLVPRGSHMASMTGGQQMGRGSGPRTDPIFKDEYDEKAKSSRYTSSWVWPEKDSVSHRGGEGSGVSTSPSGYVYYLHRGDGSYANEELITTPTITVFDPNTNEIVDEFGDNLFQSPHGIEVDSQNNIWVTDIMLNKVFKLDERGNVLATFGDDYRLGTETSLRIRNELPNFPVPMNEYTFARPTDVTVMEDGSFIVADGYRNHRIVKFNRDGNIQWEVDAYGSSDGEFNLPHGITHDQSGNIYVADRNNARIQVFDQDGQHLSTWDDTEIGRPYGIDAGNDGNIYLVDGGDYLNGERETPKSQIVVLSPKGEVIERFGSWGNKMGQLRIPHDLTVLEDGTIFVAELLNERLQKFTITE

1. **PHM**

The protein sequence of PHM (from *Rattus norvegicus*, NCBI PIR: S09583) was derived from NCBI (www.ncbi.nlm.nih.gov). Based on the previously reported work on large-scale production of PHM by Bauman et al. [3], the signal peptide (MAGRARSGLLLLLLGLLALQSSCLA) and the residues 42-356 were cloned in the pCAGGS vector to form the expression vector pCAGGS-PHM. Expression of PHM was carried out using ExpiCHO^TM^ expression system kit (Thermo Fisher Scientific), in which ExpiCHO-S^TM^ cells and serum-free medium were applied. Transfection and culture (200 mL) were performed as the user guide indicated. On the seventh day post transfection, the culture medium was collected by centrifugation and filtration (0.22 μm filter, Millex). Purification of PHM by Ni-NTA chromatography was unfruitful after numerous attempts due to unexplained loss of enzyme activity, therefore the crude enzyme was utilized for catalysis. Phosphate buffer solution (50 mM, containing 20 μM CuSO_4_, pH 7.5) was used for substitution of medium via Amicon filtration (3 kDa, Millipore), then the protein solution was concentrated to 8 mg/mL. The yield of total protein was ~1600 mg/L culture. According to the results of SDS-PAGE and Western blot, the yield of PHM was approximately 30% of the total protein. The crude enzyme solution was stored at -20°C (short-term) or -80°C (for over a year).

Protein sequence (containing His-tag and deleting the signal peptide):

SFSNECLGTIGPVTPLDASDFALDIRMPGVTPKESDTYFCMSMRLPVDEEAFVIDFKPRASMDTVHHMLLFGCNMPSSTGSYWFCDEGTCTDKANILYAWARNAPPTRLPKGVGFRVGGETGSKYFVLQVHYGDISAFRDNHKDCSGVSVHLTRVPQPLIAGMYLMMSVDTVIPPGEKVVNADISCQYKMYPMHVFAYRVHTHHLGKVVSGYRVRNGQWTLIGRQNPQLPQAFYPVEHPVDVTFGDILAARCVFTGEGRTEATHIGGTSSDEMCNLYIMYYMEAKYALSFMTCTKNVAPDMFRTIPAEANIPIPVHHHHHH

**
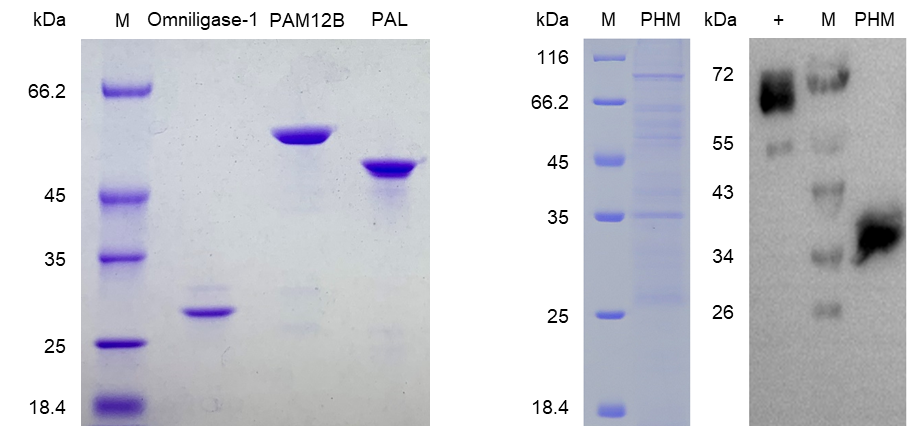
 Supplementary Figure 2.** Omniligase-1 (~28 kDa), PAM12B (~55 kDa), PAL (~41 kDa) and PHM (~36 kDa) were identified by SDS-PAGE and Western blot (anti-His-tag). M: molecular weight marker. +: positive control of HRP-conjugated His-tag antibody (Proteintech Group, USA).


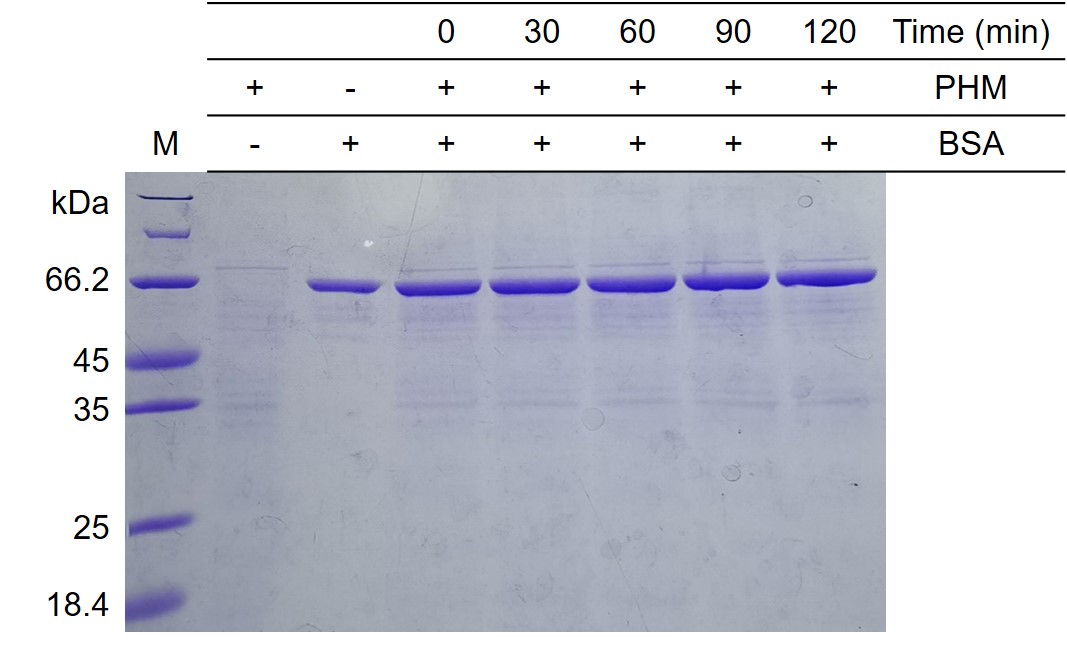


**Supplementary Figure 3.** Bovine serum albumin (BSA, 0.5 mg/mL) was incubated with the PHM crude enzyme (0.05 mg/mL) under the catalytic conditions (0.2 M MES buffer, pH 6.5, 37°C). The mixture was identified by SDS-PAGE and degradation of BSA was not observed after 120 min, indicating that the protease activity of the crude enzyme could be neglected during PHM-catalyzed amidation process. M: molecular weight marker.

## 3.2 Protein substrate preparation

1. **Recombinant expression**

Eight recombinant proteins (FAT10, C4S3, AL-EGFP, SUMO-4-OT(28-62), SUMO-NrdH-G, SUMO-Ub(1-59)-G, SUMO-mHSP10(2-47)-G and SUMO-mHSP10(64-102)) were expressed with a general protocol. *E. coli* BL21(DE3) cells carrying the expression vector were cultured in 200 mL LB broth containing 50 μg/mL ampicillin (pET21a-derived vectors) or kanamycin (pET28b-derived vectors) at 37°C, 180 rpm until OD_600_ reached approximately 0.8. At that point, protein expression was induced by the addition of 1 mM IPTG followed by incubation at 37°C, 180 rpm for 5 h. The cells were collected by centrifugation (8000 g, 10 min, 4°C) and lysed by sonication on ice in buffer A (comprising 50 mM KH_2_PO_4_, 200 mM NaCl and 20 mM imidazole, pH 7.5). FAT10 was obtained in the form of inclusion body, which was initially washed with deionized H_2_O, afterward dissolved in a 0.2 M Phosphate buffer solution containing 6 M Gn·HCl (pH 7.0). FAT10 powder was prepared after preparative HPLC purification and lyophilization, then stored at -20°C.

Other proteins were expressed in the soluble form. The cell extract was obtained after removing precipitates via centrifugation (14,000 g, 60 min, 4°C) and filtration (0.22 μm filter, Millex). Purification processes were carried out using NGC Quest 10 Medium-Pressure Chromatography Systems (Bio-Rad, Hercules, USA). The protein solution was loaded onto a 5 mL HisTrap HP column (GE Healthcare). The gradient of imidazole concentration was set as 20 to 300 mM in 20 min (buffer B comprised 50 mM KH_2_PO_4_, 200 mM NaCl and 300 mM imidazole, pH 7.5). The main protein peak (detected at 280 nm) was collected and desalted using a HiPrep 26/10 desalting column (GE Healthcare). Concentrated to ~15 mg/mL via Amicon filtration (3 kDa, Millipore), C4S3 and AL-EGFP were stored in a buffer comprising 50 mM KH_2_PO_4_, pH 7.5 at -20°C. SUMO-4-OT(28-62), SUMO-NrdH-G, SUMO-Ub(1-59)-G, SUMO-mHSP10(2-47)-G and SUMO-mHSP10(64-102) were stored in a buffer comprising 50 mM Tris·HCl and 150 mM NaCl, pH 8.0 in the concentration of 0.1~0.2 mM.

1. **Removal of SUMO**

To the aforementioned SUMO-protein solution, DTT and SUMO protease were added in the concentration of 2 mM and 1 U/mL, respectively. The reaction mixture was incubated at 37°C for 1~4 h, then loaded onto a 5 mL HisTrap HP column (GE Healthcare). The solution flowing through the column was loaded onto the column again, and the loading operation was performed for 5 times in total, then unbounded proteins were collected. NrdH-G, Ub(1-59)-G and mHSP10(2-47)-G were stored in a buffer comprising 200 mM 2-(N-Morpholino)ethanesulfonic acid (MES), pH 6.5. 4-OT(28-62) and mHSP10(64-102) were stored in a buffer comprising 50 mM KH_2_PO_4_, pH 7.5. Purification processes above were carried out using NGC Quest 10 Medium-Pressure Chromatography Systems (Bio-Rad, Hercules, USA).

1. **Protein sequences**

Protein sequences were identified through DNA sequencing, and the prepared protein substrates were identified by ESI-MS. Sequences of the recombinant proteins were shown below. Underlined parts were removed after the treatment of SUMO protease.

**FAT10-His*6** (sequence was from PDB: 6GF2)

GAMGDEELPLFLVESGDEAKRHLLQVRRSSSVAQVKAMIETKTGIIPETQIVTLNGKRLEDGKMMADYGIRKGNLLFLASYSIGGLEHHHHHH

**C4S3-His*6** (sequence was from PDB: 6CBU)

GMAEGNTLISVDYEIFGKVQGVFFRKHTQAEGKKLGLVGWVQNTDRGTVQGQLQGPISKVRHMQEWLETRGSPKSHIDKANFNNEKLIEELDYSDFQIVALEHHHHHH

**AL-EGFP-His*6** (sequence was from PDB: 2Y0G)

ALVSKGEELFTGVVPILVELDGDVNGHKFSVSGEGEGDATYGKLTLKFICTTGKLPVPWPTLVTTLTYGVQCFSRYPDHMKQHDFFKSAMPEGYVQERTIFFKDDGNYKTRAEVKFEGDTLVNRIELKGIDFKEDGNILGHKLEYNYNSHNVYIMADKQKNGIKVNFKIRHNIEDGSVQLADHYQQNTPIGDGPVLLPDNHYLSTQSALSKDPNEKRDHMVLLEFVTAAGITLGMDELYKLEHHHHHH

**His*6-SUMO-4-OT(28-62)** (sequence was from PDB: 4X19, mutant A33D)

GSSHHHHHHGSGLVPRGSASMSDSEVNQEAKPEVKPEVKPETHINLKVSDGSSEIFFKIKKTTPLRRLMEAFAKRQGKEMDSLRFLYDGIRIQADQTPEDLDMEDNDIIEAHREQIGGSRSLDDPLTSVRVIITEMAKGHFGIGGELASKVRR

**His*6-SUMO-Ub(1-59)-G** (sequence was from PDB: 1TBE)

GSSHHHHHHGSGLVPRGSASMSDSEVNQEAKPEVKPEVKPETHINLKVSDGSSEIFFKIKKTTPLRRLMEAFAKRQGKEMDSLRFLYDGIRIQADQTPEDLDMEDNDIIEAHREQIGGMQIFVKTLTGKTITLEVEPSDTIENVKAKIQDKEGIPPDQQRLIFAGKQLEDGRTLSDYG

**His*6-SUMO-NrdH-G** (sequence was from PDB: 4FIW)

GSSHHHHHHGSGLVPRGSASMSDSEVNQEAKPEVKPEVKPETHINLKVSDGSSEIFFKIKKTTPLRRLMEAFAKRQGKEMDSLRFLYDGIRIQADQTPEDLDMEDNDIIEAHREQIGGMAITVYTKPACVQCNATKKALDRAGLEYDLVDISLDEEAREYVLALGYLQAPVVVADGSHWSGFRPERIREMATAAAG

**His*6-SUMO-mHSP10(2-47)-G** (sequence was from UniProt: P61604-1)

GSSHHHHHHGSGLVPRGSASMSDSEVNQEAKPEVKPEVKPETHINLKVSDGSSEIFFKIKKTTPLRRLMEAFAKRQGKEMDSLRFLYDGIRIQADQTPEDLDMEDNDIIEAHREQIGGAGQAFRKFLPLFDRVLVERSAAETVTKGGIMLPEKSQGKVLQATVVG

**His*6-SUMO-mHSP10(64-102)** (sequence was from UniProt: P61604-1)

GSSHHHHHHGSGLVPRGSASMSDSEVNQEAKPEVKPEVKPETHINLKVSDGSSEIFFKIKKTTPLRRLMEAFAKRQGKEMDSLRFLYDGIRIQADQTPEDLDMEDNDIIEAHREQIGGSVKVGDKVLLPEYGGTKVVLDDKDYFLFRDGDILGKYVD

## 3.3 Peptide hydrazide substrate preparation

Ten milligram-scale production of the peptide hydrazides was carried out with a general protocol. Peptide amides were dissolved to ~6 mg/mL with deionized H_2_O in a 15-mL tube. 20 M N_2_H_4_ solution and PAM12B stock solution were added into the reaction mixture, then the hydrazidation reaction was carried out for 15~30 min at room temperature and monitored by HPLC (220 nm). When the conversion of peptide amide was completed, the peptide hydrazide was purified by preparative HPLC and lyophilized overnight. The isolated yield was >90% for each case. The products were identified by ESI-MS.

**Supplementary Table 4.** Final concentration of the peptide amides, hydrazine and PAM12B

|  | Peptide amide | N_2_H_4_ | PAM12B |
| --- | --- | --- | --- |
| Concentration (mM) | 2~5 | 1000 | 2×10^-5^~10^-2^ |

**Supplementary Table 5.** Peptide hydrazides prepared from the commercially-synthesized peptide amides

| No. * | Peptide amide | Peptide hydrazide |
| --- | --- | --- |
| 177 | DLSKQMEEEAVRL-NH_2_ | DLSKQMEEEAVRL-N_2_H_3_ |
| 179 | PIAQIHILEGRSDEQKETLIREVSEAI-NH_2_ | PIAQIHILEGRSDEQKETLIREVSEAI-N_2_H_3_ |
| 180 | VLRIIRIAGRGLRRLGRKIAHGVKKYGPT-NH_2_ | VLRIIRIAGRGLRRLGRKIAHGVKKYGPT- N_2_H_3_ |
| 187 | AVGSGSKG-(^Ac^K)-GGEIQPV-NH_2_ | AVGSGSKG-(^Ac^K)-GGEIQPV-N_2_H_3_ |
| 188 | SRSLDDPLTSV-NH_2_ | SRSLDDPLTSV-N_2_H_3_ |

* The serial number in **Supplementary Table 2**.

# 4 Supplementary methods and results

## 4.1 Screening of alcohols for enzymatic ligation

**
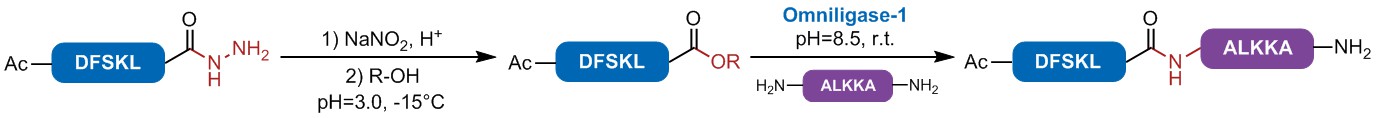
**

**Supplementary Figure 4.** The reaction route of alcohol screening for enzymatic ligation.

1. **Reagent setup**

**Ac-DFSKL-N_2_H_3_ stock solution (28 mM):**

14.9 mg of Ac-DFSKL-N_2_H_3_ was added into a 1.5-mL Eppendorf tube. Then 800 μL of 0.2 M phosphate solution containing 6 M Gn·HCl (pH 3.0) was added into the tube to dissolve the peptide hydrazide.

**ALKKA-NH_2_ stock solution (105 mM):**

10.0 mg of ALKKA-NH_2_ was added into a 1.5-mL Eppendorf tube. Then 180 µL of deionized H_2_O was added into the tube to dissolve the peptide.

**Alcohol stock solution (2 M):**

0.4 mmol of the alcohol was dissolved in 200 µL of deionized H_2_O or NaOH solution. For the alcohols with low solubility, supernatant was used after centrifugation.

1. **Reaction conditions**

The method was revised from a reported protocol [1] for peptide thioester at a ten milligram-scale. Initially, 25 µL of Ac-DFSKL-N_2_H_3_ stock solution was pipetted into the 1.5-mL eppendorf tube and precooled at -15°C for 10 min. Then, 10 µL of 0.5 M NaNO_2_ solution was added into the reaction tube and the temperature was kept at -15°C for 15 min. Next, 15 µL of alcohol stock solution was added into the reaction tube and the temperature was kept at -15°C for another 5 min. Afterwards, the tube was removed from the reactor and allowed to warm to room temperature. The pH was adjusted to 8.0~8.5 with 1 M NaOH solution. For ligation, 25 µL of the reaction mixture was transferred into a new 1.5-mL eppendorf tube, and 5 µL of ALKKA-NH_2_ stock solution and 5 µL of Omniligase-1 stock solution were added. The ligation reaction was carried out for 30 min at room temperature.

**Supplementary Table 6.** Final concentration of the peptides and Omniligase-1

|  | DFSKL | ALKKA | Omniligase-1 |
| --- | --- | --- | --- |
| Concentration (mM) | 10 | 15 | 0.03 |

1. **LC-MS analysis**

The reaction mixtures were analyzed with LC-MS general method A. The overall yields were estimated by integration of the peak areas of ligation product (A), hydrolysis product (B) and remaining peptide ester (C) monitored by HPLC (220 nm).

$$Overall yield \left( \% \right)= \frac{A}{A+B+C} \times100\%$$

1. **Results**

HPLC traces were shown in **Supplementary HPLC traces and MS figures chapter 1.1**.

**Supplementary Table 7.** Proportions of peak areas of compounds (screening of alcohols)

| Type | No. | Ligation | Hydrolysis | Other |
| --- | --- | --- | --- | --- |
| alkyl alcohols | a1 | 9.33% | 90.67% | 0.00% |
|  | a2 | 18.44% | 81.56% | 0.00% |
|  | a3 | 5.17% | 94.83% | 0.00% |
|  | a4 | 8.42% | 91.58% | 0.00% |
| polar alcohols | b1 | 20.49% | 79.51% | 0.00% |
|  | b2 | 0.00% | 2.46% | 97.54% * |
|  | b3 | 14.90% | 85.10% | 0.00% |
|  | b4 | 13.10% | 86.90% | 0.00% |
| fluorinated alcohols | c1 | 37.80% | 62.20% | 0.00% |
|  | c2 | 49.29% | 50.71% | 0.00% |
| benzyl alcohols | d1 | 0.00% | 100.00% | 0.00% |
|  | d2 | 15.80% | 84.20% | 0.00% |
|  | d3 | 10.56% | 89.44% | 0.00% |
|  | d4 | 5.26% | 94.74% | 0.00% |
|  | d5 | 34.42% | 65.58% | 0.00% |
| phenol derivatives | e1 | 2.85% | 97.15% | 0.00% |
|  | e2 | 21.57% | 8.74% | 69.68% * |
|  | e3 | 28.72% | 71.28% | 0.00% |
|  | e4 | 7.52% | 92.48% | 0.00% |
|  | e5 | 0.00% | 100.00% | 0.00% |
|  | e6 # | 86.13% | 11.36% | 2.51% (ester) |
|  | e7 | 82.58% | 11.35% | 6.07% (ester) |
|  | e8 | 84.68% | 11.31% | 4.01% (ester) |
|  | e9 | 54.79% | 45.21% | 0.00% |
|  | e10 | 37.45% | 62.55% | 0.00% |

* The molecular weight of the remaining compound was identical to that of the peptide ester according to ESI-MS. It was inferred that an amide bond was formed between C-terminus of the peptide and the alcohol.

# Racemization of the peptide phenolic esters was identified with Ac-Asp-Phe-Ser-Lys-(L)-Phe-N_2_H_3_, Ac-Asp-Phe-Ser-Lys-(D)-Phe-N_2_H_3_, Ac-Asp-Phe-Ser-Lys-(L)-Leu-N_2_H_3_ and Ac-Asp-Phe-Ser-Lys-(D)-Leu-N_2_H_3_. Experimental details indicated that the racemization extent is lower than 4%. HPLC traces were shown in **Supplementary HPLC traces and MS figures chapter 1.2**.

## 4.2 Screening of substrate spectrum of Omniligase-1

**
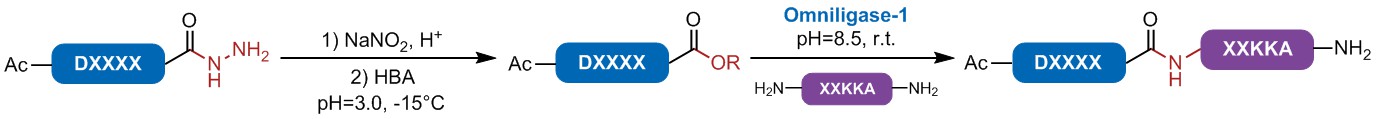
**

**Supplementary Figure 5.** The reaction route of substrate spectrum screening of Omniligase-1.

1. **Reagent setup**

**Acyl donor peptide stock solution (28 mM):**

2.8 μmol of Ac-DXXXX-N_2_H_3_ was added into a 1.5-mL Eppendorf tube. Then 100 μL of 0.2 M phosphate solution containing 6 M Gn·HCl (pH 3.0) was added into the tube to dissolve the peptide hydrazide.

**Acyl receptor peptide stock solution (105 mM):**

2.1 μmol of XXKKA-NH_2_ was added into a 1.5-mL Eppendorf tube. Then 20 μL of deionized H_2_O was added into the tube to dissolve the peptide.

1. **Reaction conditions**

Initially, 25 µL of Ac-DXXXX-N_2_H_3_ stock solution was pipetted into the 1.5-mL eppendorf tube and precooled at -15°C for 10 min. Then, 10 µL of 0.5 M NaNO_2_ solution was added into the reaction tube and the temperature was kept at -15°C for 15 min. Next, 15 µL of 2 M HBA stock solution was added into the reaction tube and the temperature was kept at -15°C for another 5 min. Afterwards, the tube was removed from the reactor and allowed to warm to room temperature. The pH was adjusted to 8.0~8.5 with 1 M NaOH solution. For ligation, 25 µL of the reaction mixture was transferred into a new 1.5-mL eppendorf tube, and 5 µL of XXKKA-NH_2_ stock solution and 5 µL of Omniligase-1 stock solution were added. The ligation reaction was carried out for 30 min at room temperature.

**Supplementary Table 8.** The final concentration of the peptides and Omniligase-1

|  | DXXXX | XXKKA | Omniligase-1 |
| --- | --- | --- | --- |
| Concentration (mM) | 10 | 15 | 0.03 |

(For conjugation of Ac-DFSKP-N_2_H_3_ and ALKKA-NH_2_, the final concentration of Omniligase-1 was 0.3 mM.)

1. **LC-MS analysis**

The reaction mixtures were analyzed with LC-MS general method A (no sign in **Supplementary Table 9-14**) or B (labelled by * in **Supplementary Table 9-14**). The ligation rates were estimated by integration of the peak areas of ligation product (A), hydrolysis product (B) and remaining peptide ester (C) monitored by HPLC (220 nm).

$$Ligation rate \left( \% \right)= \frac{A}{A+B+C} \times100\%$$

1. **Results**

HPLC traces and mass spectrum were shown in **Supplementary HPLC traces and MS figures chapter 2**.

**Supplementary Table 9.** Proportions of peak areas of compounds (screening of Omniligase-1 P4 substrates)

| Acyl donor | Acyl receptor | Ligation | Hydrolysis | Other |
| --- | --- | --- | --- | --- |
| Ac-DFSKL-N_2_H_3_ | ALKKA-NH_2_ | 89.39% | 10.61% | 0.00% |
| Ac-DVSKL-N_2_H_3_ | ALKKA-NH_2_ | 88.42% | 11.58% | 0.00% |
| Ac-DISKL-N_2_H_3_ | ALKKA-NH_2_ | 85.55% | 14.45% | 0.00% |
| Ac-DMSKL-N_2_H_3_ | ALKKA-NH_2_ | 85.37% | 14.63% | 0.00% |
| Ac-DLSKL-N_2_H_3_ | ALKKA-NH_2_ | 84.03% | 15.97% | 0.00% |
| Ac-DCSKL-N_2_H_3_ | ALKKA-NH_2_ | 79.32% | 20.68% | 0.00% |
| Ac-DASKL-N_2_H_3_ | ALKKA-NH_2_ | 75.90% * | 24.10% | 0.00% |
| Ac-DWSKL-N_2_H_3_ | ALKKA-NH_2_ | 62.98% | 37.02% | 0.00% |
| Ac-DYSKL-N_2_H_3_ | ALKKA-NH_2_ | 57.96% | 42.04% | 0.00% |
| Ac-DSSKL-N_2_H_3_ | ALKKA-NH_2_ | 32.98% * | 67.02% | 0.00% |
| Ac-DRSKL-N_2_H_3_ | ALKKA-NH_2_ | 18.53% * | 68.03% | 13.44% (ester) |
| Ac-DQSKL-N_2_H_3_ | ALKKA-NH_2_ | 10.90% * | 89.10% | 0.00% |
| Ac-DDSKL-N_2_H_3_ | ALKKA-NH_2_ | 11.03% * | 70.70% | 18.27% (ester) |
| Ac-DNSKL-N_2_H_3_ | ALKKA-NH_2_ | 11.77% * | 88.23% | 0.00% |
| Ac-DGSKL-N_2_H_3_ | ALKKA-NH_2_ | 12.70% | 87.30% | 0.00% |
| Ac-DHSKL-N_2_H_3_ | ALKKA-NH_2_ | 5.62% * | 94.38% | 0.00% |
| Ac-DESKL-N_2_H_3_ | ALKKA-NH_2_ | 3.56% * | 96.44% | 0.00% |
| Ac-DKSKL-N_2_H_3_ | ALKKA-NH_2_ | 3.38% * | 96.62% | 0.00% |
| Ac-DPSKL-N_2_H_3_ | ALKKA-NH_2_ | 27.07% | 72.93% | 0.00% |
| Ac-DTSKL-N_2_H_3_ | ALKKA-NH_2_ | 60.88% * | 39.12% | 0.00% |

**Supplementary Table 10.** Proportions of peak areas of compounds (screening of Omniligase-1 P3 substrates)

| Acyl donor | Acyl receptor | Ligation | Hydrolysis | Other |
| --- | --- | --- | --- | --- |
| Ac-DFFKL-N_2_H_3_ | ALKKA-NH_2_ | 74.43% | 25.57% | 0.00% |
| Ac-DFVKL-N_2_H_3_ | ALKKA-NH_2_ | 98.04% | 1.96% | 0.00% |
| Ac-DFIKL-N_2_H_3_ | ALKKA-NH_2_ | 73.79% * | 26.21% | 0.00% |
| Ac-DFMKL-N_2_H_3_ | ALKKA-NH_2_ | 74.24% | 25.76% | 0.00% |
| Ac-DFLKL-N_2_H_3_ | ALKKA-NH_2_ | 75.29% | 24.71% | 0.00% |
| Ac-DFCKL-N_2_H_3_ | ALKKA-NH_2_ | 84.80% | 15.20% | 0.00% |
| Ac-DFAKL-N_2_H_3_ | ALKKA-NH_2_ | 77.20% | 22.80% | 0.00% |
| Ac-DFWKL-N_2_H_3_ | ALKKA-NH_2_ | 76.43% | 23.57% | 0.00% |
| Ac-DFYKL-N_2_H_3_ | ALKKA-NH_2_ | 84.26% | 15.74% | 0.00% |
| Ac-DFSKL-N_2_H_3_ | ALKKA-NH_2_ | 89.39% | 10.61% | 0.00% |
| Ac-DFRKL-N_2_H_3_ | ALKKA-NH_2_ | 79.08% * | 20.92% | 0.00% |
| Ac-DFQKL-N_2_H_3_ | ALKKA-NH_2_ | 80.43% | 19.57% | 0.00% |
| Ac-DFDKL-N_2_H_3_ | ALKKA-NH_2_ | 78.19% | 21.81% | 0.00% |
| Ac-DFNKL-N_2_H_3_ | ALKKA-NH_2_ | 82.63% | 17.37% | 0.00% |
| Ac-DFGKL-N_2_H_3_ | ALKKA-NH_2_ | 86.95% | 13.05% | 0.00% |
| Ac-DFHKL-N_2_H_3_ | ALKKA-NH_2_ | 71.46% | 28.54% | 0.00% |
| Ac-DFEKL-N_2_H_3_ | ALKKA-NH_2_ | 87.63% | 12.37% | 0.00% |
| Ac-DFKKL-N_2_H_3_ | ALKKA-NH_2_ | 90.73% * | 9.27% | 0.00% |
| Ac-DFPKL-N_2_H_3_ | ALKKA-NH_2_ | 9.75% | 74.50% | 15.75% (ester) |
| Ac-DFTKL-N_2_H_3_ | ALKKA-NH_2_ | 82.08% | 17.92% | 0.00% |

**Supplementary Table 11.** Proportions of peak areas of compounds (screening of Omniligase-1 P2 substrates)

| Acyl donor | Acyl receptor | Ligation | Hydrolysis | Other |
| --- | --- | --- | --- | --- |
| Ac-DFSFL-N_2_H_3_ | ALKKA-NH_2_ | 72.76% | 27.24% | 0.00% |
| Ac-DFSVL-N_2_H_3_ | ALKKA-NH_2_ | 84.92% | 15.08% | 0.00% |
| Ac-DFSIL-N_2_H_3_ | ALKKA-NH_2_ | 82.39% | 17.61% | 0.00% |
| Ac-DFSML-N_2_H_3_ | ALKKA-NH_2_ | 79.01% | 20.99% | 0.00% |
| Ac-DFSLL-N_2_H_3_ | ALKKA-NH_2_ | 80.71% | 19.29% | 0.00% |
| Ac-DFSCL-N_2_H_3_ | ALKKA-NH_2_ | 85.50% | 14.50% | 0.00% |
| Ac-DFSAL-N_2_H_3_ | ALKKA-NH_2_ | 80.81% | 19.19% | 0.00% |
| Ac-DFSWL-N_2_H_3_ | ALKKA-NH_2_ | 78.90% | 21.10% | 0.00% |
| Ac-DFSYL-N_2_H_3_ | ALKKA-NH_2_ | 78.04% | 21.96% | 0.00% |
| Ac-DFSSL-N_2_H_3_ | ALKKA-NH_2_ | 87.52% | 12.48% | 0.00% |
| Ac-DFSRL-N_2_H_3_ | ALKKA-NH_2_ | 78.76% | 21.24% | 0.00% |
| Ac-DFSQL-N_2_H_3_ | ALKKA-NH_2_ | 81.75% | 18.25% | 0.00% |
| Ac-DFSDL-N_2_H_3_ | ALKKA-NH_2_ | 76.91% | 23.09% | 0.00% |
| Ac-DFSNL-N_2_H_3_ | ALKKA-NH_2_ | 80.50% | 19.50% | 0.00% |
| Ac-DFSGL-N_2_H_3_ | ALKKA-NH_2_ | 74.89% | 25.11% | 0.00% |
| Ac-DFSHL-N_2_H_3_ | ALKKA-NH_2_ | 68.59% | 31.41% | 0.00% |
| Ac-DFSEL-N_2_H_3_ | ALKKA-NH_2_ | 87.29% | 12.71% | 0.00% |
| Ac-DFSKL-N_2_H_3_ | ALKKA-NH_2_ | 89.39% | 10.61% | 0.00% |
| Ac-DFSPL-N_2_H_3_ | ALKKA-NH_2_ | 73.22% | 26.78% | 0.00% |
| Ac-DFSTL-N_2_H_3_ | ALKKA-NH_2_ | 87.31% | 12.69% | 0.00% |

**Supplementary Table 12.** Proportions of peak areas of compounds (screening of Omniligase-1 P1 substrates)

| Acyl donor | Acyl receptor | Ligation | Hydrolysis | Other |
| --- | --- | --- | --- | --- |
| Ac-DFSKF-N_2_H_3_ | ALKKA-NH_2_ | 75.89% | 24.11% | 0.00% |
| Ac-DFSKV-N_2_H_3_ | ALKKA-NH_2_ | 90.32% | 9.68% | 0.00% |
| Ac-DFSKI-N_2_H_3_ | ALKKA-NH_2_ | 89.51% | 10.49% | 0.00% |
| Ac-DFSKM-N_2_H_3_ | ALKKA-NH_2_ | 81.66% | 18.34% | 0.00% |
| Ac-DFSKL-N_2_H_3_ | ALKKA-NH_2_ | 89.39% | 10.61% | 0.00% |
| Ac-DFSKC-N_2_H_3_ | ALKKA-NH_2_ | 11.81% | 88.19% | 0.00% |
| Ac-DFSKA-N_2_H_3_ | ALKKA-NH_2_ | 87.60% | 12.40% | 0.00% |
| Ac-DFSKW-N_2_H_3_ | ALKKA-NH_2_ | 74.62% | 25.38% | 0.00% |
| Ac-DFSKY-N_2_H_3_ | ALKKA-NH_2_ | 75.66% * | 24.34% | 0.00% |
| Ac-DFSKS-N_2_H_3_ | ALKKA-NH_2_ | 84.08% * | 15.92% | 0.00% |
| Ac-DFSKR-N_2_H_3_ | ALKKA-NH_2_ | 32.27% * | 67.73% | 0.00% |
| Ac-DFSKQ-N_2_H_3_ | ALKKA-NH_2_ | 80.94% * | 19.06% | 0.00% |
| Ac-DFSKD-N_2_H_3_ | ALKKA-NH_2_ | 1.80% * | 98.20% | 0.00% |
| Ac-DFSKN-N_2_H_3_ | ALKKA-NH_2_ | 70.20% * | 29.80% | 0.00% |
| Ac-DFSKG-N_2_H_3_ | ALKKA-NH_2_ | 83.74% * | 16.26% | 0.00% |
| Ac-DFSKH-N_2_H_3_ | ALKKA-NH_2_ | 66.61% * | 33.39% | 0.00% |
| Ac-DFSKE-N_2_H_3_ | ALKKA-NH_2_ | 37.65% * | 62.35% | 0.00% |
| Ac-DFSKK-N_2_H_3_ | ALKKA-NH_2_ | 38.28% * | 26.87% | 34.85% (dimer) |
| Ac-DFSKP-N_2_H_3_**^*^** | ALKKA-NH_2_ | 82.62% * | 17.38% | 0.00% |
| Ac-DFSKT-N_2_H_3_ | ALKKA-NH_2_ | 86.34% * | 13.66% | 0.00% |

**Supplementary Table 13.** Proportions of peak areas of compounds (screening of Omniligase-1 P1’ substrates)

| Acyl donor | Acyl receptor | Ligation | Hydrolysis | Other |
| --- | --- | --- | --- | --- |
| Ac-DFSKL-N_2_H_3_ | FLKKA-NH_2_ | 83.47% | 16.53% | 0.00% |
| Ac-DFSKL-N_2_H_3_ | VLKKA-NH_2_ | 83.86% * | 16.14% | 0.00% |
| Ac-DFSKL-N_2_H_3_ | ILKKA-NH_2_ | 68.95% | 31.05% | 0.00% |
| Ac-DFSKL-N_2_H_3_ | MLKKA-NH_2_ | 74.91% * | 25.09% | 0.00% |
| Ac-DFSKL-N_2_H_3_ | LLKKA-NH_2_ | 57.26% | 42.74% | 0.00% |
| Ac-DFSKL-N_2_H_3_ | CLKKA-NH_2_ | 82.78% | 17.22% | 0.00% |
| Ac-DFSKL-N_2_H_3_ | ALKKA-NH_2_ | 89.39% | 10.61% | 0.00% |
| Ac-DFSKL-N_2_H_3_ | WLKKA-NH_2_ | 93.21% | 6.79% | 0.00% |
| Ac-DFSKL-N_2_H_3_ | YLKKA-NH_2_ | 80.48% | 19.52% | 0.00% |
| Ac-DFSKL-N_2_H_3_ | SLKKA-NH_2_ | 88.13% | 11.87% | 0.00% |
| Ac-DFSKL-N_2_H_3_ | RLKKA-NH_2_ | 71.30% | 28.70% | 0.00% |
| Ac-DFSKL-N_2_H_3_ | QLKKA-NH_2_ | 86.57% | 13.43% | 0.00% |
| Ac-DFSKL-N_2_H_3_ | DLKKA-NH_2_ | 80.57% | 19.43% | 0.00% |
| Ac-DFSKL-N_2_H_3_ | NLKKA-NH_2_ | 85.63% | 14.37% | 0.00% |
| Ac-DFSKL-N_2_H_3_ | GLKKA-NH_2_ | 84.34% | 15.66% | 0.00% |
| Ac-DFSKL-N_2_H_3_ | HLKKA-NH_2_ | 78.48% | 21.52% | 0.00% |
| Ac-DFSKL-N_2_H_3_ | ELKKA-NH_2_ | 82.28% | 17.72% | 0.00% |
| Ac-DFSKL-N_2_H_3_ | KLKKA-NH_2_ | 69.22% | 30.78% | 0.00% |
| Ac-DFSKL-N_2_H_3_ | PLKKA-NH_2_ | 11.30% | 88.70% | 0.00% |
| Ac-DFSKL-N_2_H_3_ | TLKKA-NH_2_ | 85.76% | 14.24% | 0.00% |

**Supplementary Table 14.** Proportions of peak areas of compounds (screening of Omniligase-1 P2’ substrates)

| Acyl donor | Acyl receptor | Ligation | Hydrolysis | Other |
| --- | --- | --- | --- | --- |
| Ac-DFSKL-N_2_H_3_ | AFKKA-NH_2_ | 91.53% | 8.47% | 0.00% |
| Ac-DFSKL-N_2_H_3_ | AVKKA-NH_2_ | 91.34% | 8.66% | 0.00% |
| Ac-DFSKL-N_2_H_3_ | AIKKA-NH_2_ | 87.90% | 12.10% | 0.00% |
| Ac-DFSKL-N_2_H_3_ | AMKKA-NH_2_ | 91.65% | 8.35% | 0.00% |
| Ac-DFSKL-N_2_H_3_ | ALKKA-NH_2_ | 89.39% | 10.61% | 0.00% |
| Ac-DFSKL-N_2_H_3_ | ACKKA-NH_2_ | 83.95% | 16.05% | 0.00% |
| Ac-DFSKL-N_2_H_3_ | AAKKA-NH_2_ | 66.56% | 33.44% | 0.00% |
| Ac-DFSKL-N_2_H_3_ | AWKKA-NH_2_ | 94.69% | 5.31% | 0.00% |
| Ac-DFSKL-N_2_H_3_ | AYKKA-NH_2_ | 93.84% | 6.16% | 0.00% |
| Ac-DFSKL-N_2_H_3_ | ASKKA-NH_2_ | 47.50% | 52.50% | 0.00% |
| Ac-DFSKL-N_2_H_3_ | ARKKA-NH_2_ | 67.01% * | 32.99% | 0.00% |
| Ac-DFSKL-N_2_H_3_ | AQKKA-NH_2_ | 64.19% | 35.81% | 0.00% |
| Ac-DFSKL-N_2_H_3_ | ADKKA-NH_2_ | 22.71% | 77.29% | 0.00% |
| Ac-DFSKL-N_2_H_3_ | ANKKA-NH_2_ | 57.78% | 42.22% | 0.00% |
| Ac-DFSKL-N_2_H_3_ | AGKKA-NH_2_ | 51.63% | 48.37% | 0.00% |
| Ac-DFSKL-N_2_H_3_ | AHKKA-NH_2_ | 90.34% * | 9.66% | 0.00% |
| Ac-DFSKL-N_2_H_3_ | AEKKA-NH_2_ | 28.76% | 71.24% | 0.00% |
| Ac-DFSKL-N_2_H_3_ | AKKKA-NH_2_ | 85.57% * | 14.43% | 0.00% |
| Ac-DFSKL-N_2_H_3_ | APKKA-NH_2_ | 4.51% | 95.49% | 0.00% |
| Ac-DFSKL-N_2_H_3_ | ATKKA-NH_2_ | 78.58% | 21.42% | 0.00% |

These reactions (except P1 = Pro) were carried out under the identical conditions, and the hydrolysis rate can be reduced by improving the conditions for a specific reaction. For example, other Peptiligase family mutants (e.g. Thymoligase) bearing various substrate preferences are available for use, and the acyl acceptor peptide concentration can be raised. In the practical application on protein total synthesis, shifting the Peptiligase-contacting frame to select a new ligation site tends to be a more efficient way to raise the ligation efficiency due to broad sequence compatibility of Omniligase-1.

## 4.3 Screening of substrate spectrum of PAM12B


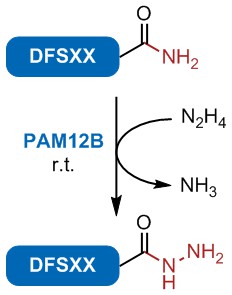


**Supplementary Figure 6.** Reaction route for screening of PAM12B substrate spectrum with N_2_H_4_.

1. **Reagent Setup**

**Peptide amide stock solution (25 mM):**

2.0 μmol of Ac-DFSXX-NH_2_ was added into a 1.5-mL Eppendorf tube. Then 80 μL of deionized H_2_O was added into the tube to dissolve the peptide. The peptides with poor-solubility (e.g. P2 = Val peptide) were dissolved after the addition of N_2_H_4_ solution.

1. **Reaction conditions**

Initially, 10 µL of Ac-DFSXX-NH_2_ stock solution and (35-Y) μL of deionized H_2_O were pipetted into the 1.5-mL eppendorf tube. Then Y µL of 20 M N_2_H_4_ solution and 5 µL of appropriately diluted PAM12B solution were added into the mixture. The hydrazidation reaction was carried out at room temperature for 5~60 min generally and monitored by HPLC every 10~15 min.

1. **HPLC analysis**

The reaction mixtures were analyzed with HPLC general method C. The hydrazidation rates were estimated by integration of the peak areas of hydrazidation product (A), hydrolysis product (B) and remaining peptide amide (C) monitored by HPLC (220 nm).

$$Hydrazidation rate \left( \% \right)= \frac{A}{A+B+C} \times100\%$$

1. **Results**

HPLC traces were shown in **Supplementary HPLC traces and MS figures chapter 3**.

**Supplementary Table 15.** Proportions of peak areas of compounds (screening of PAM12B P2 substrates)

| P2  residue | Peptide conc. (mM) | Hydrazine conc. (M) | PAM12B conc. (µM) | Reaction time (min) | Hydrazidation | Hydrolysis | Amide |
| --- | --- | --- | --- | --- | --- | --- | --- |
| L | 5 | 1.0 | 0.12 | 5 | 89.61% | 3.75% | 6.64% |
| Y | 5 | 1.0 | 0.96 | 35 | 86.79% | 10.05% | 3.16% |
| V | 5 | 1.0 | 0.06 | 15 | 97.60% | 2.40% | 0.00% |
| I | 5 | 1.0 | 0.06 | 20 | 98.68% | 1.32% | 0.00% |
| M | 5 | 1.0 | 0.06 | 15 | 94.91% | 2.57% | 2.52% |
| F | 5 | 1.0 | 0.06 | 55 | 96.31% | 3.69% | 0.00% |
| W | 5 | 1.0 | 0.06 | 35 | 96.94% | 3.06% | 0.00% |
| A | 5 | 1.0 | 0.60 | 5 | 100.00% | 0.00% | 0.00% |
| R | 5 | 1.0 | 0.06 | 15 | 90.54% | 7.98% | 1.48% |
| G | 5 | 1.0 | 0.12 | 5 | 93.51% | 6.49% | 0.00% |
| E | 5 | 1.0 | 6.00 | 65 | 96.26% | 3.74% | 0.00% |
| S | 5 | 1.0 | 0.02 | 35 | 97.66% | 2.34% | 0.00% |
| T | 5 | 1.0 | 0.48 | 15 | 92.55% | 7.45% | 0.00% |
| H | 5 | 1.0 | 0.12 | 5 | 82.26% | 17.74% | 0.00% |
| K | 5 | 0.5 | 0.06 | 45 | 98.75% | 1.25% | 0.00% |
| D | 5 | 1.0 | 0.12 | 25 | 100.00% | 0.00% | 0.00% |
| C | 5 | 1.0 | 0.06 | 50 | 91.90% | 8.10% | 0.00% |
| Q | 5 | 1.0 | 0.12 | 5 | 100.00% | 0.00% | 0.00% |
| P | 5 | 1.0 | 0.02 | 25 | 97.54% | 2.46% | 0.00% |
| N | 5 | 0.5 | 0.06 | 45 | 97.59% | 0.82% | 1.59% |

**Supplementary Table 16.** Proportions of peak areas of compounds (screening of PAM12B P1 substrates)

| P1  residue | Peptide conc. (mM) | Hydrazine conc. (M) | PAM12B conc. (µM) | Reaction time (min) | Hydrazidation | Hydrolysis | Amide |
| --- | --- | --- | --- | --- | --- | --- | --- |
| L | 5 | 0.5 | 0.06 | 45 | 96.43% | 3.57% | 0.00% |
| Y | 5 | 0.5 | 0.06 | 15 | 94.08% | 1.38% | 4.54% |
| V | 5 | 1.0 | 0.30 | 15 | 98.05% | 1.95% | 0.00% |
| I | 5 | 0.5 | 0.24 | 50 | 97.29% | 2.46% | 0.25% |
| M | 5 | 0.5 | 0.24 | 15 | 92.64% | 7.36% | 0.00% |
| F | 5 | 0.5 | 0.03 | 15 | 96.32% | 3.68% | 0.00% |
| W | 5 | 0.5 | 0.06 | 20 | 96.76% | 3.24% | 0.00% |
| A | 5 | 0.5 | 0.03 | 50 | 90.75% | 9.25% | 0.00% |
| R | 5 | 0.5 | 0.03 | 50 | 97.05% | 2.95% | 0.00% |
| G | 5 | 0.5 | 0.12 | 35 | 90.02% | 9.98% | 0.00% |
| E | 5 | 1.0 | 1.20 | 50 | 90.43% | 4.54% | 5.03% |
| S | 5 | 1.0 | 0.60 | 15 | 84.67% | 15.33% | 0.00% |
| T | 5 | 1.0 | 0.60 | 15 | 88.85% | 11.15% | 0.00% |
| H | 5 | 1.0 | 1.20 | 100 | 96.74% | 3.26% | 0.00% |
| K | 5 | 1.5 | 0.12 | 50 | 97.07% | 2.93% | 0.00% |
| D | 5 | 1.5 | 12.0 | 180 | 88.02% | 10.67% | 1.31% |
| C | 5 | 1.0 | 0.12 | 55 | 87.17% | 10.54% | 2.29% |
| Q | 5 | 1.0 | 0.60 | 5 | 96.17% | 3.83% | 0.00% |
| P | 5 | 1.0 | 12.0 | 180 | 0.00% | 0.00% | 100.00% |
| N | 5 | 1.0 | 0.60 | 5 | - | - | - |

(It was observed that hydrazidation of Ac-DFSKN-NH_2_ took place on both α-amide group and side chain amide group. This by-product was inferred to be derived from the cyclization by-product of α-hydrazide with side chain amide group, which was reported by Fang et al. [4])

## 4.4 Screening of substrate spectrum of PHM and PAL


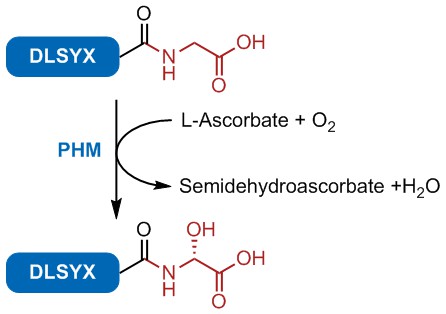

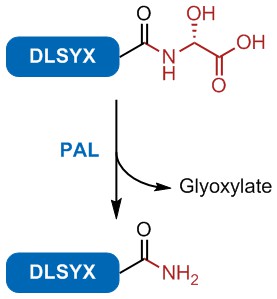


**Supplementary Figure 7.** Reaction route of substrate spectrum screening of PHM and PAL.

1. **Reagent Setup**

**DLSYXG-OH stock solution (5 mM):**

0.5 μmol of DLSYXG-OH was added into a 1.5-mL Eppendorf tube. Then 100 μL of deionized H_2_O was added into the tube to dissolve the peptide.

1. **Reaction conditions**

Initially, 10 µL of DLSYXG-OH stock solution and 25 μL of 0.2 M MES buffer (pH 6.5) were pipetted into the 1.5-mL eppendorf tube. Then 2.5 µL of 0.2 M sodium ascorbate solution, 1 µL of 0.1 mM CuSO_4_ solution, 2 µL of PHM stock solution, 2 µL of PAL stock solution and 7.5 µL of deionized H_2_O were added into the mixture. The amidation reaction was carried out at 37°C for 15 min (4 h for P1 = Cys peptide).

1. **LC-MS analysis**

The reaction mixtures were analyzed with LC-MS general method C. The amidation rates were estimated by integration of the peak areas of peptide amide (A) and remaining peptide carboxylic acid (B) monitored by HPLC (220 nm).

$$Amidation rate \left( \% \right)= \frac{A}{A+B} \times100\%$$

1. **Results**

HPLC traces were shown in **Supplementary HPLC traces and MS figures chapter 4**.

**Supplementary Table 17.** Proportions of peak areas of compounds (screening of PHM & PAL P1 substrates)

| P1  residue | Peptide conc. (mM) | PHM  conc. (µM) | PAL  conc. (µM) | Reaction time (min) | Amidation | Other |
| --- | --- | --- | --- | --- | --- | --- |
| A | 1 | 2.7 | 4.8 | 15 | 100.00% | 0.00% |
| R | 1 | 2.7 | 4.8 | 15 | 100.00% | 0.00% |
| D | 1 | 2.7 | 4.8 | 15 | 100.00% | 0.00% |
| C | 1 | 2.7 | 4.8 | 240 | 100.00% | 0.00% |
| E | 1 | 2.7 | 4.8 | 15 | 100.00% | 0.00% |
| Q | 1 | 2.7 | 4.8 | 15 | 100.00% | 0.00% |
| G | 1 | 2.7 | 4.8 | 15 | 100.00% | 0.00% |
| H | 1 | 2.7 | 4.8 | 15 | 100.00% | 0.00% |
| I | 1 | 2.7 | 4.8 | 15 | 100.00% | 0.00% |
| L | 1 | 2.7 | 4.8 | 15 | 100.00% | 0.00% |
| K | 1 | 2.7 | 4.8 | 15 | 100.00% | 0.00% |
| M | 1 | 2.7 | 4.8 | 15 | 100.00% | 0.00% |
| F | 1 | 2.7 | 4.8 | 15 | 100.00% | 0.00% |
| S | 1 | 2.7 | 4.8 | 15 | 100.00% | 0.00% |
| T | 1 | 2.7 | 4.8 | 15 | 100.00% | 0.00% |
| W | 1 | 2.7 | 4.8 | 15 | 100.00% | 0.00% |
| Y | 1 | 2.7 | 4.8 | 15 | 100.00% | 0.00% |
| V | 1 | 2.7 | 4.8 | 15 | 100.00% | 0.00% |
| P | 1 | 2.7 | 4.8 | 15 | 100.00% | 0.00% |
| N | 1 | 2.7 | 4.8 | 15 | 100.00% | 0.00% |

## 4.5 Cascade reaction of PHM, PAL and PAM12B


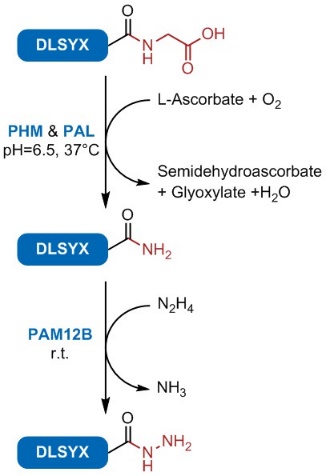


**Supplementary Figure 8.** Reaction route from peptidyl glycine to peptide hydrazide.

1. **Reaction conditions**

Initially, 30 µL of the amidation reaction mixture was transferred into a new 1.5-mL eppendorf tube. Then Y μL of 20 M N_2_H_4_, (5-Y) μL of deionized H_2_O and 5 μL of appropriately diluted PAM12B solution were added into the mixture. The hydrazidation reaction was carried out at room temperature for 5~60 min and monitored by HPLC every 10~15 min.

1. **LC-MS analysis**

The reaction mixtures were analyzed with LC-MS general method C. The hydrazidation rates were estimated by integration of the peak areas of hydrazidation product (A), hydrolysis product (B) and remaining peptide amide (C) monitored by HPLC (220 nm).

$$Hydrazidation rate \left( \% \right)= \frac{A}{A+B+C} \times100\%$$

1. **Results**

HPLC traces were shown in **Supplementary HPLC traces and MS figures chapter 4**.

**Supplementary Table 18.** Proportions of peak areas of compounds (cascade reaction of the three enzymes)

| P1  residue | Peptide conc. (mM) | Hydrazine conc. (M) | PAM12B conc. (µM) | Reaction time (min) | Hydrazidation | Hydrolysis | Amide |
| --- | --- | --- | --- | --- | --- | --- | --- |
| A | 0.75 | 1.0 | 0.16 | 5 | 95.39% | 4.61% | 0.00% |
| R | 0.75 | 1.0 | 0.16 | 20 | 90.89% | 9.11% | 0.00% |
| D | 0.75 | 1.0 | 16.0 | 50 | 94.91% | 5.09% | 0.00% |
| C | 0.75 | 1.5 | 1.60 | 20 | 64.96% | 35.04% | 0.00% |
| E | 0.75 | 1.0 | 16.0 | 60 | 96.43% | 3.57% | 0.00% |
| Q | 0.75 | 1.0 | 0.32 | 30 | 90.19% | 9.81% | 0.00% |
| G | 0.75 | 1.0 | 0.16 | 30 | 95.96% | 4.04% | 0.00% |
| H | 0.75 | 1.0 | 1.60 | 5 | 92.93% | 7.07% | 0.00% |
| I | 0.75 | 1.0 | 0.96 | 30 | 92.22% | 7.78% | 0.00% |
| L | 0.75 | 1.5 | 1.60 | 30 | 92.09% | 7.91% | 0.00% |
| K | 0.75 | 1.0 | 0.96 | 20 | 97.10% | 2.90% | 0.00% |
| M | 0.75 | 1.5 | 1.60 | 30 | 96.46% | 3.54% | 0.00% |
| F | 0.75 | 1.0 | 0.06 | 20 | 90.10% | 9.90% | 0.00% |
| S | 0.75 | 1.0 | 0.16 | 30 | 100.00% | 0.00% | 0.00% |
| T | 0.75 | 1.0 | 0.16 | 30 | 100.00% | 0.00% | 0.00% |
| W | 0.75 | 1.0 | 0.16 | 20 | 91.37% | 8.63% | 0.00% |
| Y | 0.75 | 1.0 | 0.06 | 20 | 94.87% | 5.13% | 0.00% |
| V | 0.75 | 1.0 | 0.32 | 30 | 100.00% | 0.00% | 0.00% |
| P | 0.75 | 1.0 | 16.0 | 60 | 0.00% | 0.00% | 100.00% |
| N | 0.75 | 1.0 | 0.16 | 30 | - | - | - |

(Hydrazidation of Ac-DFSKN-NH_2_ took place on both α-amide group and side chain amide group.)

## 4.6 Conjugation between Ac-DFSKV-G and ALKKA-NH_2_


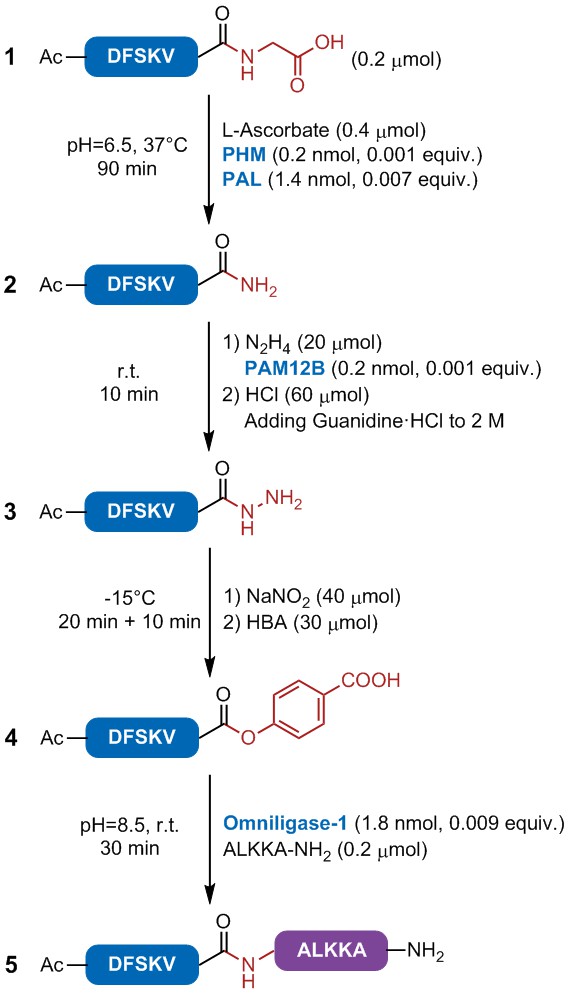


**Supplementary Figure 9.** Reaction route of conjugation between Ac-DFSKV-G and ALKKA-NH_2_.

1. **Reagent Setup**

**Ac-DFSKVG stock solution (20 mM):**

1.4 mg of Ac-DFSKVG was added into a 1.5-mL Eppendorf tube. Then 100 μL of 0.2 M MES buffer solution (pH 6.5) was added into the tube to dissolve the peptide.

**ALKKA-NH_2_ stock solution (40 mM):**

2.1 mg of ALKKA-NH_2_ was added into a 1.5-mL Eppendorf tube. Then 100 μL of deionized H_2_O was added into the tube to dissolve the peptide.

1. **Reaction conditions**

**Amidation:** 10 µL of Ac-DFSKVG stock solution, 2 µL of 0.2 M sodium ascorbate solution, 4 µL of PHM stock solution and 4 µL of PAL stock solution were added into a 1.5-mL eppendorf tube and cultured at 37°C for 90 min.

**Hydrazidation:** To the reaction mixture above, 1 µL of 20 M N_2_H_4_ solution and 3 µL of PAM12B stock solution were added. 10 min later, 5 µL of 12 M HCl solution and 15 µL of 0.2 M phosphate buffer (containing 6 M Gn·HCl, pH 3.0) were added into the mixture to terminate the reaction.

**Esterification:** The reaction mixture was precooled at -15°C for 10 min. Then 8 µL of 5 M NaNO_2_ solution was added into the mixture in batches (8×1 µL, intervals of 1 min). When all of NaNO_2_ solution had been peptited into the tube, the solution and froth were mixed thoroughly and the temperature was kept at -15°C for 10 min. Afterwards, 10 µL of 3 M HBA solution was added and the temperature was kept at -15°C for another 10 min.

**Ligation:** The tube was removed from the reactor and the forth was eliminated by centrifugation (18000 g, 1 min). The pH was adjusted to 8.0~8.5 with 1 M NaOH solution. For ligation, 5 µL of ALKKA-NH_2_ stock solution and 10 µL of Omniligase-1 stock solution were added. The ligation reaction was carried out for 30 min at room temperature.

1. **Results**


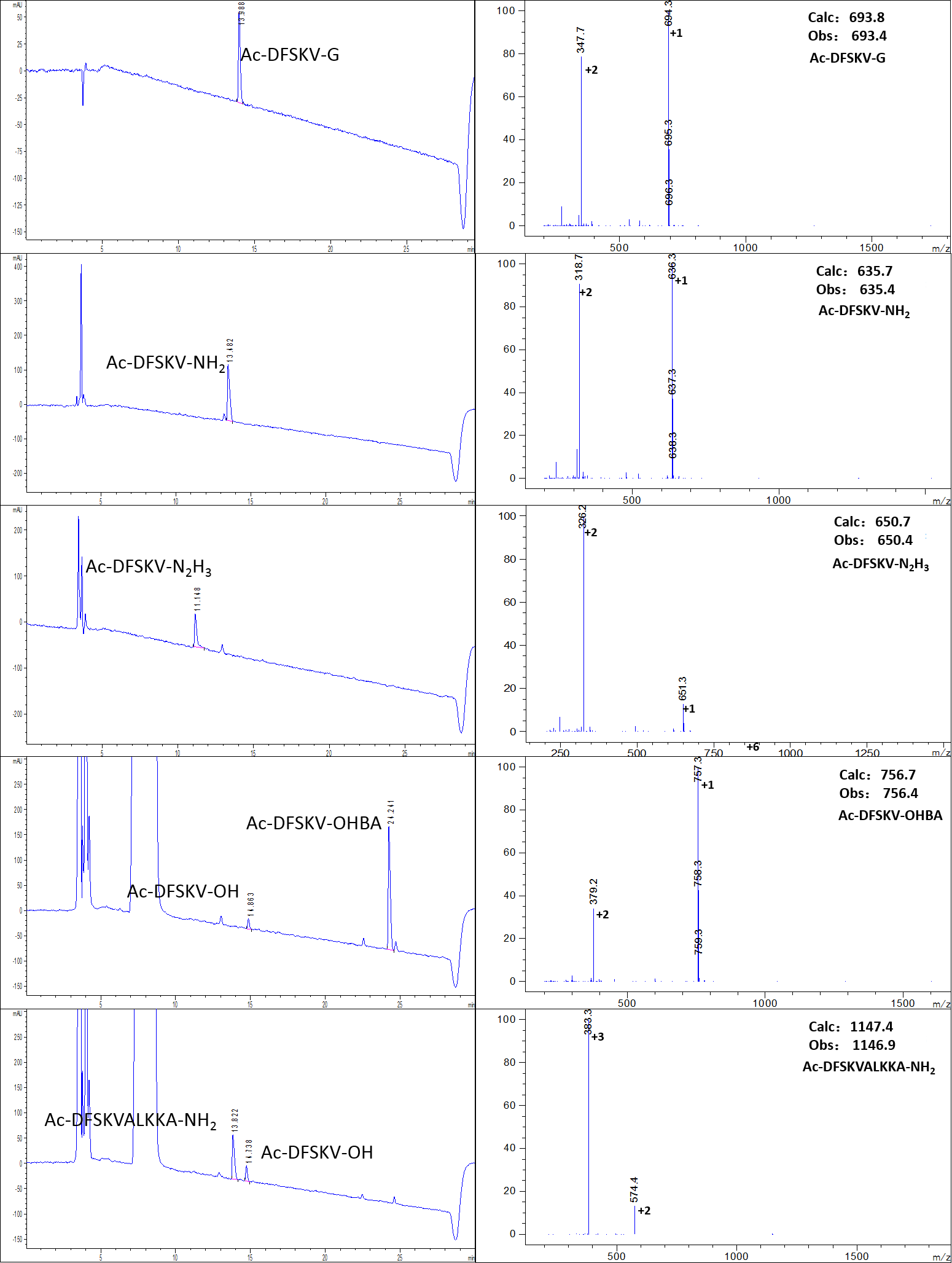


**Supplementary Figure 10.** HPLC traces and mass spectrum of conjugation between Ac-DFSKV-G and ALKKA-NH_2_. (top to bottom) Substrate, after amidation, after hydrazidation, after esterification (pH 8.5), after ligation. Reaction products were analyzed with LC-MS general method A.

## 4.7 Total synthesis of exenatide


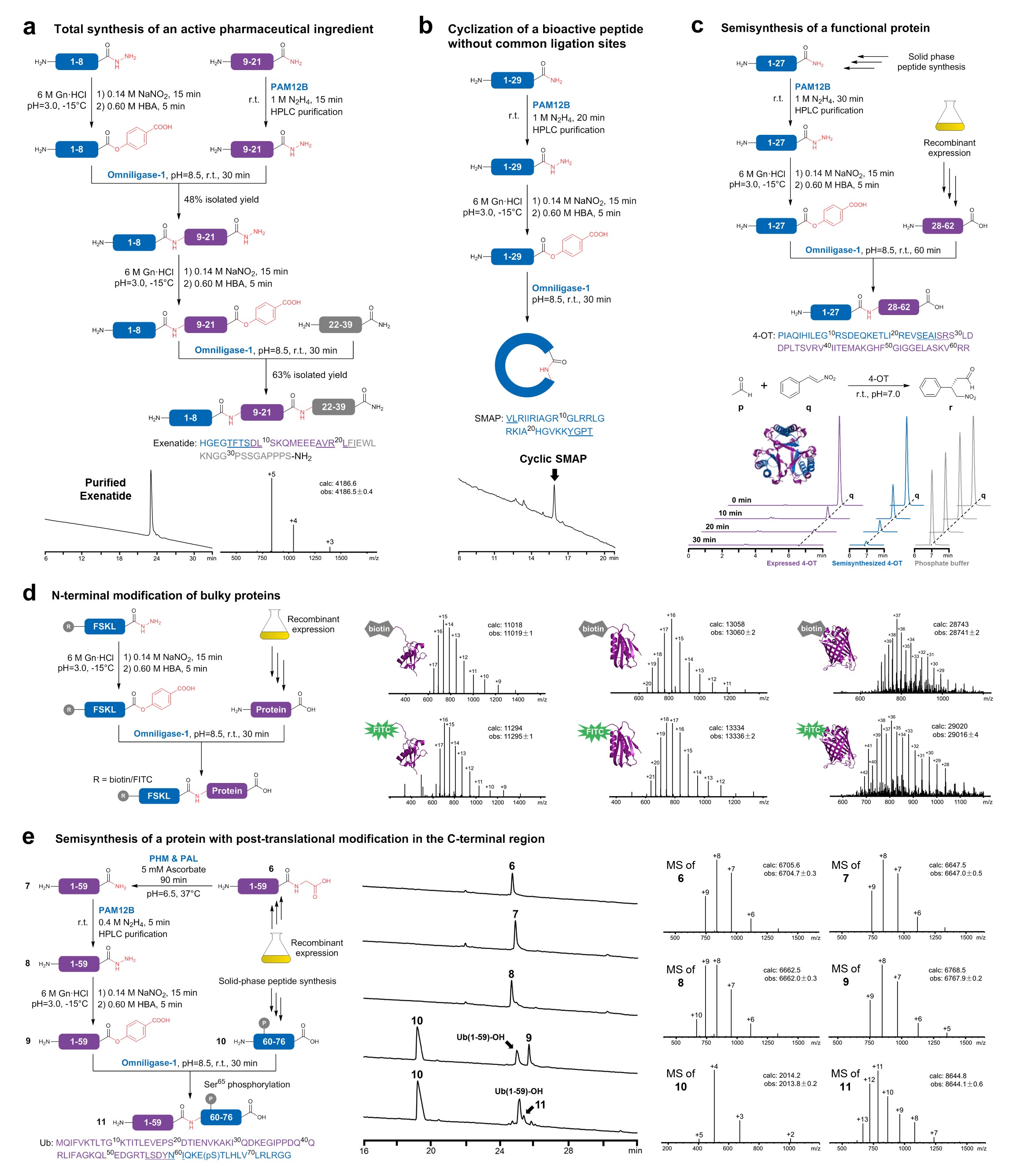


**Supplementary Figure 11.** The reaction route of total synthesis of exenatide.

1. **Synthesis of exenatide-(1-21)-N_2_H_3_**

Initially, 2.0 mg of Ex-(1-8)-N_2_H_3_ and 250 µL of 0.2 M phosphate buffer (containing 6 M Gn·HCl, pH 3.0) were added into the 1.5-mL eppendorf tube and precooled at -15°C for 10 min. Then, 100 µL of 0.5 M NaNO_2_ solution was added into the reaction tube and the temperature was kept at -15°C for 15 min. Next, 150 µL of 2 M HBA stock solution was added into the reaction tube and the temperature was kept at -15°C for another 5 min. Afterwards, the tube was removed from the reactor and allowed to warm to room temperature. The pH was adjusted to 8.0~8.5 with 1 M NaOH solution. For ligation, 7.4 mg of Ex-(9-21)-N_2_H_3_ (2.0 equiv., prepared in Supplementary methods and results chapter 3.3) and 100 µL of Omniligase-1 stock solution were added. The ligation reaction was carried out for 30 min at room temperature. 2.7 mg of purified Ex-(1-21)-N_2_H_3_ was obtained after preparative HPLC purification and lyophilization overnight (approximately 48% isolated yield). Residual acyl acceptor peptide was recycled during preparative HPLC purification (for all preparative-scale cases in this text).

**Supplementary Table 19.** Final concentration of the peptides and Omniligase-1

|  | Ex-(1-8) | Ex-(9-21) | Omniligase-1 |
| --- | --- | --- | --- |
| Concentration (mM) | 8 | 16 | 0.03 |

1. **Synthesis of full-length exenatide**

Initially, 2.7 mg of Ex-(1-21)-N_2_H_3_ and 125 µL of 0.2 M phosphate buffer (containing 6 M Gn·HCl, pH 3.0) were added into the 1.5-mL eppendorf tube and precooled at -15°C for 10 min. Then, 50 µL of 0.5 M NaNO_2_ solution was added into the reaction tube and the temperature was kept at -15°C for 15 min. Next, 75 µL of 2 M HBA stock solution was added into the reaction tube and the temperature was kept at -15°C for another 5 min. Afterwards, the tube was removed from the reactor and allowed to warm to room temperature. The pH was adjusted to 8.0~8.5 with 1 M NaOH solution. For ligation, 3.4 mg of Ex-(22-39)-NH_2_ (1.6 equiv.) and 50 µL of Omniligase-1 stock solution were added. The ligation reaction was carried out for 30 min at room temperature. 3.0 mg of purified full-length exenatide was obtained after preparative HPLC purification and lyophilization overnight (approximately 63% isolated yield).

**Supplementary Table 20.** Final concentration of the peptides and Omniligase-1

|  | Ex-(1-21) | Ex-(22-39) | Omniligase-1 |
| --- | --- | --- | --- |
| Concentration (mM) | 4.2 | 6.7 | 0.03 |


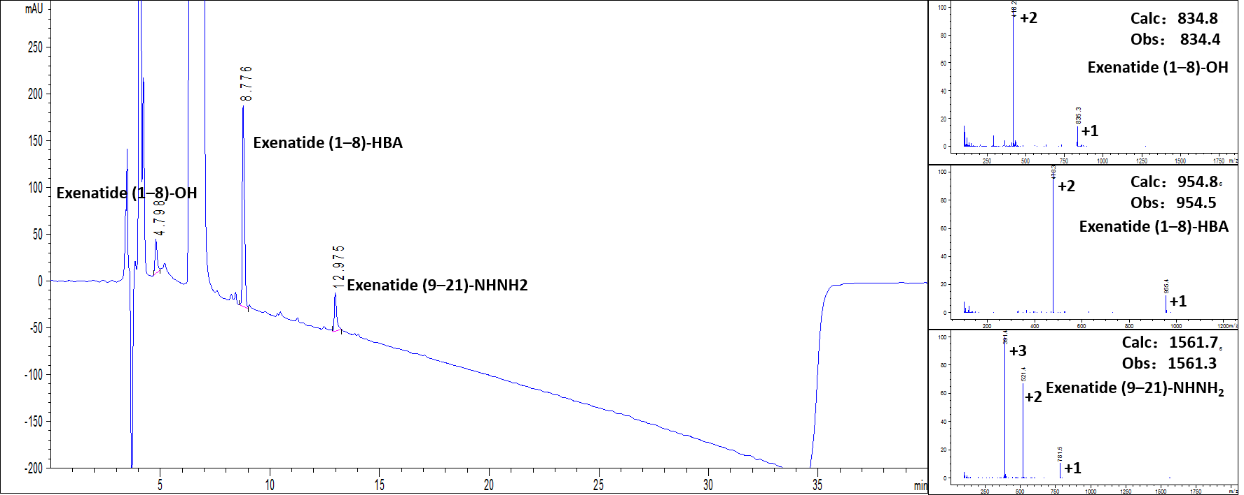

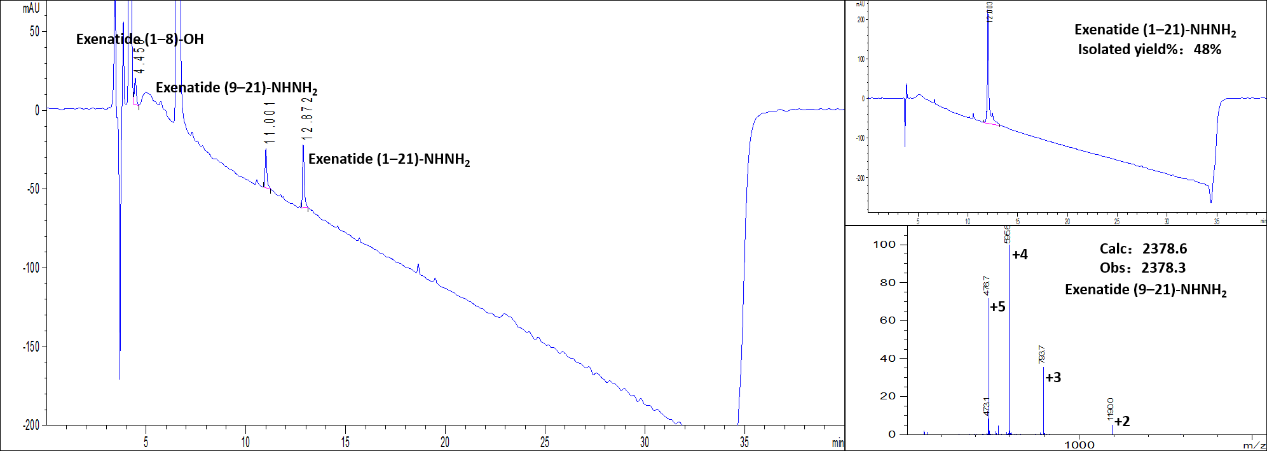


Before the addition

of Omniligase-1

Ligation for 30 min

**Supplementary Figure 12.** LC-MS spectrum of the 1st ligation for total synthesis of exenatide.


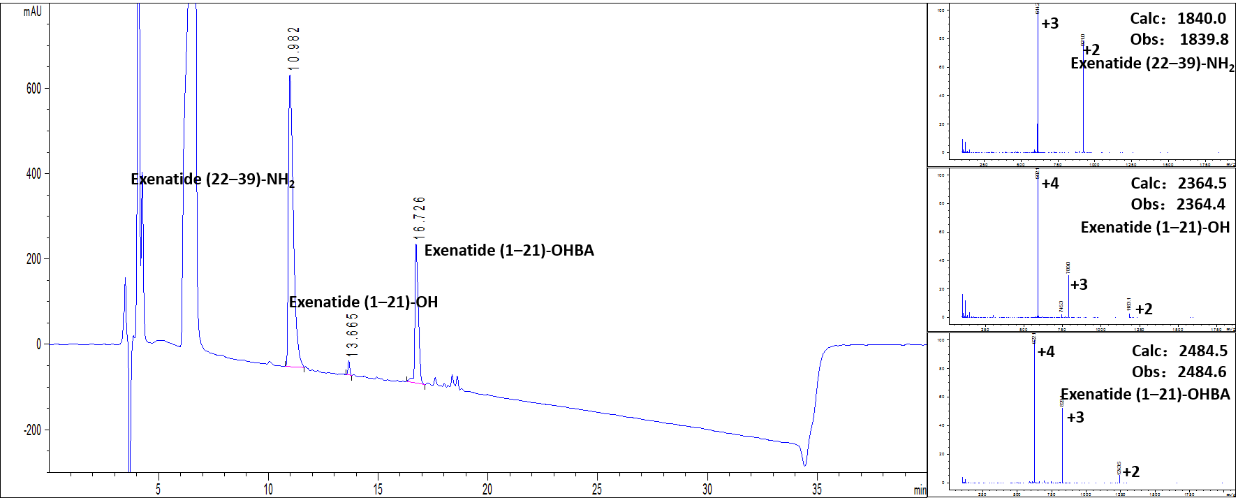

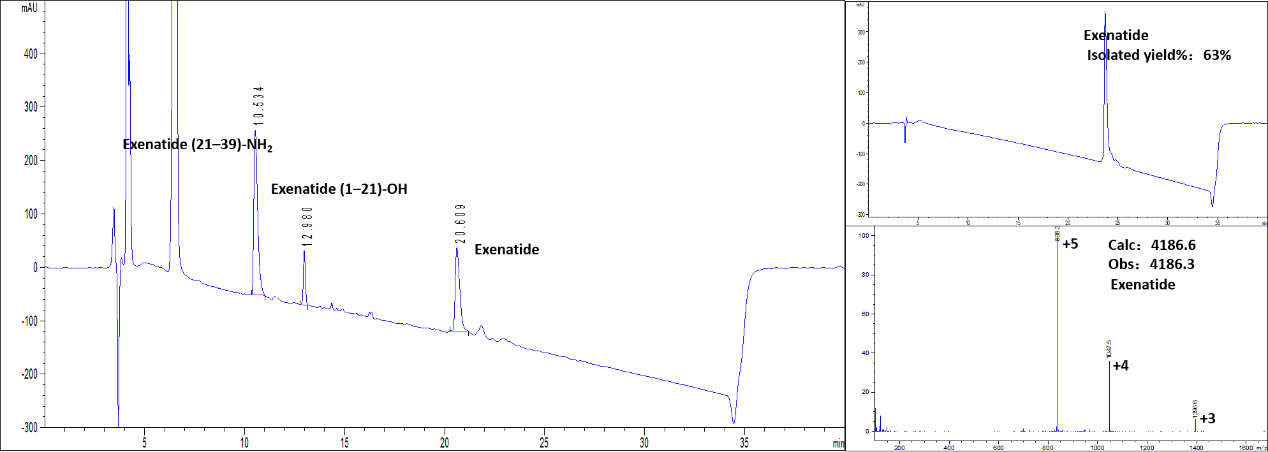


Before the addition

of Omniligase-1

Ligation for 30 min

**Supplementary Figure 13.** LC-MS spectrum of the 2nd ligation for total synthesis of exenatide.

## 4.8 Cyclization of sheep myeloid antimicrobial peptide


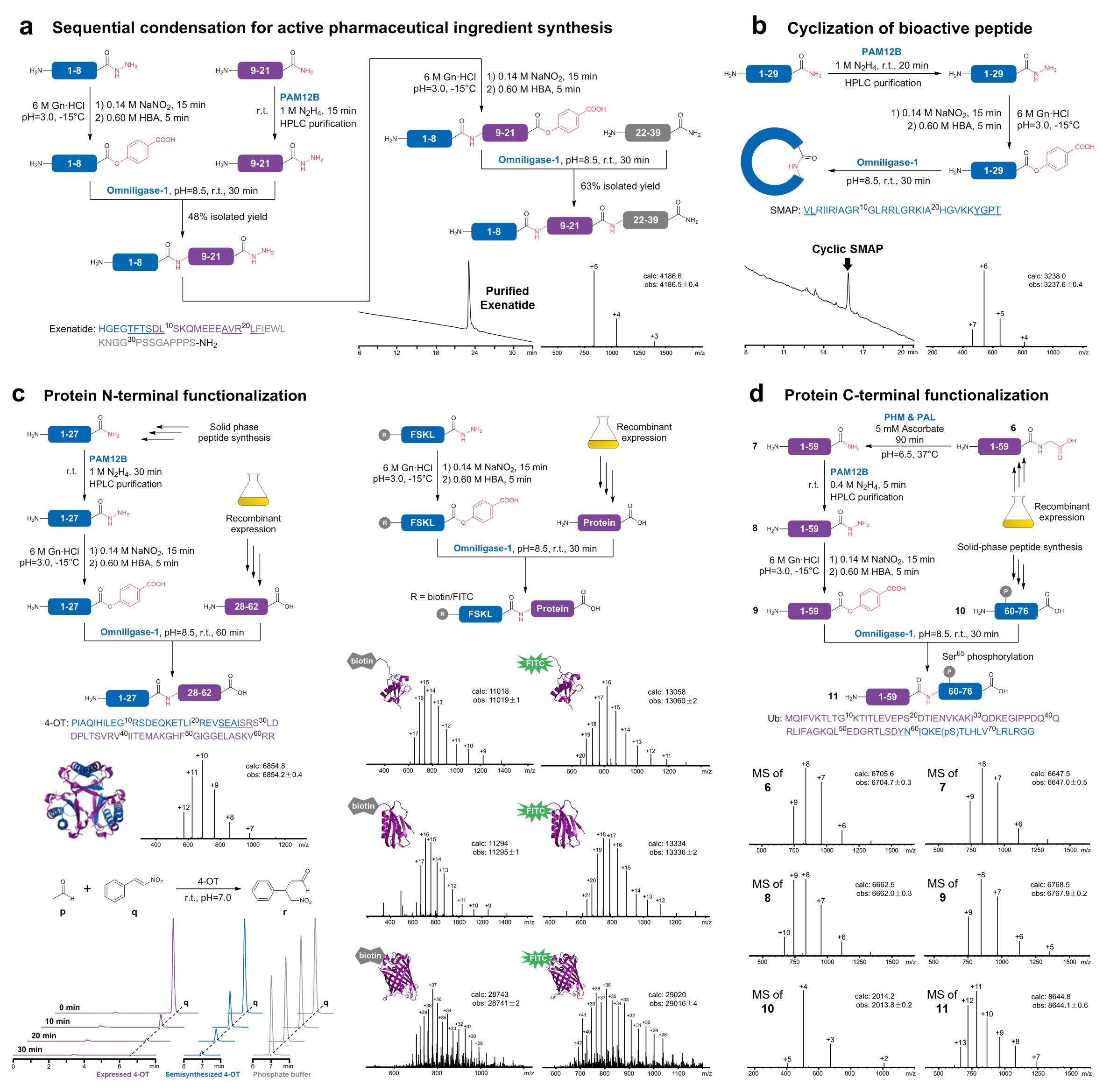


**Supplementary Figure 14.** Reaction route of cyclization of sheep myeloid antimicrobial peptide (SMAP).

Initially, 0.4 mg of SMAP-N_2_H_3_ (prepared in Supplementary methods and results chapter 3.3) and 12.5 µL of 0.2 M phosphate buffer (containing 6 M Gn·HCl, pH 3.0) were added into the 1.5-mL eppendorf tube and precooled at -15°C for 10 min. Then, 5.0 µL of 0.5 M NaNO_2_ solution was added into the reaction tube and the temperature was kept at -15°C for 15 min. Next, 7.5 µL of 2 M HBA stock solution was added into the reaction tube and the temperature was kept at -15°C for another 5 min. Afterwards, the tube was removed from the reactor and allowed to warm to room temperature. 1 µL of the reaction mixture and 29 µL of 0.05 M Phosphate buffer solution (pH 7.5) were transferred into a new 1.5-mL eppendorf tube, and the pH was adjusted to 8.0~8.5 with 1 M NaOH solution. For ligation, 5 µL of Omniligase-1 stock solution was added. The ligation reaction was carried out for 30 min at room temperature. Cyclization product was obtained in an assumed HPLC yield of 86% by integration of the peak areas of cyclization product, hydrolysis product and chemical by-product (likely due to Curtius rearrangement of the peptide azide) monitored by HPLC (220 nm).

**Supplementary Table 21.** Final concentration of the peptide and Omniligase-1

|  | SMAP | Omniligase-1 |
| --- | --- | --- |
| Concentration (mM) | 0.1 | 0.03 |

**
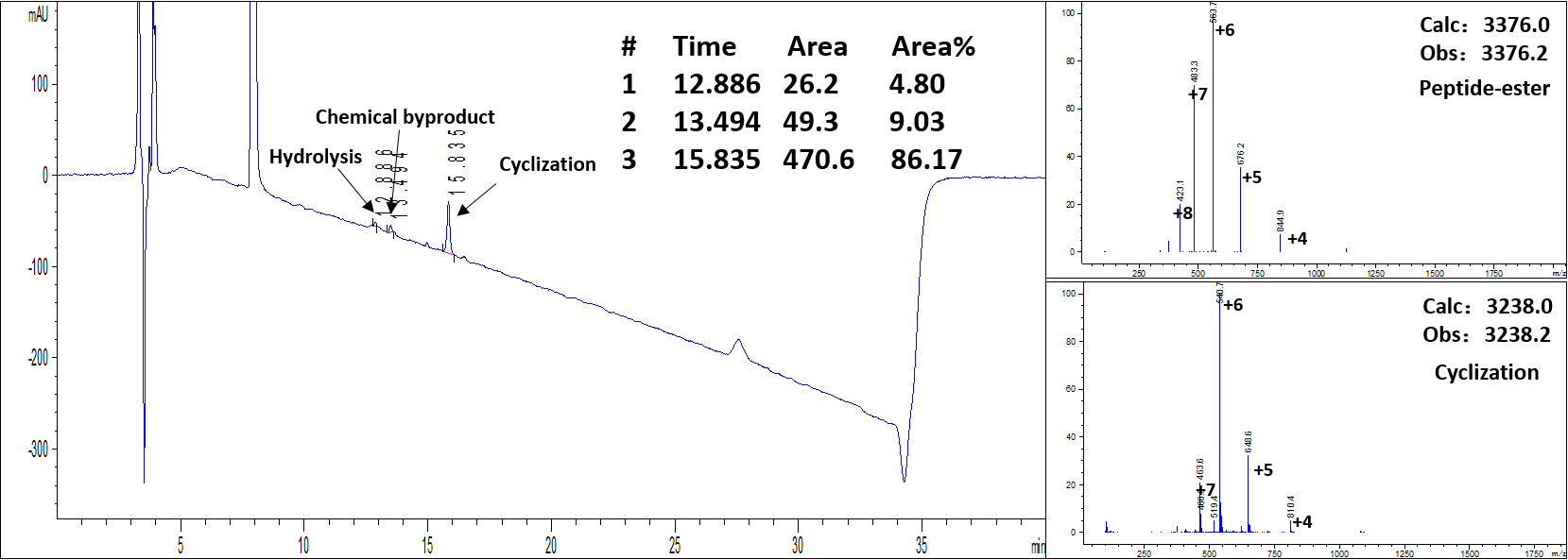
**

**Supplementary Figure 15.** LC-MS spectrum of cyclization of SMAP.

## 4.9 Semisynthesis of 4-oxalocrotonate tautomerase


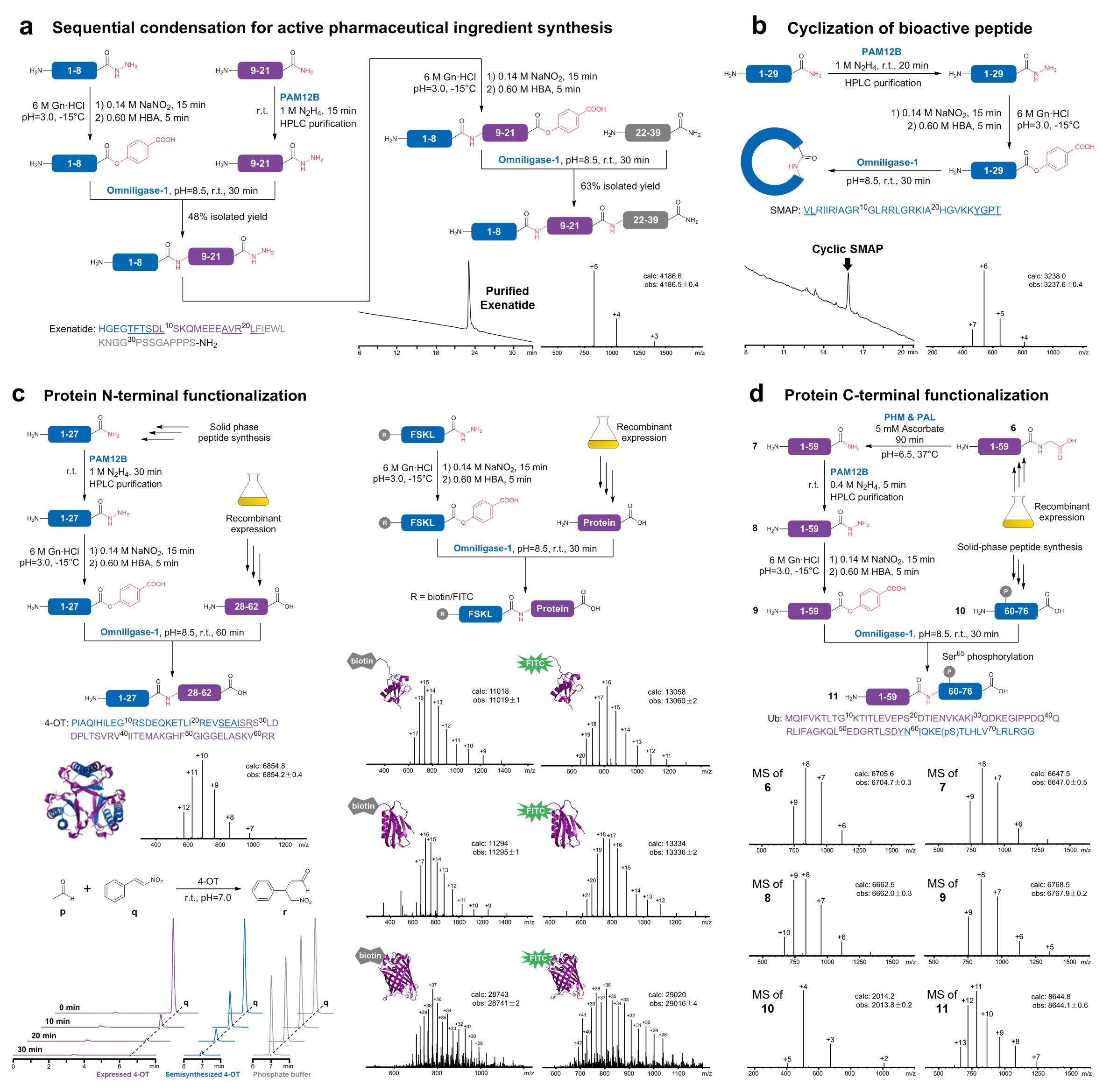


**Supplementary Figure 16.** Reaction route of semisynthesis of 4-oxalocrotonate tautomerase (4-OT).

1. **Semisynthesis of full-length 4-OT**

Initially, 0.3 mg of 4-OT-(1-27)-N_2_H_3_ (prepared in Supplementary methods and results chapter 3.3) and 50 µL of 0.2 M phosphate buffer (containing 6 M Gn·HCl, pH 3.0) were added into the 1.5-mL eppendorf tube and precooled at -15°C for 10 min. Then, 20 µL of 0.5 M NaNO_2_ solution was added into the reaction tube and the temperature was kept at -15°C for 15 min. Next, 30 µL of 2 M HBA stock solution was added into the reaction tube and the temperature was kept at -15°C for another 5 min. Afterwards, the tube was removed from the reactor and allowed to warm to room temperature. The pH was adjusted to 8.0~8.5 with 1 M NaOH solution. For ligation, 0.4 mg of 4-OT-(28-62)-OH (1.1 equiv.) and 20 µL of Omniligase-1 stock solution were added. The ligation reaction was carried out for 60 min at room temperature. 0.2 mg of purified full-length 4-OT was obtained after preparative HPLC purification and lyophilization overnight.

**Supplementary Table 22.** Final concentration of the peptides and Omniligase-1

|  | 4-OT-(1-27) | 4-OT-(28-62) | Omniligase-1 |
| --- | --- | --- | --- |
| Concentration (mM) | 0.8 | 0.9 | 0.03 |

1. **Reaction conditions of Michael-type addition of acetaldehyde to nitroalkene**

**Enzyme preparation:**

0.2 mg of semisynthesized 4-OT was dissolved in 50 µL of 0.2 M phosphate buffer (containing 6 M Gn·HCl, pH 7.0), then gradually diluted with 0.05 M phosphate buffer (pH 7.0) to 0.2 mg/mL. After centrifugation (18000 g, 5 min) the supernatant was used for activity test. Recombinant 4-OT (containing His-tag at C-terminus) was prepared as the protocol for expression of PAM12B.

**Reaction conditions:**

80 µL of 0.05 M phosphate buffer (pH 7.0) was pipetted into a 1.5-mL eppendorf tube. Then 10 µL of 500 mM acetaldehyde solution, 5 µL of 20 mM trans-β-Nitrostyrene and 5 µL of ~0.2 mg/mL 4-OT solution were added into the tube. The reaction was carried out at room temperature and monitored by HPLC (320 nm) every 10 min. HPLC method: C18 reversed-phase column (4.6×250 mm, pore size: 100 Å, particle size 5 μm, Nanochrom, Suzhou, China); temperature: 25°C; eluent A: 0.1% formic acid in water, eluent B: acetonitrile, A:B=65:35; flow rate: 1 mL·min^−1^.


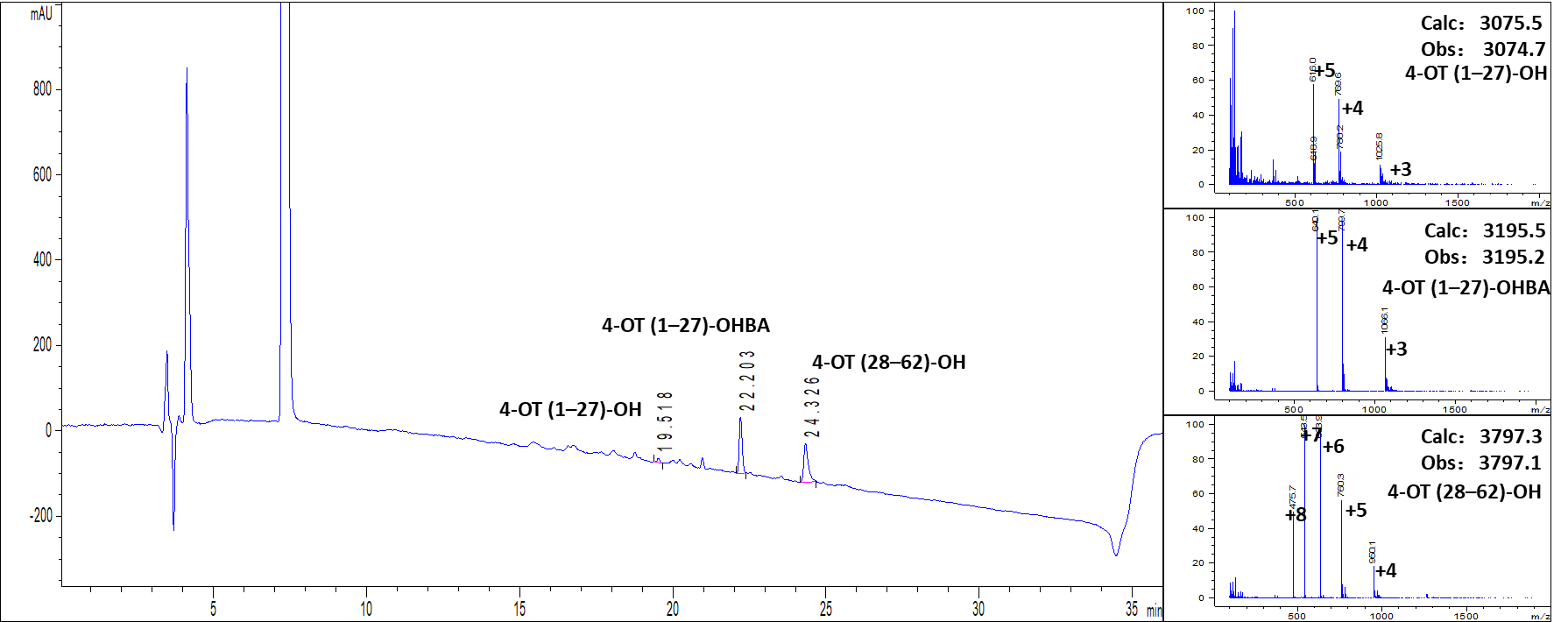

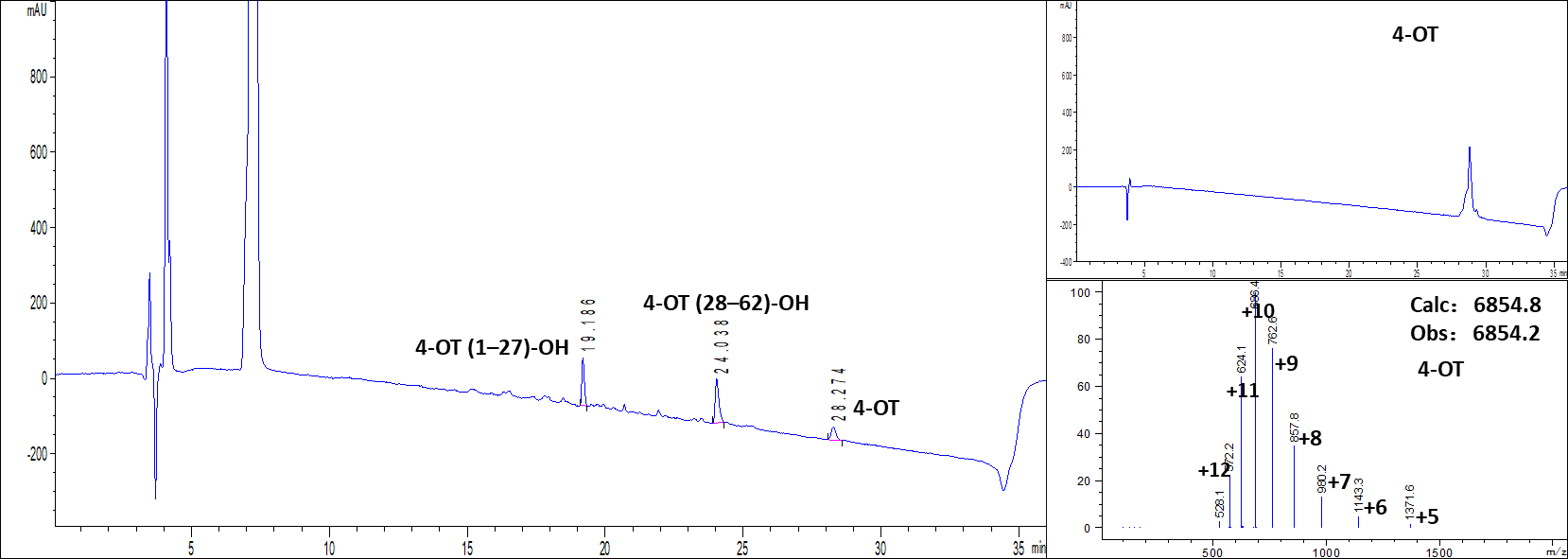


Before the addition

of Omniligase-1

Ligation for 60 min

**Supplementary Figure 17.** LC-MS spectrum of semisynthesis of 4-OT.

## 4.10 Total synthesis of 4-oxalocrotonate tautomerase

**
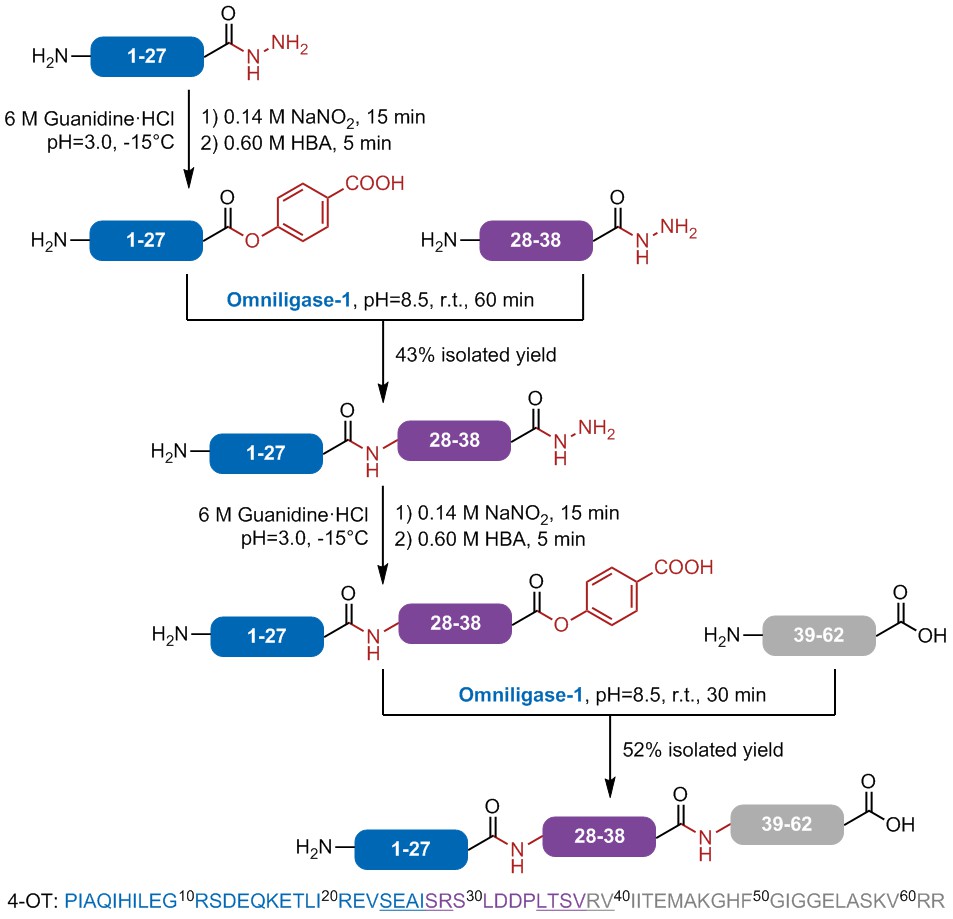
**

**Supplementary Figure 18.** Reaction route of total synthesis of 4-OT.

1. **Synthesis of 4-OT-(1-38)-N_2_H_3_**

Initially, 2.0 mg of 4-OT-(1-27)-N_2_H_3_ and 250 µL of 0.2 M phosphate buffer (containing 6 M Gn·HCl, pH 3.0) were added into the 1.5-mL eppendorf tube and precooled at -15°C for 10 min. Then, 100 µL of 0.5 M NaNO_2_ solution was added into the reaction tube and the temperature was kept at -15°C for 15 min. Next, 150 µL of 2 M HBA stock solution was added into the reaction tube and the temperature was kept at -15°C for another 5 min. Afterwards, the tube was removed from the reactor and allowed to warm to room temperature. The pH was adjusted to 8.0~8.5 with 1 M NaOH solution. For ligation, 1.4 mg of 4-OT-(28-38)-N_2_H_3_ (1.8 equiv.) and 100 µL of Omniligase-1 stock solution were added. The ligation reaction was carried out for 60 min at room temperature. 1.2 mg of purified 4-OT-(1-38)-N_2_H_3_ was obtained after preparative HPLC purification and lyophilization overnight (approximately 43% isolated yield).

**Supplementary Table 23.** Final concentration of the peptides and Omniligase-1

|  | 4-OT-(1-27)-N_2_H_3_ | 4-OT-(28-38)-N_2_H_3_ | Omniligase-1 |
| --- | --- | --- | --- |
| Concentration (mM) | 1.1 | 2.0 | 0.03 |

1. **Synthesis of full-length 4-OT**

Initially, 1.2 mg of 4-OT-(1-38)-N_2_H_3_ and 100 µL of 0.2 M phosphate buffer (containing 6 M Gn·HCl, pH 3.0) were added into the 1.5-mL eppendorf tube and precooled at -15°C for 10 min. Then, 40 µL of 0.5 M NaNO_2_ solution was added into the reaction tube and the temperature was kept at -15°C for 15 min. Next, 60 µL of 2 M HBA stock solution was added into the reaction tube and the temperature was kept at -15°C for another 5 min. Afterwards, the tube was removed from the reactor and allowed to warm to room temperature. The pH was adjusted to 8.0~8.5 with 1 M NaOH solution. For ligation, 1.5 mg of 4-OT-(39-62)-OH (2.0 equiv.) and 50 µL of Omniligase-1 stock solution were added. The ligation reaction was carried out for 30 min at room temperature. 1.0 mg of purified full-length 4-OT was obtained after preparative HPLC purification and lyophilization overnight (approximately 52% isolated yield).

**Supplementary Table 24.** Final concentration of the peptides and Omniligase-1

|  | 4OT-(1-38)-N_2_H_3_ | 4OT-(39-62)-OH | Omniligase-1 |
| --- | --- | --- | --- |
| Concentration (mM) | 1.1 | 2.2 | 0.03 |


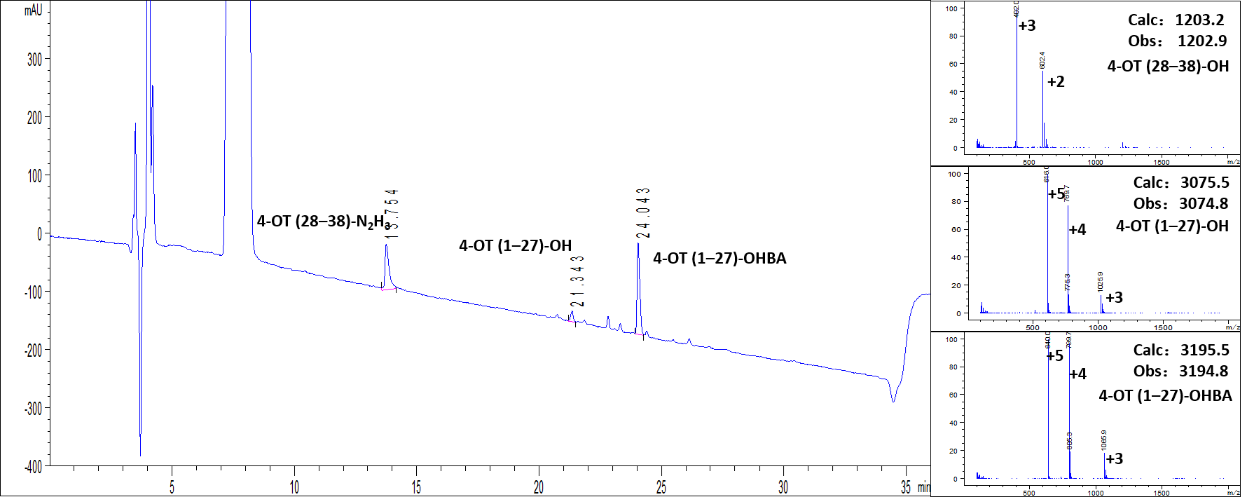

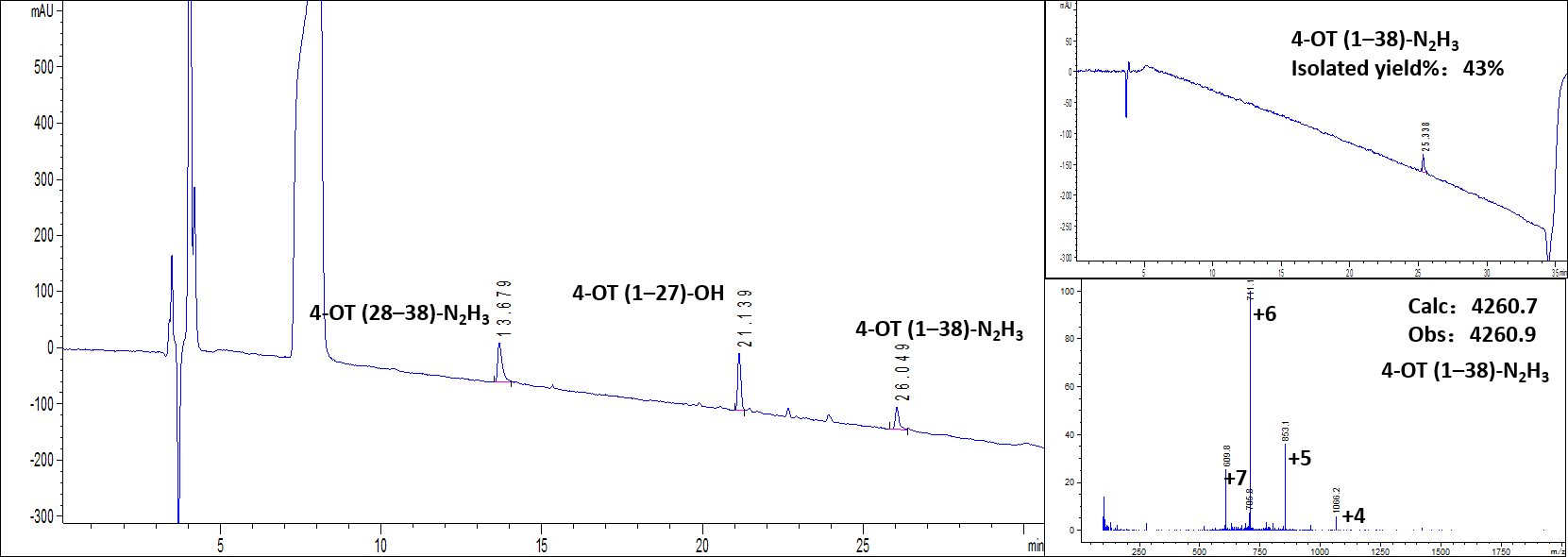


Before the addition

of Omniligase-1

Ligation for 60 min

**Supplementary Figure 19.** LC-MS spectrum of the 1st ligation for total synthesis of 4-OT.


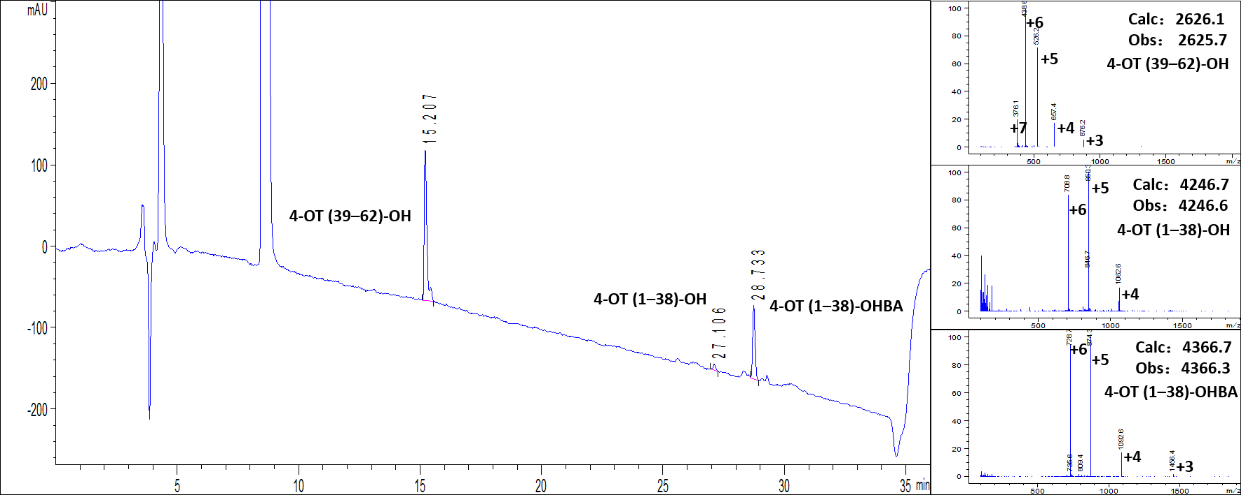

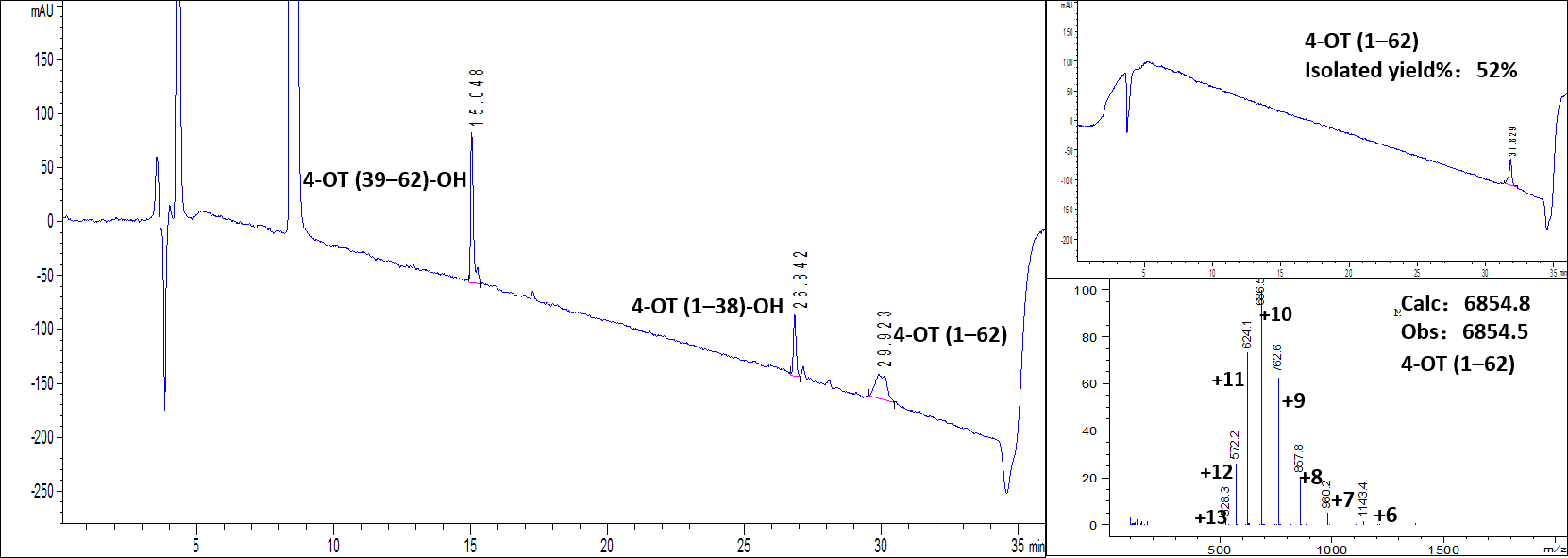


Before the addition

of Omniligase-1

Ligation for 30 min

**Supplementary Figure 20.** LC-MS spectrum of the 2nd ligation for total synthesis of 4-OT.

## 4.11 N-terminal modification of FAT10, C4S3 and EGFP


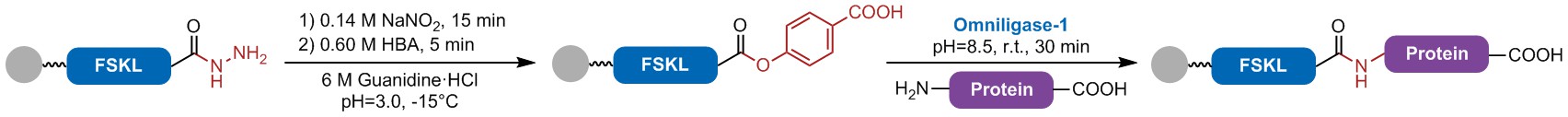


**Supplementary Figure 21.** Reaction route of N-terminal modification of the recombinant proteins.

Initially, 1.1 mg of Biotin-FSKL-N_2_H_3_ (or 1.5 mg FITC-FSKL-N_2_H_3_) and 50 µL of 0.2 M phosphate buffer (containing 6 M Gn·HCl, pH 3.0) were added into the 1.5-mL eppendorf tube and precooled at -15°C for 10 min. Then, 20 µL of 0.5 M NaNO_2_ solution was added into the reaction tube and the temperature was kept at -15°C for 15 min. Next, 30 µL of 2 M HBA stock solution was added into the reaction tube and the temperature was kept at -15°C for another 5 min. Afterwards, the tube was removed from the reactor and allowed to warm to room temperature. The pH was adjusted to 8.0~8.5 with 1 M NaOH solution. For ligation, 10 µL of the reaction mixture was transferred into a new 1.5-mL eppendorf tube, and 15 µL of the recombinant protein solution (~1 mM) and 5 µL of Omniligase-1 stock solution were added.The ligation reaction was carried out for 30 min at room temperature.

**Supplementary Table 25.** Final concentration of the peptides and Omniligase-1

|  | Biotin/FITC-FSKL | Recombinant protein | Omniligase-1 |
| --- | --- | --- | --- |
| Concentration (mM) | 5.0 | 0.5 (0.25 for EGFP) | 0.03 |


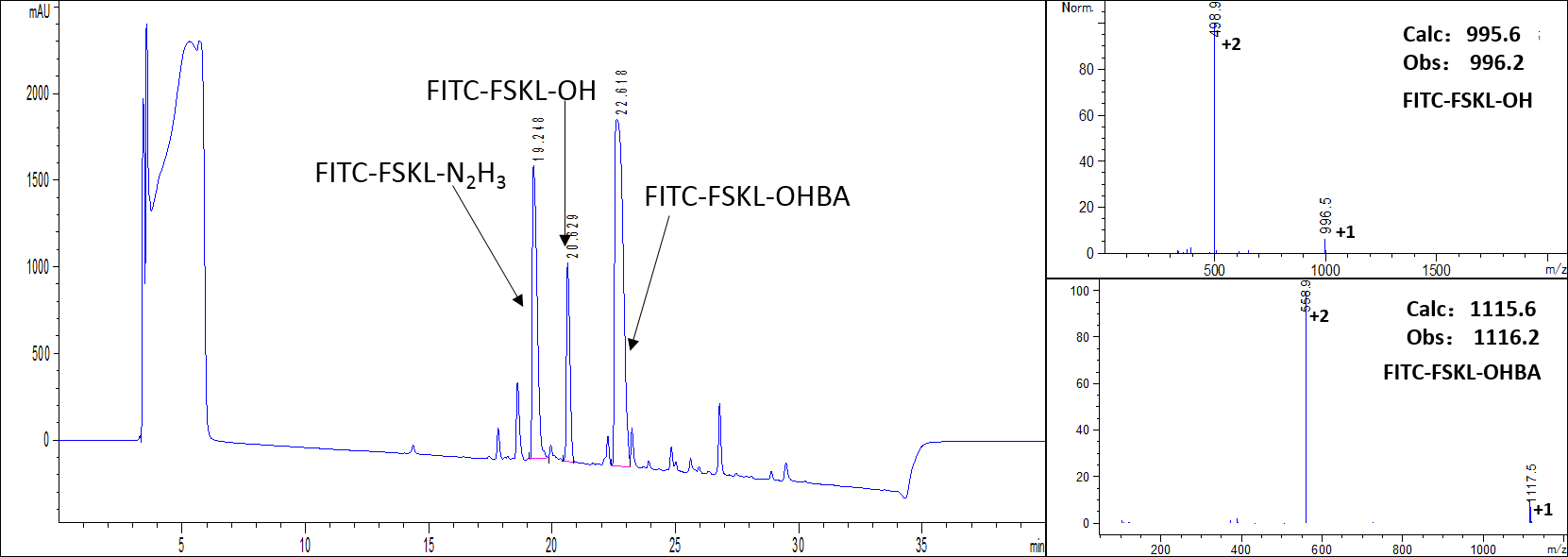

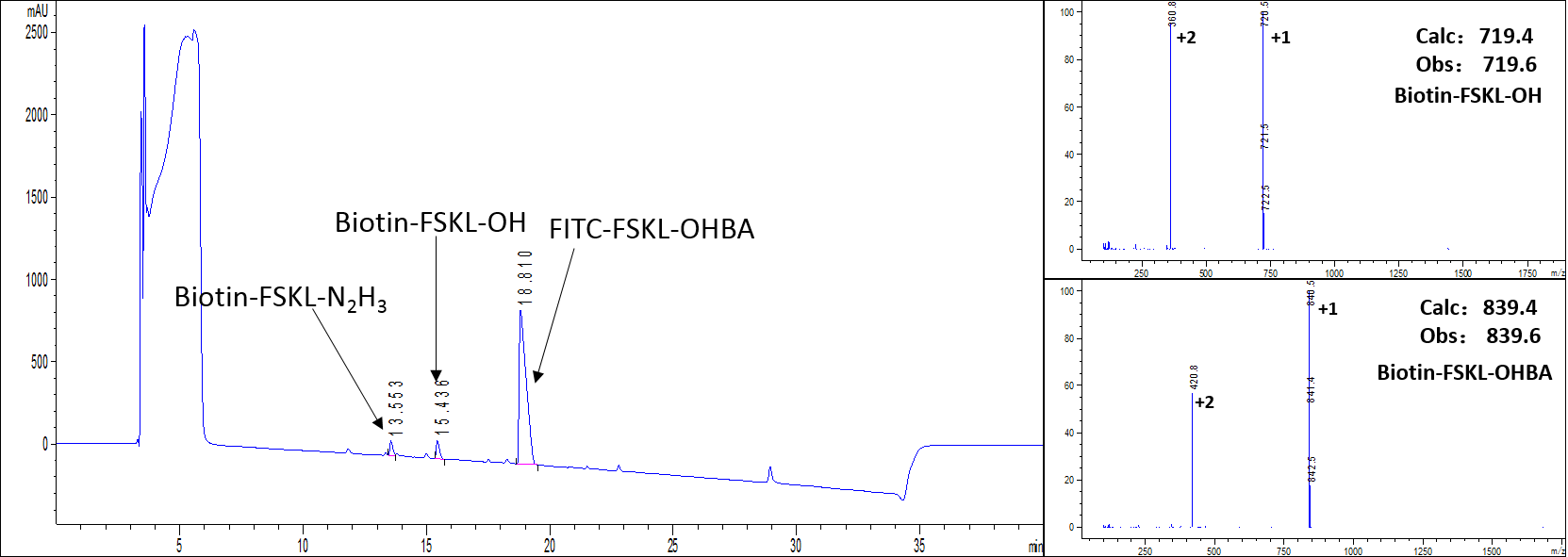


**Supplementary Figure 22.** LC-MS spectrum of modified peptide esters.


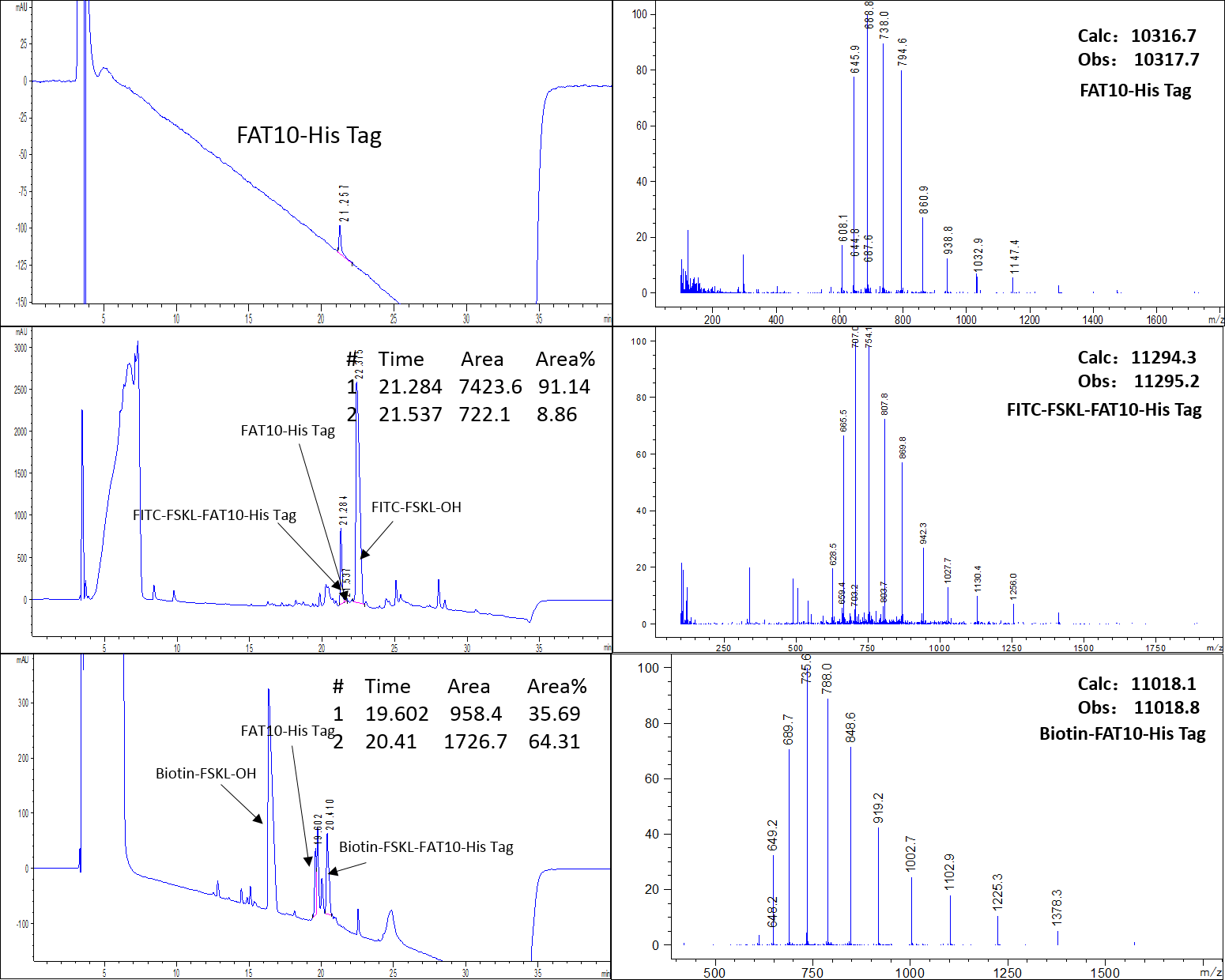


**Supplementary Figure 23.** LC-MS spectrum of N-terminal modification of FAT10.


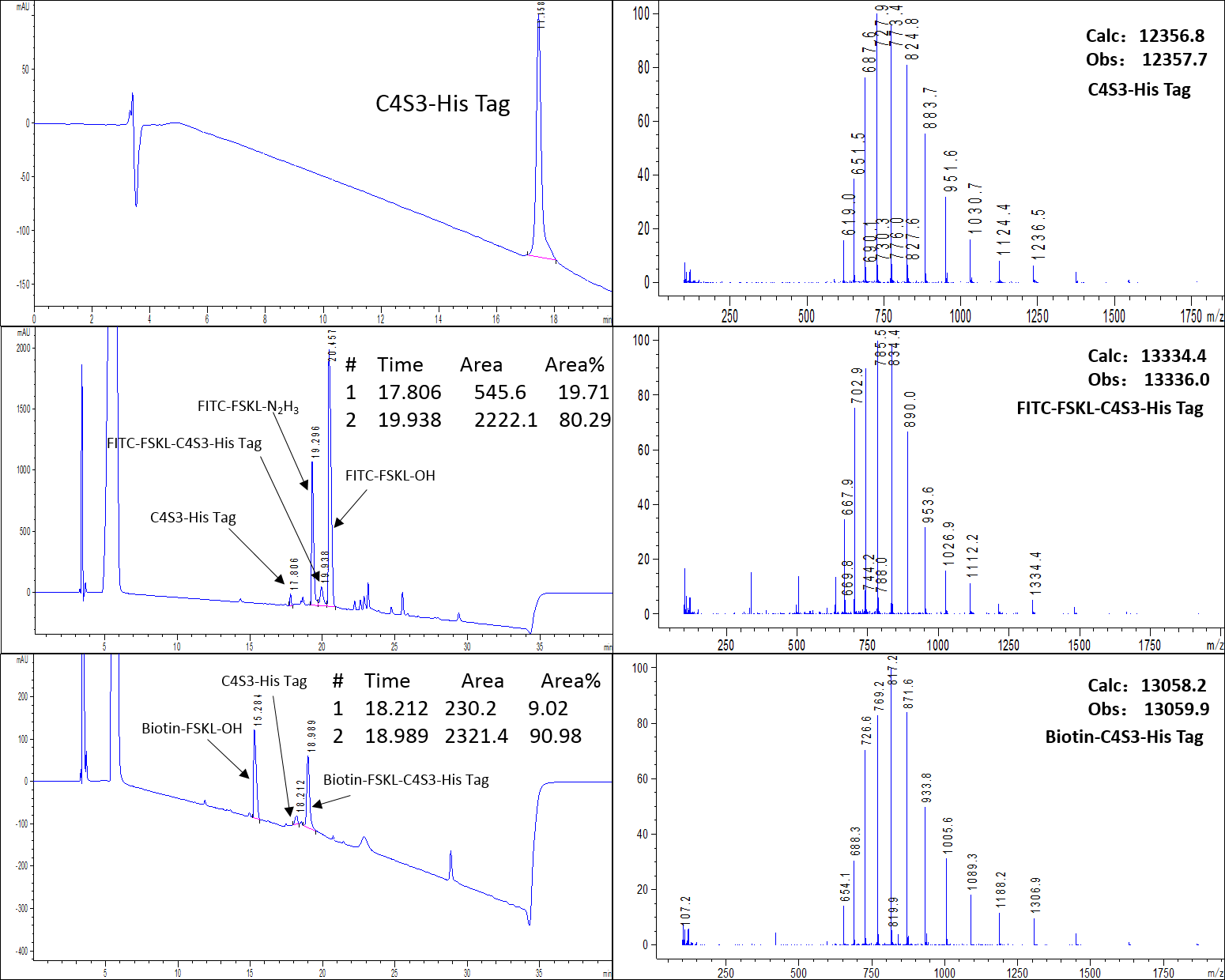


**Supplementary Figure 24.** LC-MS spectrum of N-terminal modification of C4S3.


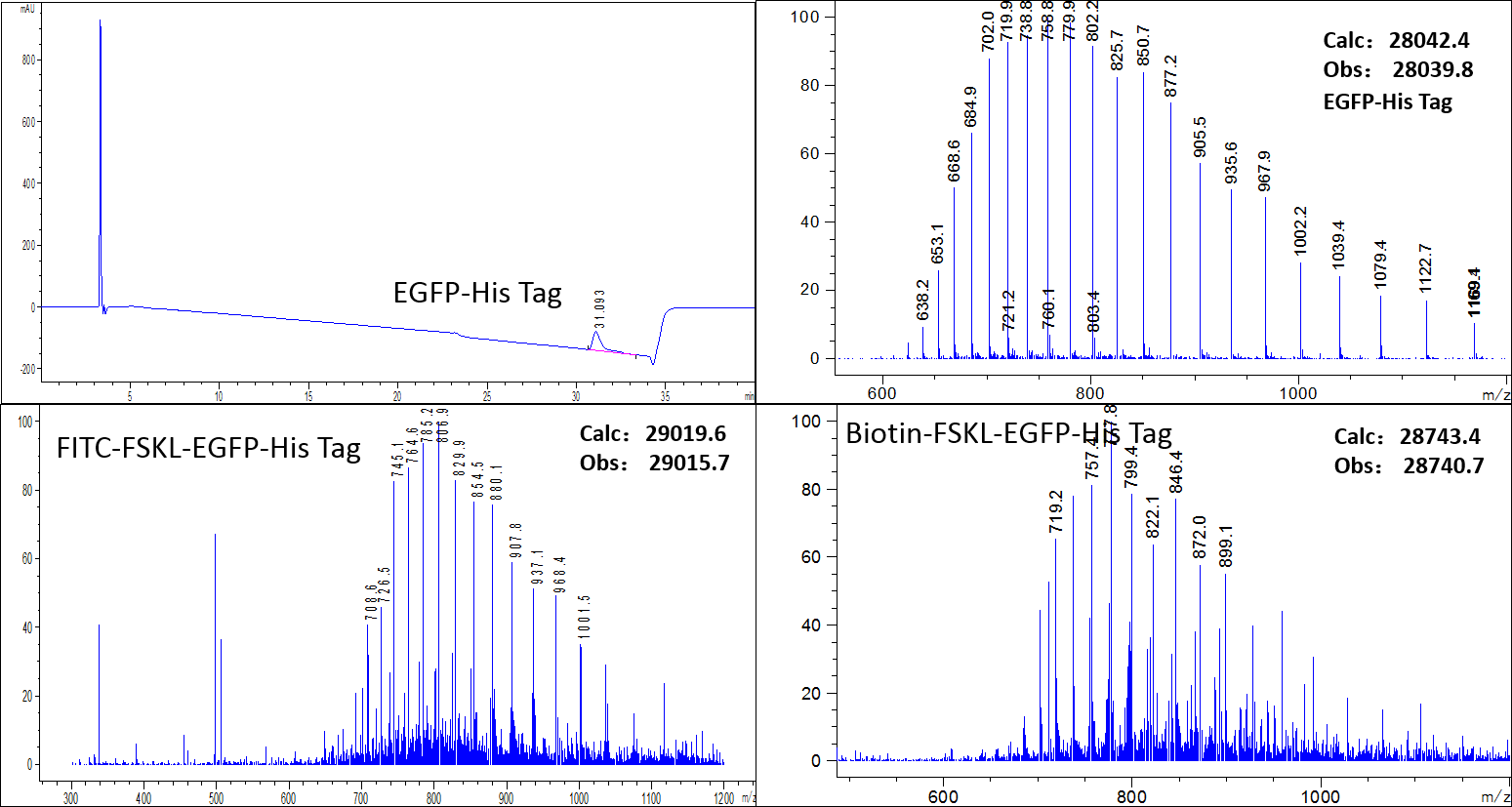


**Supplementary Figure 25.** LC-MS spectrum of N-terminal modification of EGFP.

## 4.12 Semisynthesis of pSer65-ubiquitin by expressed protein ligation

**
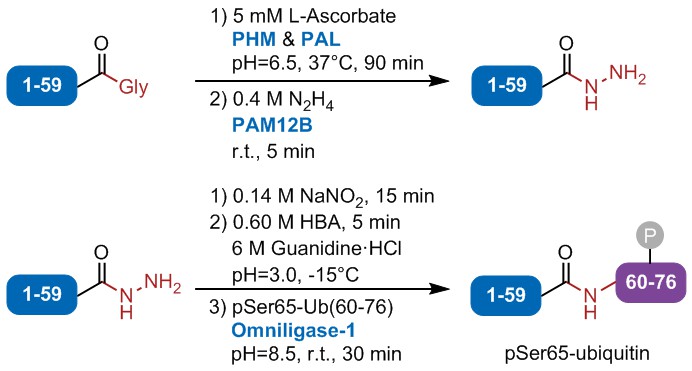
**

**Supplementary Figure 26.** Reaction route of semisynthesis of pSer65-Ub by expressed protein ligation.

1. **Reaction conditions**

200 µL of Ub(1-59)-G solution (~10 mg/mL) was pipetted into a 15-mL culture tube with dual position cap. Then 5 µL of 0.2 M L-ascorbate solution, 20 µL of PHM stock solution (~0.004 equiv.) and 20 µL of PAL stock solution (~0.02 equiv.) were added into the reaction mixture and the tube was incubated in an air bath shaker at 37°C, 180 rpm. 90 min later, 5 µL of 20 M N_2_H_4_ solution and 15 µL of PAM12B stock solution (~0.007 equiv.) were added into the mixture. Hydrazidation reaction was carried out at room temperature for 5 min, then the mixture was injected into preparative HPLC equipments to purify the protein hydrazide.

After lyophilization overnight, the isolated protein was dissolved in 50 µL of 0.2 M phosphate buffer (containing 6 M Gn·HCl, pH 3.0) and precooled at -15°C for 10 min. Then, 20 µL of 0.5 M NaNO_2_ solution was added into the reaction tube and the temperature was kept at -15°C for 15 min. Next, 30 µL of 2 M HBA stock solution was added into the reaction tube and the temperature was kept at -15°C for another 5 min. Afterwards, the tube was removed from the reactor and allowed to warm to room temperature. The pH was adjusted to 8.0~8.5 with 1 M NaOH solution. For ligation, 50 µL of the reaction mixture was transferred into a new 1.5-mL eppendorf tube, and 1.5 mg of pSer65-Ub(60-76) and 10 µL of Omniligase-1 stock solution were added. The ligation reaction was carried out for 30 min at room temperature.

**Supplementary Table 26.** Final concentration of the peptides and Omniligase-1

|  | Ub(1-59) | Ub(60-76) | Omniligase-1 |
| --- | --- | --- | --- |
| Concentration (mM) | 2.5 | 12.5 | 0.03 |

1. **Results**

Reaction mixtures were analyzed with LC-MS General method A.
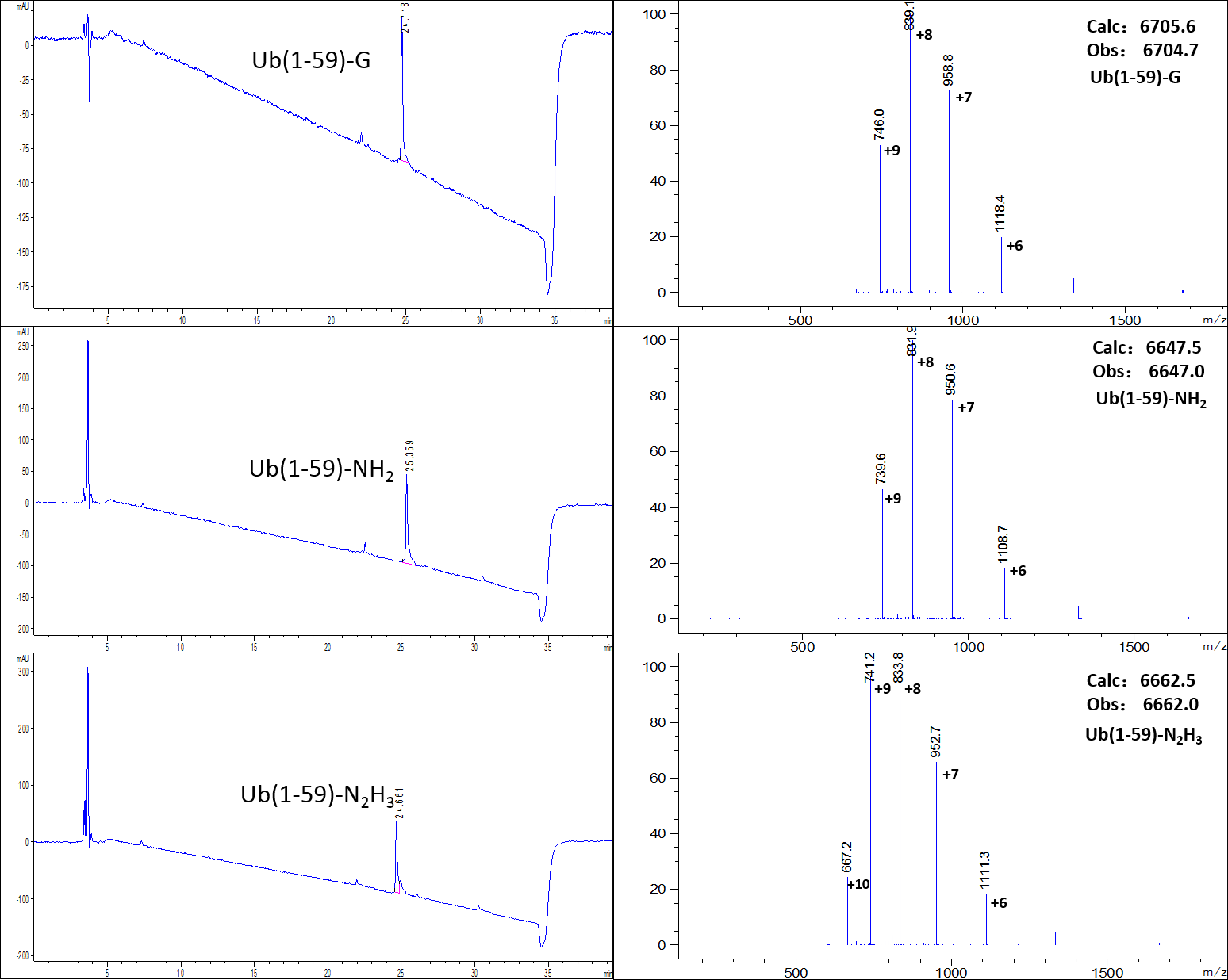


**Supplementary Figure 27.** HPLC traces and mass spectrum of amidation and hydrazidation of Ub(1-59)-G. (top to bottom) Substrate, after amidation, after hydrazidation.


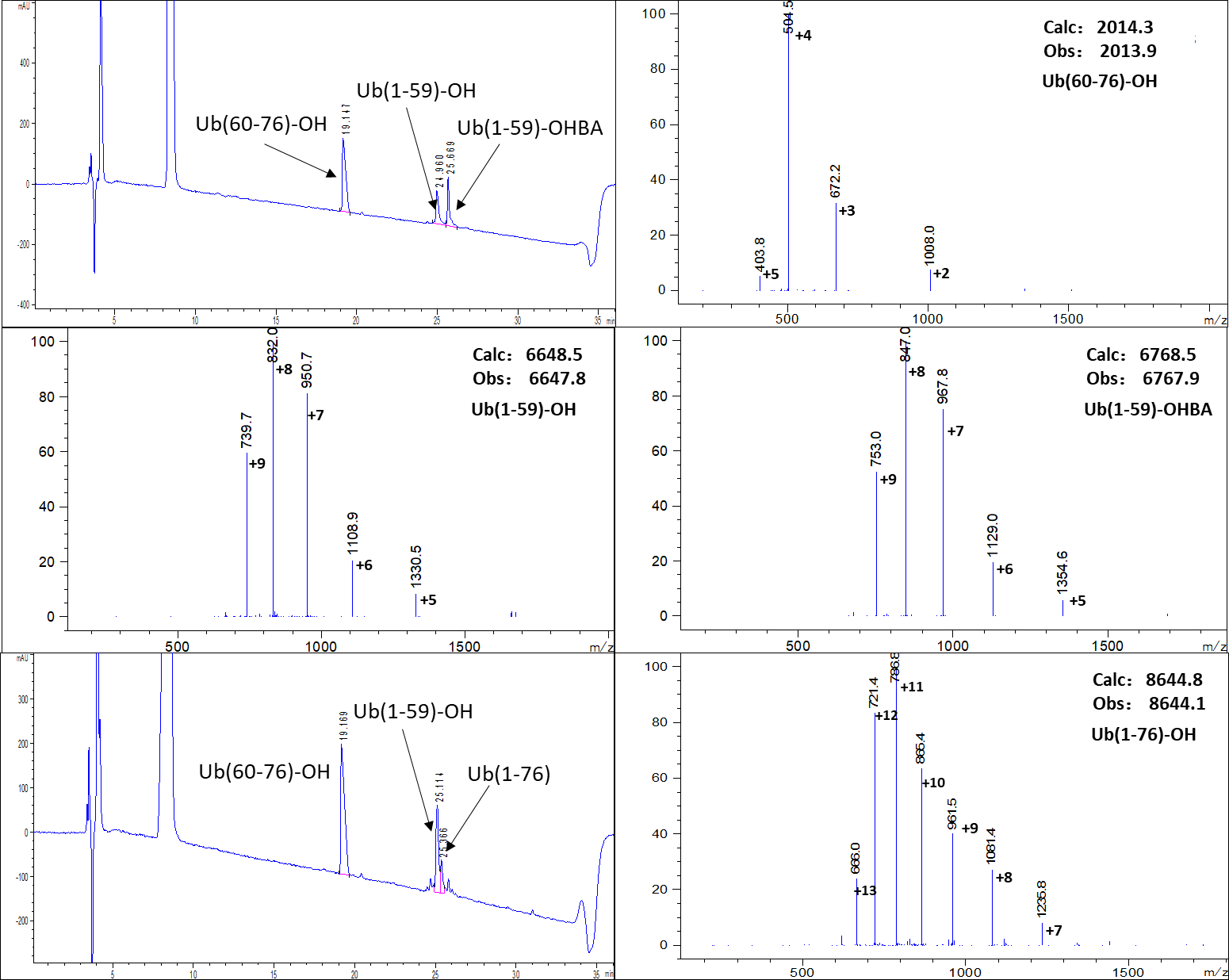


**Supplementary Figure 28.** HPLC traces and mass spectrum of esterification and ligation of Ub(1-59)-N_2_H_3_ and pSer65-Ub(60-76). (top to bottom) after esterification and addition of pSer65-Ub(60-76), 30 min after ligation.

## 4.13 C-terminal modification of NrdH-redoxin by expressed protein ligation

**
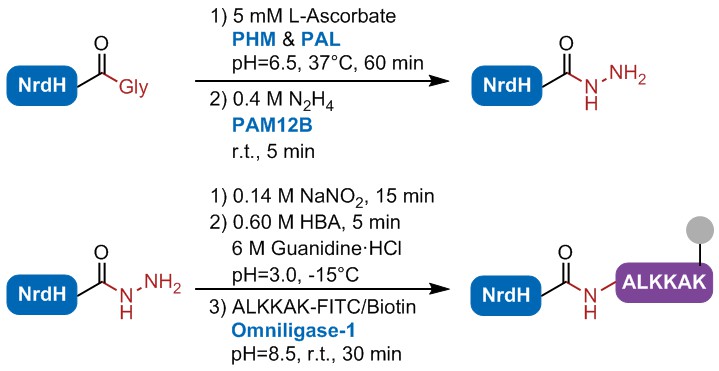
**

**Supplementary Figure 29.** Reaction route of C-terminal modification of NrdH-redoxin.

1. **Reagent Setup**

**ALKKAK-Biotin stock solution (50 mM):**

2.1 mg of ALKKAK-Biotin was added into a 1.5-mL Eppendorf tube. Then 50 μL of deionized H_2_O was added into the tube to dissolve the peptide.

**ALKKAK-FITC stock solution (50 mM):**

2.5 mg of ALKKAK-FITC was added into a 1.5-mL Eppendorf tube. Then 50 μL of deionized H_2_O was added into the tube to dissolve the peptide.

1. **Reaction conditions**

400 µL of NrdH-G (~2.5 mg/mL) was pipetted into a 15-mL culture tube with dual position cap. Then 10 µL of 0.2 M L-ascorbate solution, 20 µL of PHM stock solution (~0.01 equiv.) and 20 µL of PAL stock solution (~0.05 equiv.) were added into the reaction mixture and the tube was incubated in an air bath shaker at 37°C, 180 rpm. 60 min later, 10 µL of 20 M N_2_H_4_ solution and 15 µL of PAM12B stock solution (~0.02 equiv.) were added into the mixture. Hydrazidation reaction was carried out at room temperature for 5 min, then the mixture was injected into preparative HPLC equipments to purify the protein hydrazide.

After lyophilization overnight, the isolated protein was dissolved in 50 µL of 0.2 M phosphate buffer (containing 6 M Gn·HCl, pH 3.0) and precooled at -15°C for 10 min. Then, 20 µL of 0.5 M NaNO_2_ solution was added into the reaction tube and the temperature was kept at -15°C for 15 min. Next, 30 µL of 2 M HBA stock solution was added into the reaction tube and the temperature was kept at -15°C for another 5 min. Afterwards, the tube was removed from the reactor and allowed to warm to room temperature. The pH was adjusted to 8.0~8.5 with 1 M NaOH solution. For ligation, 25 µL of the reaction mixture was transferred into a new 1.5-mL eppendorf tube, and 5 µL of ALKKAK-Biotin/FITC stock solution and 5 µL of Omniligase-1 stock solution were added. The ligation reaction was carried out for 30 min at room temperature.

**Supplementary Table 27.** Final concentration of the peptides and Omniligase-1

|  | NrdH | ALKKAK-Biotin/FITC | Omniligase-1 |
| --- | --- | --- | --- |
| Concentration (mM) | 0.9 | 7.2 | 0.03 |

1. **Results**

Reaction mixtures were analyzed with LC-MS General method A.


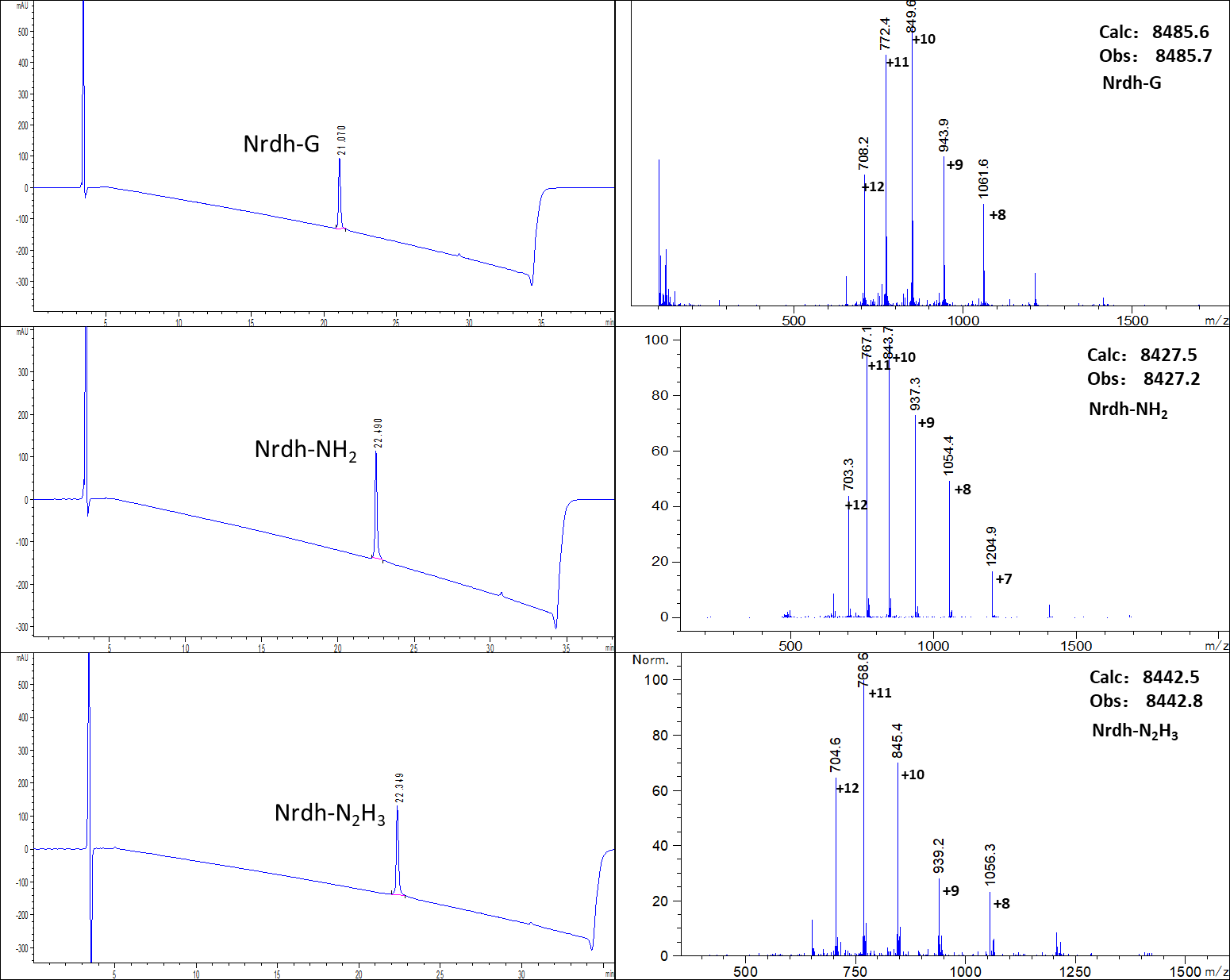


**Supplementary Figure 30.** HPLC traces and mass spectrum of amidation and hydrazidation of NrdH-redoxin. (top to bottom) Substrate, after amidation, after hydrazidation.


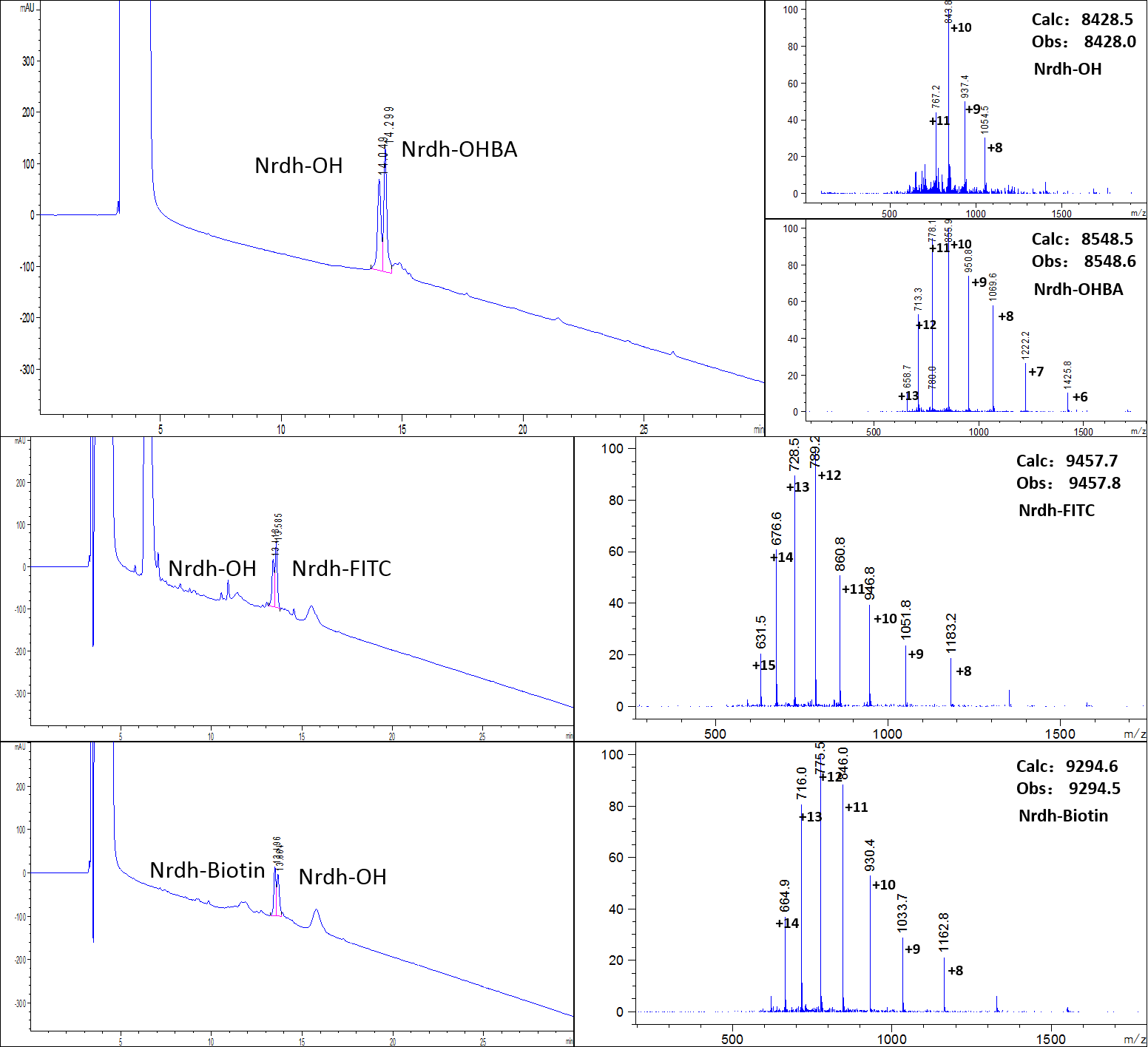


**Supplementary Figure 31.** HPLC traces and mass spectrum of esterification and ligation of NrdH-redoxin and biotin/FITC-modified peptides. (top to bottom) after esterification (pH 8.5), after ligation with ALKKAK-FITC, after ligation with ALKKAK-biotin.

## 4.14 Semisynthesis of Lys56-acetylated mitochondrial heat shock protein 10


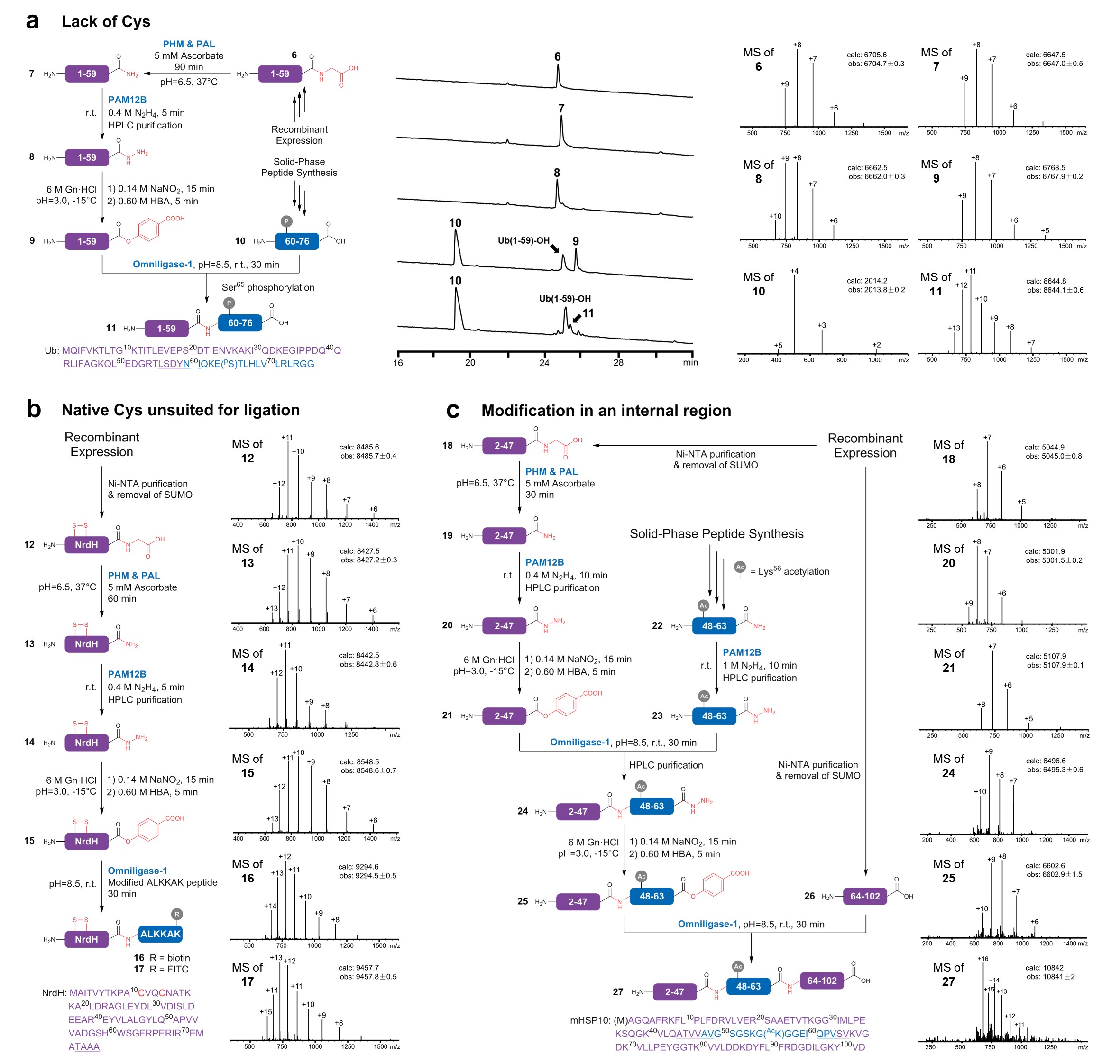


**Supplementary Figure 32.** Reaction route of C-terminal modification of NrdH-redoxin.

1. **Reaction conditions**

12.0 mg of mHSP10(48-63)-NH_2_ was dissolved in 4 mL of deionized H_2_O in a 15-mL culture tube. Then 200 µL of 20 M N_2_H_4_ solution and 60 µL of PAM12B stock solution (~0.001 equiv.) were added into the reaction mixture and the tube was incubated at room temperature for 10 min. mHSP10(48-63)-N_2_H_3_ was purified by HPLC purification, and 11.0 mg of purified product (91% isolated yield) was obtained after lyophilization.

3 mL of mHSP10(2-47)-G solution (~0.4 mg/mL) was pipetted into a 15-mL culture tube with dual position cap. Then 75 µL of 0.2 M L-ascorbate solution, 90 µL of PHM stock solution (~0.02 equiv.) and 30 µL of PAL stock solution (~0.04 equiv.) were added into the reaction mixture and the tube was incubated in an air bath shaker at 37°C, 180 rpm. 30 min later, 70 µL of 20 M N_2_H_4_ solution and 100 µL of PAM12B stock solution (~0.02 equiv.) were added into the mixture. Hydrazidation reaction was carried out at room temperature for 5 min, then the mixture was injected into preparative HPLC equipments to purify the protein hydrazide.

After lyophilization overnight, the isolated protein was dissolved in 75 µL of 0.2 M phosphate buffer (containing 6 M Gn·HCl, pH 3.0) and precooled at -15°C for 10 min. Then, 30 µL of 0.5 M NaNO_2_ solution was added into the reaction tube and the temperature was kept at -15°C for 15 min. Next, 45 µL of 2 M HBA stock solution was added into the reaction tube and the temperature was kept at -15°C for another 5 min. Afterwards, the tube was removed from the reactor and allowed to warm to room temperature. The pH was adjusted to 8.0~8.5 with 1 M NaOH solution, then 0.9 mg of mHSP10(48-63)-N_2_H_3_ (in 20 µL of deionized H_2_O) and 20 µL of Omniligase-1 stock solution were added. The ligation reaction was carried out for 30 min at room temperature. The ligation product was purified by HPLC.

**Supplementary Table 28.** Final concentration of the peptides and Omniligase-1

|  | mHSP10(2-47) | mHSP10(48-63) | Omniligase-1 |
| --- | --- | --- | --- |
| Concentration (mM) | 1.2 | 3.0 | 0.02 |

After lyophilization overnight, the isolated protein (0.3 mg) was dissolved in 30 µL of 0.2 M phosphate buffer (containing 6 M Gn·HCl, pH 3.0) and precooled at -15°C for 10 min. Then, 12 µL of 0.5 M NaNO_2_ solution was added into the reaction tube and the temperature was kept at -15°C for 15 min. Next, 18 µL of 2 M HBA stock solution was added into the reaction tube and the temperature was kept at -15°C for another 5 min. Afterwards, the tube was removed from the reactor and allowed to warm to room temperature. The pH was adjusted to 8.0~8.5 with 1 M NaOH solution, then 1.0 mg of mHSP10(64-102) (in 40 µL of a buffer comprising 50 mM KH_2_PO_4_, pH 7.5) and 10 µL of Omniligase-1 stock solution were added. The ligation reaction was carried out for 30 min at room temperature.

**Supplementary Table 29.** Final concentration of the peptides and Omniligase-1

|  | mHSP10(2-63) | mHSP10(64-102) | Omniligase-1 |
| --- | --- | --- | --- |
| Concentration (mM) | 0.4 | 2.0 | 0.02 |

1. **Results**

Reaction mixtures were analyzed with LC-MS General method A.


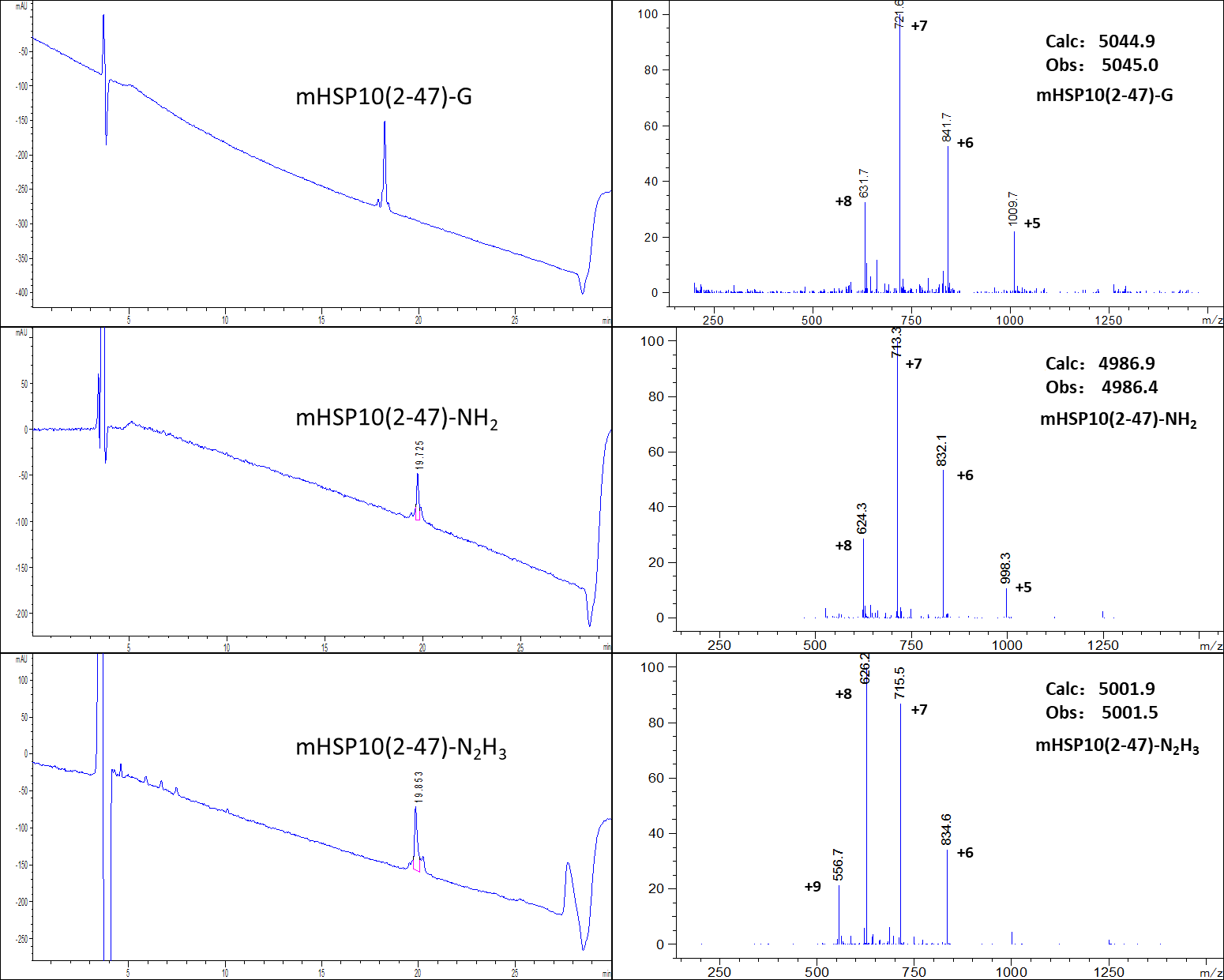


**Supplementary Figure 33.** HPLC traces and mass spectrum of amidation and hydrazidation of mHSP10(2-47)-G. (top to bottom) Substrate, after amidation, after hydrazidation.


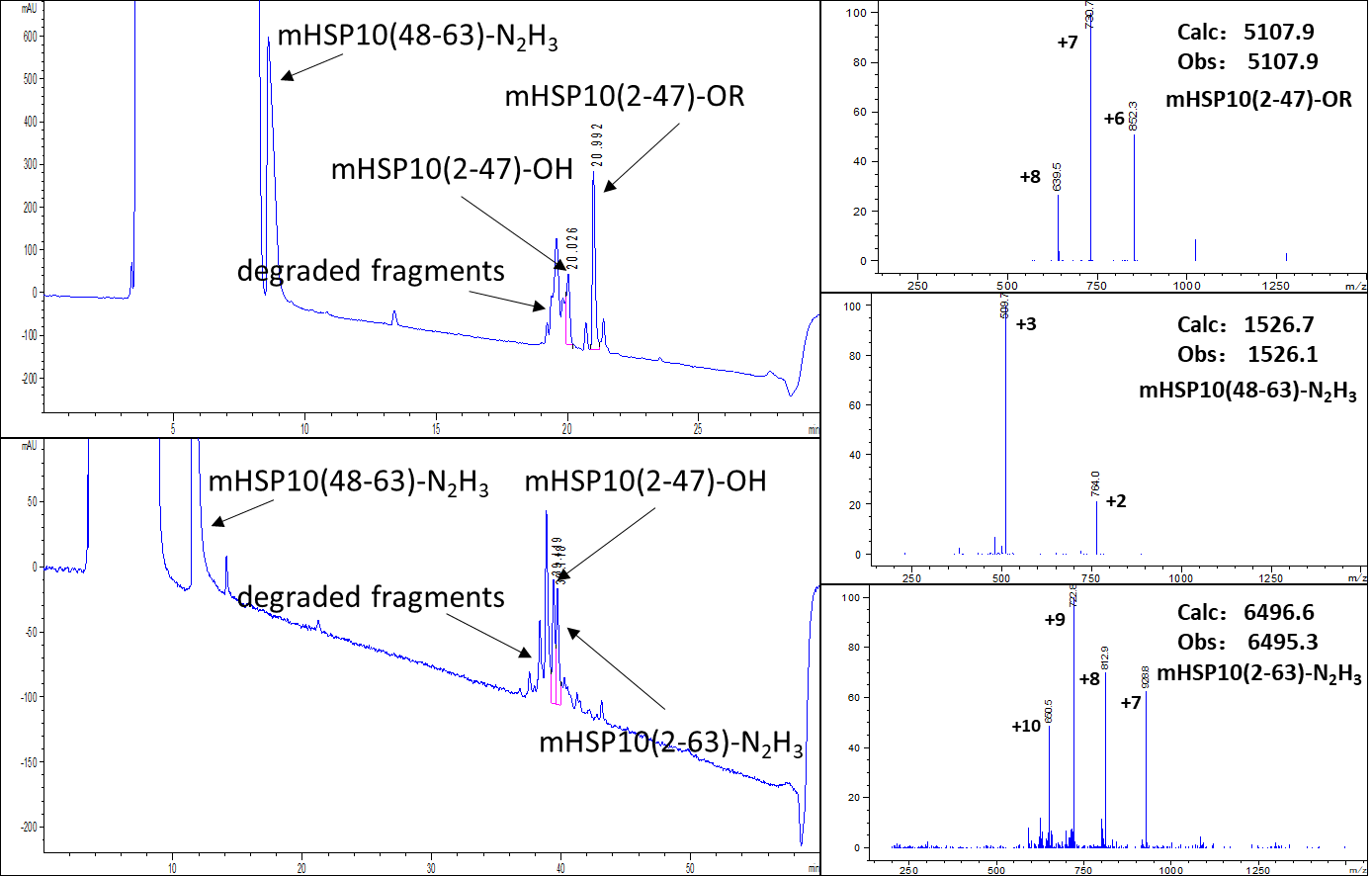


**Supplementary Figure 34.** HPLC traces and mass spectrum of esterification and ligation of mHSP10(2-47)-N_2_H_3_ and mHSP10(48-63)-N_2_H_3_. (top to bottom) after esterification and addition of mHSP10(48-63)-N_2_H_3_, 30 min after ligation. It was observed that mHSP10(2-47) was not stable during both recombinant expression period and HNO_2_ oxidation period.


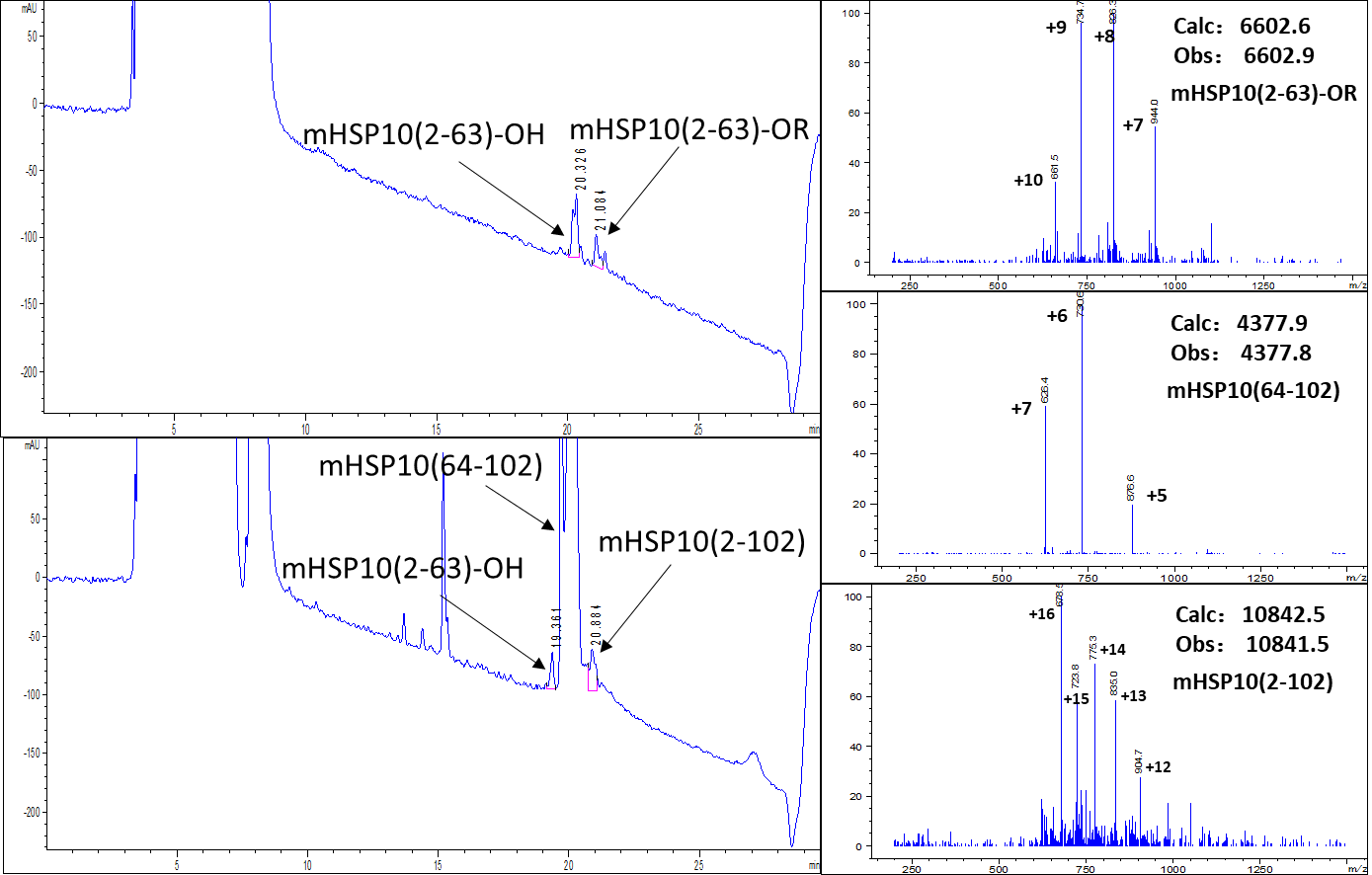


**Supplementary Figure 35.** HPLC traces and mass spectrum of esterification and ligation of mHSP10(2-63)-N_2_H_3_ and mHSP10(64-102). (top to bottom) after esterification, 30 min after ligation.

# References

[1] Zheng, J., Tang, S., Qi, Y., Wang Z. & Liu L. Chemical synthesis of proteins using peptide hydrazides as thioester surrogates. *Nat. Protoc.* **8**, 2483-2495 (2013)

[2] Wu, B., Wijma, H. J., Song, L., Rozeboom, H. J., Poloni, C., Tian, Y., Arif, M. I., Nuijens, T., Quaedflieg, P. J. L. M., Szymanski, W., Feringa, B. L. & Janssen, D. B. Versatile peptide C-terminal functionalization via a computationally engineered peptide amidase. *ACS Catal.* **6,** 5405-5414 (2016).

[3] Bauman, A. T., Ralle M. & Blackburn N. J. Large scale production of the copper enzyme peptidylglycine monooxygenase using an automated bioreactor. *Protein Expr. Purif.* **51,** 34-38 (2007).

[4] Fang, G., Li, Y., Shen, F., Huang, Y. C., Li, J., Lin, Y., Cui, H. K. & Liu, L. Protein chemical synthesis by ligation of peptide hydrazides. *Angew. Chem. Int. Ed.* **50,** 7645-7649 (2011).
